# Cortical astrocytes develop in a plastic manner at both clonal and cellular levels

**DOI:** 10.1038/s41467-019-12791-5

**Published:** 2019-10-25

**Authors:** Solène Clavreul, Lamiae Abdeladim, Edwin Hernández-Garzón, Dragos Niculescu, Jason Durand, Sio-Hoï Ieng, Raphaëlle Barry, Gilles Bonvento, Emmanuel Beaurepaire, Jean Livet, Karine Loulier

**Affiliations:** 1 Sorbonne Université, INSERM, CNRS, Institut de la Vision, 17 rue Moreau, Paris, F-75012 France; 2 Laboratory for Optics and Biosciences, Ecole polytechnique, CNRS, INSERM, IP Paris, 91128 Palaiseau, France; 3 Commissariat à l’Energie Atomique et aux Energies Alternatives (CEA), Département de la Recherche Fondamentale, Institut de biologie François Jacob, Molecular Imaging Research Center (MIRCen), CNRS UMR 9199, Université Paris-Sud, Université Paris-Saclay, 18 Route du Panorama, Fontenay-aux-Roses Cedex, 92265 France; 4 Present Address: Institut des Neurosciences de Montpellier, INSERM U1051, 80 Avenue Augustin Fliche, 34091 Montpellier Cedex 5, France

**Keywords:** Glial development, Gliogenesis

## Abstract

Astrocytes play essential roles in the neural tissue where they form a continuous network, while displaying important local heterogeneity. Here, we performed multiclonal lineage tracing using combinatorial genetic markers together with a new large volume color imaging approach to study astrocyte development in the mouse cortex. We show that cortical astrocyte clones intermix with their neighbors and display extensive variability in terms of spatial organization, number and subtypes of cells generated. Clones develop through 3D spatial dispersion, while at the individual level astrocytes acquire progressively their complex morphology. Furthermore, we find that the astroglial network is supplied both before and after birth by ventricular progenitors that scatter in the neocortex and can give rise to protoplasmic as well as pial astrocyte subtypes. Altogether, these data suggest a model in which astrocyte precursors colonize the neocortex perinatally in a non-ordered manner, with local environment likely determining astrocyte clonal expansion and final morphotype.

## Abstract (web summary)

Previous studies on astrocyte development have led to controversial results due to a lack of pertinent tools. Here, authors analyze large numbers of astrocyte clones generated by nearby cortical progenitors using the MAGIC Markers strategy and ChroMS 3D imaging, and show that clonally-related astrocytes organize in a non-stereotyped manner and that cortical astrocyte subtypes are not intrinsically specified.

## Introduction

Brain functions rely on the efficient cooperation of neurons and glial cells to shape and operate neural circuits. In the cerebral cortex, the center of higher cognitive functions in mammals, numerous studies have deciphered neuronal contributions to cortical circuits, and described how excitatory neurons issued from dorsal progenitors migrate in a stereotyped manner to reach their final location^1,2^. However, the development of glial cells generated by these same progenitors, in particular astrocytes, remains far less understood, despite their many crucial roles in brain circuit function and formation^3–5^. Beside their essential function at the blood brain barrier where they regulate nutrient uptake and blood flow, astrocytes also play critical roles in neuronal survival and synaptic function, recycling neurotransmitters and buffering potassium ions^6^. Other studies have also uncovered their active participation in synapse formation and pruning^7–9^.

Normal resting astrocytes present two paradoxical characteristics: closely apposed to their neighbors, they tile the gray matter and form a seemingly uninterrupted tridimensional array^10,11^, suggesting that they belong to a homogenous type; at the same time however, it is increasingly recognized that they constitute a heterogeneous population at the morphological, molecular, and functional levels^5,12–20^. Considerable divergence exists among astrocyte subtypes such as the fibroblast-like glia limitans of the pial surface^21,22^ and the protoplasmic astrocytes (PrA) of the cortical parenchyma, which themselves display layer-related differences^15,23^. Former studies have established that cortical astrocytes originate from a fraction of delaminating embryonic progenitors of the dorsal telencephalon^24–26^ (see ref. ^27^ for review), while a possible additional contribution of the postnatal germinal zones is disputed^28–31^. Following the colonization of the cortical parenchyma by astrocyte precursors, local proliferation considerably augments their numbers during early postnatal life^31^. Despite these advances concerning cortical astrogenesis, a consensus has failed to emerge on the following questions: whether cortical astrocyte subtypes are produced by predetermined progenitors, as suggested by some studies^22,32^, or from nonspecified progenitors generating similar astroglial descent which locally adapt to their substrate, as recently shown in the Drosophila ventral nerve cord^33^; how stereotyped the composition and distribution of the descent of these progenitors is; and how they primarily invade the neocortex.

Here, using the MAGIC Markers (MM) combinatorial labeling strategy^34^ together with a new large volume chromatic multiphoton serial microscopy technique (ChroMS)^23^, we performed multiclonal lineage tracing in the mouse cerebral cortex and analyzed large numbers of astrocyte clones issued from nearby cortical progenitors, marked prior to the start of gliogenesis and tracked over long periods of time. We show that cortical astrocyte clones display extensive variability concerning their structural organization, number of cells and subtype composition, with a significant increase in clonal size in the upper layers of the cortex. Furthermore, the dispersion of these clones diverges from the columnar patterns observed for neurons^24,35^. In addition, we demonstrate that the cortical astrocyte network develops through a dynamic phase of proliferation accompanied by sister cell spacing, and a maturation phase where morphological complexity and volume increase at the single cell level, while proliferation and spatial dispersion progressively abate. Moreover, we uncover a significant astroglial contribution of early postnatal progenitors in addition to prenatal delaminated apical progenitors, seeding the cortical parenchyma in a scattered manner and capable of generating both protoplasmic and pial astrocyte subtypes. Altogether, these results indicate that cortical astrocyte network development relies on plastic rather than stereotyped clonal units derived from progenitors that colonize the cortical wall in a largely disordered manner. These clones differentially expand and produce astrocytes that can adopt different fates, suggesting that their final characteristics are determined by interactions with their neighbors and/or environment.

## Results

### Multiplexed 3D imaging of cortical astrocyte clones

**Fig. 1:**
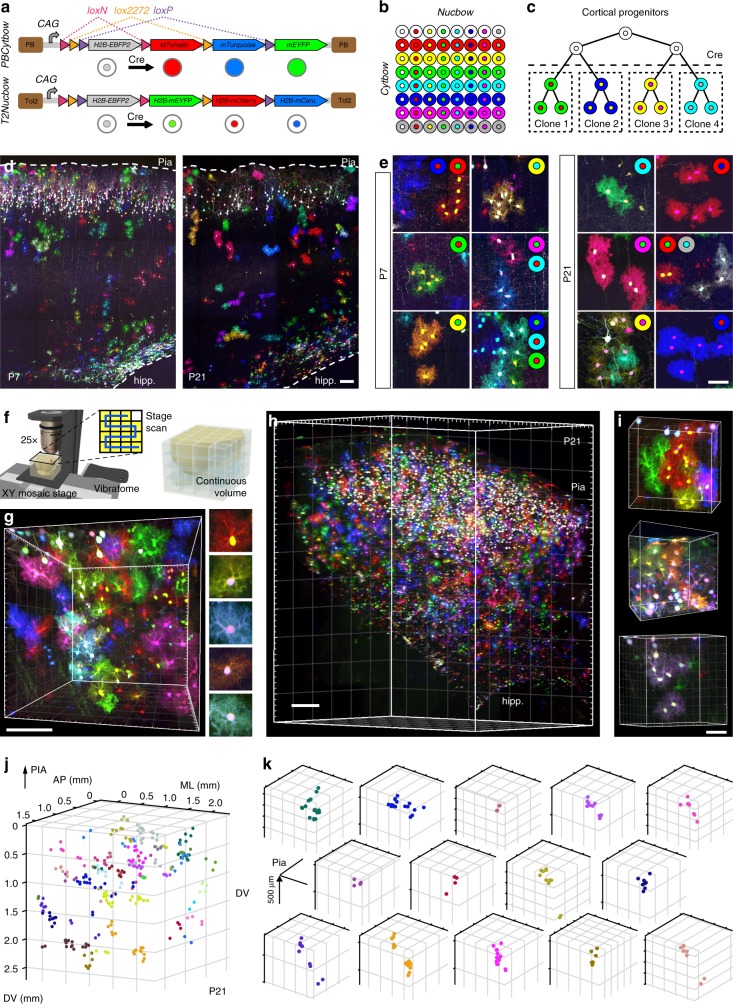
MAGIC Markers associated with ChroMS microscopy reveal astrocyte clonal patterns diversity. **a** MAGIC Markers (MM) constructs for genomic combinatorial labeling: transgenes express a nuclear EBFP2 by default under the control of a *CAG* promoter. Three recombination possibilities created by alternating pairs of incompatible *lox* sites each trigger expression of a distinct FP (mCerulean/mTurquoise2, mEYFP, or tdTomato/mCherry) in specific subcellular compartments: cytoplasm (*Cytbow*) or nucleus (*Nucbow*). 5′ and 3′ Tol2 (T2) or piggyBac (PB) transposition sequences frame the transgenes. **b** 63 theoretical color combinations are possible in cells containing 1–3 copies of both ^*PB*^*Cytbow* and ^*T2*^*Nucbow*. **c** MM are used to label astrocyte clones arising from distinct cortical progenitors. **d** E15 IUE of MM and self-excising Cre (SeCre) along with PB and Tol2 transposases labels astrocytes at P7 and P21 in all layers of the mouse cerebral cortex. **e** Rare nuclear/cytoplasmic color combinations allow the distinction of astrocyte clones. **f** Chromatic serial multiphoton (ChroMS) microscopy relies on the integration of trichromatic two-photon excitation by wavelength mixing with automated blockface imaging. Adapted from the graphics used in Abdeladim et al., Nat. Commun. 2019 Apr 10;10(1):1662, and licensed under a Creative Commons Attribution 4.0 International License, http://creativecommons.org/licenses/by/4.0/. **g**, **h** ChroMS microscopy allows high-resolution 3D imaging of large volumes of MM-labeled cortex, and visualization of astrocyte clones spatial arrangement (**i**, **j**). **k** Examples of single clones plotted in (**j**) presenting various sizes and spatial distributions. *Hipp.* hippocampus, *DV* dorsoventral axis, *AP* anteroposterior axis, *ML* mediolateral axis. Scale bars: 100 (**d**, **g**, **i**); 200 (**h**); 50 (**e**) µm

To determine how the astroglial tridimensional matrix is established during corticogenesis, we tracked astrocyte development with the MM strategy^34^, which enabled us to analyze the clonal descent of multiple individual embryonic progenitors marked with combinations of fluorescent proteins (FP). In this approach, astrocyte clones are labeled by in utero electroporation (IUE) of two transposable Brainbow transgenes capable of genomic integration under the action of the piggyBac (PB) or Tol2 (T2) transposase (^*PB*^*Cytbow* and ^*T2*^*Nucbow*). Following recombination by a self-excising Cre recombinase (SeCre), they stochastically express one of three FP (cyan, yellow or red) in the cell cytoplasm or nucleus (Fig. 1a, b). Expression from the broadly active *CAG* promoter avoids biases associated with unequally regulated astrocyte markers such as GFAP^19,36^. We delivered the MM plasmids (^*PB*^*Cytbow* and ^*T2*^*Nucbow)* along with transposase-expressing and SeCre plasmids to cortical progenitors at embryonic day (E)15, prior to gliogenesis, to permanently mark these cells and their descent and study the spatial organization of astrocyte clones and its evolution during postnatal brain development (Fig. 1c–e, Supplementary Fig. 1a, b). Inventory of cytoplasmic and nuclear RGB color labels in 57,535 astrocytes from 12 analyzed animals and calculation of their frequency enabled us to define criteria for astrocyte clone identification based on: (i) rare combinatorial labels (<2% of labeled astrocytes) resulting from the coexpression of ≥1 copy of ^*PB*^*Cytbow* and ^*T2*^*Nucbow* transgenes (Supplementary Fig. 1c–e), ii) final color display and (iii) a maximal spatial distance among sister cells <600 µm (Supplementary Fig. 1f–h, see Methods). Based on these criteria, 36–160 astrocyte clones were identified per brain.

To analyze in an unbiased manner the spatial distribution and structure of astrocyte clones during the three first postnatal weeks, we performed tridimensional multicolor volume imaging of brains labeled with MM using a new ChroMS microscopy approach^23^ (Fig. 1f–i). This enabled us to reconstruct large volumes (≥8 mm^3^) of cortical parenchyma at P7 and P21 stages with near-micrometric resolution, thus giving us access to the spatial position and tridimensional arrangement of each labeled clone, with all their astroglial cells accounted for (Fig. 1j, k).

### Astrocyte clones show variable and intermixed organization

**Fig. 2:**
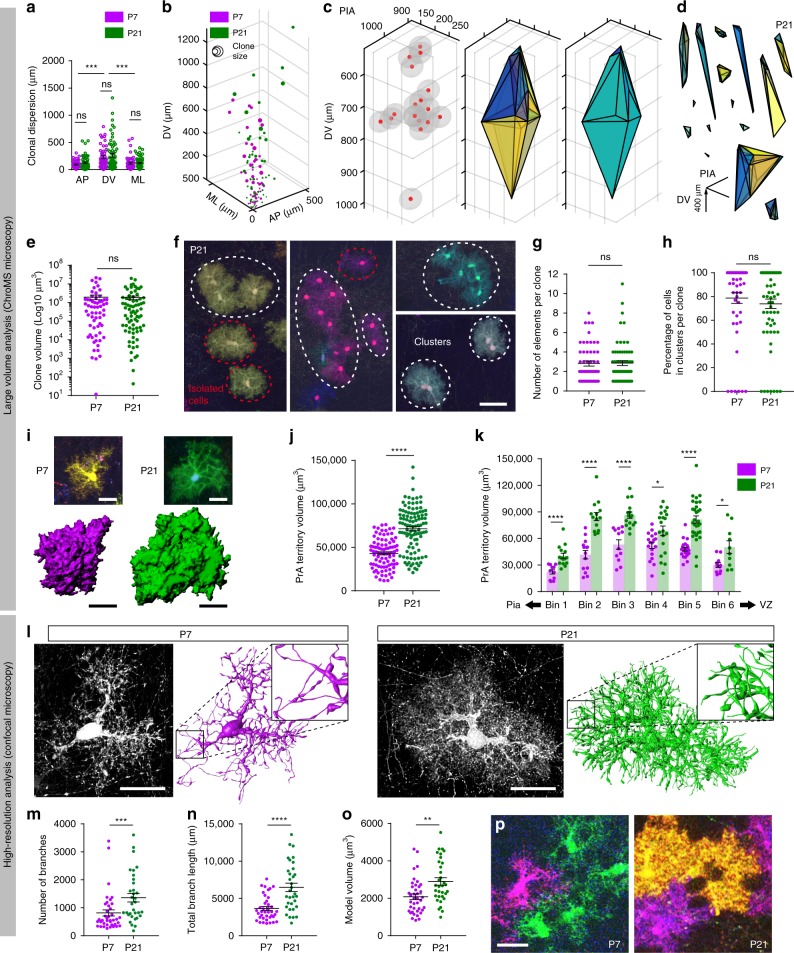
Clonal organization of astrocytes remains stable from P7 to P21 while their arbor expands and complexifies. (**a**, **b**) 3D analysis reveals variable PrA clonal dispersion up to 1.25 and 0.5 mm along the DV and ML/AP axis, but no difference between P7 and P21 (*n* = 113). **c** Example of a 17-cell clone where red represents nuclei and gray the astrocyte territory (left) along with Delaunay Triangulation (DT, middle) and Convex Hull extraction (CH, right). **d** DT of random PrA clones highlights their distinct shapes. **e** CH analysis shows high diversity of the volume covered by clones, but stability of its distribution between P7 and P21. **f** PrA clones were automatically separated into clusters of apposed cells or isolated cells (elements) using their *XYZ* coordinates and astrocyte mean diameter + s.d. at each stage. **g** On average PrA clones are composed of 2.8 disconnected elements and this arrangement is stable from P7 to P21. **h** Totally, 76.2% of PrA belong to clusters and this proportion is stable from P7 to P21. **i** Segmentation of color-isolated astrocytes from ChroMS images at P7 (left) and P21 (right). **j**, **k** Segmented astrocyte territorial volume increases by 65.7% from P7 to P21 (**j**) and this expansion occurs in the entire cortical parenchyma, here divided in six equivalent bins (Bin 1 = pial surface, **k**). **l** High resolution reconstruction of astrocyte arborizations at P7 and P21 reveals a significant increase in branch number (**m**), total branch length (**n**), and volume of the model (**o**). **p** Close-ups of neighboring PrA labeled with distinct colors (single optical section) show incomplete filling of cortical space by P7 PrA compared to P21 PrA. *DV* dorsoventral axis, *AP* anteroposterior axis, *ML* mediolateral axis, *VZ* ventricular zone. Graph values indicate means ± s.e.m. Kruskal–Wallis associated with Dunn’s multiple comparisons (**a**) and Mann–Whitney (**e**, **g**, **h**, **j**, **k**, **m**–**o**) statistical tests have been performed. *, ***, **** indicate *p* value < 0.05, <0.0005, <0.0001, respectively. *N* = 4 (**a**, **e**, **g**, **h**, **j**, **k**) and 9 (**m**–**o**) animals. Scale bars f: 50 (**f**), 20 (**i**, **l**, **p**) µm

Tridimensional mapping with ChroMS microscopy revealed a high variability of PrA clones in terms of both their 3D spatial dispersion and volume at P7 and P21. We observed that on average, PrA clones were composed of 7.1 ± 0.6 (s.e.m.) cells at P7 and 5.9 ± 0.5 cells at P21 (nonsignificant difference) but with a high s.d. (respectively 4.6 and 4.1). They dispersed over several dozen microns on all three axes with a significant wider spread along the dorsoventral (DV) axis (Fig. 2a, b), and presented no preferential location in specific cortical layers. Further analysis showed that although the principal axis of the clones exhibited a preferential radial orientation, many of them deviated from this behavior (Supplementary Fig. 2a–c). While probing the spatial organization and dispersion of PrA clones using cell coordinates and Delaunay triangulation analysis (Fig. 2c, Supplementary Fig. 2d), we found that PrA clones could be composed of tightly linked clusters of cells, but also of multiple spatially separated elements (clusters or isolated cells). Clones could scatter over extended volumes (up to 1.86 × 10^6^ µm^3^, i.e., more than 20 times the volume of individual astrocyte domains, Fig. 2d, e, Supplementary Fig. 2e, f), and there was hence significant intermixing with cells of neighboring clones. The spatial arrangement and volume of the clones were highly variable, at P7 as well as P21 (Fig. 2d, e, Supplementary Fig. 2f, g, also see Supplementary Dataset showing the 3D layout of each clone). Yet at both stages, we found that PrA clones were composed of a similar number of disconnected elements (2.8 ± 0.3 at P7 vs. 2.9 ± 0.3, at P21, Fig. 2f, g), and the proportion of clustered clonal PrA remained stable (78.7% ± 4.5 at P7 and 73.8% ± 4.0 at P21, Fig. 2h). The absence of variation of these two parameters between P7 and P21 suggests that no major cellular rearrangement takes place among PrA clones during this time period.

Thus the local proliferation of astrocyte precursors located in the cortical parenchyma, recognized as the main source of cortical astrocyte production during the first postnatal week^31^, is not fully cohesive, sister cells being able to separate and intermix with neighboring clones, but the spatial arrangement of the clones after this period seems largely stable.

### Astrocyte arbors expand and complexify as they mature

To understand how the astroglial anatomical network develops after P7 in absence of proliferation, we analyzed the morphology of individual somatosensory PrA imaged by ChroMS microscopy through semiautomated segmentation (Fig. 2i, Supplementary Fig. 3a, b). We observed that at an individual level, the volume of neural tissue that they covered (termed domain or territorial volume^10,15,37^) increased by 65.7% between P7 and P21 (Fig. 2j), close to the 61% increase observed previously in the primary visual cortex (V1)^38^. This age-dependent expansion of PrA was seen in all cortical layers (Fig. 2k and Supplementary Fig. 3c). To characterize the changes in astrocyte arbor that accompany the expansion of their outer frontiers, we reconstructed in situ the detailed morphology of individual MM-labeled somatosensory cortical astrocytes from high N.A. diffraction-limited confocal microscopy images acquired in brain sections using a new semiautomated tracing and segmentation pipeline (see Methods). This enabled us to analyze for the first time how the arborization of normal resting PrA evolves in situ during cortical development. Our results revealed a strong increase in astrocyte process complexity, with a significant rise in the number and length of arbor branches between P7 and P21 (Fig. 2l–o and Supplementary Fig. 3d–g), accompanied by a 6.0% and 5.7% increase in the density of branches and endings, respectively, within the astrocyte domain between P7 and P21. These data explain why astrocyte coverage of the neuropil appears denser at P21 than at P7 (Fig. 2p). Thus, in situ, astrocyte arborization complexifies between P7 and P21, at a time when the relative arrangement of astrocytic soma does not further evolve.

### Non-cohesive clones arise from early spatial dispersion

**Fig. 3:**
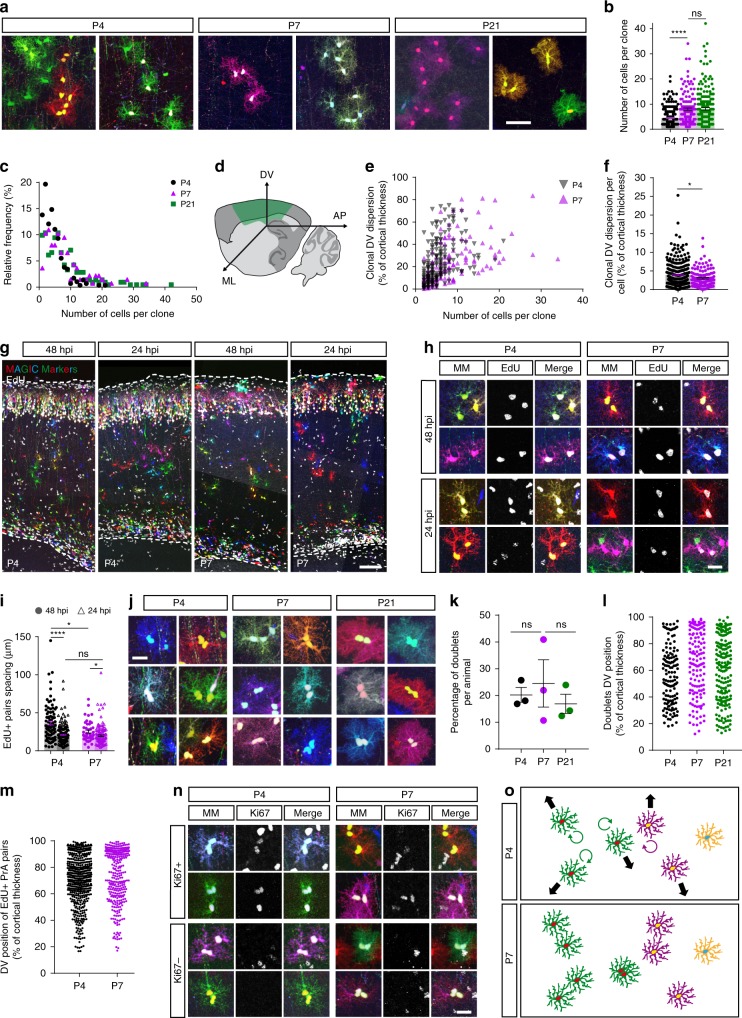
Clonal expansion based on proliferation and spacing of sister astrocytes lessens during development. **a** PrA clones of various sizes at P4, P7, and P21 after MM IUE at E15. **b** PrA clones issued from cortical progenitors targeted at E15 grow in average from 4.5 ± 0.19 cells at P4 to 7.9 ± 0.51 cells at P7 and 8.1 ± 0.47 cells at P21. **c** PrA clones exhibit various sizes at P4, P7, and P21, with clones composed of 1 to 42 cells. **d** Schematic view of DV, ML, and AP dispersion on serial sagittal sections. **e** DV dispersion of PrA clones ≥2 cells as a function of clone size is broad and variable. **f** DV dispersion per cell expressed as % of cortical thickness (DV clonal dispersion/ clone size) decreases from P4 to P7. **g** P4 and P7 sagittal sections co-labeled by MM IUE at E15 and EdU injection 48 or 24 h prior to analysis. Proliferating PrA are found throughout the entire cortical wall. **h** Examples of EdU + PrA astrocyte identified as sister cells with color markers. **i** Distance between EdU + paired PrA decreases from 48 to 24 h postinjection and from P4 to P7. **j** Sister cells closer than astrocyte nucleus mean size (6 µm) form doublets. **k** Doublets of PrA were found at P4, P7, and P21 in stable proportions (20%). **l** Doublets relative DV positioning (in % of cortical thickness, see Supplementary Fig. 5e) shows that they occur in the entire cortical parenchyma from P4 to P21 whereas EdU + astrocyte pairs are located mostly in upper cortical layers (**m**). **n** Doublets found at P4–P7 are not all comprised of Ki67+ cycling cells. **o** Model of astrocyte clonal maturation through densification between P4 and P7. DV dorsoventral, ML mediolateral, AP anteroposterior. Graph values indicate means ± s.e.m. One-tailed Mann–Whitney (**f**) and Kruskal–Wallis associated with Dunn’s multiple comparisons (**i**, **k**) statistical tests have been performed. **** indicates *p* value < 0.0001. *N* = 6 (**b**), 10 (**i**), 9 (**k**) animals. Scale bars: 50 (**a**), 100 (**g**), 20 (**h**, **j**, **n**) µm

To understand how the scattered arrangement of astrocyte clones observed above develops, we explored the expansion dynamics of sister cells. This analysis was led in parallel to the ChroMS mapping presented above, by electroporating MM at E15 and analyzing astroglial clones displaying rare color markers at P4, P7, and P21 on serial brain sections (Fig. 3a). Clone sizes measured with the two methods at P7 and P21 were comparable, confirming the stability of the network between these stages. However, a twofold increase was observed from P4 to P7 (4.5 ± 0.19 and 7.9 ± 0.51 cells, respectively; Fig. 3b), reflecting the doubling of astrocyte population that occurs during the first postnatal week^26,30,31^. Already at P4, these clones were highly variable in size (from 1 to 21 cells, s.d. = 3.2), suggesting unstereotyped expansion during development (Fig. 3c), and displayed variable spatial arrangements that prefigured those observed at P7–P21 (columns, clusters, or mix of both, Figs. 1 and 2, Supplementary Figs. 2 and 4a). Indeed, analysis of the spatial dispersion of PrA clones at P4 and P7, during and at the end of their proliferative period, showed considerable heterogeneity along both the DV (Fig. 3d, e) and mediolateral (ML, Supplementary Fig. 4b) axes, with clones that could for instance form tight clusters or spread over most of the cortical wall. In addition, we observed that the relative dispersion of the clones (spread of clones divided by total number of sister cells) did not increase between P4 and P7, showing that astrocyte local proliferation during that period was not accompanied by a wide scattering of clonally related cells. Instead, cells densified over time, as indicated by a reduced relative dispersion (Fig. 3f and Supplementary Fig. 4c). To better understand this process, we analyzed sister cell proliferation within clones. We injected EdU either 48 or 24 h prior to analysis at P4 and P7 (Fig. 3g, h) in order to determine whether recently divided sister astrocytes stay together as pairs of closely apposed cells, long considered features of astrocyte proliferation^31^, or move away from each other over time. We found that sister cells labeled with EdU 48 h prior to sacrifice were located further apart than those marked 24 h in advance, and this both at P4 and P7 (Fig. 3i), showing that their cell bodies moved apart from each other over time. However, markedly smaller spacing (on average inferior to the diameter of astrocyte domains) was observed at P7, indicating that network expansion slowed down as development proceeded (Fig. 3i). Interestingly, we observed that 20% of MM-labeled cells belonged to sister cell “doublets” separated by a distance inferior to the mean size of astrocyte nuclei (6 µm), irrespective of the developmental stage (Fig. 3j, k). In addition, whereas these doublets were not preferentially located in specific cortical layers (Fig. 3l), proliferating sister cells labeled with EdU were mostly found in the upper cortical layers (Fig. 3m). This, added to the drastic drop in the proliferation rate during the first postnatal week (almost null at P21, see ref. ^31^), and the observation of astrocyte doublets negative for the proliferation marker Ki67 (Fig. 3n), led us to conclude that astrocyte doublets are not a reliable indicator of recent divisions but can result from the stalling of astrocyte expansion in late postnatal development.

Altogether, these data suggest that astrocyte clone expansion, after an early phase of non-cohesive proliferation that generates the dispersed patterns observed at P4, evolves towards increasingly cohesive cell divisions, leading to densification of the clones between P4 and P7. Newly generated sister cells move away from their siblings at a distance that diminishes over time and the final matrix architecture reflects the progressive stalling of this process, with numerous astrocytes remaining as doublets (Fig. 3o).

### Single progenitors can produce distinct astrocyte subtypes

**Fig. 4:**
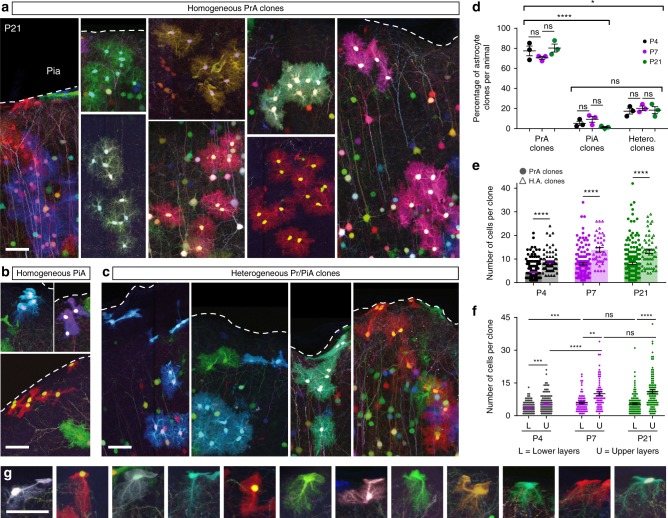
Individual cortical progenitors can generate both protoplasmic and pial astrocytes. **a–c** Examples of homogeneous clones of PrA (**a**) or PiA (**b**) and heterogeneous clones containing both astrocyte types (**c**). **d** PrA, PiA, and heterogenous clones were found in stable proportions (respectively 76%, 5%, and 19% of clones) throughout postnatal development (P4–P7–P21), with a significant majority of homogeneous PrA. **e** Heterogeneous astrocyte clones comprise significantly larger number of cells than homogeneous PrA clones. **f** PrA clones whose barycenter is located in the upper half of the cortex (U) are larger than those in lower layers (L) at P4, P7, and P21. Clone barycenter is calculated using the mean of sister cells relative DV positions. **g** Examples of intermediate morphologies between PiA and PrA observed at P21, presenting both fibroblast-like features at one extremity and bushy ramifications at the other extremity. Graph values indicate means ± s.e.m. Kruskal–Wallis associated with Dunn’s multiple comparisons statistical tests have been performed. **** indicates *p* value < 0.0001. *N* = 9 animals. Scale bars: 50 µm

Next, we investigated the subtype composition of astrocyte clones (Fig. 4, Supplementary Fig. 5a, b). Three types of clones could be unambiguously identified based on the distinct morphologies of the cells that they comprised: (i) homogeneous PrA clones (Fig. 4a), (ii) homogeneous pial astrocyte (PiA) clones (Fig. 4b) found at the pial surface, and (iii) heterogeneous clones containing both PrA and PiA (Fig. 4c). The vast majority of labeled clones were composed of only PrA (76%), whereas exclusively PiA clones represented a minority (5%) and 19% contained both PrA and PiA (Fig. 4d), with most PiA (>80%) belonging to heterogeneous clones. This differed from previous work that supported the existence of distinct lineages^22^ for these two astrocyte subtypes playing specific roles in the adult brain^17,39,40^. Even as the astrocyte population doubled between P4 and P7–P21, the proportion of each type of clones remained stable (Fig. 4d), and both PrA and PiA Ki67+ cycling astrocyte populations were reduced by half (Supplementary Fig. 5c), indicating that the proliferation decline occurring during this period affects these two subtypes equally. Interestingly, however, pulse labeling with EdU at P3 labeled a larger proportion of PiA (23%) compared to PrA (9%) at P4, suggesting that the two subtypes may follow different mitotic behavior at earlier stages (Supplementary Fig. 5d). Analysis of clone size showed that heterogeneous clones were larger than homogeneous PrA ones (Fig. 4e). However, the size of both homogeneous and heterogeneous clones significantly increased along the radial axis (i.e., clones found in the upper part of the cortex were larger than those located in lower layers, Fig. 4f, Supplementary Fig. 5e, f), suggesting that their position and not their subtype composition was the main factor influencing their capacity to expand. These results are consistent with the notion that cortical astrocytes arise from a unique type of progenitor and that clonally related cortical astrocytes have the ability to adopt different morphotypes according to their location in the cerebral cortex, and are therefore not fate-restricted. Strikingly indeed, we found at the pial surface multiple cases of cells displaying an intermediate morphology that combines the fibroblast-like extension of PiA and the bushy ramifications of PrA (Fig. 4g).

### Pre- and postnatal progenitors supply the astrocyte matrix

**Fig. 5:**
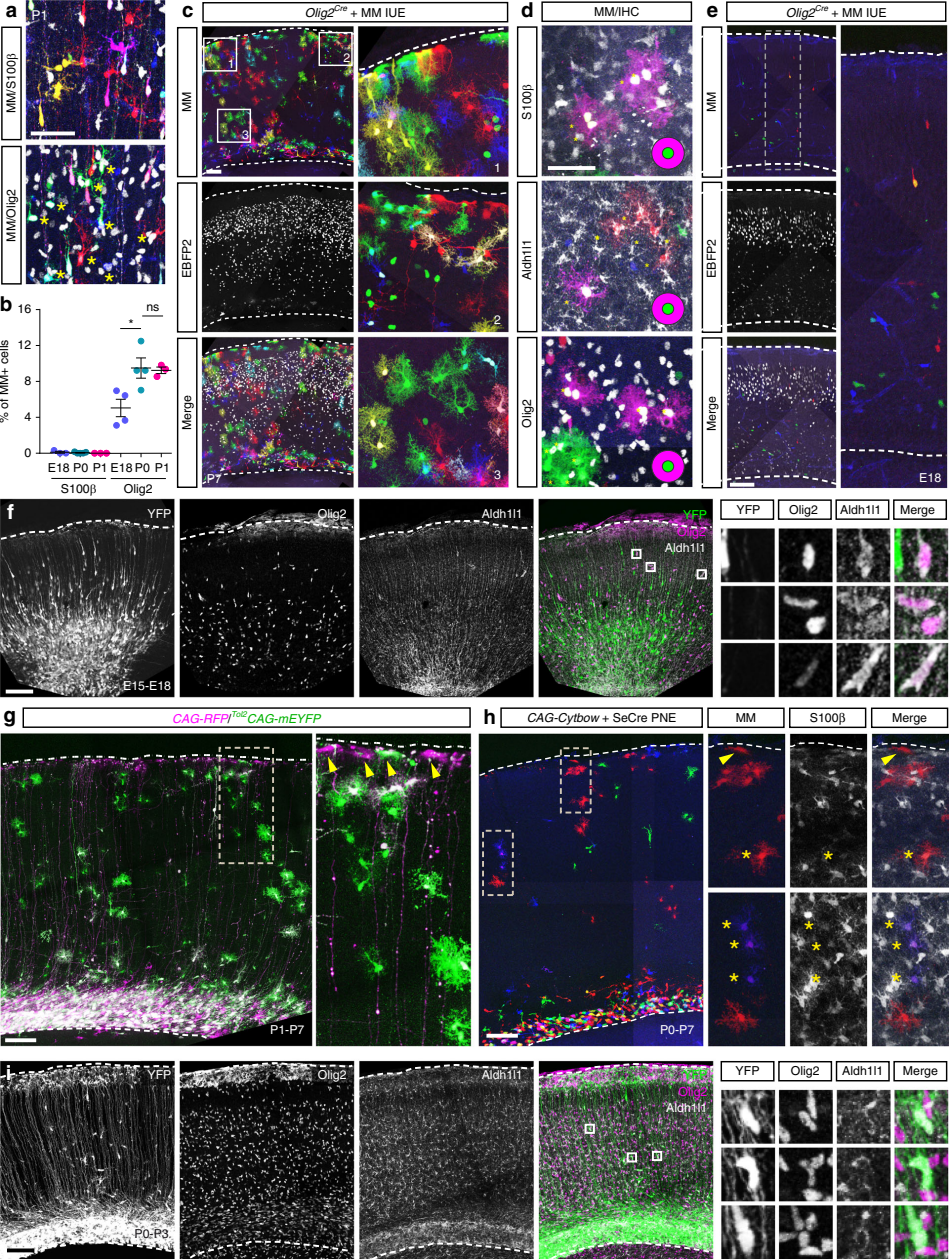
Cortical astrocytes arise from Olig2+ seeding units issued from both pre- and postnatal progenitors. **a** MM electroporated at E15 colocalize with Olig2 marker at P1 (stars in bottom image) but not with S100β (top). **b** The percentage of Olig2+ cells among MM-labeled cells increases from 5% at E18 to 10% at P0 and is then stable until P1, while no MM+/S100β+ cell is detected at these stages. **c** IUE of MM in *Olig2*^*Cre*^ mice at E13 results in labeling of astrocyte-like cells at P7 both at the pial surface and in the cortical parenchyma. **d** Example of a P7 MM-labeled clone occupying three consecutive serial sections comprising S100β+, Aldh1l1+, and Olig2+ cells, revealed by immunostaining. **e** IUE of MM in E13 *Olig2*^*Cre*^ embryos yields sparse recombined cells at E18. Other cells express nuclear EBFP2, indicating efficient targeting of cortical progenitors. **f** Olig2+/Aldh1l1+ cells are found scattered in the E18 cerebral cortex after E15 IUE of integrative ^*Tol2*^*CAG-mEYFP* vector. **g** P1 co-electroporation of episomal *CAG-RFP* and integrative ^*Tol2*^*CAG-mEYFP* vectors labels few astrocytes expressing RFP and markedly more expressing EYFP at P7, among both protoplasmic and pial subtypes (arrows). **h** P0 electroporation of SeCre plasmid in *CAG-Cytbow* mice labels clones of PrA and PiA (arrows) at P7. PrA identity is confirmed by S100β immunostaining (stars). **i** P0 electroporation of integrative ^*Tol2*^*CAG-mEYFP* plasmid labels YFP+ cells scattered in the entire cortical thickness at P3, several of which coexpress Olig2 and Aldh1l1. Graph values indicate mean ± s.e.m. A two-tailed Mann–Whitney statistical test has been performed. * indicates *p* -value < 0.05. *N* = 11 animals. Scale bars: 50 (**a**, **d**), 100 (**c**, **e**–**i**) µm

Having characterized the organization and expansion dynamics of cortical astrocyte clones, we next sought to determine how and when their parent precursors are seeded in the neocortex during development. While embryonic colonization followed by local proliferation was recently proposed as the sole source of astrogenesis^31^, former studies have suggested additional contribution from postnatal progenitors^29,30^. Since astrocytes are labeled by embryonic electroporation of MM, their precursors should also be labeled, providing an opportunity to explore candidate markers of early astrocyte lineage, until now lacking. While S100β, a marker of differentiated astrocytes, did not co-localize with MM pre- or perinatally, immunolabeling revealed that the Olig2 transcription factor was present in 5.1% (±1.0%) at E18 and 9.5% (±1.1%) at P0 of MM + cell population (Fig. 5a, b). Olig2 status as a general macroglial lineage marker expressed by astrocyte precursors in the gray matter of the brain remains debated^41–44^. To clarify its link with the astroglial lineage, we electroporated MM constructs in *Olig2*^*Cre*^ embryos and characterized the identity of labeled cells at postnatal stages, when astrocyte identity can be reliably assessed by morphology and marker expression (Supplementary Fig. 6a, b). At P7 and P21, in addition to oligodendrocytes, Olig2-traced cells also comprised astrocytes of both protoplasmic and pial subtype, identified by morphological features (Fig. 5c and Supplementary Fig. 6a, b) and the presence of the astrocyte-specific markers S100β and Aldh1l1 (Fig. 5d). Expression of Olig2 and these two markers could even be observed in sister cells of a single clone (Fig. 5d). Further confirmation of Olig2 as a marker of astrocyte lineage in the cortical gray matter was obtained by crossing *Olig2*^*Cre*^ mice with a transgenic floxed reporter line, which yielded near-complete PrA labeling in the adult cortical parenchyma (Supplementary Fig. 6c). Thus, a pool of cortical  Olig2+ precursors gives rise to the vast majority of, if not all, cortical astrocytes. To understand how Olig2 lineage-derived cells (and their astrogenic population) invade the cortex, we performed MM electroporation in E13 *Olig2*^*Cre*^ embryos and analyzed at E18 the spatial distribution of color-expressing cells (Fig. 5e). These cells were found scattered along the DV axis of the neocortex with no apparent preferential location or orchestrated segregation. Moreover, Olig2+/Aldh1l1+ cells were also found scattered in the E18 neocortex (Fig. 5f). Together with the clonal results presented previously, these data support the idea that astrocyte precursors disperse prenatally in the cortical parenchyma where they expand as scattered clonal units.

Next, we determined whether cortical astrocytes were solely derived from precursors that colonized the neocortex prenatally or whether they could also originate from postnatally delaminating progenitors. We performed postnatal co-electroporation of episomal (*CAG-RFP*) and integrative vectors (^*Tol2*^*CAG-mEYFP* transposon) into the neuroepithelium, respectively expressing fluorescent reporters transiently in electroporated progenitors (due to episomal markers dilution with successive divisions) or in their entire descent. We observed few astrocytes labeled with the episomal marker, located mainly in the cortical white matter (Fig. 5g), as previously found^31,38^. However, we found numerous PiA and PrA labeled with ^*Tol2*^*CAG-mEYFP*, showing that a significant population of the two subtypes derives from postnatal ventricular progenitors (Fig. 5g), contrary to what has been previously reported^31^. The absence of episomal expression in most labeled cells indicates that their progenitors divide multiple times after the electroporation (P0–P1). Furthermore, electroporation of a Cre-expressing plasmid in transgenic *CAG-Cytbow* mice at P0 (which results in indelible labeling of ventricular progenitors like the integrative markers) labeled numerous color-expressing cortical astrocytes, identified based on morphology as well as S100β and Aldh1l1 expression. These included both pial and protoplasmic subtypes, the latter being observed in all cortical layers (Fig. 5h; Supplementary Fig. 6d, e). Similarly, astrocytes labeled by transposon injection at P0 were found as early as three days post-electroporation scattered in both upper and lower cortical layers, with no apparent preferential location (Fig. 5i). Thus a significant proportion of cortical astrocytes originate from postnatally delaminating cells and like those that emigrate at embryonic stages from the ventricular niche, they disseminate into the cortical parenchyma and give rise to scattered clones that can comprise PrA and more surprisingly PiA, in contradiction with previous work^31^.

These data indicate that both embryonically and postnatally delaminated cortical progenitors contribute to the generation of both PrA and PiA astrocyte subtypes. These progenitors seed the cortical parenchyma in a scattered manner, in contrast with the orderly cohorts of neurons that form sequential layers during the course of corticogenesis.

## Discussion

**Fig. 6:**
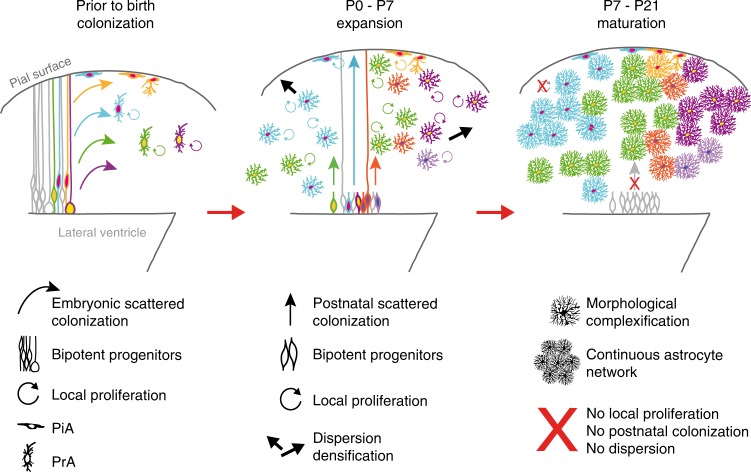
Comprehensive model for astrocyte development in the mouse cortex. Until now the accepted model of mouse cortical astrocyte development consisted of a first phase where embryonic progenitors colonize the neocortical wall followed by a second step relying on local proliferation of these first settlers after birth, the contribution of postnatal progenitors being debated. Here, we propose that mouse cortical astrocytes are issued from a dual contribution of delaminated embryonic apical progenitors and early postnatal progenitors that both generate pial (PiA) and protoplasmic (PrA) astrocytes. Furthermore, our data show that during the first postnatal week (P0–P7) both pre- and postnatal progenitors scatter throughout the neocortical wall while proliferating. This dynamic phase is followed by a maturation phase (P7–P21) where the clones stop both expansion and proliferation while individual astrocytes increase their volume and the complexity of their processes

Altogether our data provide a comprehensive view of astrocyte network formation and maturation during mouse cortical development. This complex process, spread out during prenatal and postnatal development, can be separated in three successive, partially overlapping steps (Fig. 6): (1) colonization of the neocortical wall by a fraction of embryonic apical progenitors which delaminate from the VZ; (2) expansion during the first postnatal week involving the local proliferation of astrocytes derived from scattered delaminated embryonic progenitors, but also continued, non-ordered colonization of the cortical parenchyma by new precursors that remain capable of generating both protoplasmic and pial astrocytes; (3) a maturation phase where individual astrocytes increase both the volume of their territory and the complexity of their processes, while addition of new astrocytes to the already existing network has ceased.

By combining multiplexed clonal labeling with MM and high-resolution 3D large volume multichannel imaging using ChroMS microscopy, we provide for the first time a detailed view of the clonal architecture of the astroglial tridimensional matrix. We show that the apparent continuity of this anatomical network conceals its disparate clonal composition. Contrary to pyramidal neuron clones, which form relatively stereotyped columnar units typically spanning the entire thickness of the cortex^24^, the astrocyte matrix is built of a patchwork of clones highly diverse in terms of cell numbers, subtype composition and shape (Figs. 2 and 4 and Supplementary Fig. 2). This clonal variability, characterized here in mammals, parallels that recently reported for the Drosophila neural cord where individual neuroblasts generate fixed sets of neurons but produce variable numbers of neuropil glia that adopt non-stereotyped morphologies^33^. The average cortical astrocyte clone is comprised of eight cells distributed in two to three separate clusters that are not systematically radially oriented. This suggests the following developmental model, supported by our time-course and birth dating data (Fig. 3): during early stages of clonal expansion, sister astrocytes can disperse in all directions (and not necessarily in strict radially restricted manner) and intermix with neighboring clones, either through non-cohesive divisions or intercalation of newly delaminated precursors. As development proceeds, clonal dispersion progressively diminishes, resulting in the formation of cohesive clusters of sister cells within clones (Fig. 3i). Ultimately, as space becomes restricted, newly divided cells cannot separate entirely from each other and form stable doublets with juxtaposed cell bodies (Fig. 3j). As a result of this developmental process, the astroglial matrix presents a high degree of clonal intermixing at any location in the cortex, a feature that we hypothesize could confer robustness against somatic mutations affecting a fraction of clones.

Interestingly, we were not able to link any property of astrocyte clones with their final location in the cortical parenchyma except for their size, clones present in upper cortical layers being significantly larger compared to those in lower layers (Fig. 4f). This may be due to several reasons: first, their parent progenitor may have colonized the upper neocortex early on and have had more time to divide. However, upper layers continue to be supplied by new astrocytes postnatally (Fig. 5g, h), arguing against this idea. Sparser precursor seeding in upper layers (Fig. 5i) may also necessitate larger clonal expansion to establish continuous tiling in these areas. The augmented clonal size could also be related to the presence of mitotic cues in the upper part of the cortex that could for instance be produced by the surface vasculature. In the rest of the cortex, which are the factors that may determine and regulate the size of astrocyte clones? A recent study, based on MADM genetic fate mapping has shown that astrocyte intermediate progenitor proliferation increases in a cell autonomous manner in absence of Lgl1^45^. While intrinsic determinants such as Lgl1 may limit astrocyte proliferation, the variability of clonal size and patterns that we observed indicates that additional factors are likely at play. Competition for access to neurons and blood vessels may condition their ability to proliferate. Astrocytes are also likely candidates to regulate their own proliferation through competition for space and homeostatic interactions with their neighbors^33,46^, which may suffice to ensure complete tiling of the neuropil. Astrocyte doublets may result from such interplay. Molecular cues produced by astrocytes, neurons and endothelial cells could also participate in this process. The nature of these instructive signals remains to be established, as well as their source of production. A candidate may be vascular derived HBEGF, found to modulate astrocyte survival in vitro^47^. Many other diffusible or membrane-bound candidates could be considered, such as proliferative growth factors that may be secreted by the basal lamina or endothelial cells, or trophic factors and transmembrane proteins produced by neighboring neurons.

The diversity of cortical astrocyte clones that we observed in terms of size and spatial localization argues against the existence of heterogeneous progenitors with distinct and deterministic behaviors. A second observation is also not in favor of such deterministic progenitors: a significant proportion of heterogeneous clones (19%) comprise both PrA and PiA, and most of PiA (>80%) belong to heterogeneous clones, arguing in favor of a common progenitor for these two subtypes. This finding is corroborated by the striking observation of cells displaying an intermediate morphology between PrA and PiA (Fig. 4g). This suggests that these two categories of astrocytes do not represent defined subtypes specified by an intrinsic program, but correspond instead to plastic morphotypes resulting from the adaptation of a single type to its local environment. Indeed each morphotype retains the ability to form close interactions with astrocytes of the other category, ensuring continuous tiling of cortical tissue. This conclusion is at odds with previous clonal studies based on the StarTrack clonal labeling strategy^22^, which reported only a minority (20%) of PiA-containing clones that also included PrA. This led the authors to conclude that these two astrocyte subtypes derive from largely separate lineage branches. Our discordant conclusions are likely explained by the use of a GFAP promoter to drive StarTrack clonal markers^22,48^, known to be unequally regulated among astrocytes, with high PiA and low PrA expression^19,20,36^. MM label both astrocyte populations more widely, thus identifying a larger proportion of bipotent precursors.

Beyond astrocyte lineage, multicolor labeling grants access to fine cellular morphology in dense labeling conditions, which allowed us to assess the morphological changes that astrocytes undertake during corticogenesis. Recently, Stogsdill and collaborators^38^ have shown an increase in the territory and volume infiltrated by V1 astrocytes between P7 and P21. Detailed reconstructions enabled by our semiautomated segmentation pipeline provide access to astrocyte arborization at these stages, showing how its complexity increases over time in situ. Our data also indicate that cortical astrocyte territorial expansion can be at least partially accounted for by the filling of gaps between astrocyte main branches (Fig. 2l, p). In addition, local adaptation of astrocytes to their substrate likely plays an important role in shaping their territory. Indeed in a recent study, Lanjakornsiripan et al.^15^ have found layer differences in astrocyte territory that are altered in *reeler* mutant mice presenting inverted cortical layers. Yet, to which extent variations in astrocyte territory may be explained by modifications in the underlying neuropil remains to be examined.

The availability of the Olig2 marker also enabled us to follow the morphology of early invading glial cells. During embryogenesis, as well as at P4, these cells exhibited mainly a bipolar morphology, but contrary to neurons of the same developmental stage, they presented ramified extremities that were not strictly radially oriented (Fig. 5a, e). Thus, the morphology of young cortical astrocytes becomes gradually more complex over time, apparently independent of the timing of their arrival in the cortex or their proliferation status, but rather correlated to their environment. Indeed, at any given stage, all astrocytes appear to present a same degree of morphological complexity that progressively increases from embryonic to mature stages, independent of their mitotic status.

Tracking the earliest steps of astrocyte development has been difficult due to a lack of marker. Our findings reveal that the transcription factor Olig2, classically associated with oligodendrocyte lineage, also allows the tracing of the entire cortical gray matter astroglial lineage, extending previous findings^41–44^. Although not specific to astrocytes, this marker enabled us to follow the population of young glial cells that includes astrocyte precursors as they colonize the neocortex. With this tool, we did not observe an organized pattern of migration like that of pyramidal neurons which radially migrate in cohorts related to their birth date^1,2^. Non-ordered colonization of the cortex by astrocytes was confirmed by labeling postnatal progenitors, which showed the same nonrestricted potentialities as those targeted during embryogenesis to invade all cortical layers and, contrary to previous reports^28^, to generate distinct astrocyte subtypes. We thus reconcile previous observations about the source of cortical astrocytes, delaminated embryonic^26,31^ versus postnatal progenitors^28–30^, by showing that both contribute to astroglial development. Active division of the latter ones explains why postnatal electroporation of episomal reporter vector fails to label more than a few astrocytes^31,38^. These postnatal progenitors are accessible to postnatal ventricular electroporation and could thus comprise either radial glia cells that remain present during the perinatal period, but also subependymal zone progenitors. Both delaminated embryonic and postnatal-derived astrocyte precursors colonize the neocortex seemingly at random, forming scattered seeding units at the basis of the clonal architecture described above.

In conclusion, our work argues in favor of the existence of nonspecified astrocyte progenitors generating plastic, intermixed clones and whose daughter cells may adopt different morphotypes through interactions with their environment.

## Methods

### Transgenes

MM (^*PB*^*CAG-Cytbow* and ^*Tol2*^*CAG-Nucbow* transposons) and vectors expressing self-excisable Cre recombinase, PB and T2 transposases from a *CAG* promoter^49^ are described in ref. ^34^. The ^*Tol2*^*CAG-mEYFP* integrative vector was produced by cloning *mEYFP*^50^ under a *CAG* promoter in a custom-designed plasmid bearing *5*′ and *3*′ *Tol2* transposition sequences^51^. A *CAG-*driven vector expressing mRFP1^52^ was also used in some experiments. All plasmid concentrations utilized are indicated in Supplementary Table 1. Detailed maps and sequences of the vectors are available upon request.

### Animals

Mice were housed in a 12 h light/12 h dark cycle with free access to food, and animal procedures were carried out in accordance with institutional guidelines. Animal protocols were approved by the Charles Darwin animal experimentation ethical board (CEEACD/N°5). Transgenic *CAG-Cytbow* mice were generated by pronuclear injection of the corresponding transgene linearized with HindIII and MluI. *Olig2*^*Cre*^ (*Olig2tm2(TVA,cre)Rth*) mice were kindly provided by A. Chédotal.

### In vivo experiments

In utero electroporation (IUE): timed-pregnant females were anesthetized with Ketamine/Xylazine, injected with Buprenorphine (Buprecare) to allow pain relief, shaved and placed on a heating pad. A midline laparotomy was performed and uterine horns were exposed under oblique illumination to allow embryo visualization. One microlitre of DNA combined with sterile Fast Green dye (Sigma) was injected with a FemtoJet microinjector (Eppendorf) into the lateral ventricle of each embryo with a glass capillary (FHC, 10-10-L). A CUY21EDIT electroporator (NepaGene) was used to induce four (resp. three) 50 ms pulses of 35 V in Swiss (resp. C57BL/6) embryos with 1.5–3 mm diameter Tweezertrodes (Sonidel Limited) positioned to target the dorsal wall of the lateral ventricle. Following surgery, the incision site was sutured (4-0, Ethicon) and mice were allowed to wake up in a warming chamber. *Postnatal electroporation*: neonatal animals were anesthetized on ice for 1 min and 2 µl of DNA combined with sterile Fast Green dye (Sigma) were injected with a glass capillary (FHC, 10-10-L) into the lateral ventricle by mouth pipetting. A CUY21EDIT electroporator (NepaGene) was used to induce five 50 ms pulses of 80 V with 5 mm diameter Tweezertrodes (Sonidel Limited). Depending on the experiment, recombination was induced either by SeCre DNA electroporation or with Cre-expressing mouse lines.

Mice were euthanized by cervical dislocation at the required developmental stage to harvest embryonic brains. Pups and adults were anesthetized with Dolethal (Vetoquinol) and perfused intracardially with 4% paraformaldehyde (Antigenfix, Diapath) for postnatal analysis. Dissected brains were postfixed for 4 h (embryos) or O/N (pups and adults) at 4 °C under agitation in Antigenfix before several washes in phosphate-buffered saline 1X (PBS). Electroporation and analysis stages, DNA concentrations, mouse lines and associated genetic background used in each figure are listed in Supplementary Table 1.

### Histology and immunostaining

Totally, 80 (embryos and pups) or 60 (adults) µm mouse brains sections were cut with a vibrating-blade microtome (VT1000S, Leica) following embedding in 3% agarose dissolved in PBS 1X. Immunohistology or EdU revelation (Click-iT^™^ Plus EdU Cell Proliferation Kit for Imaging, Alexa Fluor^™^ 647 dye, Sigma) were performed on free floating sections after 1 h incubation in a 0.2% gelatin, 0.5% Triton X-100 (Sigma) PBS 1X blocking solution. Sections were then incubated O/N (embryos) to 72 h (adults) at room temperature with 1:400 rabbit (Merck Millipore AB9610) or 1:200 goat (R&D systems Bio-Techne AF2418) anti-Olig2, 1:500 rabbit anti-Aldh1l1 (Abcam ab87117), 1:500 rabbit anti-S100β (Abcam ab52642), and 1:500 rabbit anti-Ki67 (Abcam ab15580) primary antibodies diluted in blocking solution, then rinsed 3 × 5 min with PBS 1X and incubated 3 h at room temperature with 1:500 goat anti-rabbit Alexa Fluor 647 (Life A21245), 1:500 donkey anti-goat Alexa Fluor 594 (Life A11058) or 1:500 donkey anti-rabbit Alexa Fluor 647 (Jackson 711-605-152) species-specific secondary antibodies. All samples were rinsed 3 × 5 min with PBS 1X and mounted in Vectashield mounting medium (Vector Labs).

### Confocal imaging

Confocal image stacks were acquired with a 20 × 0.8 NA objective on an Olympus FV1000 microscope, using 405, 440, 515, 559, and 633 nm laser lines to separately excite EBFP2, mCerulean/mTurquoise2, EYFP, tdTomato/mCherry/mRFP1, and Alexa Fluor 647, respectively, with a XYZ sampling of 0.62 × 0.62 × 1–1.42 µm. Isolated astrocytes were imaged using a Leica SP8 confocal system with an oil immersion objective 63 × 1.4 NA using 514 and 554 nm laser lines for YFP and tdTomato respectively and *XY* resolution and Z-step size were automatically optimized based on the Nyquist criterion.

### ChroMS microscopy

ChroMS imaging was performed on a lab-built laser scanning two-photon microscope equipped with a vibrating-blade microtome^23^ using a water-immersion objective (25× 1.05 NA, XLPLN25XWMP2, Olympus) and the wavelength mixing method described in ref. ^53^. Imaging depth was set from 117 to 160 µm and slicing from 80 to 120 µm, with a XYZ sampling of 0.4–0.46 × 0.4–0.46 × 1.5 µm.

### Image processing

In all figures, tdTomato/mCherry/mRFP1/Alexa Fluor 594, EYFP, mCerulean/mTurquoise and EBFP2/Alexa Fluor 647 are respectively rendered in red (or magenta in Fig. 5f, g, i), green, blue and gray. Stitching and image contrast adjustments were performed using Fiji^54,55^ or Fluoview (Olympus) and Photoshop (Adobe). 3D renderings were generated using Imaris (Bitplane, Switzerland).

### Clonal analysis

The Fiji TrakEM2 plugin^56^ was used to manually point labeled astrocytes and obtain their *XYZ* coordinates from tridimensional ChroMS datasets (Figs. 1f–k, 2a–k, Supplementary Fig. 2) or from maximal intensity projections of serial 80 µm sagittal sections imaged by tiled confocal microscopy (Figs. 1d–e, 3–5, Supplementary Fig. 1a, c, f, 4a–c, 5, and 6, *Z* coordinate corresponding to section number). Clones were defined as groups of cells expressing the same rare combination of cytoplasmic/nuclear markers sharing the same color display in these two compartments and located less than 600 µm apart, a threshold determined from observations of very rare color combinations only found in a unique animal among those analyzed (Supplementary Fig. 1c–h). Relative positions within the cerebral cortex thickness on the *Y*-axis were obtained automatically from *XYZ* cell coordinates and manually drawn masks of the cortical upper and lower limits using Matlab. For tridimentional analysis (Figs. 1f–k, 2a–k, Supplementary Fig. 2), clonal dispersion was obtained using the *delaunayTriangulation* Matlab function from manually pointed astrocyte *XYZ* coordinates and by measuring automatically all segments formed by the triangulation. The *convexHull* function was used to calculate the volume of the clones. The main axis of each clone was computed by fitting the 3D coordinates of clonally related cells with a line that follows the function *L*(*t*) = *M* + t*V, where *M* is the average of all cell coordinates and *V*, the vector, obtained through singular value decomposition of the coordinate variance-covariance matrix. The angle of this line fit to the radial orientation and its radial projection were evaluated and compared to a randomized dataset. The latter was comprised of the original linear fit axes that were randomly rotated in 3D, resulting in an equal spacing mimicking the properties of a sphere. Connectivity within clones was analyzed by automatically sorting clusters or isolated cells using their XYZ coordinates and astrocyte mean diameter + s.d. at each developmental stage: sister cells separated by distances greater than the mean diameter + s.d. were considered disconnected (Fig. 2g, h). Analysis of astrocyte proliferation (Fig. 3g–l) was carried out in a similar way to that of clonal patterns, and by measuring the distance between EdU + astrocyte pairs in 3D using *XYZ* cell coordinates obtained from image stack.

### Astrocyte domain segmentation

3D segmentation of individual astrocyte domains was performed using Imaris (Bitplane, Switzerland) on 3D crops from the ChroMS datasets showing isolated or color-segregated astrocytes. Semiautomatic iso-intensity contours were drawn in all slices of the corresponding z-stack. Contours were manually drawn when the iso-intensity contour tool did not provide accurate segmentation. The obtained surfaces allowed the software to automatically calculate astrocyte domain volumes. Segmented astrocytes were then distributed into six equivalent bins according to the relative position of their cell body within the cerebral cortex thickness. Distances from cells to pial surface and VZ were manually measured to calculate the cells relative positions.

### Reconstruction of astrocyte arbors

High NA diffraction-limited confocal microscopy images of isolated astrocytes were analyzed using Fiji, Vaa3D^57^, Matlab (MathWorks, USA), and Autoquant X3.1 (Media Cybernetics, USA). Astrocyte arbors were reconstructed using the Vaa3D-Neuron2 Auto Tracing Based on APP2 plugin and their structure was sorted using the sort neuron SWC plugin. SWC were exported to Matlab using the TREES toolbox and the model was resized to the real dimensions from the initial acquisition. Finally, information from the model was extracted using L-measure^58^.

### Statistics

Graphs were obtained with GraphPad Prism (USA) or Matlab (3D graphs). Statistics were done with GraphPad Prism: 2 groups and ≥3 groups comparisons were carried out using the nonparametric Mann–Whitney test, or the nonparametric Kruskal–Wallis test associated with Dunn's multiple comparisons test, respectively. Statistics results, tests, *p* values and *n* associated to all figures are summarized in Supplementary Table 2.

### Reporting summary

Further information on research design is available in the Nature Research Reporting Summary linked to this article.

## Supplementary information

Supplementary Information

Reporting Summary

Description of Additional Supplementary Files

Supplementary Dataset 1

## Data Availability

The data that support the findings of this study are included in the paper. The 3D layout of each astrocyte clone is available as Supplementary Resource. All astrocyte reconstructions are shared through NeuroMorpho.org repository (neuromorpho.org/dableFiles/hernandez/Supplementary/Brainbow_Files.zip). Raw unprocessed data is available from the authors upon request.

## Code availability

All custom codes are available upon request.
