# Supplementary material for: Cortical astrocytes develop in a plastic manner at both clonal and cellular levels
Source: Nat Commun. 2019 Oct 25;10:4884. doi: 10.1038/s41467-019-12791-5 (PMC6814723; doi:10.1038/s41467-019-12791-5)
Supplement: Supplementary file 1 — Supplementary Information [file 41467_2019_12791_MOESM1_ESM.pdf]

Supplementary information for:

Cortical astrocytes develop in a plastic manner at both clonal and cellular levels

Clavreul et al.

Supplementary Figure 1.

In utero electroporation of MAGIC Markers in the mouse cortex permits to identify and resolve astrocyte clones

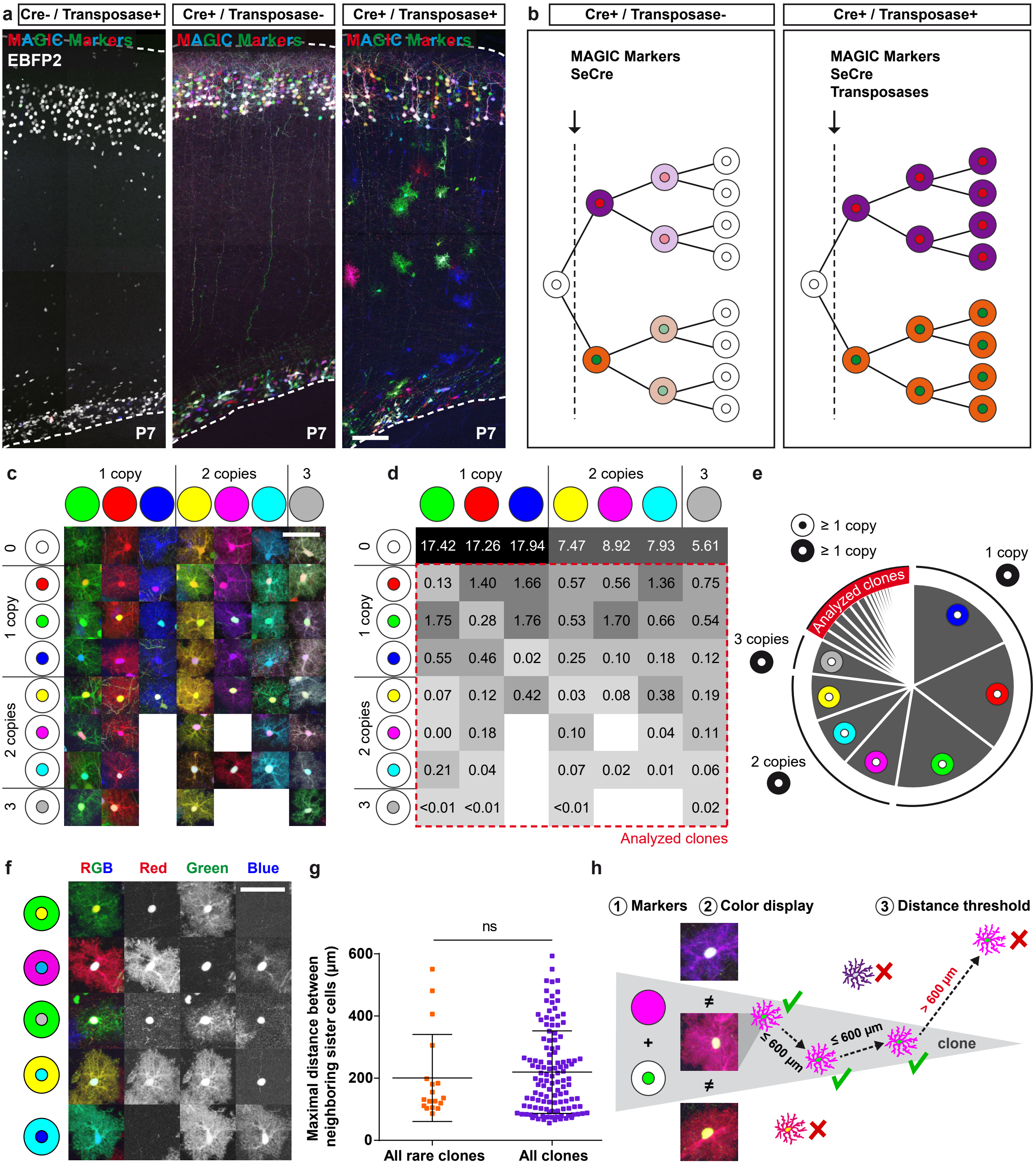

(a) In absence of Cre recombinase, IUE of MAGIC Markers in E15 mouse embryos gives no recombined astrocytes at P7 (EBFP2 is expressed by default and used as electroporation control). In absence of piggyBac and Tol2 transposases, pyramidal neurons located in layers 2-3 are labeled but no cortical astrocytes express FPs. In presence of Cre and transposases, cortical astrocytes are efficiently labeled, among other cell types. (b) In absence of transposases, episomal vectors are diluted prior to astrocyte production (left) whereas transgene integration in cortical progenitors results in stable marker expression in their astrocyte progeny (right). (c) Inventory of color combinations observed in 57,535 cells from 12 animals enables to calculate their frequency (d). (e) Based on the analysis of color combinations frequency, clonal analysis was restricted to nucleus-cytoplasm combinations, each representing less than 2% of labeled astrocytes. (f) Very rare RGB combinations (found only within a single animal) were used to establish a spatial distance threshold beyond which two cells were not considered clonally-related. (g) The maximal distance between neighboring sister cells in these unique clones (600 µm) was used as spatial threshold. (h) Based on the above observations, astrocyte clones were defined by their RGB distribution in both cytoplasm and nucleus (presence or absence of FPs in each compartment), final color display, and a maximal distance of 600 µm from one sister cell to another. Graph values indicate mean ± s.d. A two-tailed Mann-Whitney statistical test has been performed. N=4 animals. Scale bars: 100 (a), 50 (c,f) µm.

Supplementary Figure 2.

3D analysis of protoplasmic astrocyte clones reveals highly variable patterns that appear stable from P7 to P21

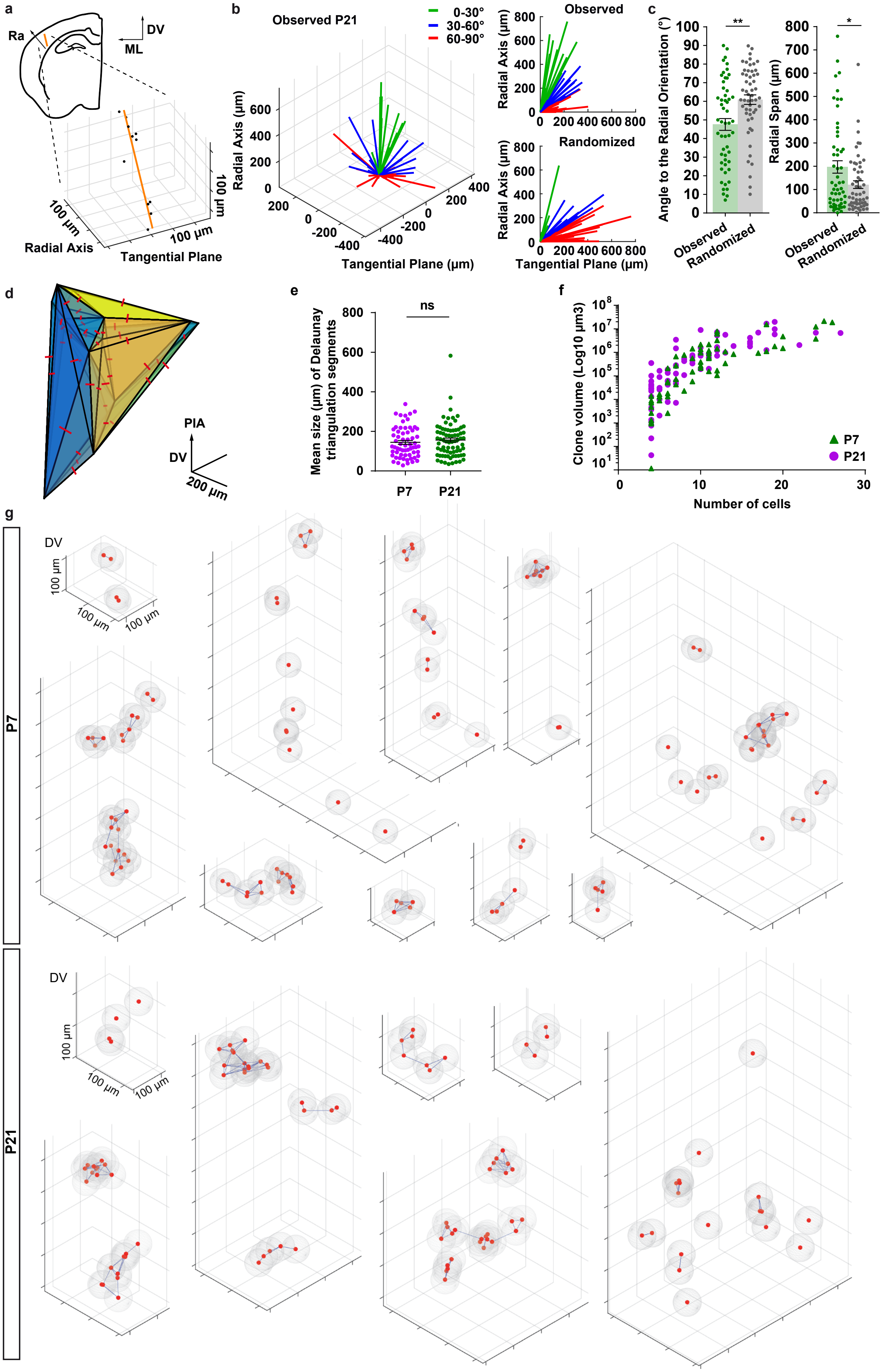

Supplementary Figure 2.
3D analysis of protoplasmic astrocyte clones reveals highly variable patterns that appear stable from P7 to P21

(a) Schematic of a coronal forebrain section and example of an astrocyte clone whose principal axis, obtained by a 3D linear fit of all its cell positions, is displayed in orange. (b) 3D plot comparing the principal axes of P21 clones, superimposed at their deepest point, illustrating the variability of their orientation with respect to the radial direction (left); 2D plots (right) showing the clones' principal axis length and angle to the radial orientation for observed (top) and randomized (bottom) data. (c) The average angle of P21 astrocyte clones axis to the radial orientation is significantly smaller than that obtained from randomized data, while their radial span is significantly larger. (d) The length of segments (red marks) formed by Delaunay triangulation of astrocyte clones is used to estimate their dispersion. (e) The mean length of astrocyte clones Delaunay segments is similar at P7 and P21, revealing stability of sister cell dispersion between the two stages. (f) Convex hull analysis shows similar distribution of clonal volumes (globally related to clone size) at P7 and P21, despite important variability.(g) Connectivity diagrams of P7 and P21 astrocyte clones reveal variable patterns of organization. Each gray sphere represents the domain of one sister cell (sphere size is based on mean astrocyte diameter at each developmental stage) and its nucleus (in red, based on nucleus mean size at each stage). Sister cells at a distance < PrA mean diameter + s.d are considered connected and linked by a blue line. DV: dorsoventral. Graph values indicate mean±s.e.m. Two-tailed (S2c1, S2e) and one-tailed (S2c2) Mann-Whitney statistical tests have been performed. ** and * indicate p-value <0.005 and <0.05, respectively. N=2 (S2c1, S2c2) and 4 (S2e) animals.

Supplementary Figure 3.
Semi-automated 3D segmentation and high resolution reconstruction of isolated PrA reveals their morphological heterogeneity

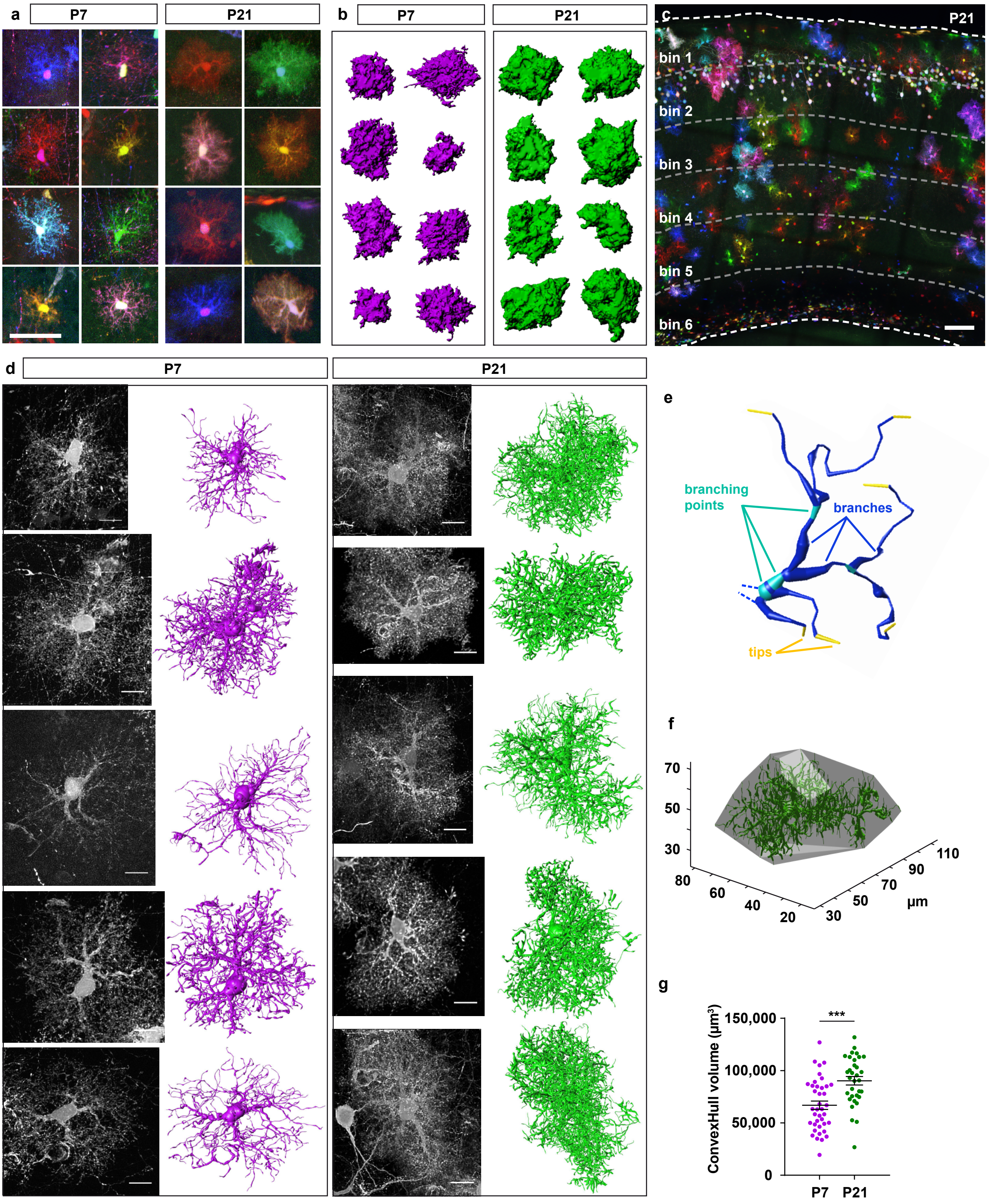

(a, b) Representative examples of semi-automated segmentation of the territory of 193 spatially isolated MM-labeled PrA imaged by ChroMS microscopy (n= 193), enabling extraction of their volume and surface, analyzed in Fig 2j. (c) The cortical parenchyma is divided in six equivalent bins (Bin 1 = pial surface) for PrA volume comparisons, shown in Fig. 2k. (d-e) High resolution imaging and tracing of multiple cortical PrA highlight the morphological heterogeneity and cellular complexity of cortical astrocytes. (f) ConvexHull volume of PrA models increases significantly from P7 to P21 (g). Graph values indicate mean ± s.e.m. A two-tailed Mann-Whitney statistical test has been performed. *** indicates p-value = 0.0001. N=10 animals. Scale bars (µm): 50 (a-b), 100 (c), 10 (d).

Supplementary Figure 4.

PrA clones display variable spatial distribution that becomes denser during the first postnatal week

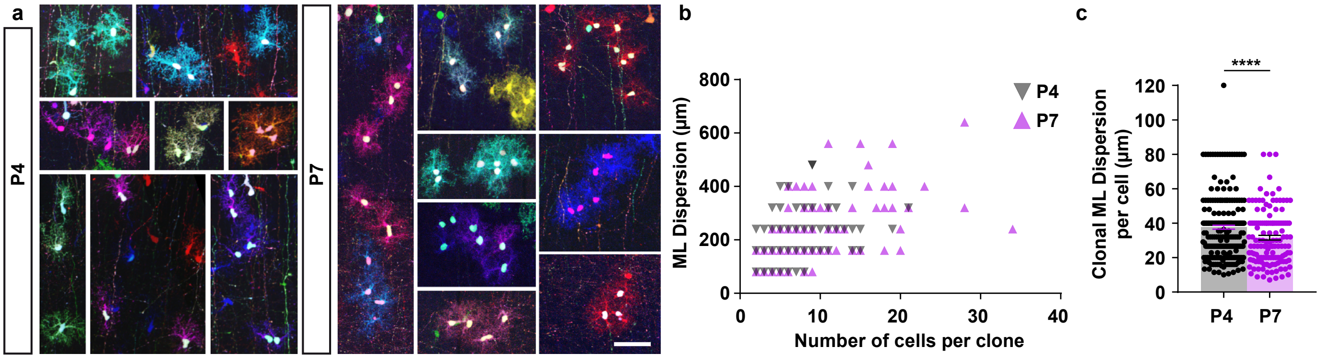

(a) PrA clones labeled by IUE of MAGIC Markers at E15 display various spatial arrangements (clusters, columns, combinations of both) at P4, P7 and P21. (b) ML dispersion of PrA clones ≥2 cells as a function of clone size is broad and variable. (c) ML dispersion per cell in µm (ML clonal dispersion/ clone size) decreases from P4 to P7. ML: mediolateral. Graph values indicate mean ± s.e.m. A two-tailed Mann-Whitney statistical test has been performed. *** indicates p-value = 0.001. N=6 animals. Scale bar (µm): 50 (a).

Supplementary Figure 5.
Cortical progenitors can generate heterogeneous clones comprising both PrA and PiA and proliferation of these two subtypes decreases drastically between P4 and P7

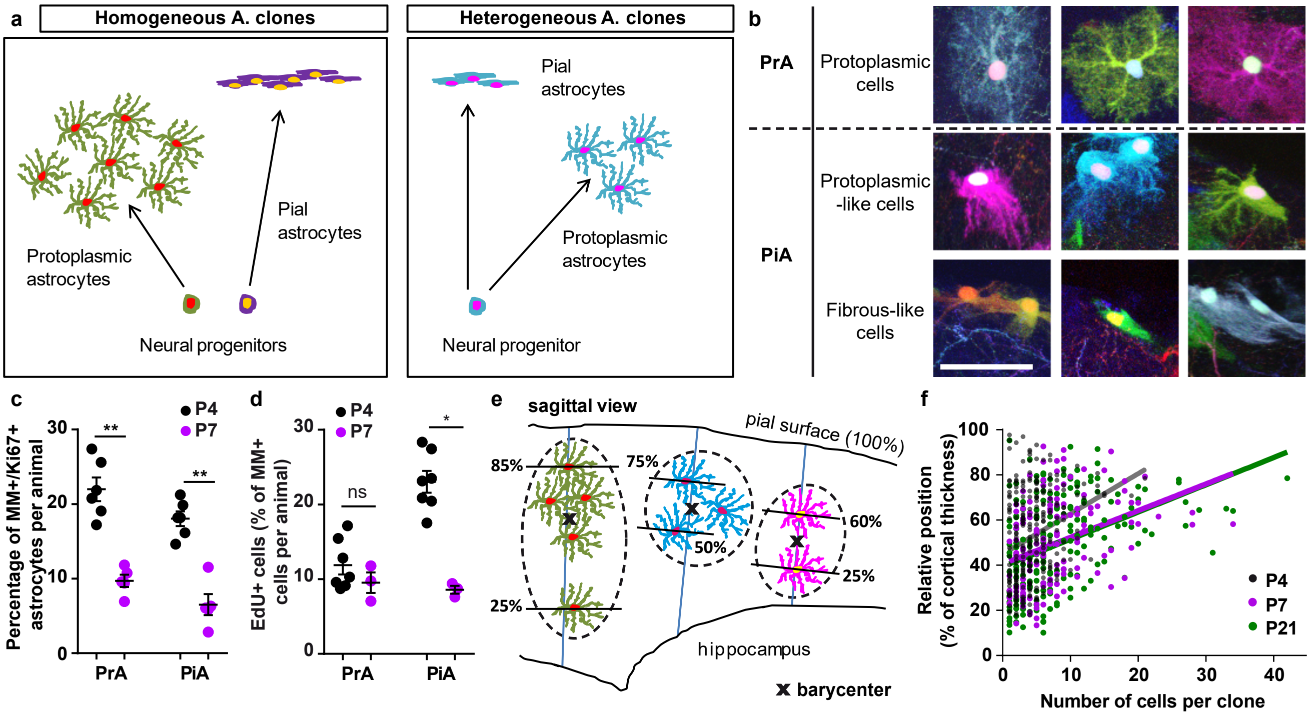

(a) Representative scheme of the three classes of astrocyte clones found in the mouse cerebral cortex: homogeneous PrA, homogeneous PiA and heterogeneous clones containing both PrA and PiA subtypes. (b) PiAs display various morphologies and can be separated in two categories: protoplasmic-like cells and fibrous-like cells. (c) The percentage of Ki67+ cells among astrocytes labeled with MAGIC Markers is halved between P4 and P7 for both PrA and PiA subtypes. (d) 11% of labeled PrA are EdU+ at both P4 and P7 whereas this proportion drops from 23 to 9% for PiA. (e) Approach used in Fig 3d-f, l-m and Supplementary Fig. 5f for relative DV positioning of astrocytes with respect to the pial surface (taken as 100%) and the VZ (relative position = d(VZ-cell)/ d(VZ-pial surface) *100). (f) Distribution and linear regression of the relative positions of astrocyte clone barycenters as a function of clone size, showing that astrocyte clones grow larger as they are located closer to the pial surface. DV: dorsoventral. Scale bar: 50 µm. Graph values indicate means ± s.e.m. Two-tailed Mann-Whitney statistical tests have been performed. ** and * indicate p-value = 0.0043 (S5c) and 0.0167 (S5d), respectively. N=11 (S5c) and 10 (S5d) animals. Scale bars: 50 µm.

Supplementary Figure 6.
Olig2 lineage and postnatal progenitors contribute to astrogliogenesis

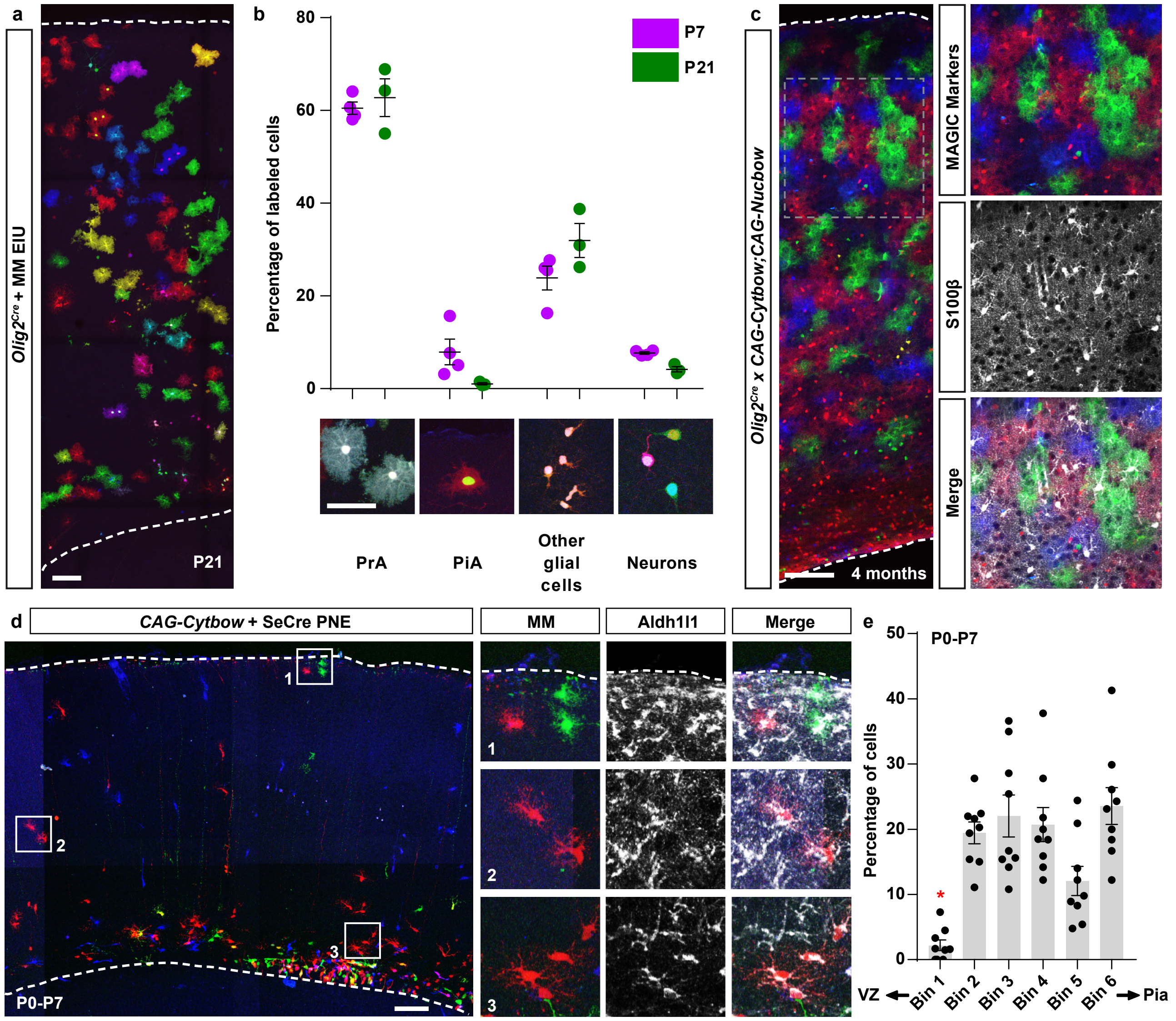

(a) E13 IUE of MM in *Olig2^Cre^* embryos results in dense labeling at P21 where 61, 5, 27 and 6% of labeled cells can be identified as PrA, PiA, or other glial cells and neurons, respectively (b). (c) 4 month-old *Olig2^Cre^;CAG-Cytbow;CAG-Nucbow* animals displaying near-complete coverage of the cerebral cortex with astrocytes, identified by their morphology and S100β expression. (d) Cells labeled at P7 by electroporation of SeCre in *CAG-Cytbow* animals at P0 are Aldh1l1+ astrocytes located in various cortical layers. (e) Protoplasmic astrocytes labeled in this manner are observed at all radial positions in the cortical parenchyma, here divided in six equivalent bins. Graph shows mean ± s.e.m. from 774 astrocytes. Bin 1 shows significantly lower number of cells (red asterisk), but white matter cells in this bin were excluded from analysis. Graph values indicate means ± s.e.m. Two-tailed Mann-Whitney (S6b) and Kruskal-Wallis associated with Dunn's multiple comparisons (S6e) statistical tests have been performed. **** indicates p-value <0.0001. N=7 (S6b) and 9 (S6e) animals. Scale bars (µm): 100 (a,c,d), 50 (b).

Supplementary Table 1 : Electroporation and analysis stages, DNA concentrations, mouse lines and associated genetic background used in each figure

Figure	Analysis	Imaging method	Elec. stage	Mouse line	Genetic Background	[^{PB}Cytbow]	[^{Tol2}Nucbow]	[PB transposase]	[Tol2 transposase]	[SeCre]
**3a-f,j-l/4/S4a-c/S5f**	**P4**	**Serial sections**	E15	WT	Swiss	0.8 µg µl^{-1}	0.8 µg µl^{-1}	0.4 µg µl^{-1}	0.4 µg µl^{-1}	0.16 µg µl^{-1}
**1d,e/2l-p/3a-f,j-l/4/ S3d-g/S4a-c,/S5f**	**P7**	**Serial sections**	E15	WT	Swiss	0.8 µg µl^{-1}	0.8 µg µl^{-1}	0.4 µg µl^{-1}	0.4 µg µl^{-1}	0.16 µg µl^{-1}
**1d,e/2l-p/3j-l/4/S3d-g/S5b,f**	**P21**	**Serial sections**	E15	WT	Swiss	0.8 µg µl^{-1}	0.8 µg µl^{-1}	0.4 µg µl^{-1}	0.4 µg µl^{-1}	0.16 µg µl^{-1}
**1f-k/2a-k/S2/S3a-c**	**P7**	**ChroMS Microscopy**	E15	WT	Swiss	0.8 µg µl^{-1}	0.8 µg µl^{-1}	0.4 µg µl^{-1}	0.4 µg µl^{-1}	0.16 µg µl^{-1}
**1f-k/2a-k/S2/S3a-c**	**P21**	**ChroMS Microscopy**	E15	WT	Swiss	0.8 µg µl^{-1}	0.8 µg µl^{-1}	0.4 µg µl^{-1}	0.4 µg µl^{-1}	0.16 µg µl^{-1}
**3g-i,m,n/S5c,d**	**P4**	**Serial sections**	E15	WT	Swiss	0.8 µg µl^{-1}	1.0 µg µl^{-1}	0.4 µg µl^{-1}	0.5 µg µl^{-1}	0.18 µg µl^{-1}
**3g-i,m,n/S5c,d**	**P7**	**Serial sections**	E15	WT	Swiss	0.8 µg µl^{-1}	1.0 µg µl^{-1}	0.4 µg µl^{-1}	0.5 µg µl^{-1}	0.18 µg µl^{-1}
**5a,b**	**E18**	**Serial sections**	E15	WT	Swiss	0.8 µg µl^{-1}	0.8 µg µl^{-1}	0.4 µg µl^{-1}	0.4 µg µl^{-1}	0.16 µg µl^{-1}
**5a,b**	**P0**	**Serial sections**	E15	WT	Swiss	0.8 µg µl^{-1}	0.8 µg µl^{-1}	0.4 µg µl^{-1}	0.4 µg µl^{-1}	0.16 µg µl^{-1}
**5a,b**	**P1**	**Serial sections**	E15	WT	Swiss	0.8 µg µl^{-1}	0.8 µg µl^{-1}	0.4 µg µl^{-1}	0.4 µg µl^{-1}	0.16 µg µl^{-1}
**5h/S6d-e**	**P7**	**Serial sections**	P0	*CAG-Cytbow*	*C57BL/6*	-	-	-	-	0.80 µg µl^{-1}
**S1a (CTL Cre-)**	**P7**	**Serial sections**	E15	WT	Swiss	0.8 µg µl^{-1}	0.8 µg µl^{-1}	0.4 µg µl^{-1}	0.4 µg µl^{-1}	-
**S1a (CTL Tp-)**	**P7**	**Serial sections**	E15	WT	Swiss	0.8 µg µl^{-1}	0.8 µg µl^{-1}	-	-	0.16 µg µl^{-1}
**5c,d**	**P7**	**Serial sections**	E13	*Olig2tm2(TVA,cre)Rth*	*C57BL/6*	0.8 µg µl^{-1}	0.8 µg µl^{-1}	0.4 µg µl^{-1}	0.4 µg µl^{-1}	-
**5e**	**E18**	**Serial sections**	E13	*Olig2tm2(TVA,cre)Rth*	*C57BL/6*	0.8 µg µl^{-1}	0.8 µg µl^{-1}	0.4 µg µl^{-1}	0.4 µg µl^{-1}	-
**S6a,b**	**P21**	**Serial sections**	E13	*Olig2tm2(TVA,cre)Rth*	*C57BL/6*	0.8 µg µl^{-1}	0.8 µg µl^{-1}	0.4 µg µl^{-1}	0.4 µg µl^{-1}	-

Figure	Analysis	Imaging method	Elec. stage	Mouse line	Genetic Background	[^{Tol2}CAG-mEYFP]	[Tol2 transposase]	[CAG-RFP]
**5f**	**P0**	**Serial sections**	P0	WT	Swiss	3.2 µg µl^{-1}	1.6 µg µl^{-1}	-
**5g**	**P7**	**Serial sections**	P1	WT	Swiss	1.0 µg µl^{-1}	0.5 µg µl^{-1}	1.0 µg µl^{-1}
**5i**	**P3**	**Serial sections**	P0	WT	Swiss	3.2 µg µl^{-1}	1.6 µg µl^{-1}	-

Supplementary Table 2 : Summary of statistical analyses performed for each figure

Fig.	Test	Result (significant = P<0.05)	n unit	n	Mean	SD	SEM	Error bar	Dunn's multiple comparisons test	Result	Adjusted P Value
**2a**	Clonal dispersion (µm): 4 animals; Kruskal-Wallis test	*******	P7 clones (AP dispersion)	**50**	95.4	65.01	9.194	SEM	P7 AP vs. P21 AP	**ns**	0.9576
		**p=0.0003**	P21 clones (AP dispersion)	**63**	126.7	114.8	14.46	SEM	P7 DV vs. P21 DV	**ns**	>0.9999
			P7 clones (DV dispersion)	**50**	230.7	205.2	29.03	SEM	P7 ML vs. P21 ML	**ns**	>0.9999
			P21 clones (DV dispersion)	**63**	264.8	279.7	35.24	SEM	P7+P21 AP vs. P7+P21 DV	*******	0.0001
			P7 clones (ML dispersion)	**50**	120	124.1	17.55	SEM	P7+P21 AP vs. P7+P21 ML	**ns**	>0.9999
			P21 clones (ML dispersion)	**63**	120.7	98.48	12.41	SEM	P7+P21 DV vs. P7+P21 ML	*******	0.0002
**2e**	Volume of >3 cells clones (μm^3): 4 animals; Two-tailed Mann-Whitney test	**ns**	P7 clones	**62**	1959,702	4363,515	554,167	SEM			
		**p=0.9605**	P21 clones	**75**	1826,509	3587,585	414,259	SEM			
**2g**	Number of disconnected elements per PrA clone: 4 animals; Two-tailed Mann-Whitney test	**ns**	P7 clones	**53**	2.83	1.919	0.2636	SEM			
		**p=0.9043**	P21 clones	**65**	2.862	2.045	0.2537	SEM			
**2h**	% of cells in clusters within PrA clones: 4 animals; Two-tailed Mann-Whitney test	**ns**	P7 clones	**53**	78.67	32.45	4.457	SEM			
		**p=0.2747**	P21 clones	**65**	73.82	31.94	3.962	SEM			
**2j**	PrA territory volume (µm3): 4 animals; Two-tailed Mann-Whitney test	********	P7 PrA	**88**	43,068	16,994	1,812	SEM			
		**p<0.0001**	P21 PrA	**105**	71,379	25,483	2,487	SEM			
**2k**	PrA territory volume (µm3): 4 animals; Two-tailed Mann-Whitney tests	********	P7 PrA (bin 1)	**11**	22,197	8,013	2,416	SEM			
		**p<0.0001**	P21 PrA (bin 1)	**15**	40,402	11,490	2,967	SEM			
		********	P7 PrA (bin 2)	**14**	41,782	17,945	4,796	SEM			
		**p<0.0001**	P21 PrA (bin 2)	**14**	84,833	17,357	4,639	SEM			
		********	P7 PrA (bin 3)	**12**	53,228	18,524	5,348	SEM			
		**p<0.0001**	P21 PrA (bin 3)	**16**	86,394	14,118	3,530	SEM			
		*****	P7 PrA (bin 4)	**18**	52,171	15,033	3,543	SEM			
		**p=0.0146**	P21 PrA (bin 4)	**20**	68,665	24,243	5,421	SEM			
		*****	P7 PrA (bin 5)	**22**	47,881	9,620	2,051	SEM			
		**p=0.0197**	P21 PrA (bin 5)	**30**	81,415	22,621	4,130	SEM			
		********	P7 PrA (bin 6)	**11**	29,966	8,917	2,689	SEM			
		**p<0.0001**	P21 PrA (bin 6)	**10**	50,308	22,685	7,174	SEM			

**2m**	Number of PrA branches: 9 animals; Two-tailed Mann-Whitney test	***	P7 PrA	**39**	813.5	687	110	SEM			
		**p=0.0004**	P21 PrA	**33**	1,360	870.9	151.6	SEM			
**2n**	Total length of PrA branches (µm): 9 animals; Two-tailed Mann-Whitney test	****	P7 PrA	**39**	3,648	1,661	266.1	SEM			
		**p<0.0001**	P21 PrA	**33**	6,498	3,198	556.6	SEM			
**2o**	Volume of PrA models (µm3): 9 animals; Two-tailed Mann-Whitney test	**	P7 PrA	**39**	2,080	930.3	149	SEM			
		**p=0.0014**	P21 PrA	**33**	2,900	1,143	198.9	SEM			
**3b**	Number of cells per PrA clone: 9 animals; Kruskal-Wallis test	****	P4 clones	**290**	4.459	3.232	0.1898	SEM	P4 vs. P7 PrA clones	****	<0.0001
		**p<0.0001**	P7 clones	**137**	7.949	6.02	0.5143	SEM	P7 vs. P21 PrA clones	**ns**	>0.9999
			P21 clones	**212**	8.142	6.906	0.4743	SEM			
**3f**	Relative DV dispersion (% of cortical thickness/number of cells) per PrA clone >1 cell: 6 animals; One-tailed Mann-Whitney test	*	P4 PrA clones	**250**	3.947	3.508	0.2219	SEM			
		**p=0.0381**	P7 PrA clones	**132**	3.032	2.302	0.2004	SEM			
**3i**	Distance between EdU+ cells within MM+ PrA pairs (µm): 10 animals; Kruskal-Wallis test	****	EdU+ 48hpi /MM+ P4 PrA pairs	**70**	37.19	24.93	2.979	SEM	48hpi P4 vs. 24hpi P4	****	<0.0001
		**p<0.0001**	EdU+ 24hpi /MM+ P4 PrA pairs	**176**	21.6	11.82	0.8911	SEM	48hpi P7 vs. 24hpi P7	*	0.0446
			EdU+ 48hpi /MM+ P7 PrA pairs	**38**	24.49	13.83	2.243	SEM	48hpi P4 vs. 48hpi P7	*	0.0357
			EdU+ 24hpi /MM+ P7 PrA pairs	**108**	19.97	14.12	1.358	SEM	24hpi P4 vs. 24hpi P7	**ns**	0.1083
**3k**	% of PrA belonging to doublets among PrA per animal: 9 animals; Kruskal-Wallis test	**ns**	P4 animals	**3**	0.2022	0.04787	0.0276	SEM	P4 vs. P7	**ns**	>0.9999
		**p=0.8286**	P7 animals	**3**	0.2451	0.1528	0.0882	SEM	P7 vs. P21	**ns**	>0.9999
			P21 animals	**3**	0.1688	0.06182	0.0357	SEM			
**4d**	% of clones per animal for each clone category: 9 animals; Kruskal-Wallis tests	**	P4 animals (PrA: % of clones)	**3**	77.46	8.367	4.831	SEM	P4 vs. P7 PrA	**ns**	>0.9999
		**p=0.0024**	P7 animals (PrA: % of clones)	**3**	70.56	2.747	1.586	SEM	P7 vs. P21 PrA	**ns**	>0.9999
			P21 animals (PrA: % of clones)	**3**	80.28	6.935	4.004	SEM	P4 vs. P7 PiA	**ns**	>0.9999
			P4 animals (PiA: % of clones)	**3**	5.176	3.618	2.089	SEM	P7 vs. P21 PiA	**ns**	>0.9999
			P7 animals (PiA: % of clones)	**3**	9.253	4.987	2.879	SEM	P4 vs. P7 Heterogeneous	**ns**	>0.9999
			P21 animals (PiA: % of clones)	**3**	1.357	1.318	0.7608	SEM	P7 vs. P21 Heterogeneous	**ns**	>0.9999
			P4 animals (Heterogeneous: % of clones)	**3**	17.37	4.903	2.831	SEM	P4 vs. P21 PrA	**ns**	>0.9999
			P7 animals (Heterogeneous: % of clones)	**3**	20.19	3.866	2.232	SEM	P4 vs. P21 PiA	**ns**	>0.9999
			P21 animals (Heterogeneous: % of clones)	**3**	18.37	6.119	3.533	SEM	P4 vs. P21 Heterogeneous	**ns**	>0.9999

		****	P4P7P21 animals (PrA: % of clones)	9	76.1	7.081	2.36	-	PrA vs. PiA clones	****	<0.0001
		p<0.0001	P4P7P21 animals (PiA: % of clones)	9	5.262	4.65	1.55	-	PiA vs. Heterogenous clones	ns	0.0568
			P4P7P21 animals (Heterogeneous: % of clones)	9	19.25	3.933	1.311	-	PrA vs. Heterogeneous clones	*	0.0445
4e	Number of cells per clone for each clone category: 9 animals; Kruskal-Wallis test	****	P4 PrA clones	290	4.459	3.232	0.1898	SEM	P4 PrA vs. P4 Heterogeneous clones	****	<0.0001
		p<0.0001	P4 Heterogeneous clones	69	8.841	4.451	0.5359	SEM	P7 PrA vs. P7 Heterogeneous clones	****	<0.0001
			P7 PrA clones	137	7.949	6.02	0.5143	SEM	P21 PrA vs. P21 Heterogeneous clones	****	<0.0001
			P7 Heterogeneous clones	42	13.93	6.07	0.9367	SEM			
			P21 PrA clones	212	8.142	6.906	0.4743	SEM			
			P21 Heterogeneous clones	52	13.25	6.718	0.9316	SEM			
4f	Number of cells per PrA clone in lower or upper cortical layers: 9 animals; Kruskal-Wallis test	****	P4 Lower layers PrA clones	139	3.532	2.301	0.1952	SEM	P4 L vs. P4 U	***	0.0004
		p<0.0001	P4 Upper layers PrA clones	151	5.311	3.704	0.3015	SEM	P4 L vs. P7 L	***	0.0003
			P7 Lower layers PrA clones	70	5.914	4.141	0.495	SEM	P7 L vs. P21 L	ns	>0.9999
			P7 Upper layers PrA clones	67	10.07	6.911	0.8444	SEM	P4 U vs. P7 U	****	<0.0001
			P21 Lower layers PrA clones	109	5.385	4.599	0.4405	SEM	P7 U vs. P21 U	ns	>0.9999
			P21 Upper layers PrA clones	103	11.06	7.718	0.7605	SEM	P21 L vs. P21 U	****	<0.0001
									P7 L vs. P7 U	**	0.001
5b	% of S100β+/MM+ cells: 11 animals; Two-tailed Mann-Whitney test	*	E18 animals	4	5.045	1.941	0.9705	SEM			
		p=0.0286	P0 animals	4	9.493	2.253	1.127	SEM			
			(P1 animals)	3	9.241	0.6387	0.3688	SEM			
S1g	Maximal distance between one cell and her neighboring sister cells per clone (µm): 4 animals; Two-tailed Mann-Whitney test	ns	Rares clones	18	200.6	140	32.99	SD			
		p=0.6421	All clones	122	219.5	132.6	12.01	SD			
S2c1	Angle of PrA clones >2 cells to the radial orientation: 2 animals; Two-tailed Mann-Whitney test	**	P21 PrA clones (observed angle)	54	47.59	23.31	3.171	SEM			
		p=0.0024	P21 PrA clones (randomized angles)	54	60.93	18.72	2.547	SEM			
S2c2	Radial span (µm) of PrA clones >2 cells = projection onto the radial axis: 2 animals; One-tailed Mann-Whitney test	*	P21 PrA clones (observed radial span)	54	197	196.5	26.74	SEM			
		p=0.0459	P21 PrA clones (randomized)	54	121.1	120.5	16.39	SEM			

S2e	Mean size (µm) of Delaunay triangulation segments per clone >3 cells: 4 animals; Two-tailed Mann-Whitney test	ns	P7 clones	62	144.6	77.27	9.814	SEM			
		p=0.5447	P21 clones	75	156.3	88.26	10.19	SEM			
S3g	ConvexHull volume of PrA models (µm3): Two-tailed Mann-Whitney test	***	P7 PrA	39	66,898	24,828	3,976	SEM			
		p=0.0001	P21 PrA	33	90,270	23,176	4,034	SEM			
S4c	Relative ML dispersion (dispersion/number of cells) (µm) per PrA clone >1 cell: 6 animals; Two-tailed Mann-Whitney test	***	P4 PrA clones	250	37.69	17.78	1.125	SEM			
		p=0.001	P7 PrA clones	132	31.56	15.8	1.375	SEM			
S5c	% of MM+/Ki67+ PrA: 11 animals; Two-tailed Mann-Whitney test	**	P4 animals	6	21.98	3.872	1.581	SEM			
		p=0.0043	P7 animals	5	9.702	1.83	0.8185	SEM			
	% of MM+/Ki67+ PiA: 11 animals; Two-tailed Mann-Whitney test	**	P4 animals	6	18.04	2.404	0.9816	SEM			
		p=0.0043	P7 animals	5	6.514	3.143	1.406	SEM			
S5d	% of EdU+/MM+ PrA: 10 animals; Two-tailed Mann-Whitney test	ns	P4 animals (% of EdU+/MM+ PrA)	7	11.88	3.332	1.259	SEM			
		p=0.5167	P7 animals (% of EdU+/MM+ PrA)	3	9.533	2.371	1.369	SEM			
	% of EdU+/MM+ PiA: 10 animals; Two-tailed Mann-Whitney test	*	P4 animals (% of EdU+/MM+ PiA	7	23.04	3.863	1.46	SEM			
		p=0.0167	P7 animals (% of EdU+/MM+ PiA)	3	8.57	0.9089	0.5247	SEM			
S6b	% of MM+ cells for each cell type: 7 animals; Two-tailed Mann-Whitney tests	ns	P7 animals (PrA: % of MM+ cells)	4	60.48	2.653	1.327	SEM			
		p=0.6286	P21 animals (PrA: % of MM+ cells)	3	62.76	7.053	4.072	SEM			
		ns	P7 animals (PiA: % of MM+ cells)	4	7.93	5.528	2.764	SEM			
		p=0.0571	P21 animals (PiA: % of MM+ cells)	3	1.054	0.3872	0.2236	SEM			
		ns	P7 animals (Other glial cells: % of MM+ cells)	4	23.89	5.148	2.574	SEM			
		p=0.1143	P21 animals (Other glial cells: % of MM+ cells)	3	31.99	6.335	3.658	SEM			
		ns	P7 animals (Neurons: % of MM+ cells)	4	7.703	0.5395	0.2697	SEM			
		p=0.0571	P21 animals (Neurons: % of MM+ cells)	3	4.193	0.9862	0.5694	SEM			
S6e	% of MM+ cells in each bin: 9 animals; Kruskal-Wallis test	****	P7 animals (% PrA in Bin 1)	9	2.211	2.453	0.8176	SEM	Bin 1 vs bins 2-4	**	<0.0024
		<0.0001	P7 animals (% PrA in Bin 2)	9	19.44	4.952	1.651	SEM	Bin 1 vs bin 6	***	0.0001
			P7 animals (% PrA in Bin 3)	9	22.03	9.593	3.198	SEM	Bin 1 vs bin 5	ns	0.7709
			P7 animals (% PrA in Bin 4)	9	20.73	7.844	2.615	SEM	Other comparisons	ns	>0.1662
			P7 animals (% PrA in Bin 5)	9	12.08	6.713	2.238	SEM			
			P7 animals (% PrA in Bin 6)	9	23.56	8.496	2.832	SEM			
