# Supplementary material for: Cortical astrocytes develop in a plastic manner at both clonal and cellular levels
Source: Nat Commun. 2019 Oct 25;10:4884. doi: 10.1038/s41467-019-12791-5 (PMC6814723; doi:10.1038/s41467-019-12791-5)
Supplement: Supplementary file 2 — Reporting Summary [file 41467_2019_12791_MOESM2_ESM.pdf]

nature research

Corresponding author(s): Karine LOULIER karine.loulier@inserm.fr
Jean LIVET jean.livet@inserm.fr

Reporting Summary

Nature Research wishes to improve the reproducibility of the work that we publish. This form provides structure for consistency and transparency in reporting. For further information on Nature Research policies, see Authors & Referees and the Editorial Policy Checklist.

Statistical parameters

When statistical analyses are reported, confirm that the following items are present in the relevant location (e.g. figure legend, table legend, main text, or Methods section).

n/a	Confirmed	
☐	☒	The exact sample size (*n*) for each experimental group/condition, given as a discrete number and unit of measurement
☐	☒	An indication of whether measurements were taken from distinct samples or whether the same sample was measured repeatedly
☐	☒	The statistical test(s) used AND whether they are one- or two-sided<br>*Only common tests should be described solely by name; describe more complex techniques in the Methods section.*
☒	☐	A description of all covariates tested
☐	☒	A description of any assumptions or corrections, such as tests of normality and adjustment for multiple comparisons
☐	☒	A full description of the statistics including central tendency (e.g. means) or other basic estimates (e.g. regression coefficient) AND variation (e.g. standard deviation) or associated estimates of uncertainty (e.g. confidence intervals)
☐	☒	For null hypothesis testing, the test statistic (e.g. *F*, *t*, *r*) with confidence intervals, effect sizes, degrees of freedom and *P* value noted<br>*Give P values as exact values whenever suitable.*
☒	☐	For Bayesian analysis, information on the choice of priors and Markov chain Monte Carlo settings
☒	☐	For hierarchical and complex designs, identification of the appropriate level for tests and full reporting of outcomes
☒	☐	Estimates of effect sizes (e.g. Cohen's *d*, Pearson's *r*), indicating how they were calculated
☐	☒	Clearly defined error bars<br>*State explicitly what error bars represent (e.g. SD, SE, CI)*

Our web collection on statistics for biologists may be useful.

Software and code

Policy information about availability of computer code

Data collection	Fluorescent imaging was acquired with Fluoview (Olympus) or Leica software (confocal imaging) or LabVIEW custom software (ChroMS microscopy).
Data analysis	Images were processed and analyzed using Fiji or Imaris 8-9 (Bitplane).<br>Stitching and image display were performed using Fiji or Fluoview (Olympus) and Photoshop (Adobe).<br>3D renderings were generated using Imaris 8-9 (Bitplane).<br>Clonal analysis were performed using Fiji's plugin TrakEM2, Matlab (Mathworks) (custom codes, delaunayTriangulation and convexHull functions)<br>Arborizations analysis were performed using Fiji, Vaa3D Matlab (MathWorks), and Autoquant X3.1 (Media Cybernetics)<br>Statistical analysis and graphs were generated with GraphPad Prism 6.

For manuscripts utilizing custom algorithms or software that are central to the research but not yet described in published literature, software must be made available to editors/reviewers upon request. We strongly encourage code deposition in a community repository (e.g. GitHub). See the Nature Research guidelines for submitting code & software for further information.

Data

Policy information about availability of data

All manuscripts must include a data availability statement. This statement should provide the following information, where applicable:

- Accession codes, unique identifiers, or web links for publicly available datasets
- A list of figures that have associated raw data
- A description of any restrictions on data availability

A booklet with individual clones analyzed with ChroMS microscopy is associated with figure 2 and provided as Supplementary Resource.
Detailed maps and sequences of the vectors, clonal analysis details and custom codes are available upon request.
There is no restriction on data availability.

Field-specific reporting

Please select the best fit for your research. If you are not sure, read the appropriate sections before making your selection.

[x] Life sciences [ ] Behavioural & social sciences [ ] Ecological, evolutionary & environmental sciences

For a reference copy of the document with all sections, see nature.com/authors/policies/ReportingSummary-flat.pdf

Life sciences study design

All studies must disclose on these points even when the disclosure is negative.

Sample size	No statistical methods were used to predetermine sample size. Sample size for each experiments is indicated in figure legends and Supplementary Table 2. Animal number was minimized according to ethical guidelines.
Data exclusions	Within animals with the required genotype(s) and/or a large electroporation area, no samples or analyses were excluded in all experiments.
Replication	All experimental findings were reliably reproducible
Randomization	Animals from different litters were chosen based on their correct genotype and/or a large electroporation area. All astrocytes clones were analyzed in these animals, with no preference. For morphological analysis: isolated cells were chosen randomly in equal proportions in the different cortical layers.
Blinding	Blinding was not relevant to this work as no defect was studied and as animal stages were clearly identifiable from cell morphologies.

Reporting for specific materials, systems and methods

Materials & experimental systems

n/a	Involved in the study	
x		Unique biological materials
	x	Antibodies
x		Eukaryotic cell lines
x		Palaeontology
	x	Animals and other organisms
x		Human research participants

Methods

n/a	Involved in the study	
x		ChIP-seq
x		Flow cytometry
x		MRI-based neuroimaging

Antibodies

Antibodies used	rabbit anti-Olig2 (Merck Millipore AB9610)<br>goat anti-Olig2 (R&D systems Bio-Techne AF2418)<br>rabbit anti-Aldh1l1 (Abcam ab87117)<br>rabbit anti-S100β (Abcam ab52642)<br>rabbit anti-Ki67 (Abcam ab15580)<br>goat anti-rabbit Alexa Fluor 647 (Life A21245)<br>donkey anti-goat Alexa Fluor 594 (Life A11058)<br>donkey anti-rabbit Alexa Fluor 647 (Jackson 711-605-152)
Validation	rabbit anti-Olig2 (Merck Millipore AB9610) - validation in mouse manufacturer (http://www.merckmillipore.com/FR/fr/product/Anti-Olig-2-Antibody,MM_NF-AB9610)<br><br>goat anti-Olig2 (R&D systems Bio-Techne AF2418) - validation in mouse: manufacturer (https://www.rndsystems.com/products/human-mouse-rat-olig2-antibody_af2418)<br><br>rabbit anti-Aldh1l1 (Abcam ab87117) - validation in mouse: manufacturer (https://www.abcam.com/aldh1l1-antibody-astrocyte-marker-ab87117.html)<br><br>rabbit anti-S100β (Abcam ab52642) - validation in mouse: manufacturer (https://www.abcam.com/s100-beta-antibody-ep1576y-ab52642.html)<br><br>rabbit anti-Ki67 (Abcam ab15580) - validation in mouse: manufacturer (https://www.abcam.com/ki67-antibody-ab15580.html)<br><br>goat anti-rabbit Alexa Fluor 647 (Life A21245) - validation in mouse: manufacturer (https://www.thermofisher.com/antibody/product/Goat-anti-Rabbit-IgG-H-L-Highly-Cross-Adsorbed-Secondary-Antibody-Polyclonal/A-21245)<br><br>donkey anti-goat Alexa Fluor 594 (Life A11058) - validation in mouse: manufacturer (https://www.thermofisher.com/antibody/product/Donkey-anti-Goat-IgG-H-L-Cross-Adsorbed-Secondary-Antibody-Polyclonal/A-11058)<br><br>donkey anti-rabbit Alexa Fluor 647 (Jackson 711-605-152) - validation in mouse: manufacturer (https://www.jacksonimmuno.com/catalog/products/711-605-152)

Animals and other organisms

Policy information about studies involving animals; ARRIVE guidelines recommended for reporting animal research

Laboratory animals	Experimental mice included both males and females, between the ages of E15-P21, issued from >7 weeks old pregnant females (E13 to P0). The following strains were used:<br><br>Wildtype mice: RjOrl:SWISS (Janvier)<br>CAG-Cytbow mice: pronuclear injection of a CAG-Cytbow transgene linearized with HindIII and MluI<br>Olig2tm2(TVA,cre)Rth (Olig2Cre) mice: gift from Alain Chédotal (Jackson Laboratory 011103)<br><br>All animals were kept under typical day/night conditions of 12-hours cycles.
Wild animals	n/a
Field-collected samples	n/a
