# Supplementary material for: Cortical astrocytes develop in a plastic manner at both clonal and cellular levels
Source: Nat Commun. 2019 Oct 25;10:4884. doi: 10.1038/s41467-019-12791-5 (PMC6814723; doi:10.1038/s41467-019-12791-5)
Supplement: Supplementary file 3 — Description of Additional Supplementary Files [file 41467_2019_12791_MOESM3_ESM.docx]

**Description of Additional Supplementary Files**

File Name: Supplementary Data 1

Description: Visualization of the spatial arrangement of astrocytes clones reconstructed from two P7 and two P21 animals by 3D imaging with ChroMS microscopy. On the left panel of each page, all clones reconstructed in the dataset are displayed in random colors except one individual clone represented in brown (red arrow). This clone is separately enlarged on the right panel with red spheres representing nuclei and brown spheres the astrocyte territory (based on average size values). Connected astrocytes (whose nuclei are closer than the astrocyte average diameter + s.d.) are linked by a blue line.
