# Supplementary material for: Cortical astrocytes develop in a plastic manner at both clonal and cellular levels
Source: Nat Commun. 2019 Oct 25;10:4884. doi: 10.1038/s41467-019-12791-5 (PMC6814723; doi:10.1038/s41467-019-12791-5)
Supplement: Supplementary file 4 — Supplementary Dataset 1 [file 41467_2019_12791_MOESM4_ESM.pdf]

P7 Clones

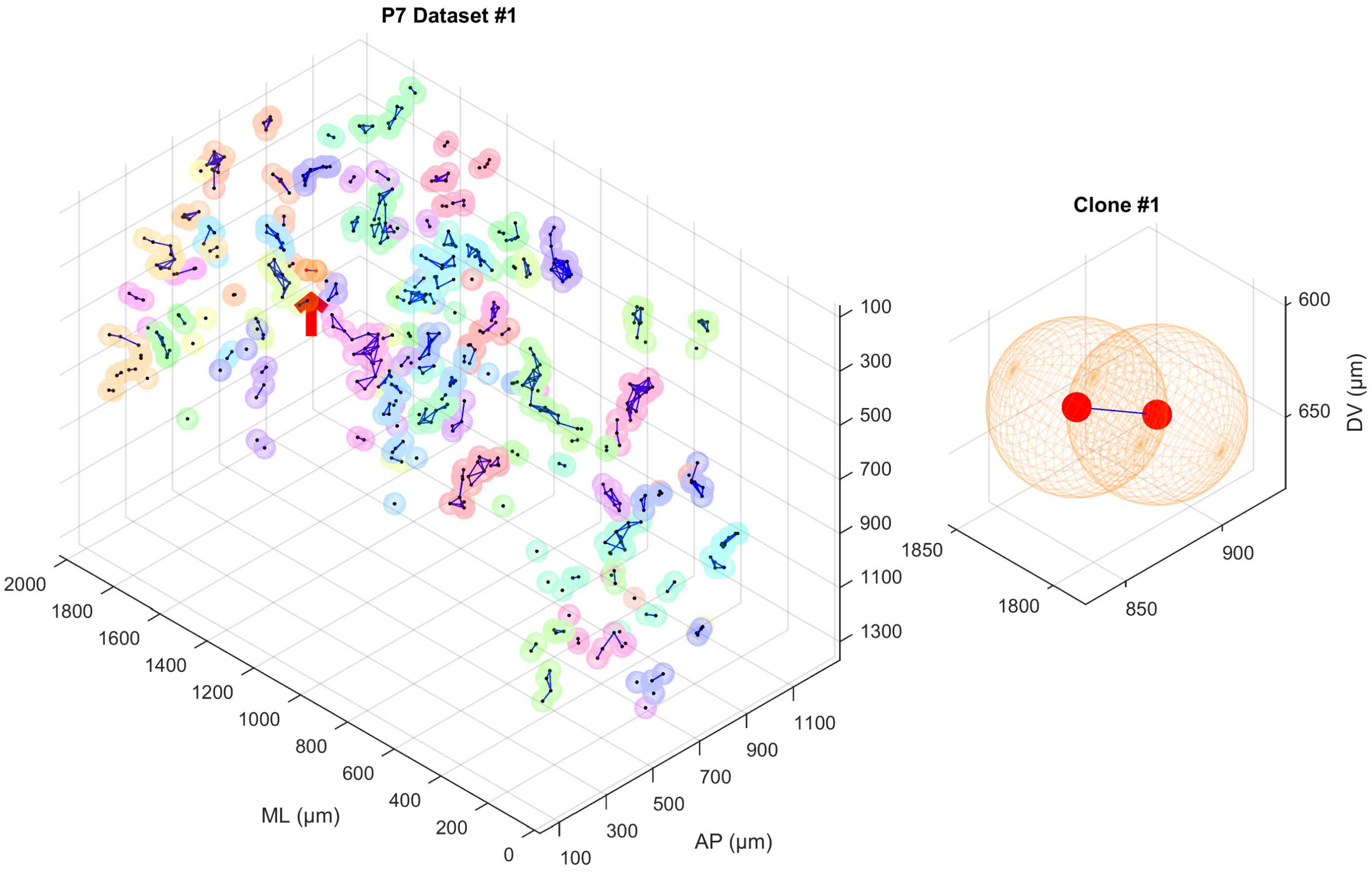

P7 Dataset #1
100
300
500
700
900
1100
1300
2000
1800
1600
1400
1200
1000
800
600
400
200
0
ML (µm)
100
300
500
700
900
1100
AP (µm)
Clone #1
600
650
DV (µm)
1850
1800
850
900

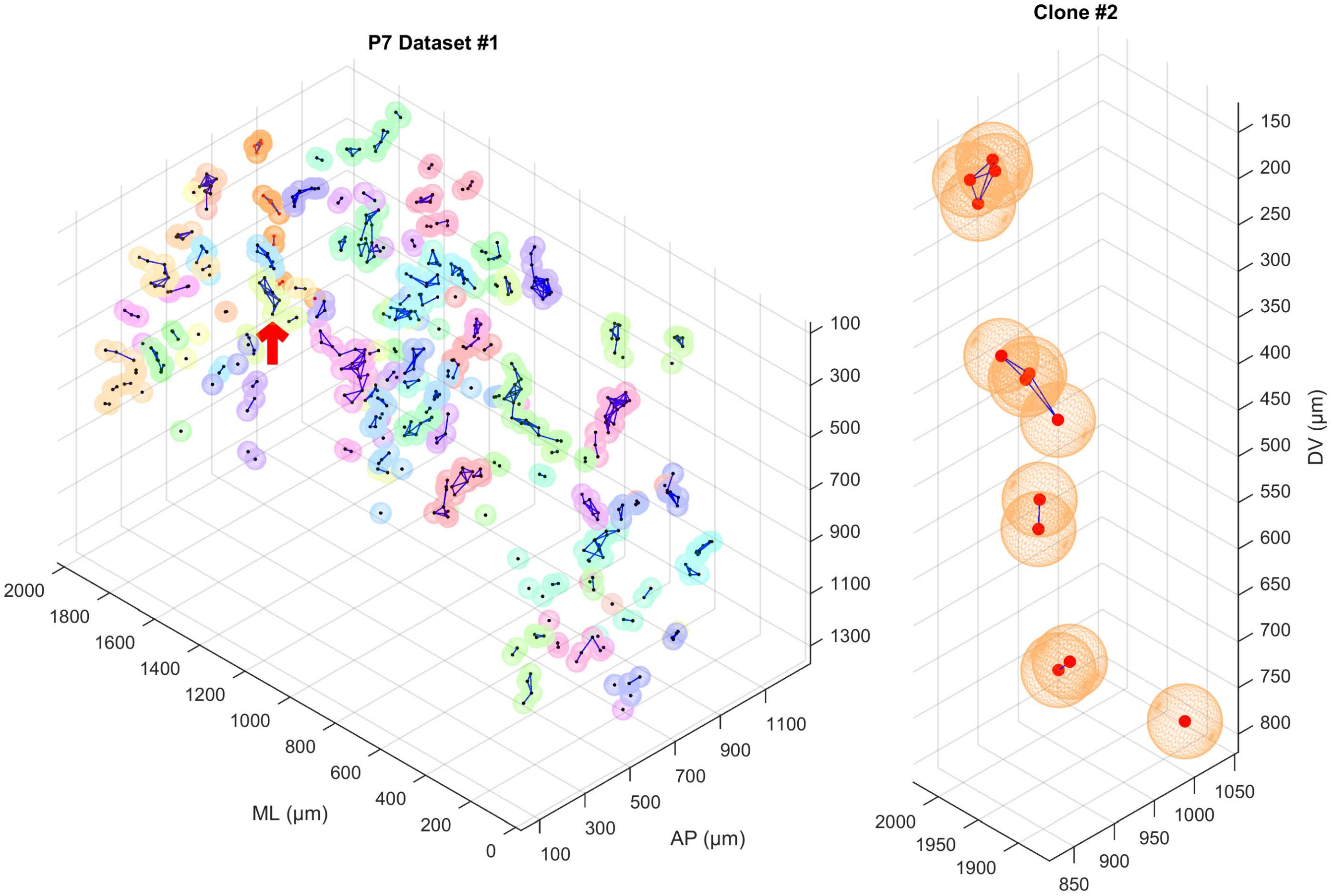

P7 Dataset #1
100
300
500
700
900
1100
1300
2000
1800
1600
1400
1200
1000
800
600
400
200
0
ML (µm)
100
300
500
700
900
1100
AP (µm)
Clone #2
150
200
250
300
350
400
450
500
550
600
650
700
750
800
DV (µm)
2000
1950
1900
850
900
950
1000
1050

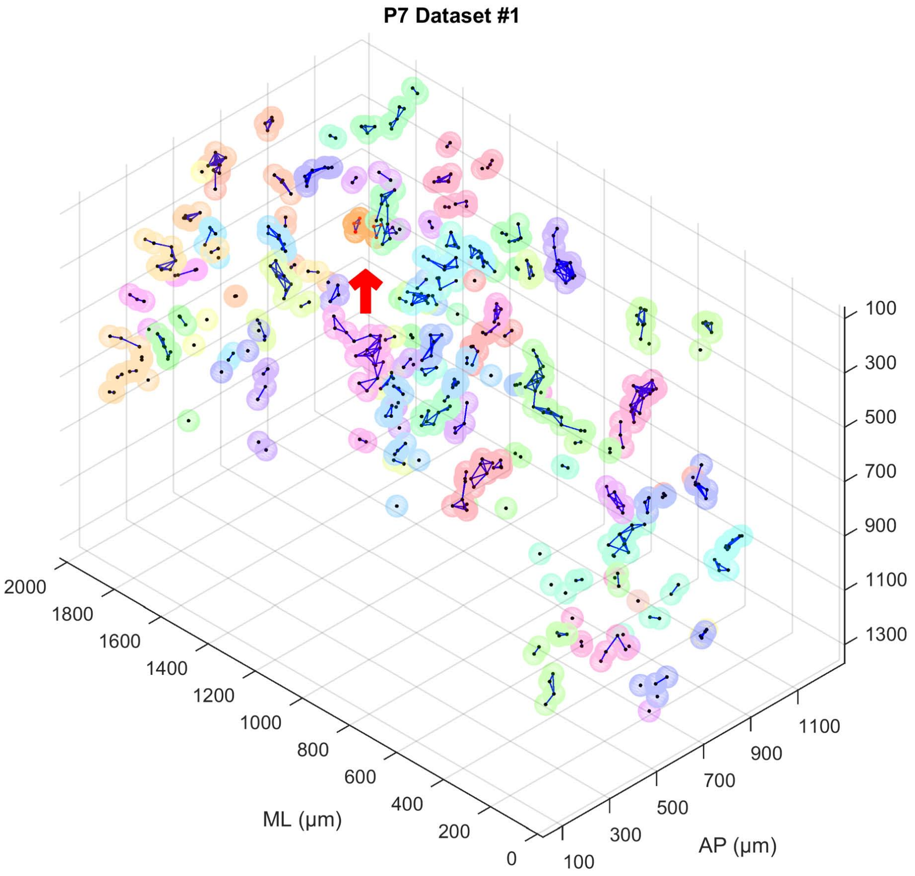
P7 Dataset #1
100
300
500
700
900
1100
1300
2000
1800
1600
1400
1200
1000
800
600
400
200
0
ML (µm)
100
300
500
700
900
1100
AP (µm)

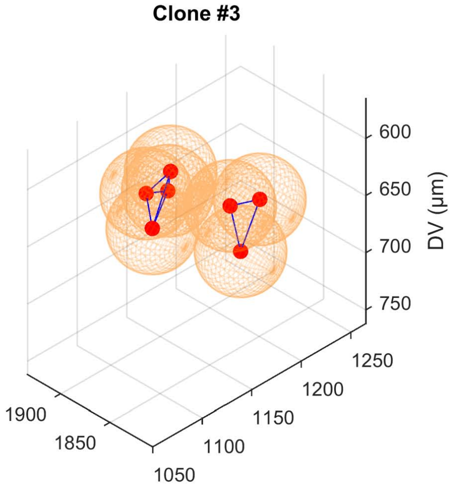
Clone #3
600
650
700
750
DV (µm)
1900
1850
1050
1100
1150
1200
1250

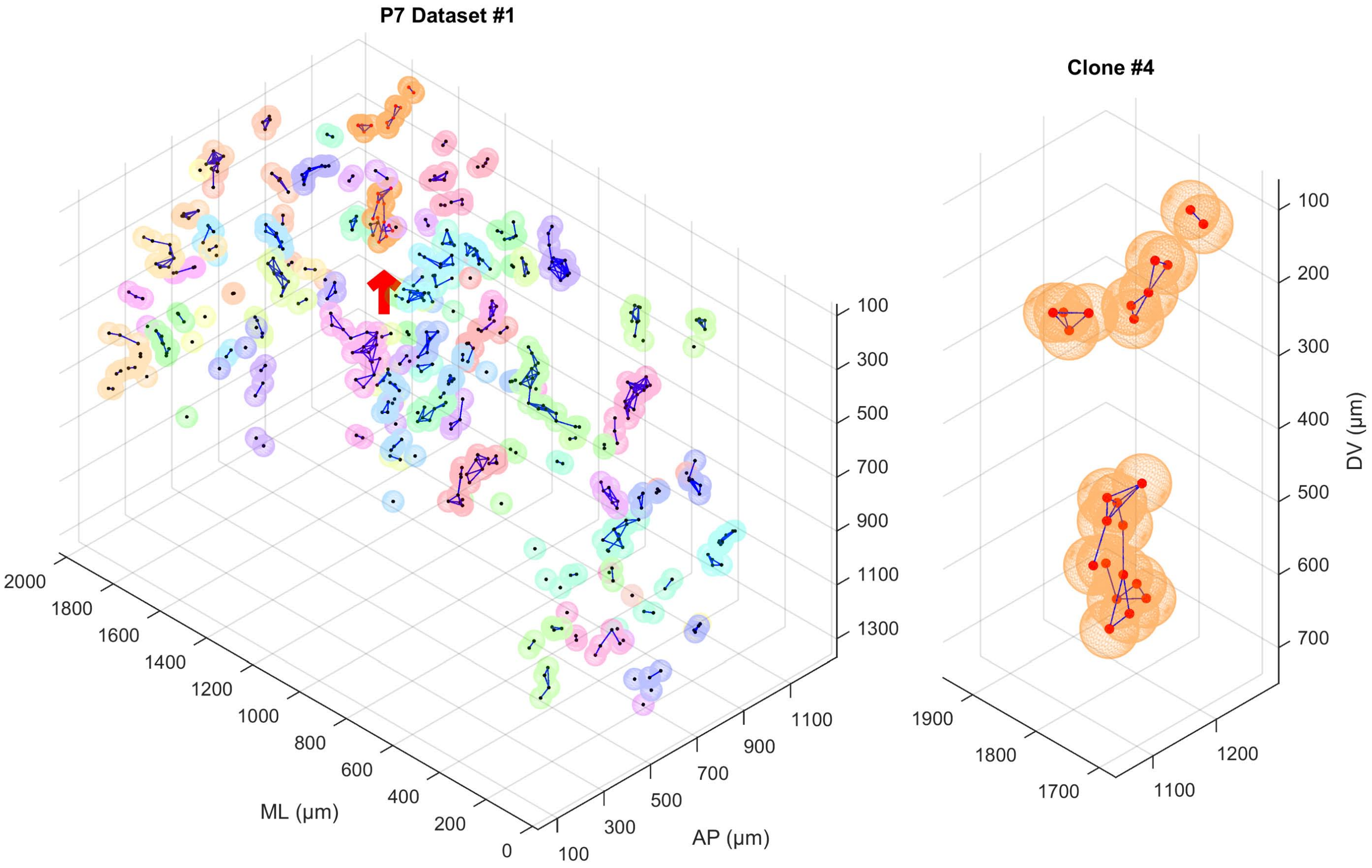
P7 Dataset #1
Clone #4
ML (μm)
AP (μm)
DV (μm)
2000
1800
1600
1400
1200
1000
800
600
400
200
0
100
300
500
700
900
1100
1300
1900
1700

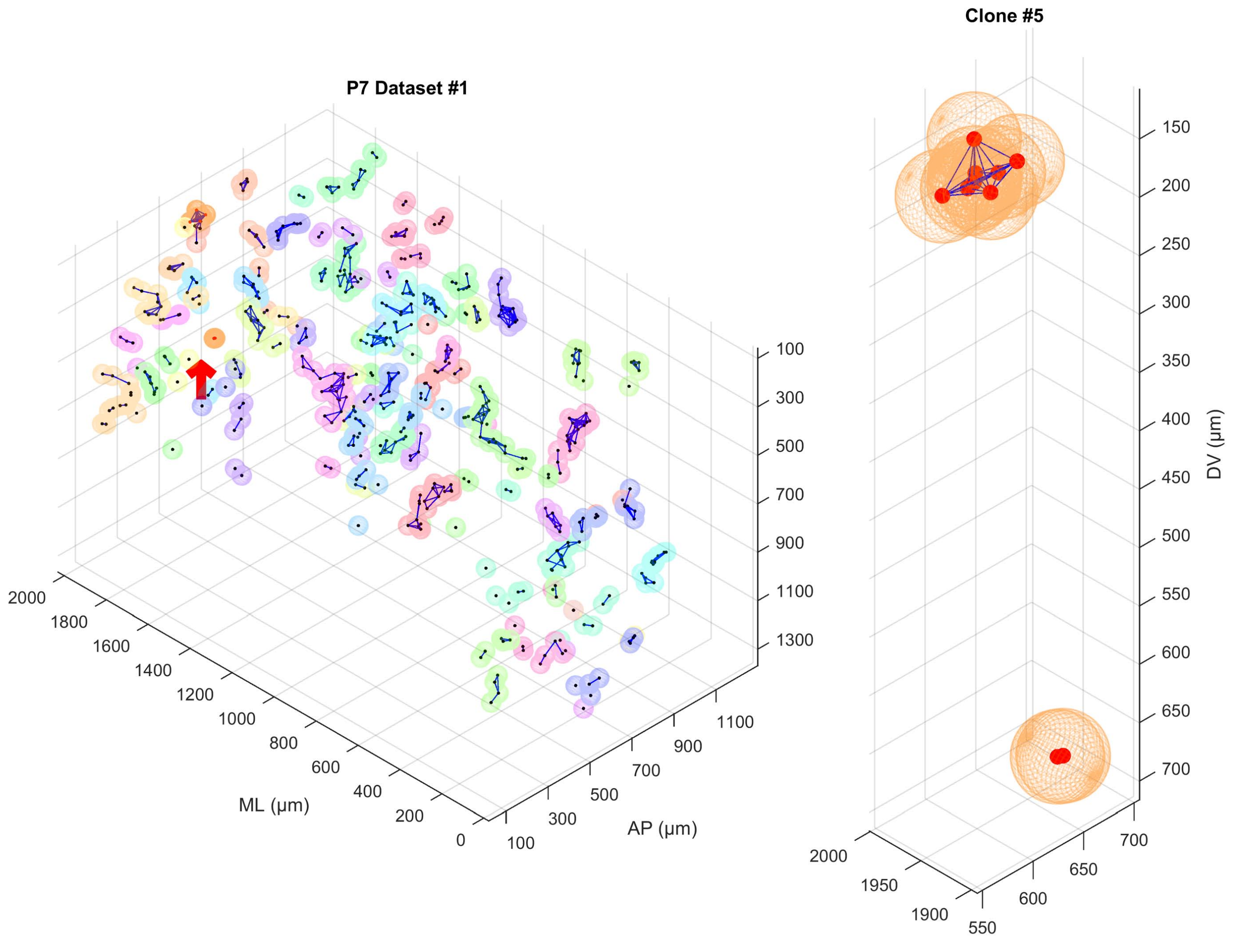
P7 Dataset #1
ML (µm)
2000
1800
1600
1400
1200
1000
800
600
400
200
0
AP (µm)
100
300
500
700
900
1100
100
300
500
700
900
1100
1300
Clone #5
DV (µm)
150
200
250
300
350
400
450
500
550
600
650
700
2000
1950
1900
550
600
650
700

P7 Dataset #1

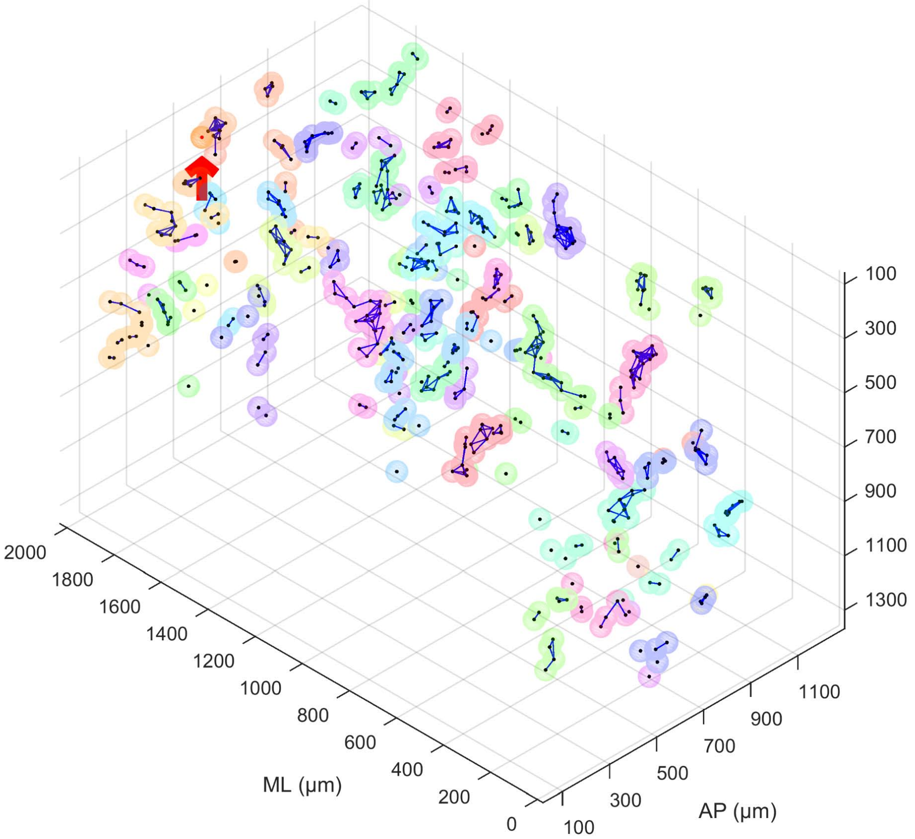

Clone #6

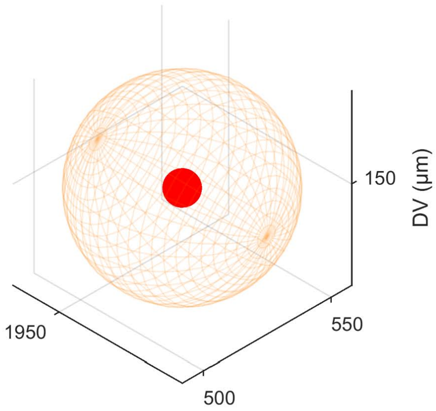

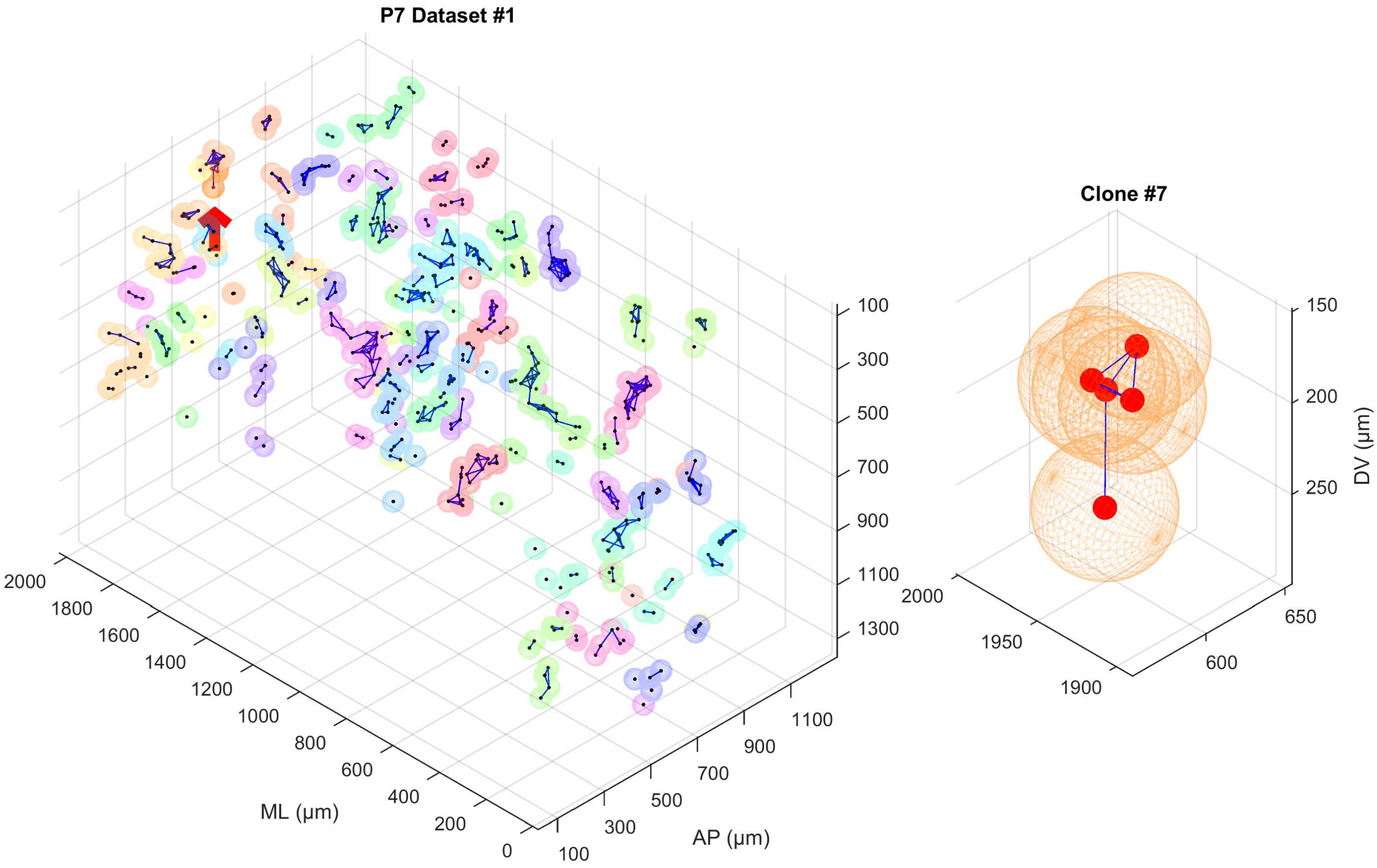

P7 Dataset #1
Clone #7
ML (µm)
AP (µm)
DV (µm)
2000
1800
1600
1400
1200
1000
800
600
400
200
0
100
300
500
700
900
1100
1300
150
200
250
1950
1900
600
650

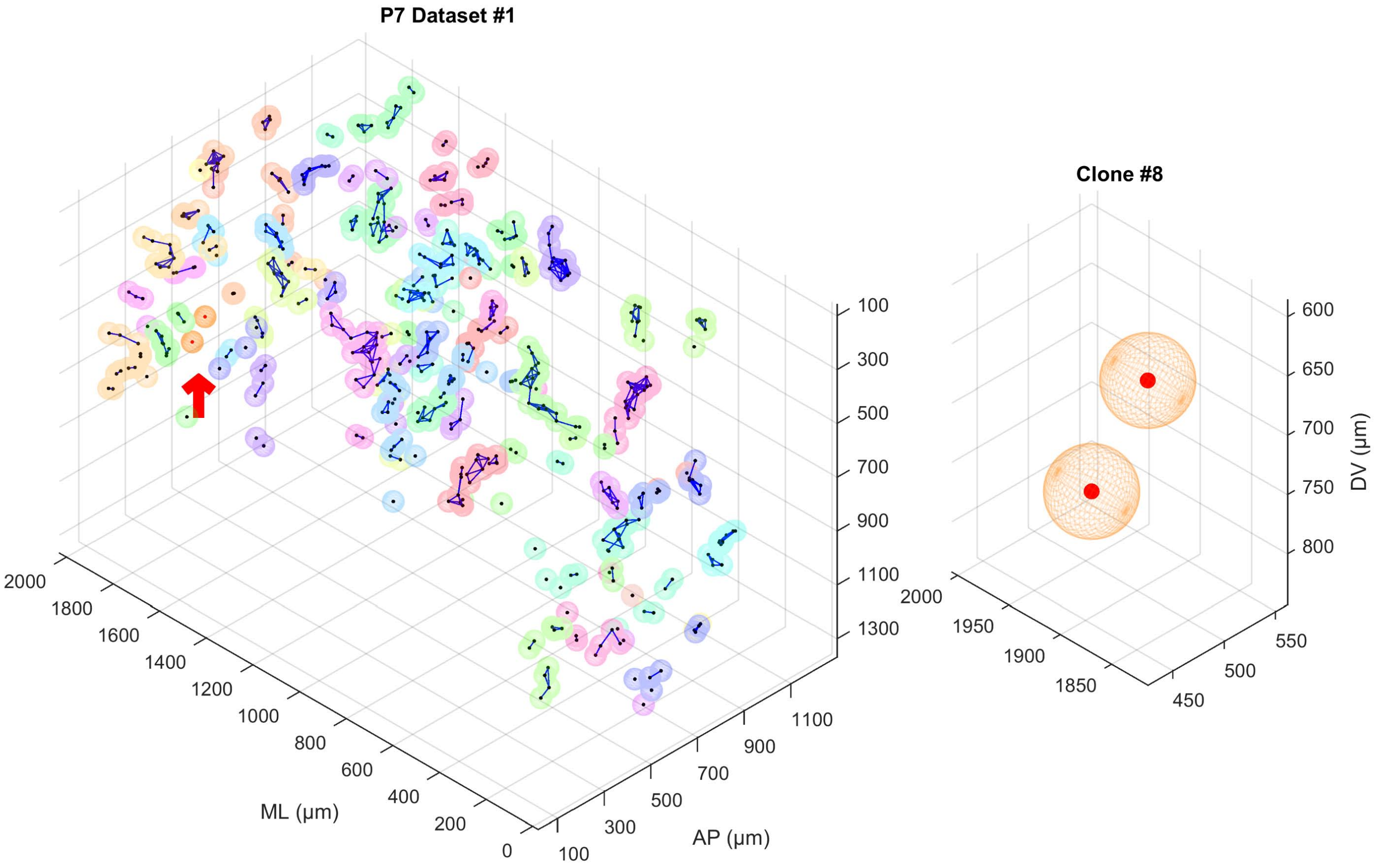

P7 Dataset #1
100
300
500
700
900
1100
1300
2000
1800
1600
1400
1200
1000
800
600
400
200
0
ML (µm)
100
300
500
700
900
1100
AP (µm)
Clone #8
600
650
700
750
800
DV (µm)
2000
1950
1900
1850
450
500
550

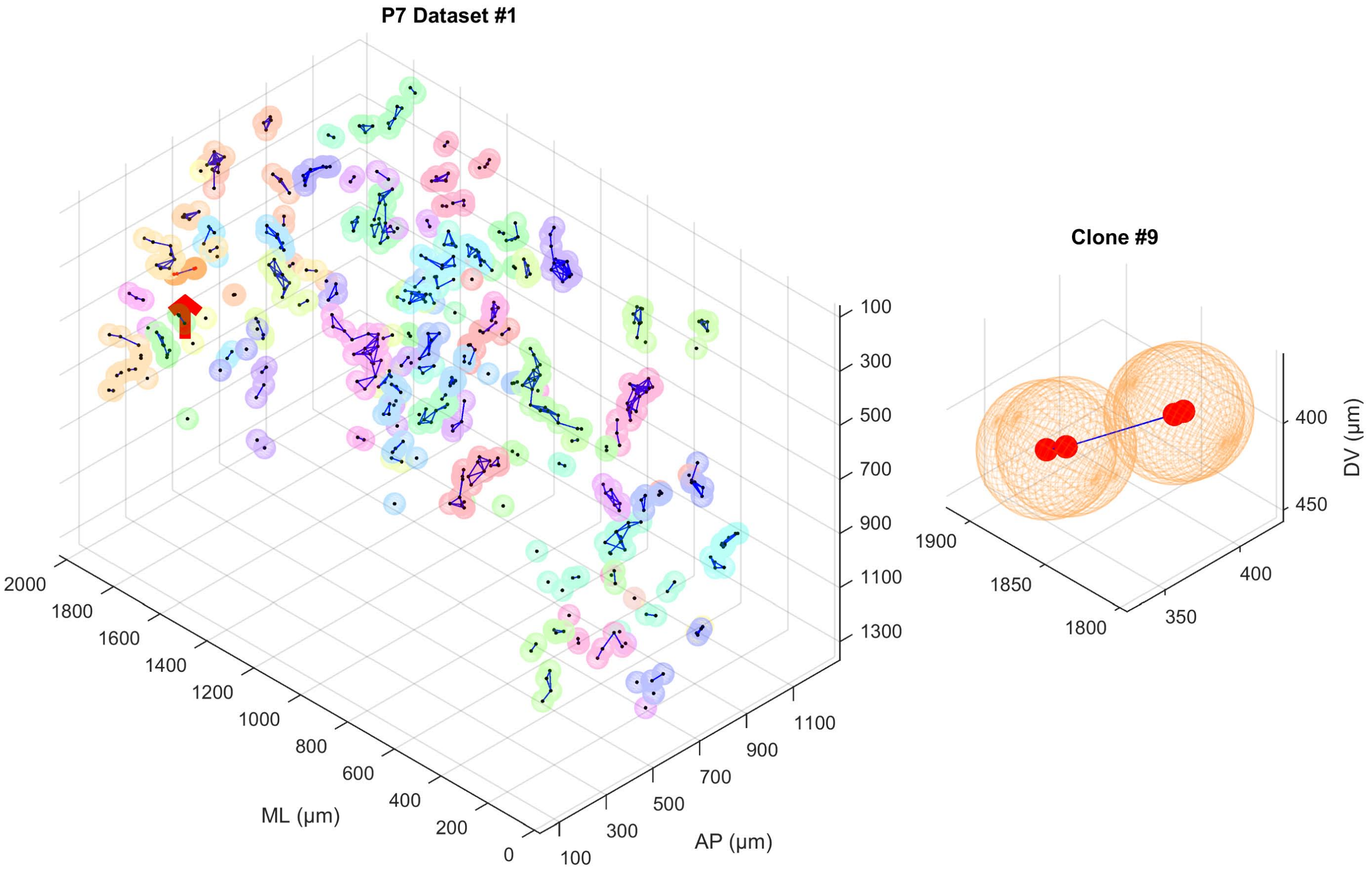
P7 Dataset #1
ML (µm)
AP (µm)
2000
1800
1600
1400
1200
1000
800
600
400
200
0
100
300
500
700
900
1100
100
300
500
700
900
1100
1300
Clone #9
DV (µm)
400
450
1900
1850
1800
350
400

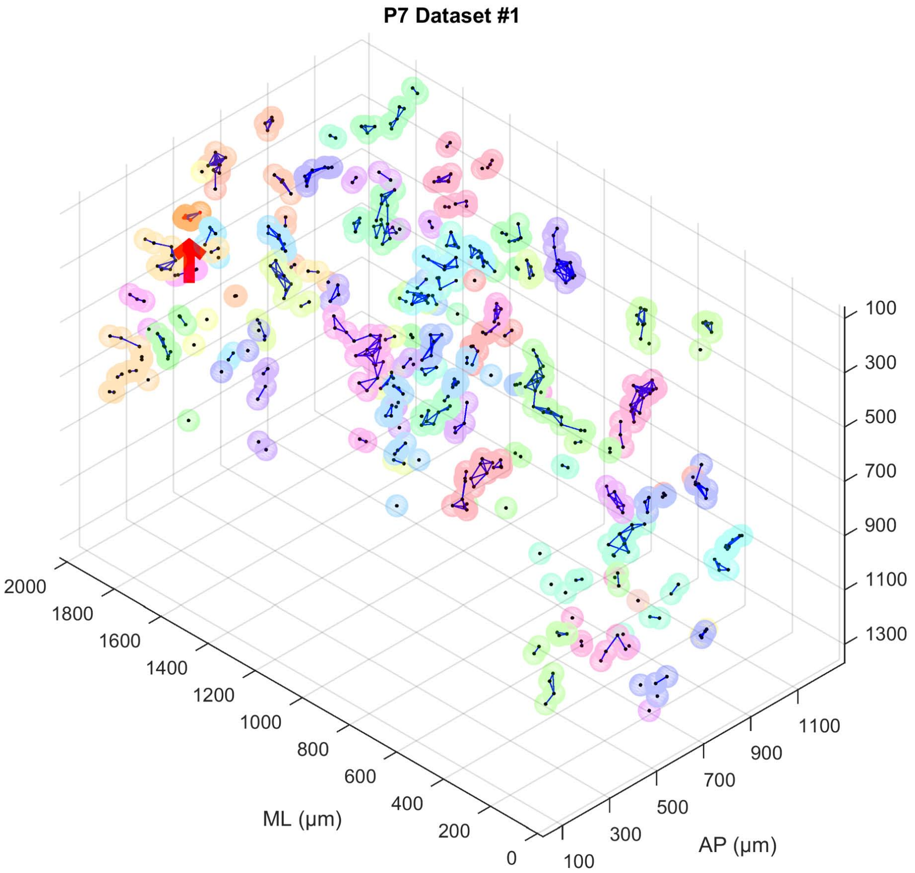
P7 Dataset #1
100
300
500
700
900
1100
1300
2000
1800
1600
1400
1200
1000
800
600
400
200
0
ML (µm)
100
300
500
700
900
1100
AP (µm)

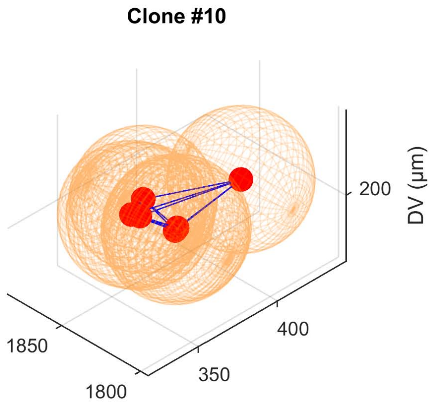
Clone #10
200
DV (µm)
1850
1800
350
400

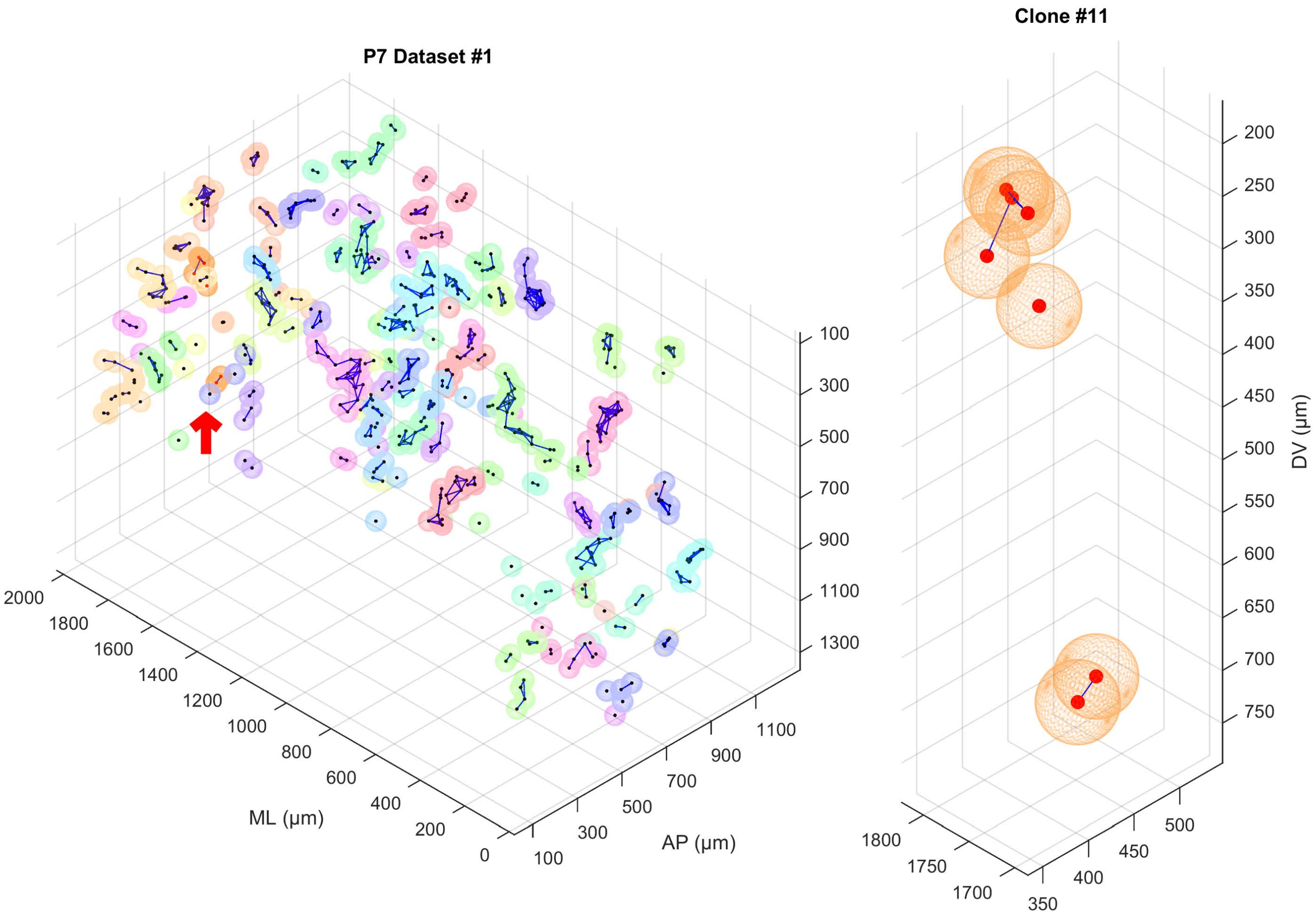

P7 Dataset #1
Clone #11
ML (µm)
AP (µm)
DV (µm)
2000
1800
1600
1400
1200
1000
800
600
400
200
0
100
300
500
700
900
1100
1300
1750
1700
350
400
450
500
200
250
300
350
400
450
500
550
600
650
700
750

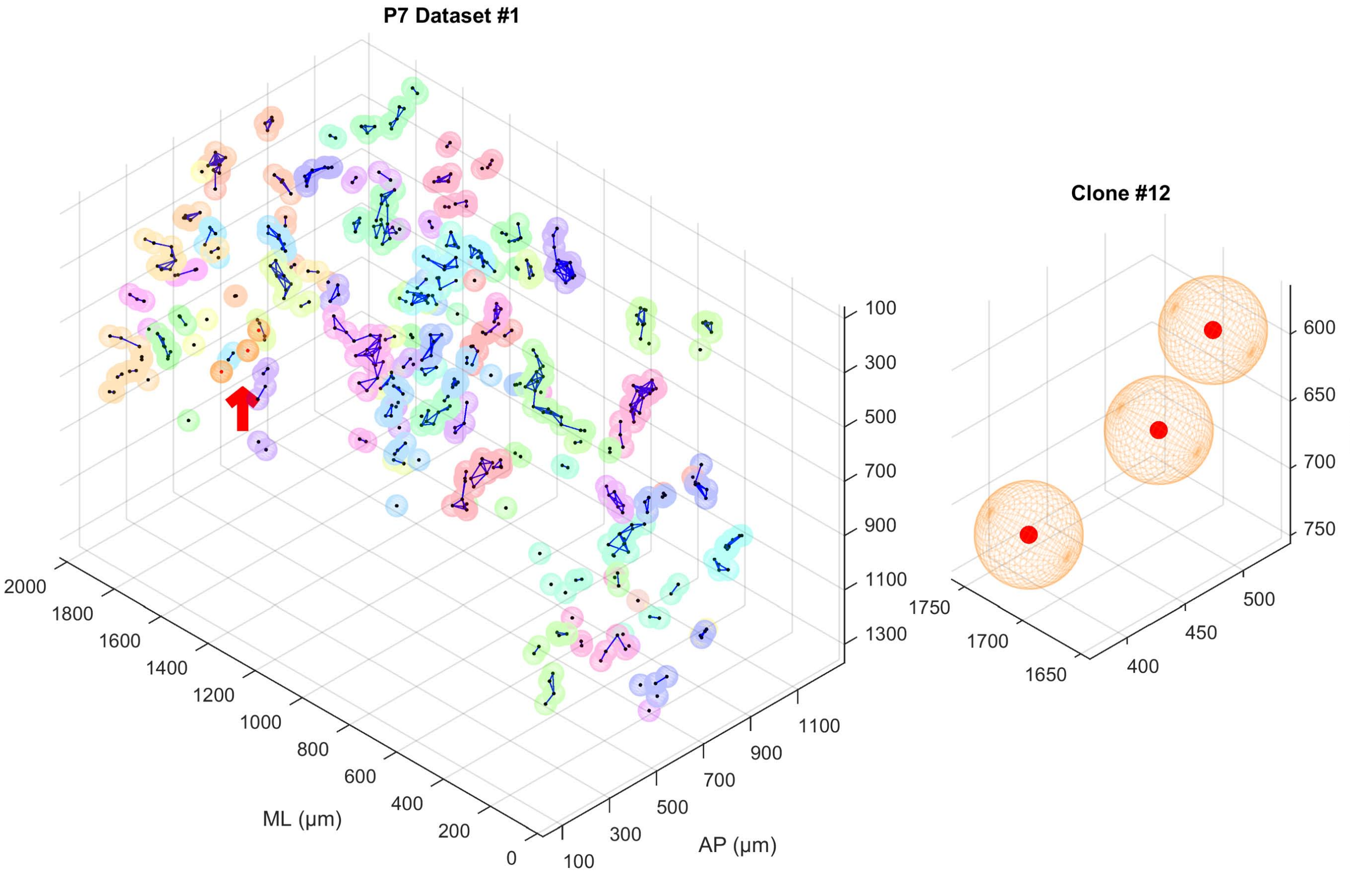

P7 Dataset #1
ML (µm)
2000
1800
1600
1400
1200
1000
800
600
400
200
0
AP (µm)
100
300
500
700
900
1100
100
300
500
700
900
1100
1300
Clone #12
DV (µm)
600
650
700
750
1750
1700
1650
400
450
500

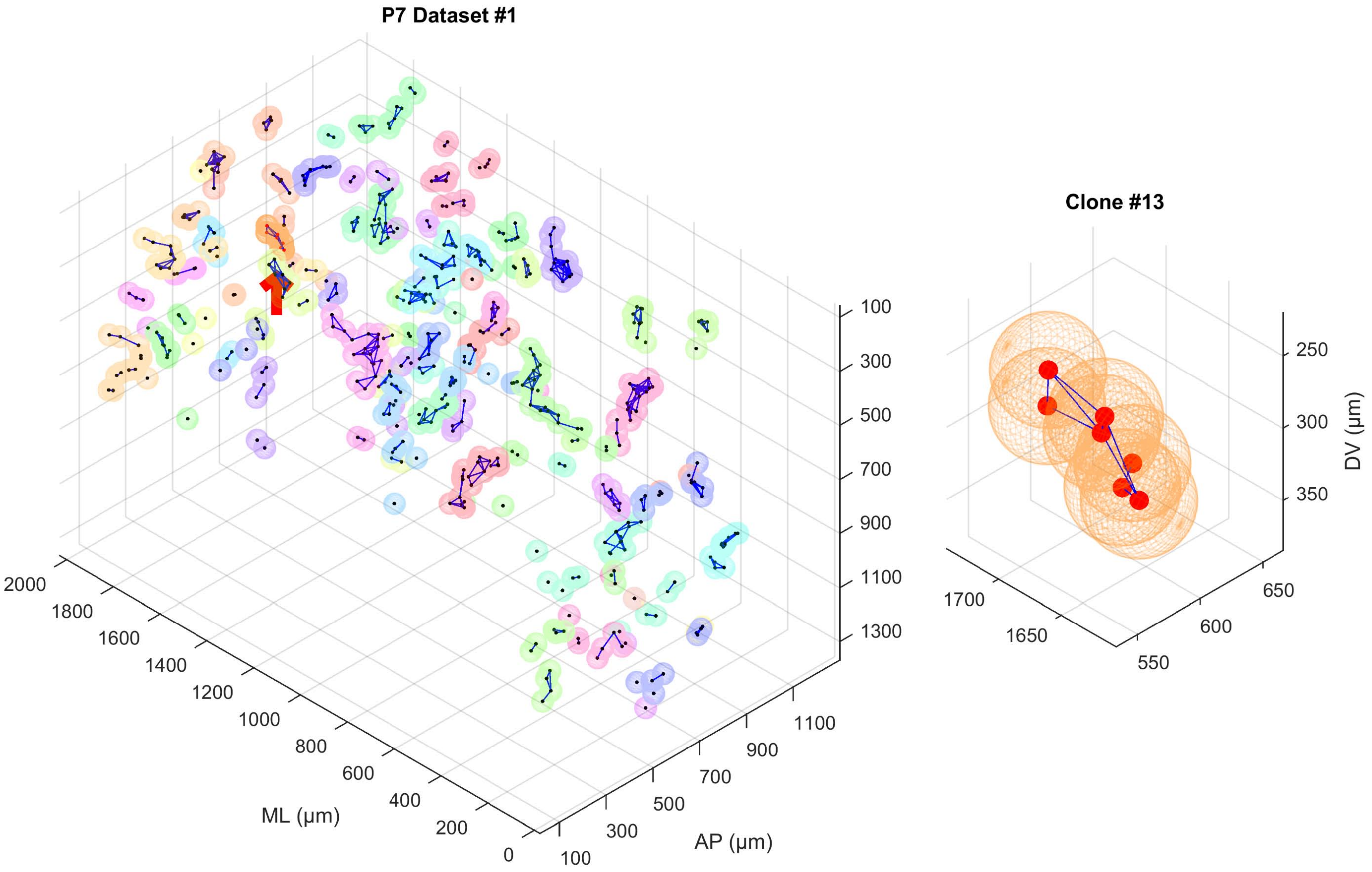

P7 Dataset #1
100
300
500
700
900
1100
1300
2000
1800
1600
1400
1200
1000
800
600
400
200
0
ML (µm)
100
300
500
700
900
1100
AP (µm)
Clone #13
250
300
350
DV (µm)
1700
1650
550
600
650

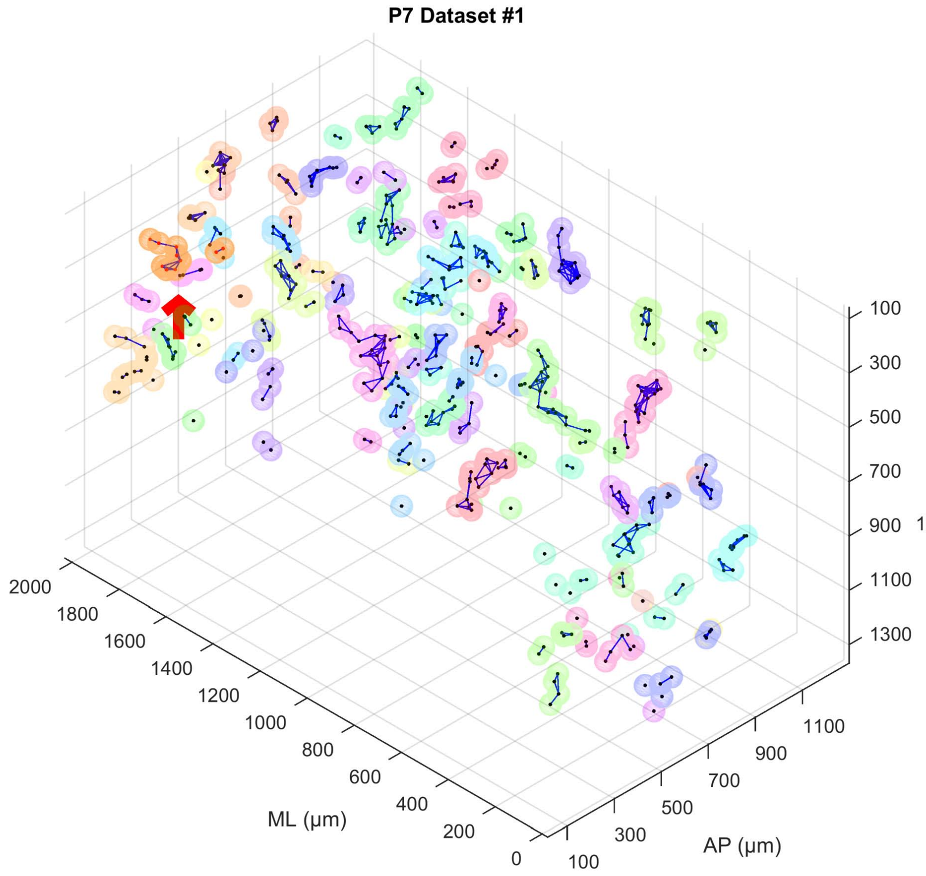

P7 Dataset #1
100
300
500
700
900
1100
1300
2000
1800
1600
1400
1200
1000
800
600
400
200
0
ML (μm)
100
300
500
700
900
1100
AP (μm)

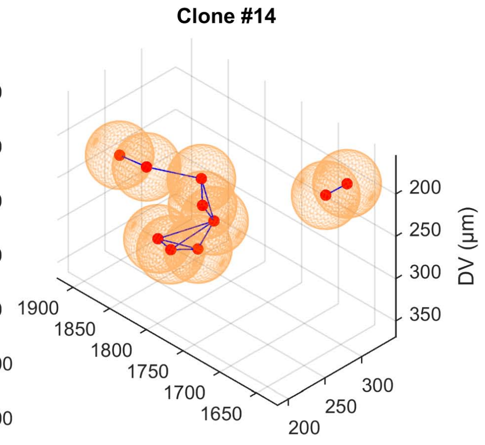

Clone #14
200
250
300
350
DV (μm)
1900
1850
1800
1750
1700
1650
200
250
300

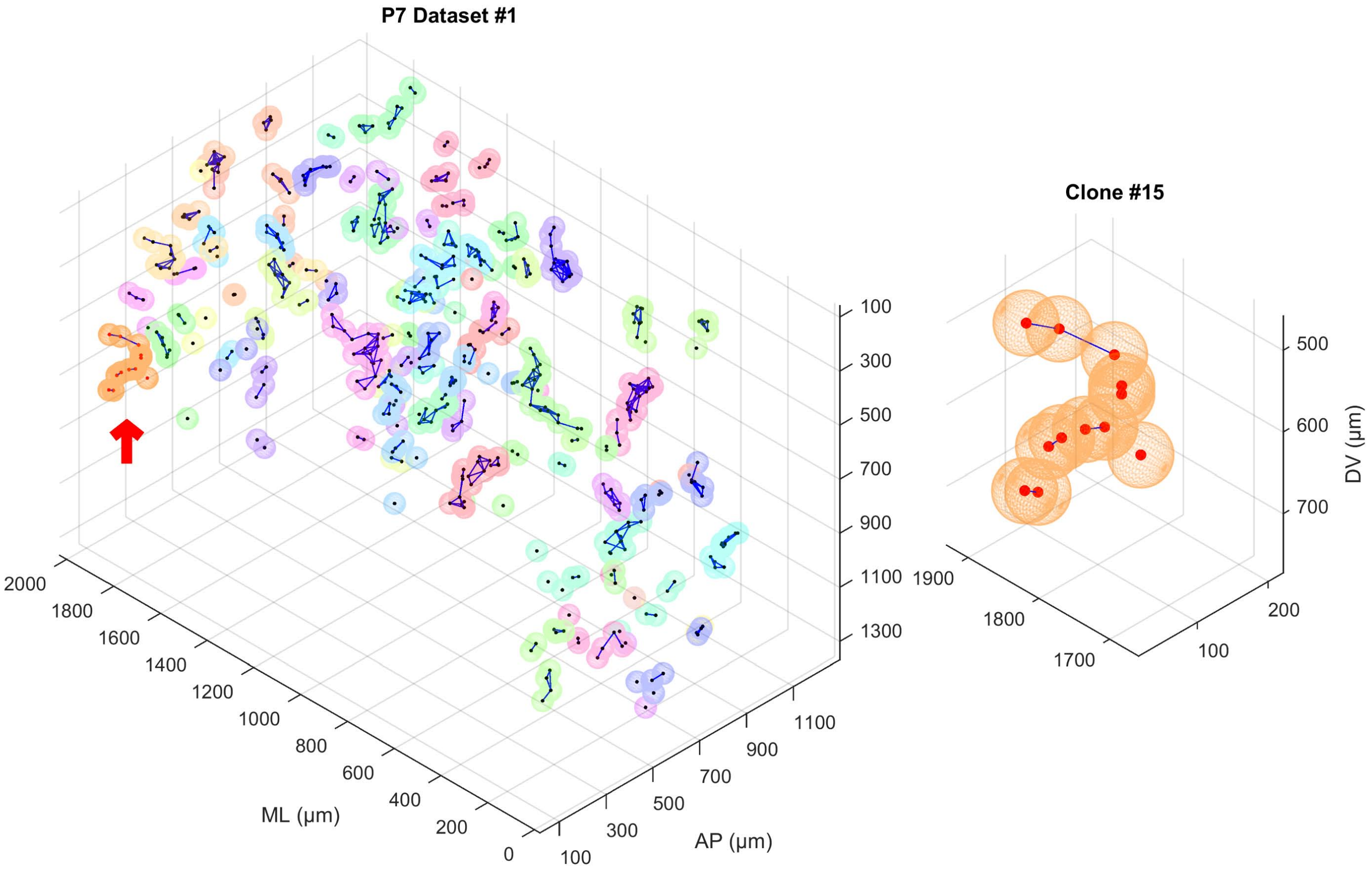

P7 Dataset #1
ML (µm)
AP (µm)
2000
1800
1600
1400
1200
1000
800
600
400
200
0
100
300
500
700
900
1100
1300
Clone #15
DV (µm)
500
600
700
1900
1800
1700
200
100

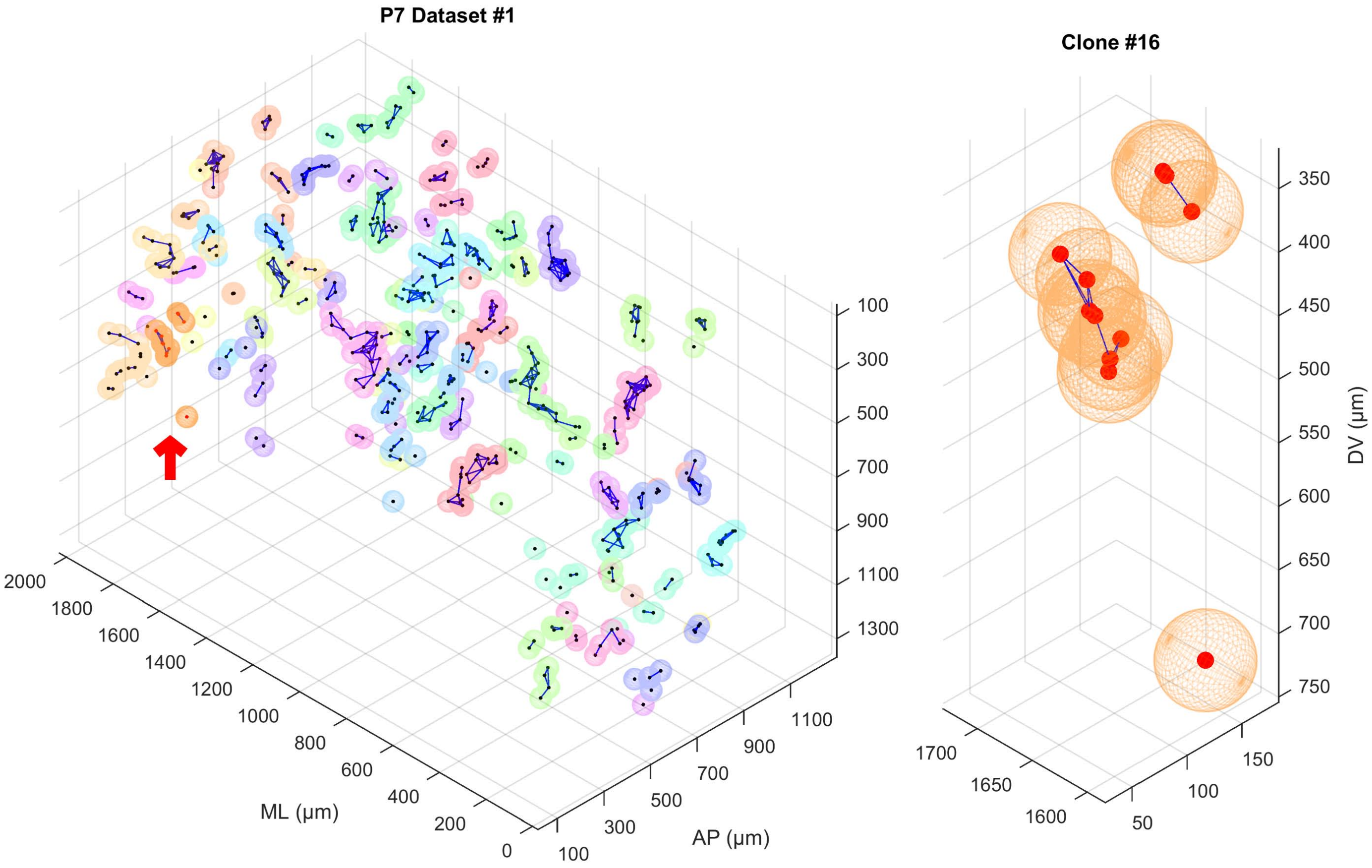

P7 Dataset #1
Clone #16
ML (µm)
AP (µm)
DV (µm)
2000
1800
1600
1400
1200
1000
800
600
400
200
0
100
300
500
700
900
1100
1300
350
400
450
500
550
600
650
750
1700
1650
1600
50
150

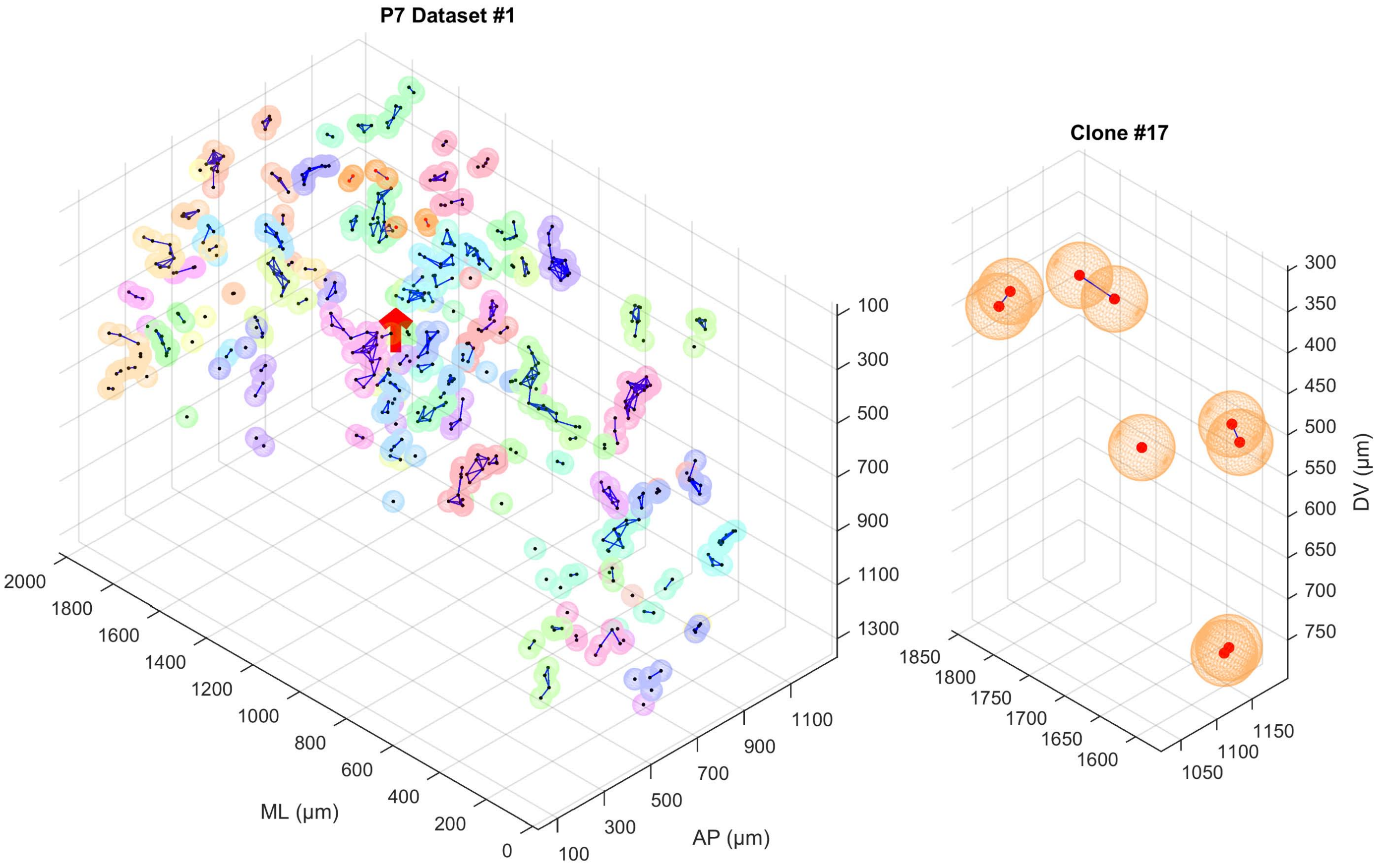

P7 Dataset #1
ML (µm)
2000
1800
1600
1400
1200
1000
800
600
400
200
0
AP (µm)
100
300
500
700
900
1100
100
300
500
700
900
1100
1300
Clone #17
DV (µm)
300
350
400
450
500
550
600
650
700
750
1850
1800
1750
1700
1650
1600
1050
1100
1150

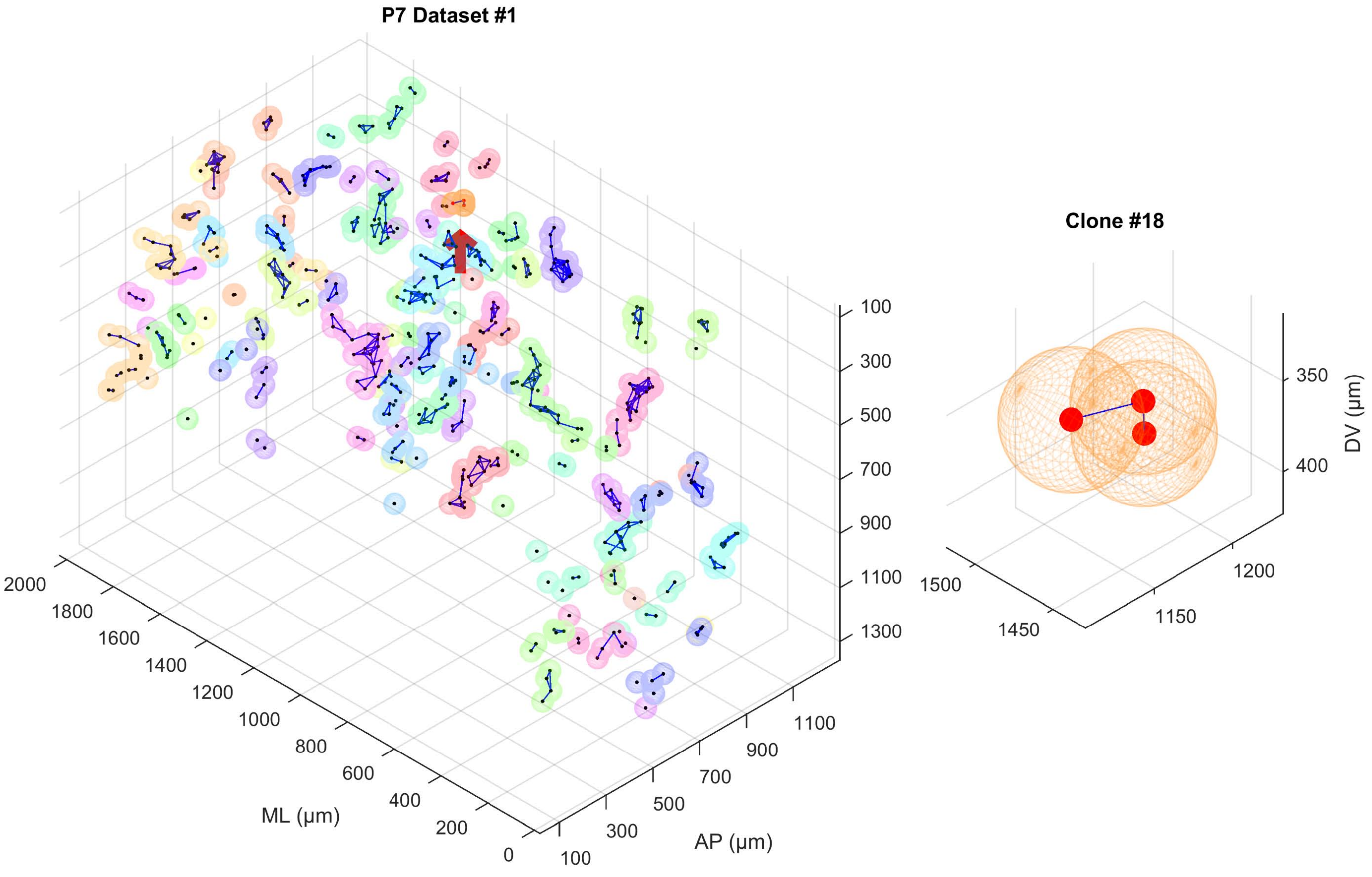
P7 Dataset #1
ML (µm)
AP (µm)
2000
1800
1600
1400
1200
1000
800
600
400
200
0
100
300
500
700
900
1100
100
300
500
700
900
1100
1300
Clone #18
DV (µm)
350
400
1500
1450
1150
1200

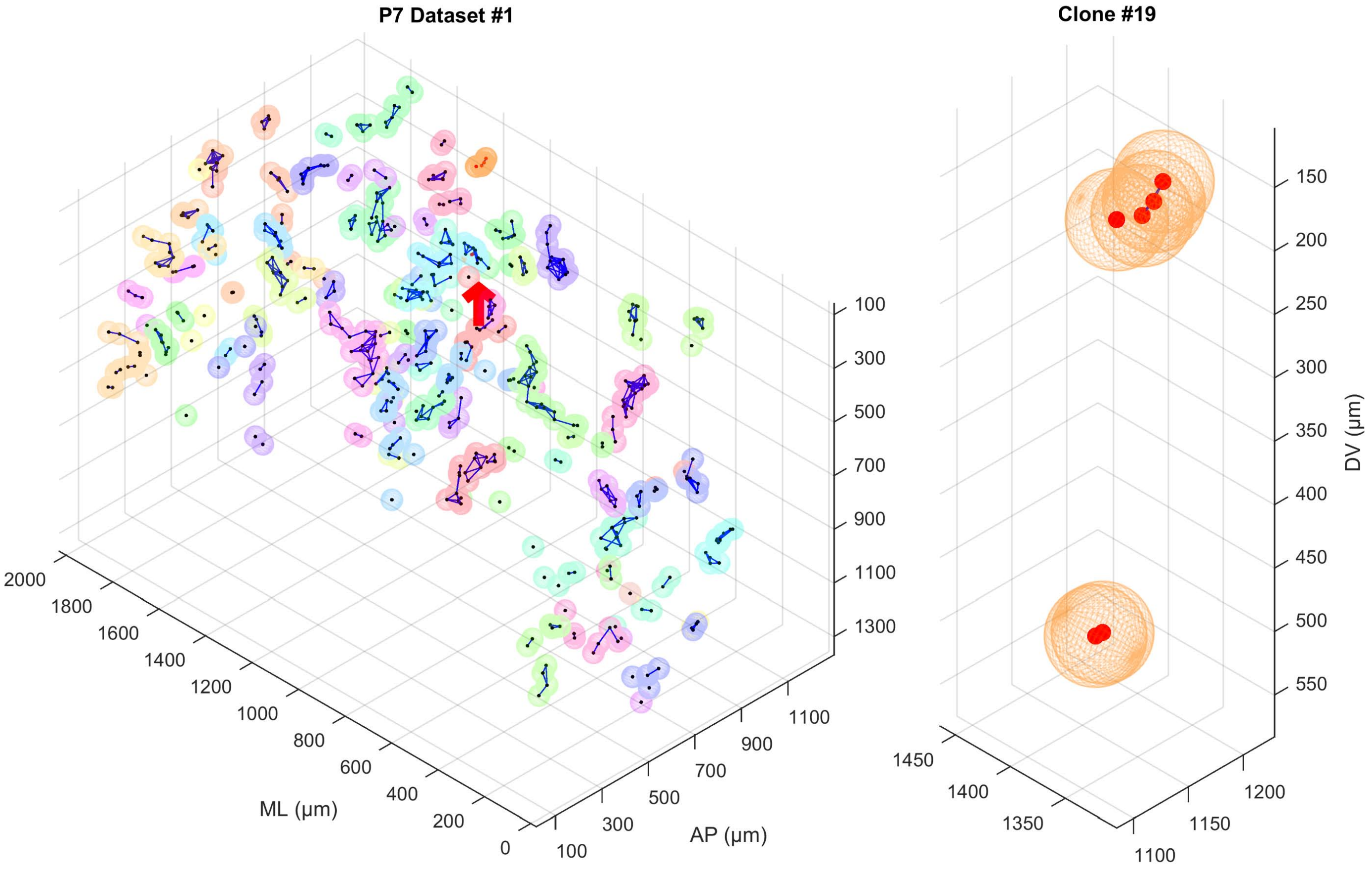

P7 Dataset #1
Clone #19
ML (μm)
AP (μm)
DV (μm)
2000
1800
1600
1400
1200
1000
800
600
400
200
0
100
300
500
700
900
1100
1300
150
200
250
300
350
400
450
500
550
1450
1400
1350
1100
1150
1200

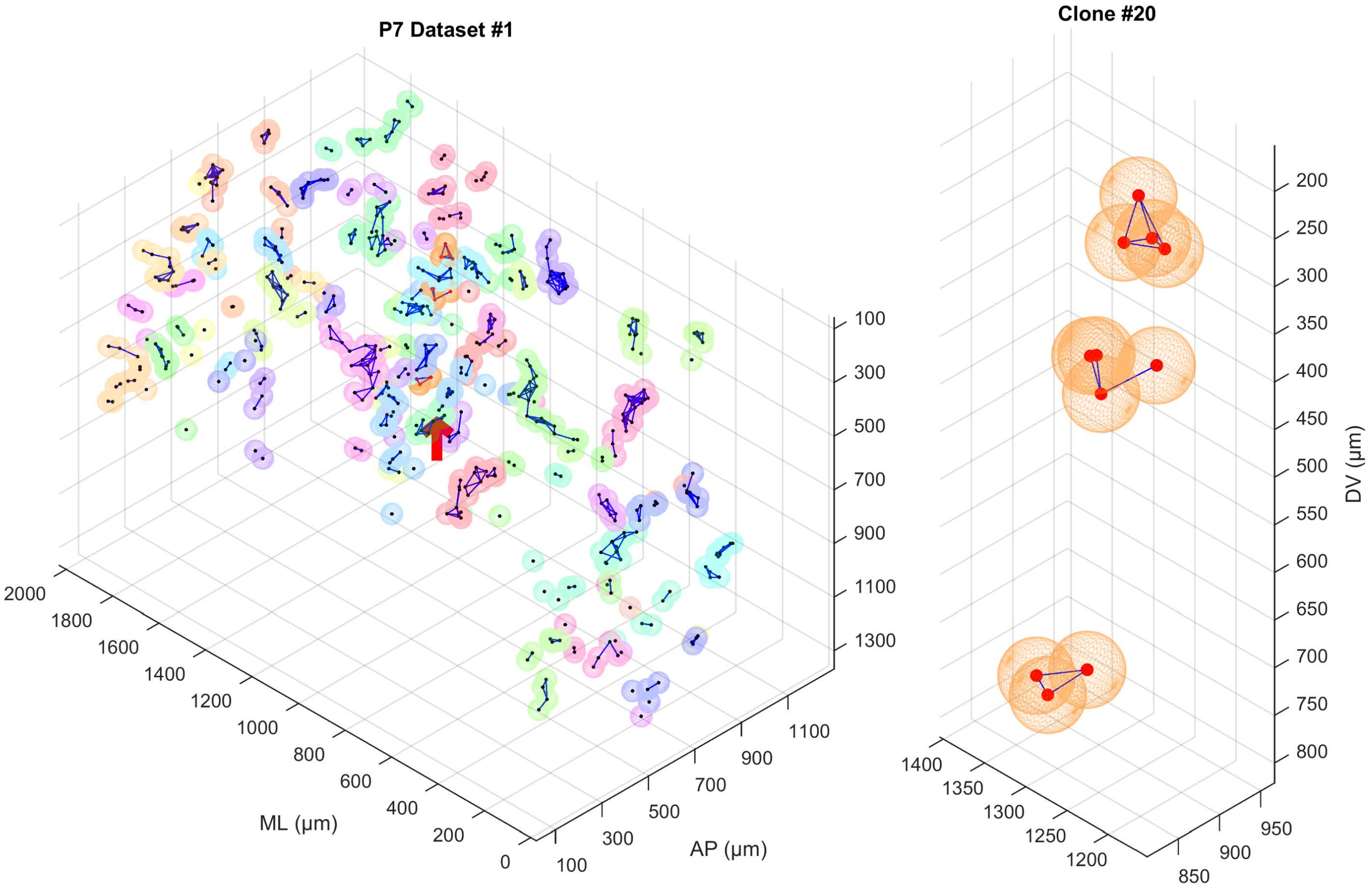

P7 Dataset #1
ML (µm)
AP (µm)
2000
1800
1600
1400
1200
1000
800
600
400
200
0
100
300
500
700
900
1100
1300
Clone #20
DV (µm)
200
250
300
350
400
450
500
550
600
650
700
750
800
1400
1350
1300
1250
1200
850
900
950

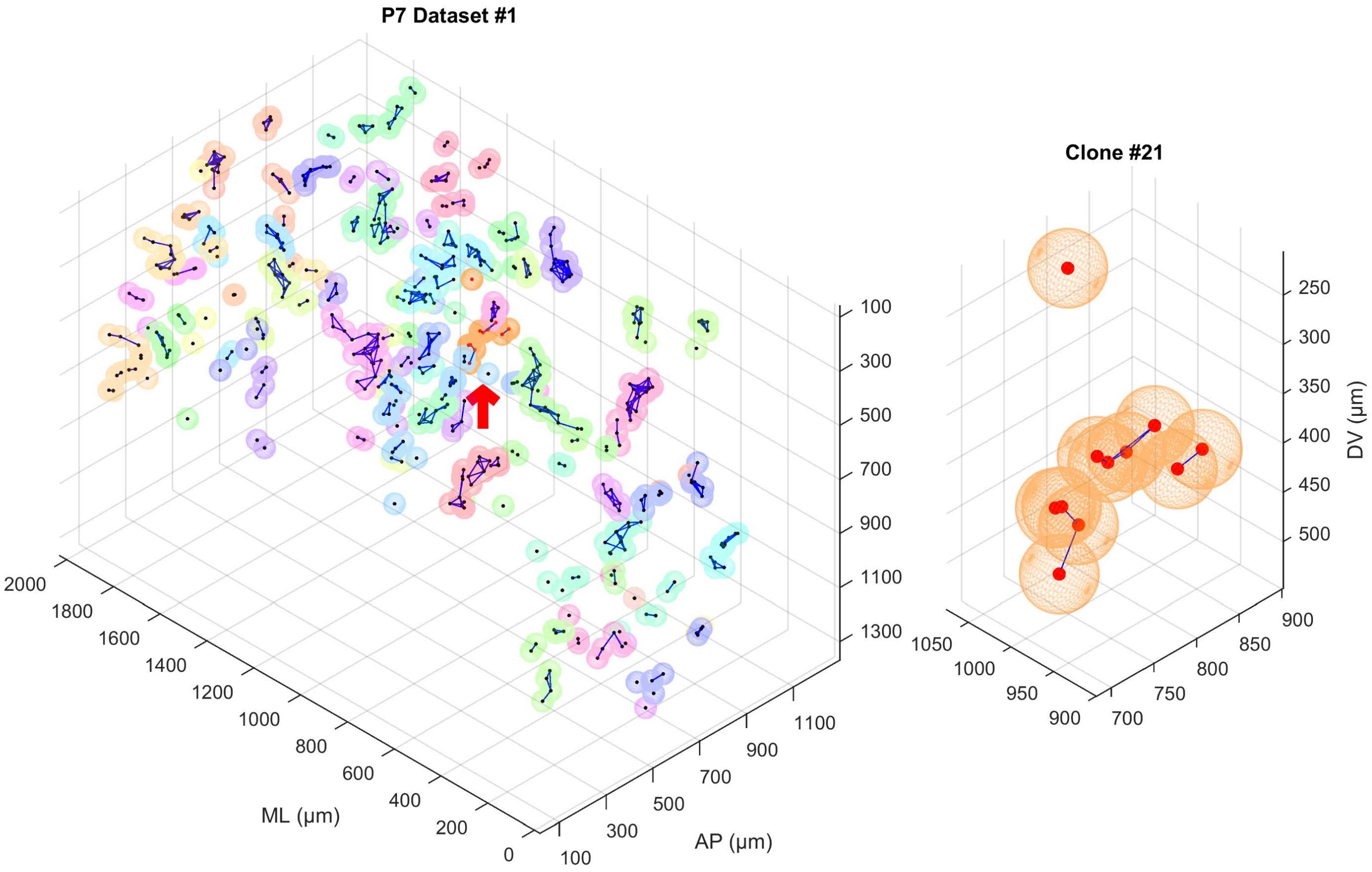
P7 Dataset #1
100
300
500
700
900
1100
1300
2000
1800
1600
1400
1200
1000
800
600
400
200
0
ML (µm)
100
300
500
700
900
1100
AP (µm)
Clone #21
250
300
350
400
450
500
DV (µm)
1050
1000
950
900
700
750
800
850
900

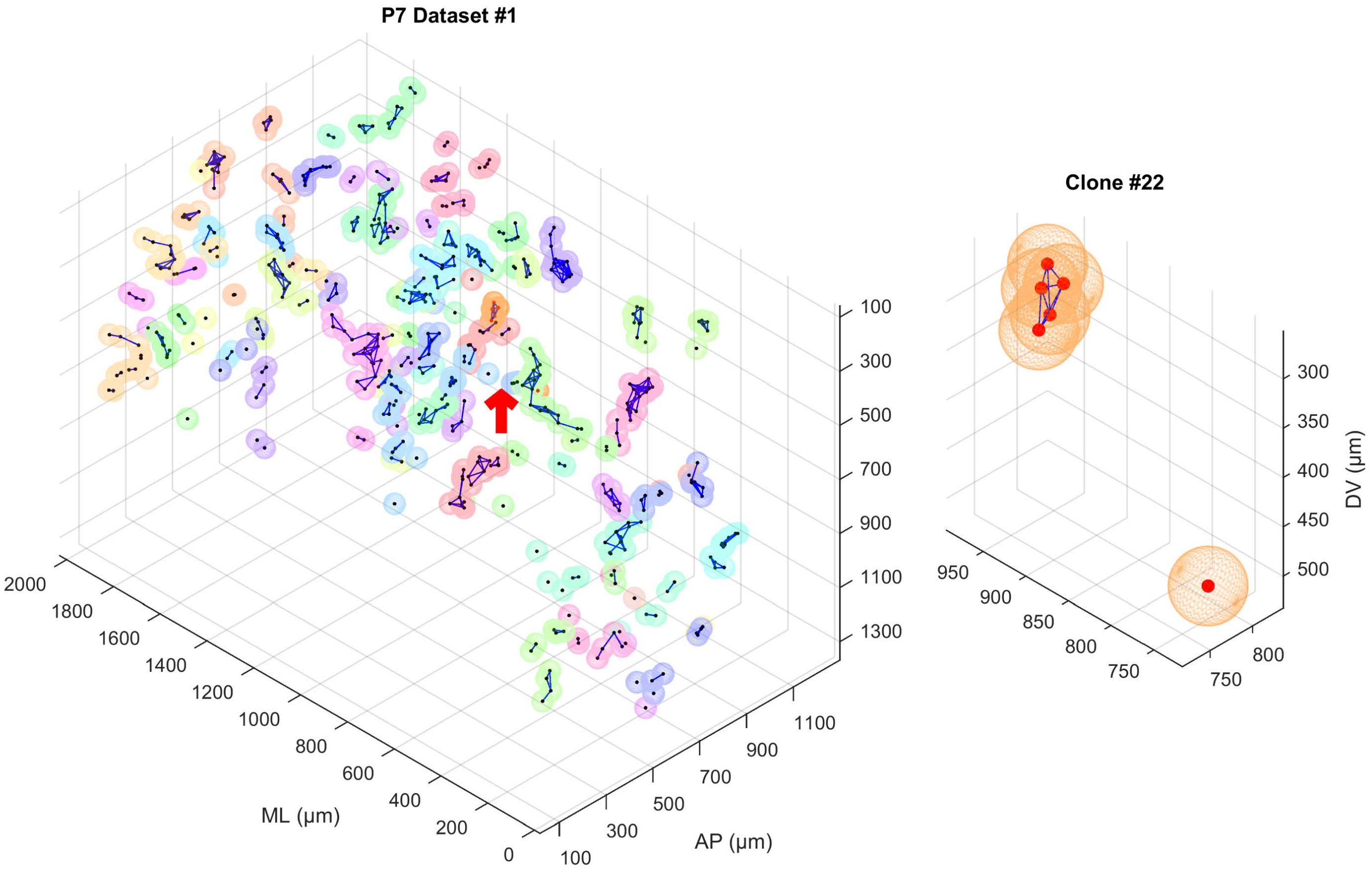

P7 Dataset #1
100
300
500
700
900
1100
1300
2000
1800
1600
1400
1200
1000
800
600
400
200
0
ML (µm)
100
300
500
700
900
1100
AP (µm)
Clone #22
300
350
400
450
500
DV (µm)
950
900
850
800
750
800
750

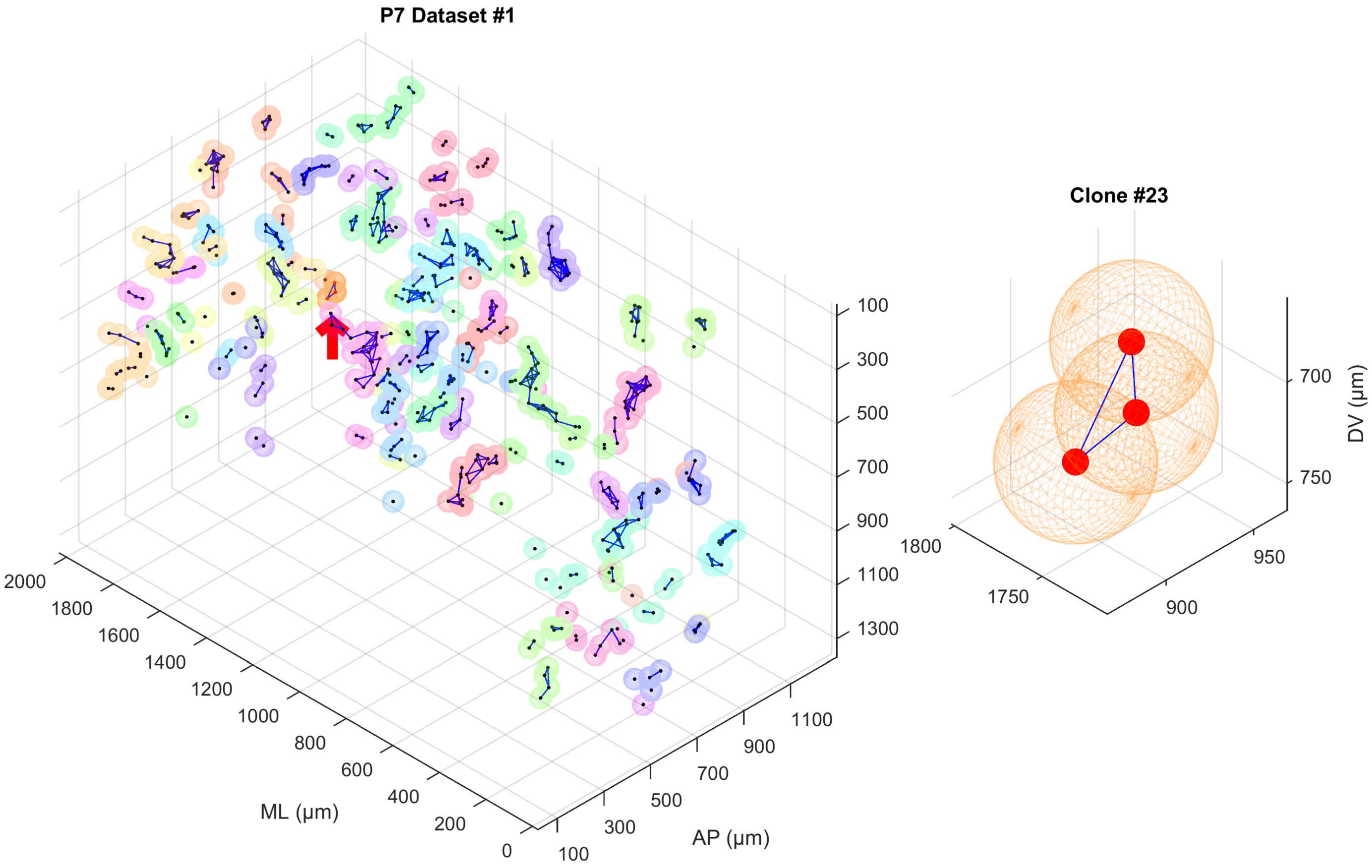
P7 Dataset #1
ML (µm)
AP (µm)
2000
1800
1600
1400
1200
1000
800
600
400
200
0
100
300
500
700
900
1100
100
300
500
700
900
1100
1300
Clone #23
DV (µm)
700
750
1800
1750
950
900

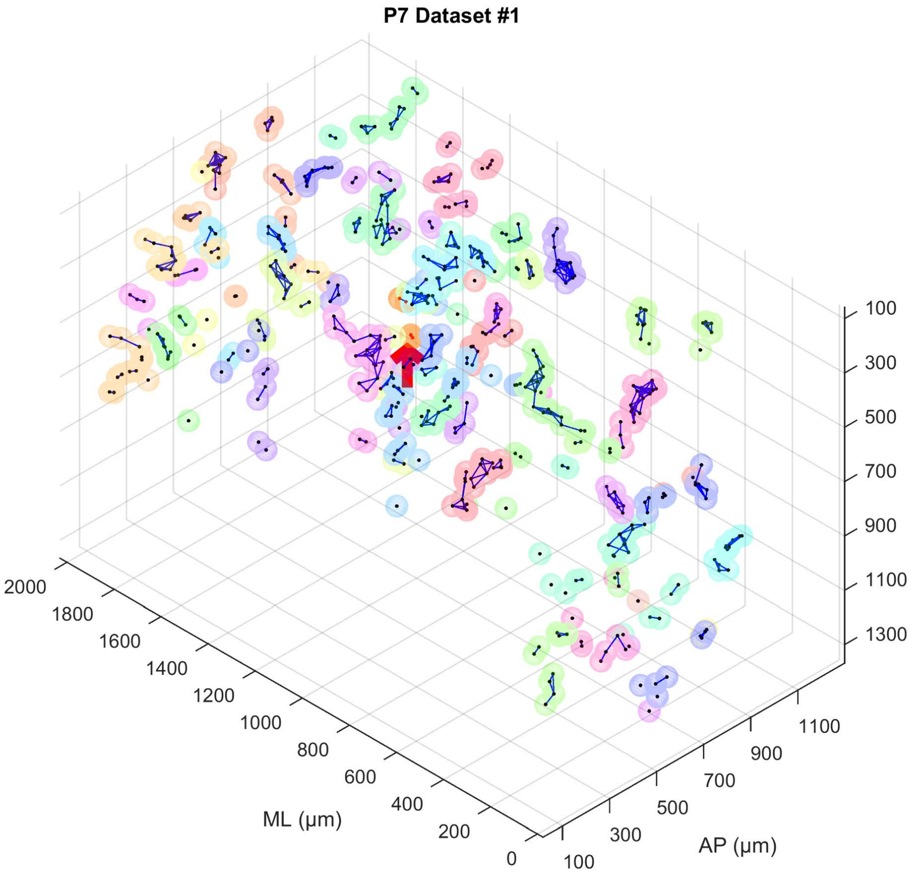

P7 Dataset #1
100
300
500
700
900
1100
1300
2000
1800
1600
1400
1200
1000
800
600
400
200
0
ML (µm)
100
300
500
700
900
1100
AP (µm)

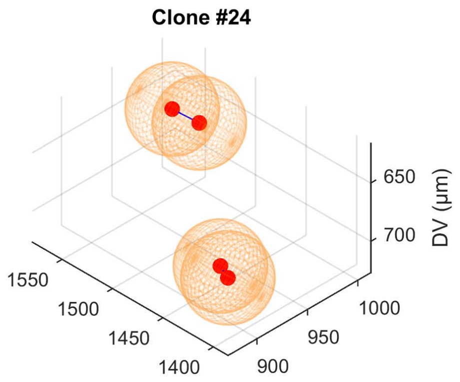

Clone #24
650
700
DV (µm)
1550
1500
1450
1400
900
950
1000

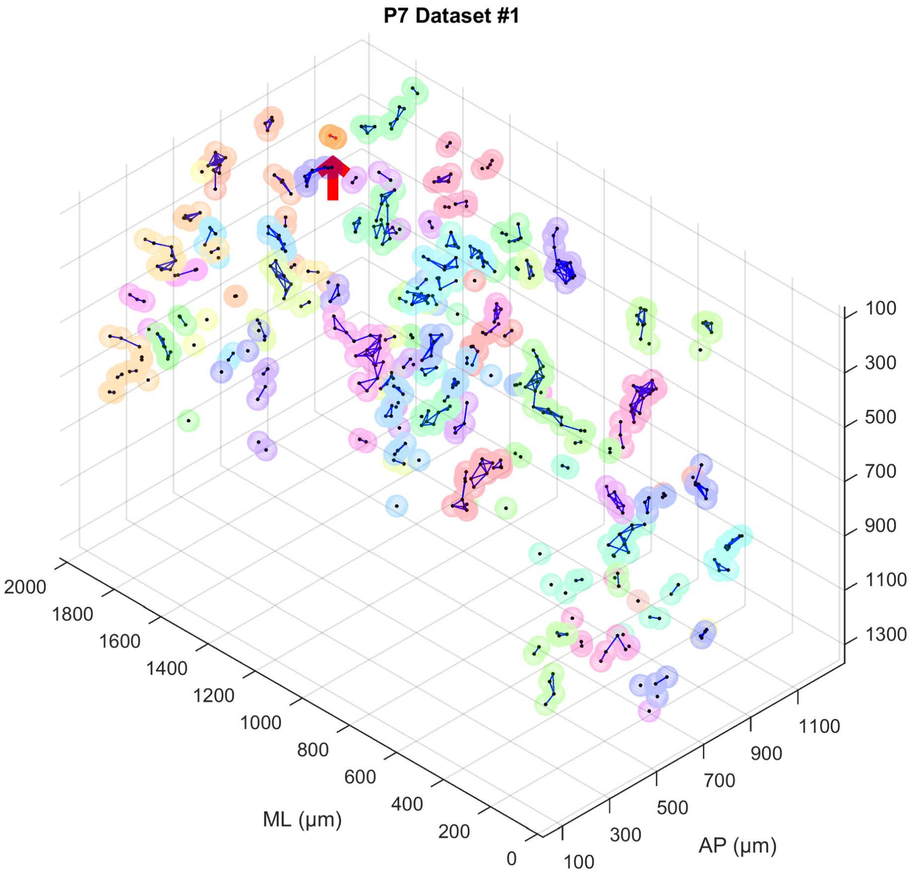

P7 Dataset #1
100
300
500
700
900
1100
1300
2000
1800
1600
1400
1200
1000
800
600
400
200
0
ML (µm)
100
300
500
700
900
1100
AP (µm)

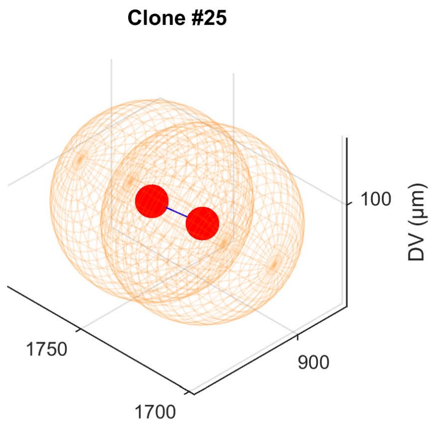

Clone #25
100
DV (µm)
1750
1700
900

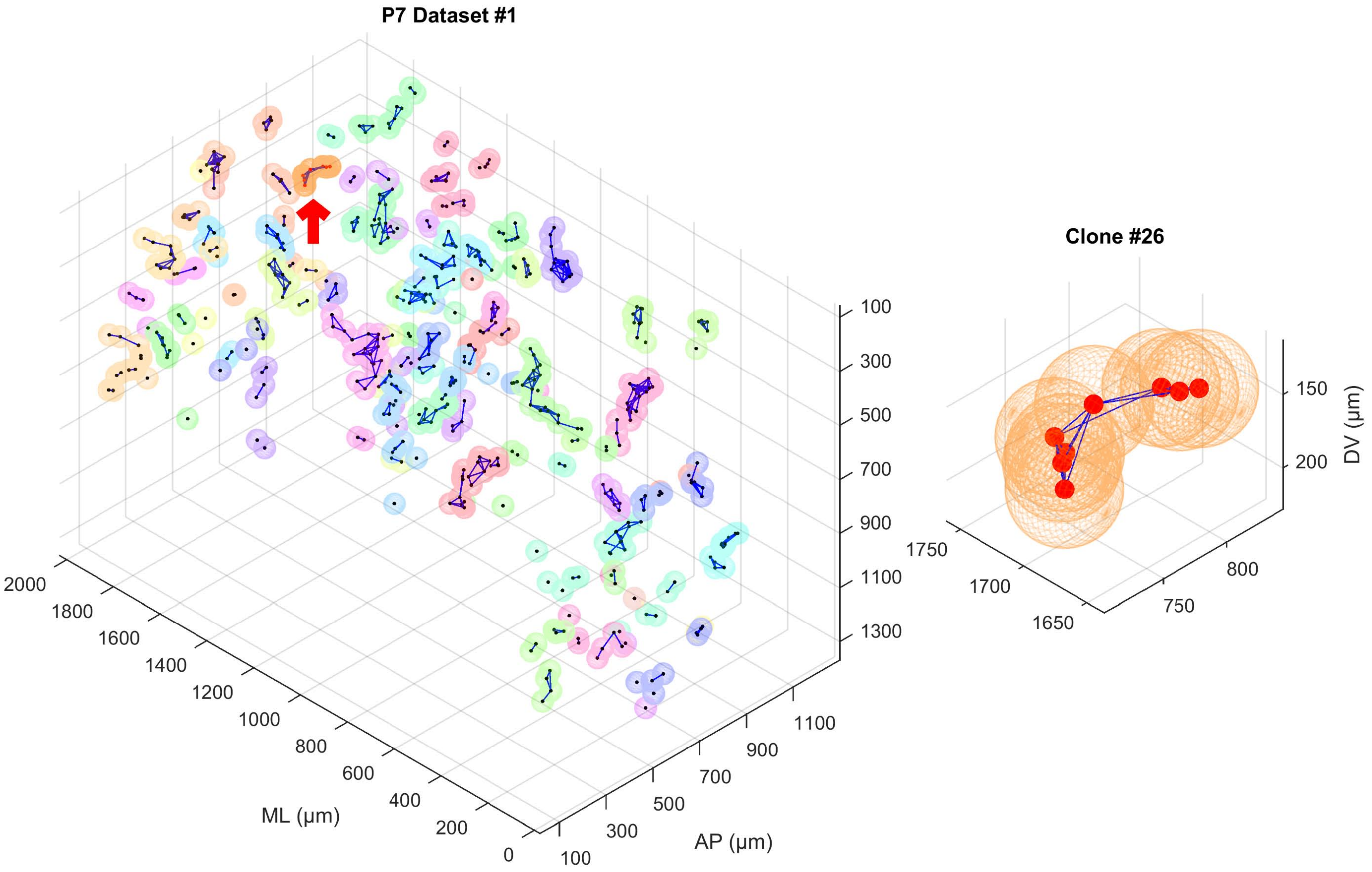

P7 Dataset #1
100
300
500
700
900
1100
1300
2000
1800
1600
1400
1200
1000
800
600
400
200
0
ML (μm)
100
300
500
700
900
1100
AP (μm)
Clone #26
150
200
DV (μm)
1750
1700
1650
750
800

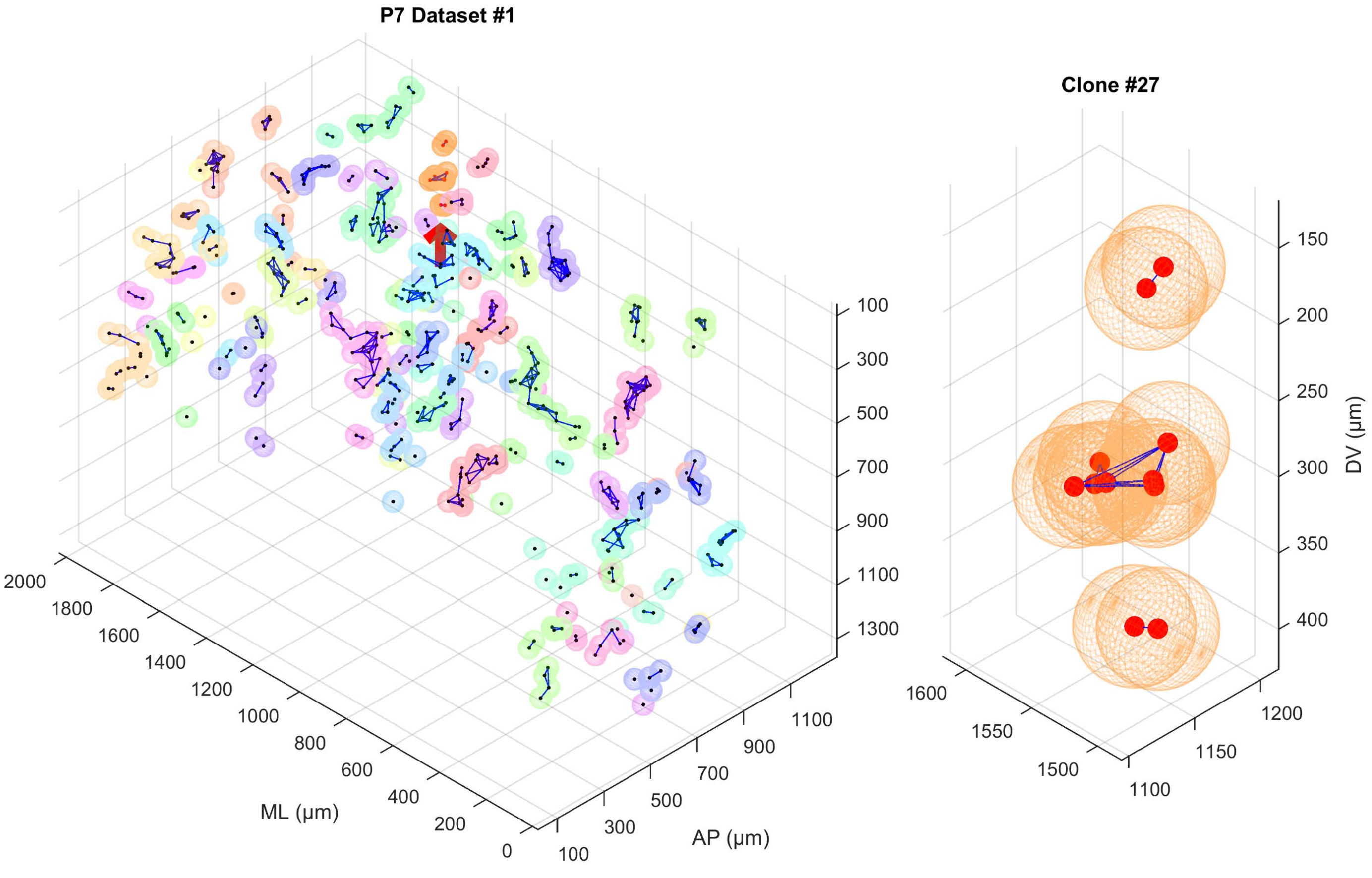

P7 Dataset #1
Clone #27
ML (µm)
AP (µm)
DV (µm)
2000
1800
1600
1400
1200
1000
800
600
400
200
0
100
300
500
700
900
1100
1300
150
200
250
300
350
400
1500
1550
1600
1100
1150
1200

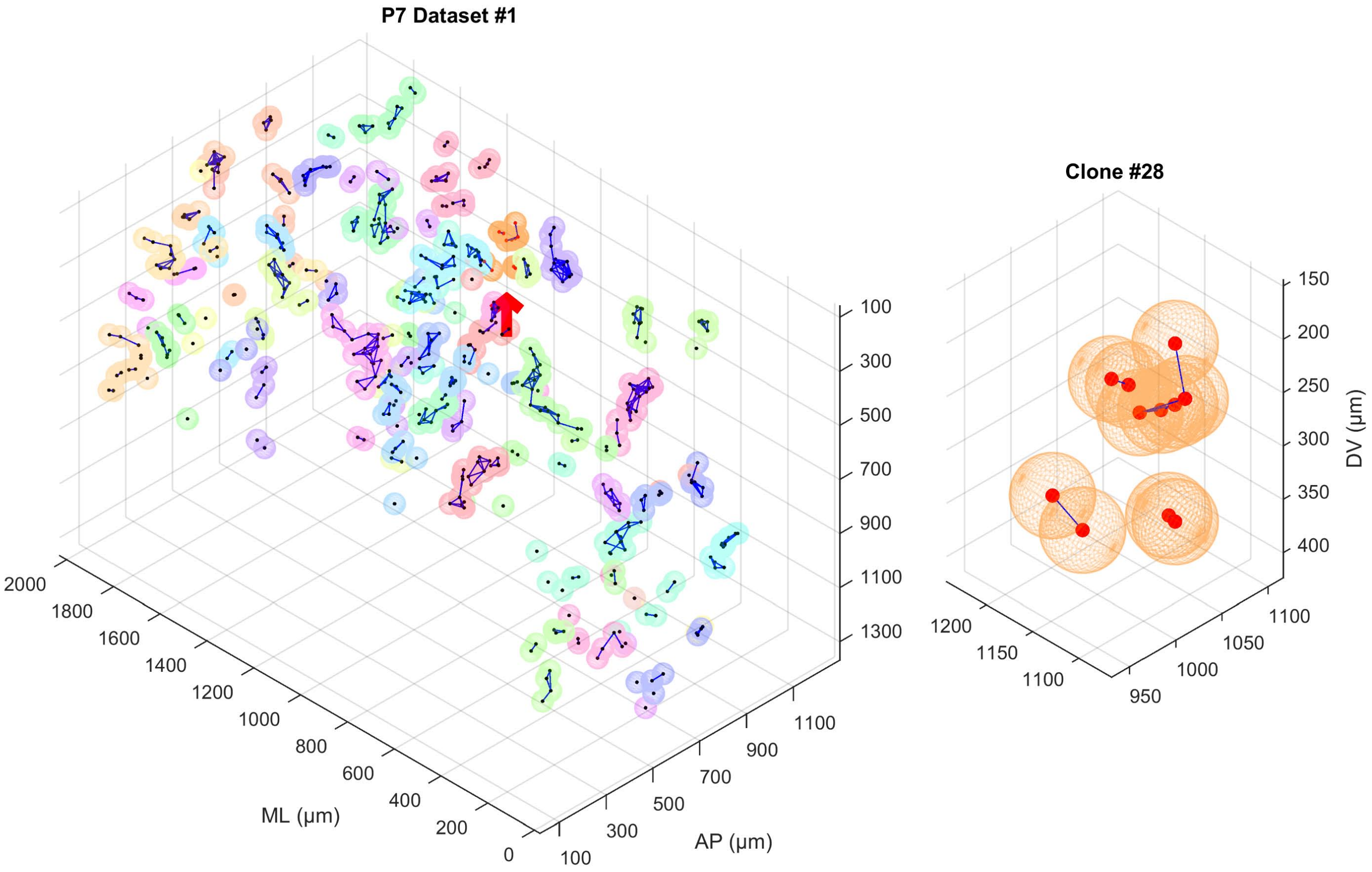
P7 Dataset #1
ML (µm)
AP (µm)
2000
1800
1600
1400
1200
1000
800
600
400
200
0
100
300
500
700
900
1100
1300
Clone #28
DV (µm)
150
200
250
300
350
400
1200
1150
1100
950
1000
1050
1100

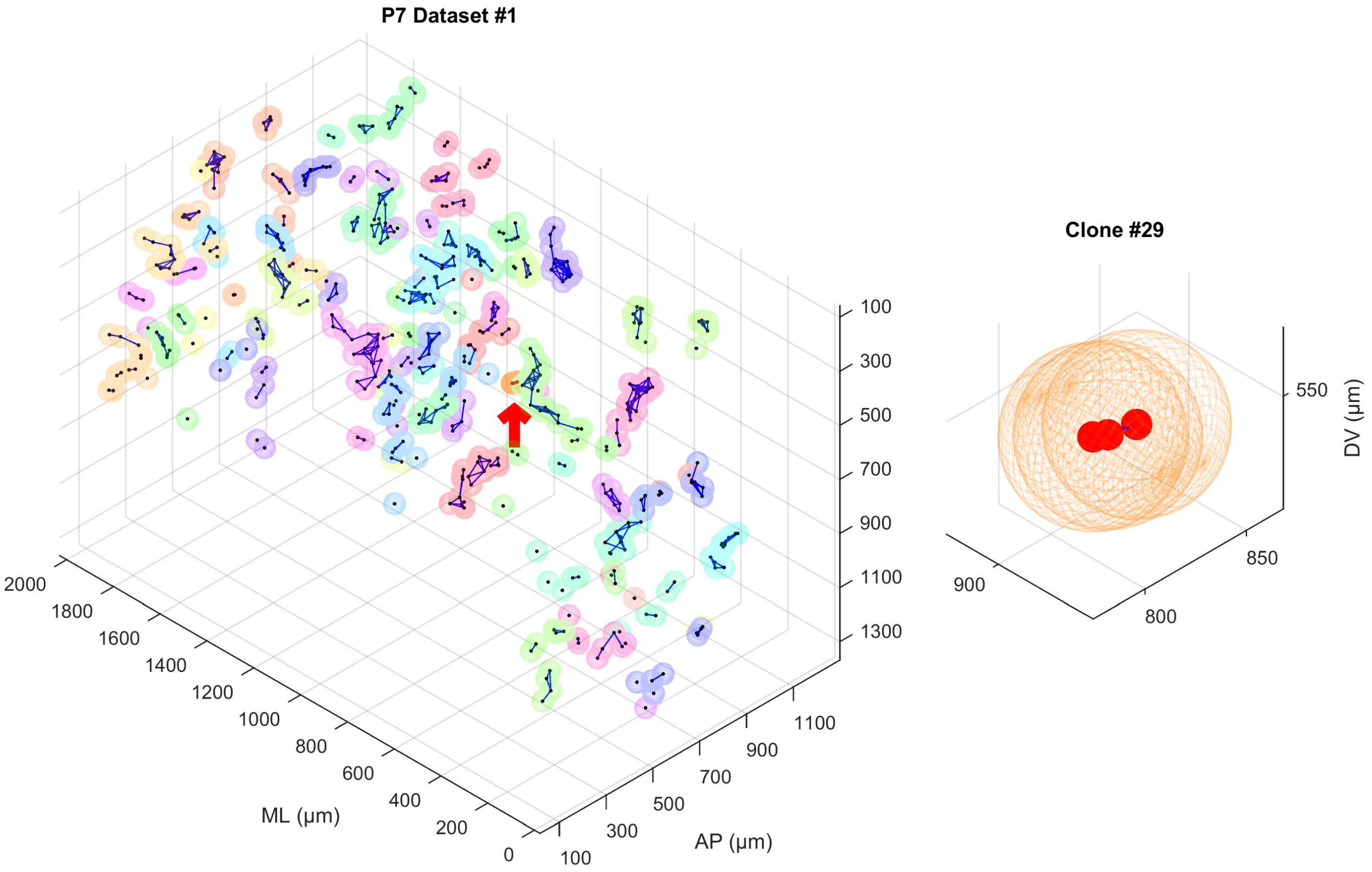

P7 Dataset #1
100
300
500
700
900
1100
1300
2000
1800
1600
1400
1200
1000
800
600
400
200
0
ML (μm)
100
300
500
700
900
1100
AP (μm)
Clone #29
550
DV (μm)
900
850
800

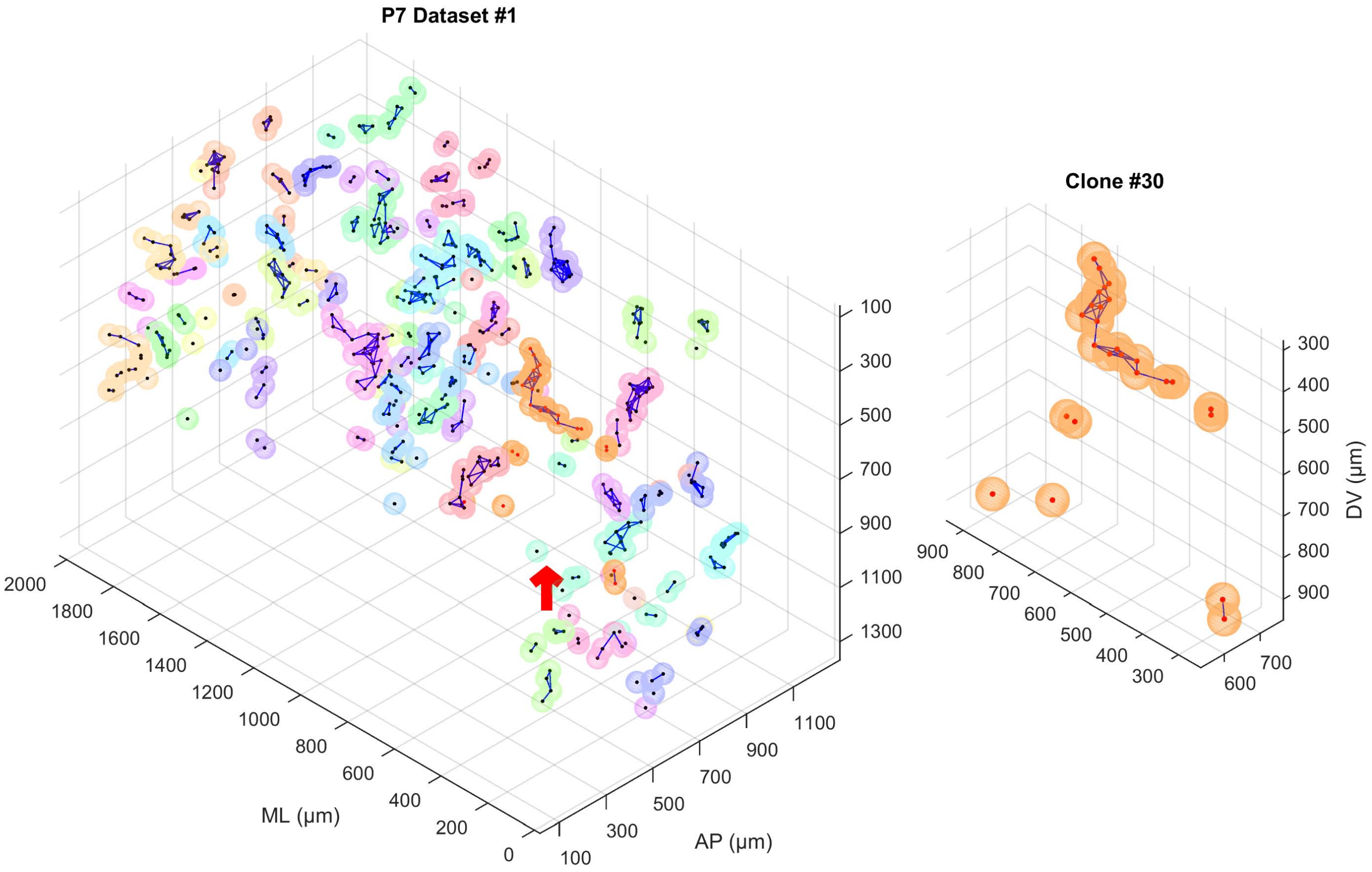

P7 Dataset #1
ML (µm)
AP (µm)
2000
1800
1600
1400
1200
1000
800
600
400
200
0
100
300
500
700
900
1100
1300
Clone #30
DV (µm)
900
800
700
600
500
400
300

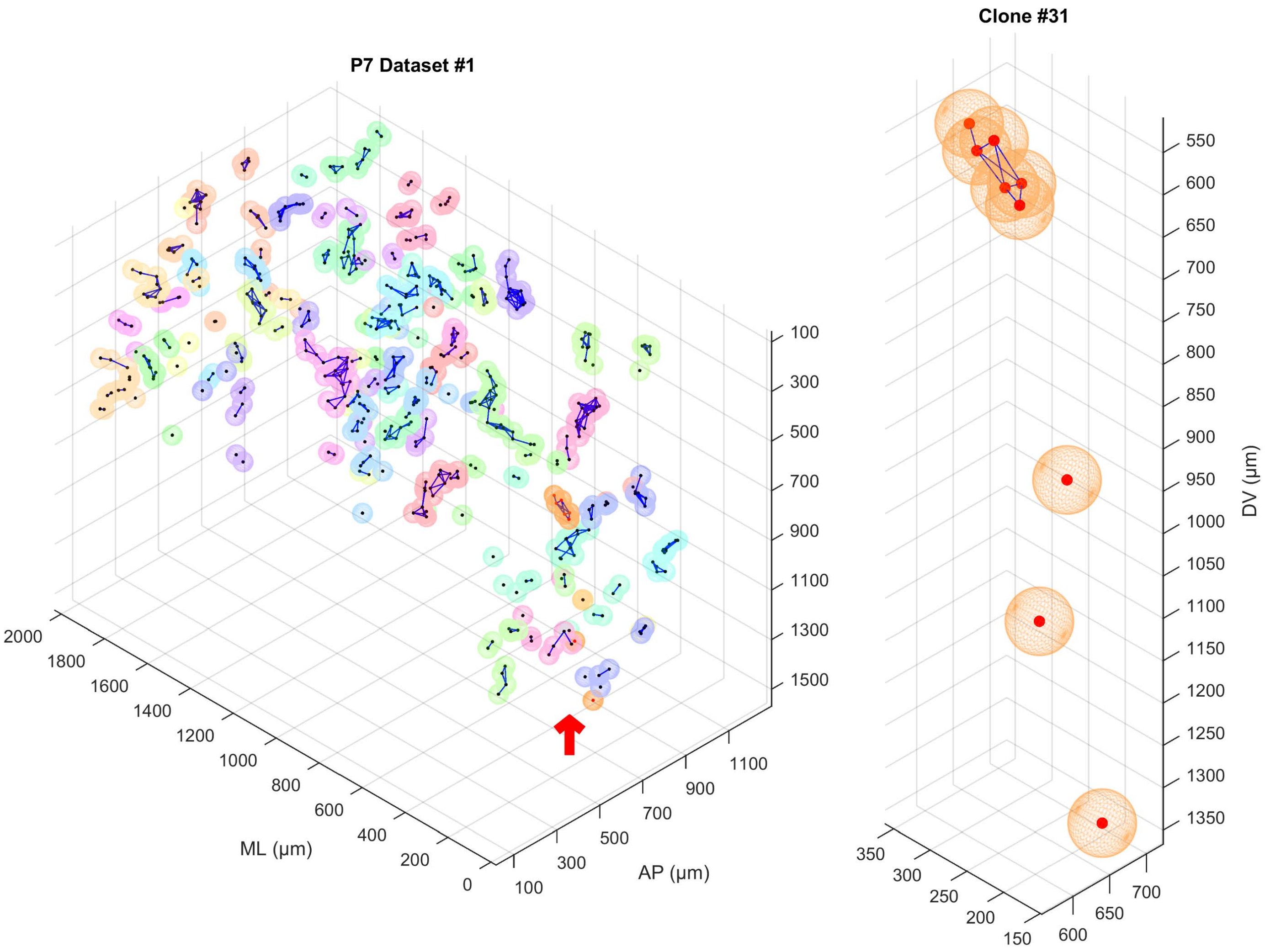

P7 Dataset #1
100
300
500
700
900
1100
1300
1500
2000
1800
1600
1400
1200
1000
800
600
400
200
0
ML (µm)
100
300
500
700
900
1100
AP (µm)
Clone #31
550
600
650
700
750
800
850
900
950
1000
1050
1100
1150
1200
1250
1300
1350
DV (µm)
350
300
250
200
150
600
650
700

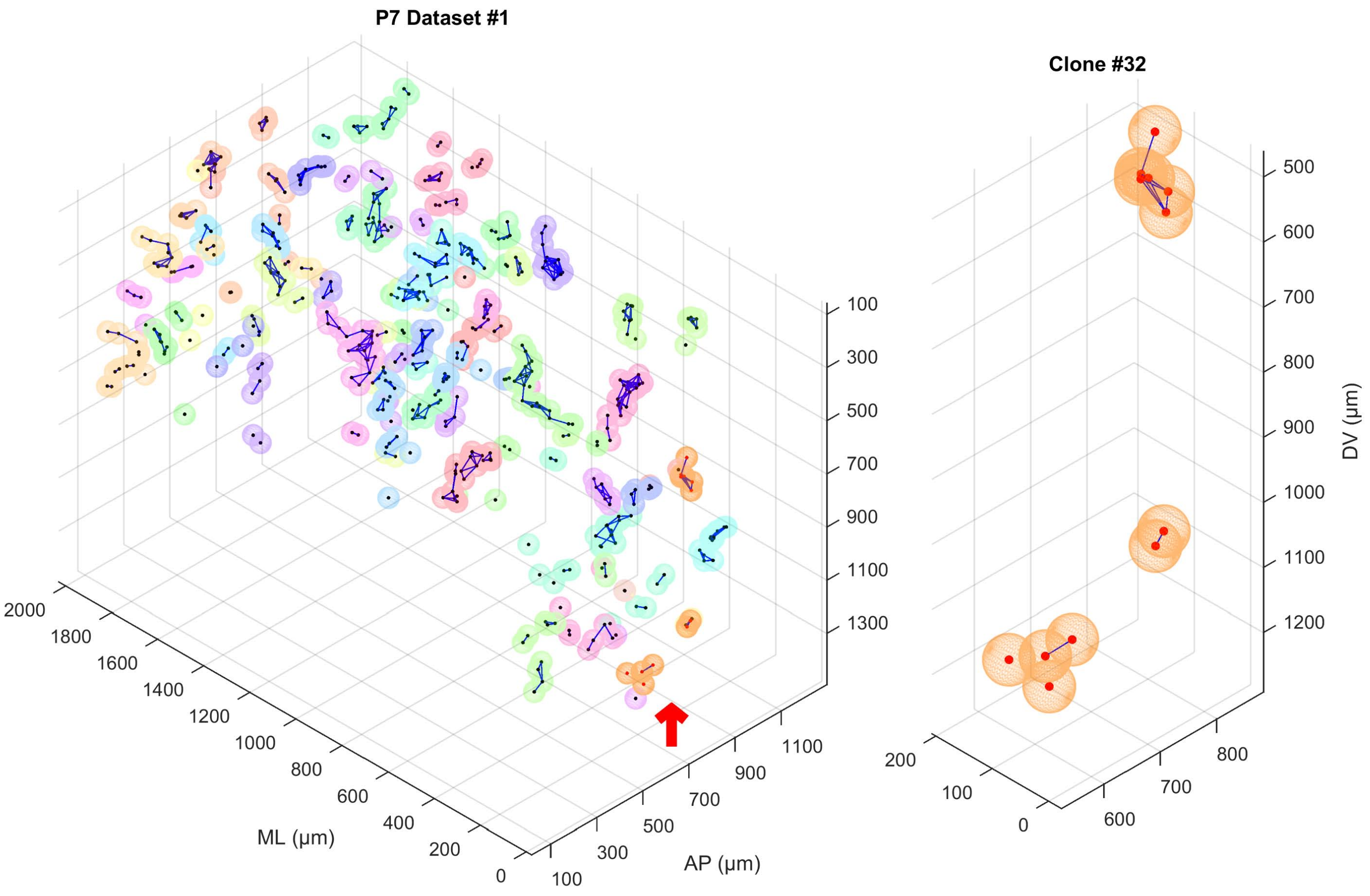

P7 Dataset #1
Clone #32
ML (µm)
AP (µm)
DV (µm)
2000
1800
1600
1400
1200
1000
800
600
400
200
0
100
300
500
700
900
1100
1300

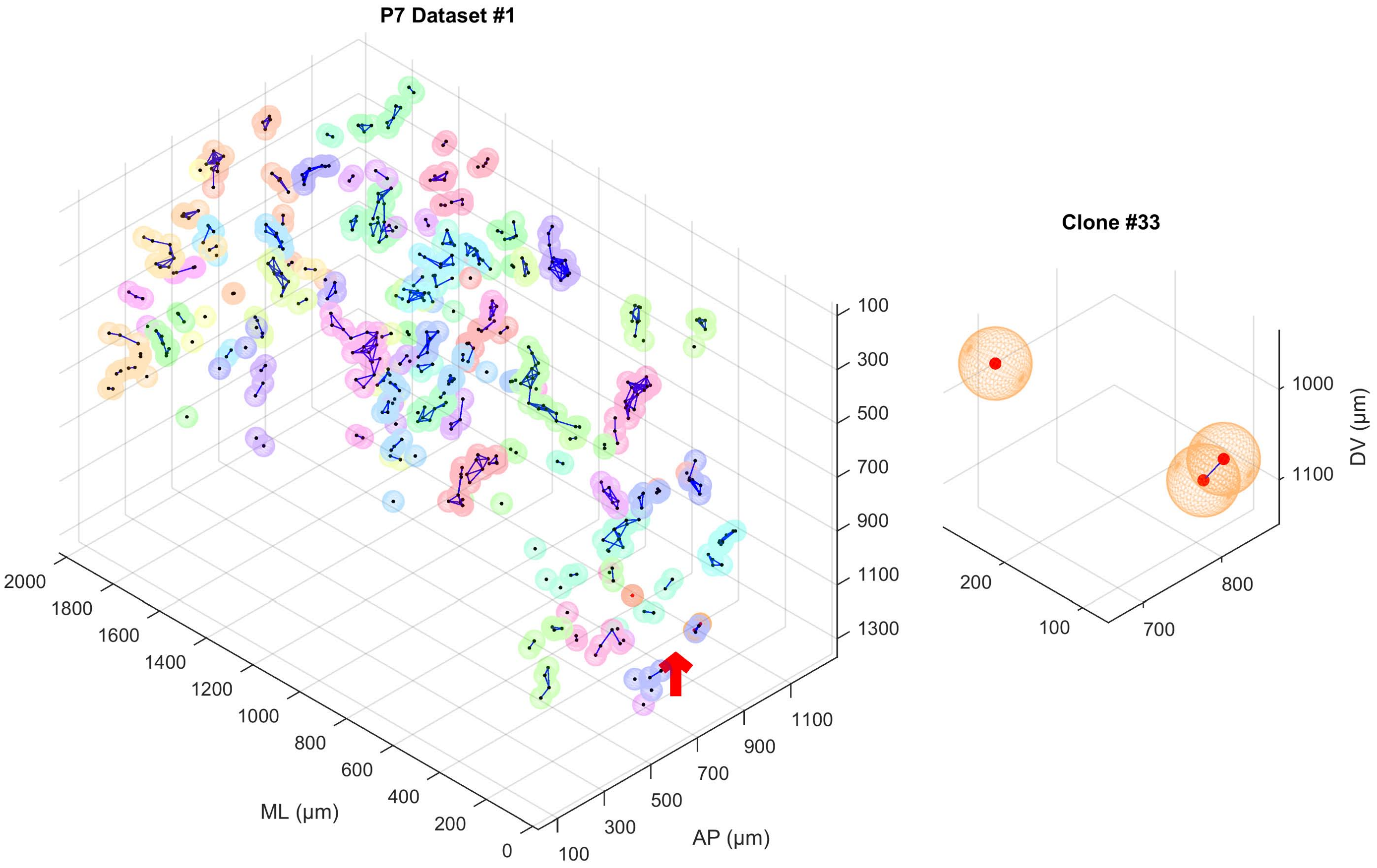

P7 Dataset #1
100
300
500
700
900
1100
1300
2000
1800
1600
1400
1200
1000
800
600
400
200
0
ML (µm)
100
300
500
700
900
1100
AP (µm)
Clone #33
1000
1100
DV (µm)
200
100
800
700

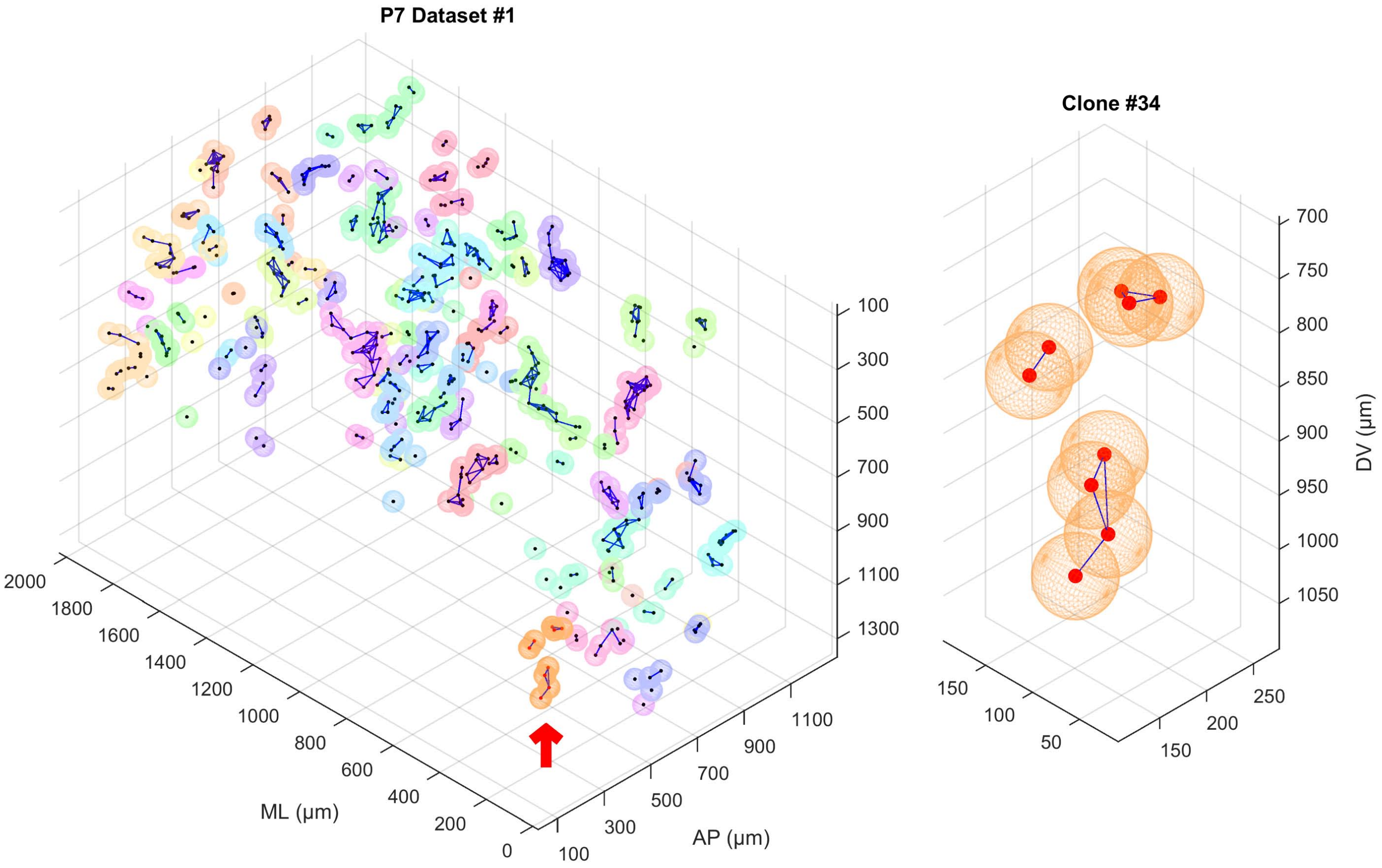

P7 Dataset #1
ML (µm)
AP (µm)
2000
1800
1600
1400
1200
1000
800
600
400
200
0
100
300
500
700
900
1100
1300
Clone #34
DV (µm)
700
750
800
850
900
950
1000
1050
150
100
50
250
200

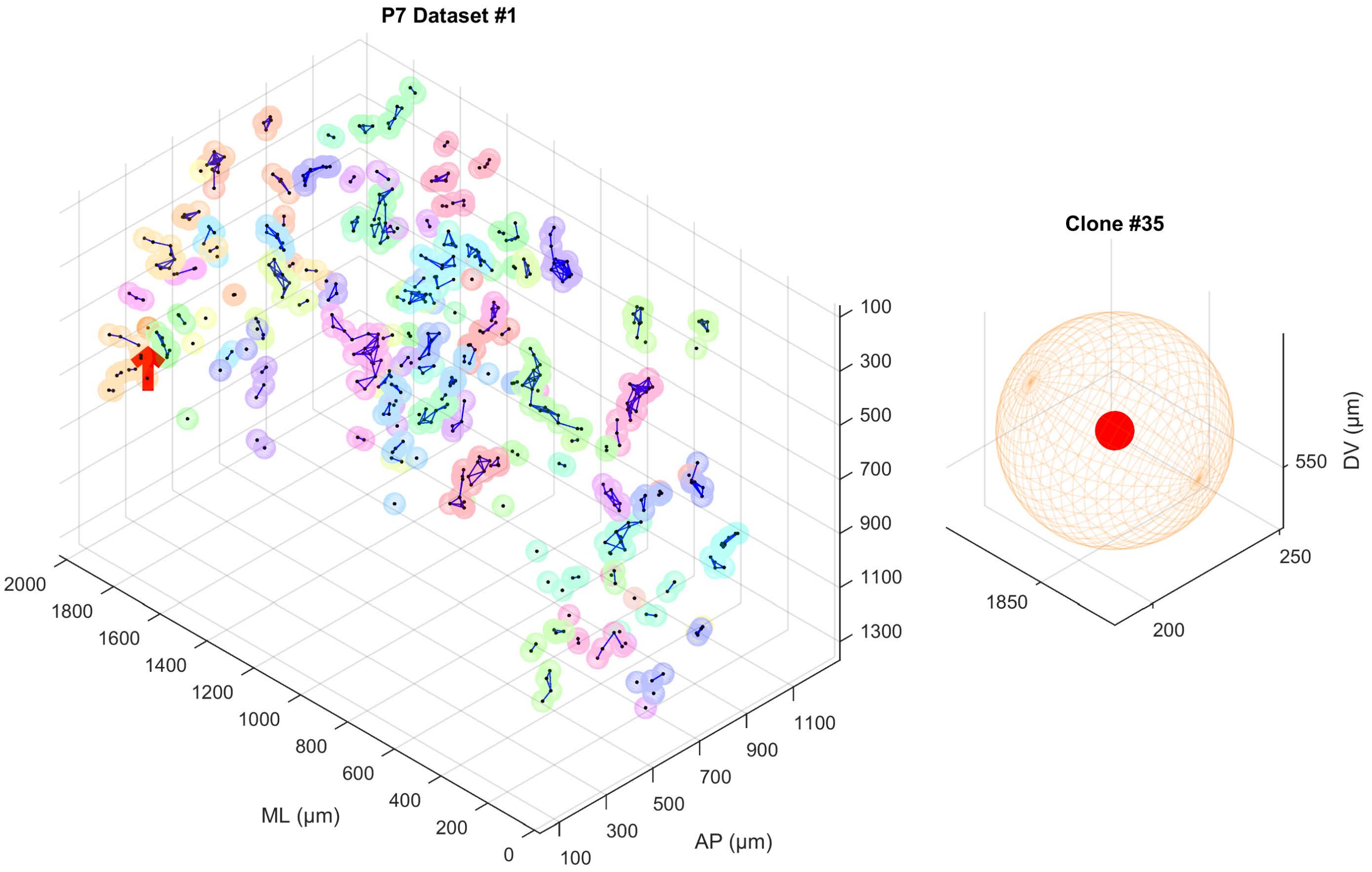

P7 Dataset #1
ML (µm)
AP (µm)
2000
1800
1600
1400
1200
1000
800
600
400
200
0
100
300
500
700
900
1100
100
300
500
700
900
1100
1300
Clone #35
DV (µm)
550
1850
200
250

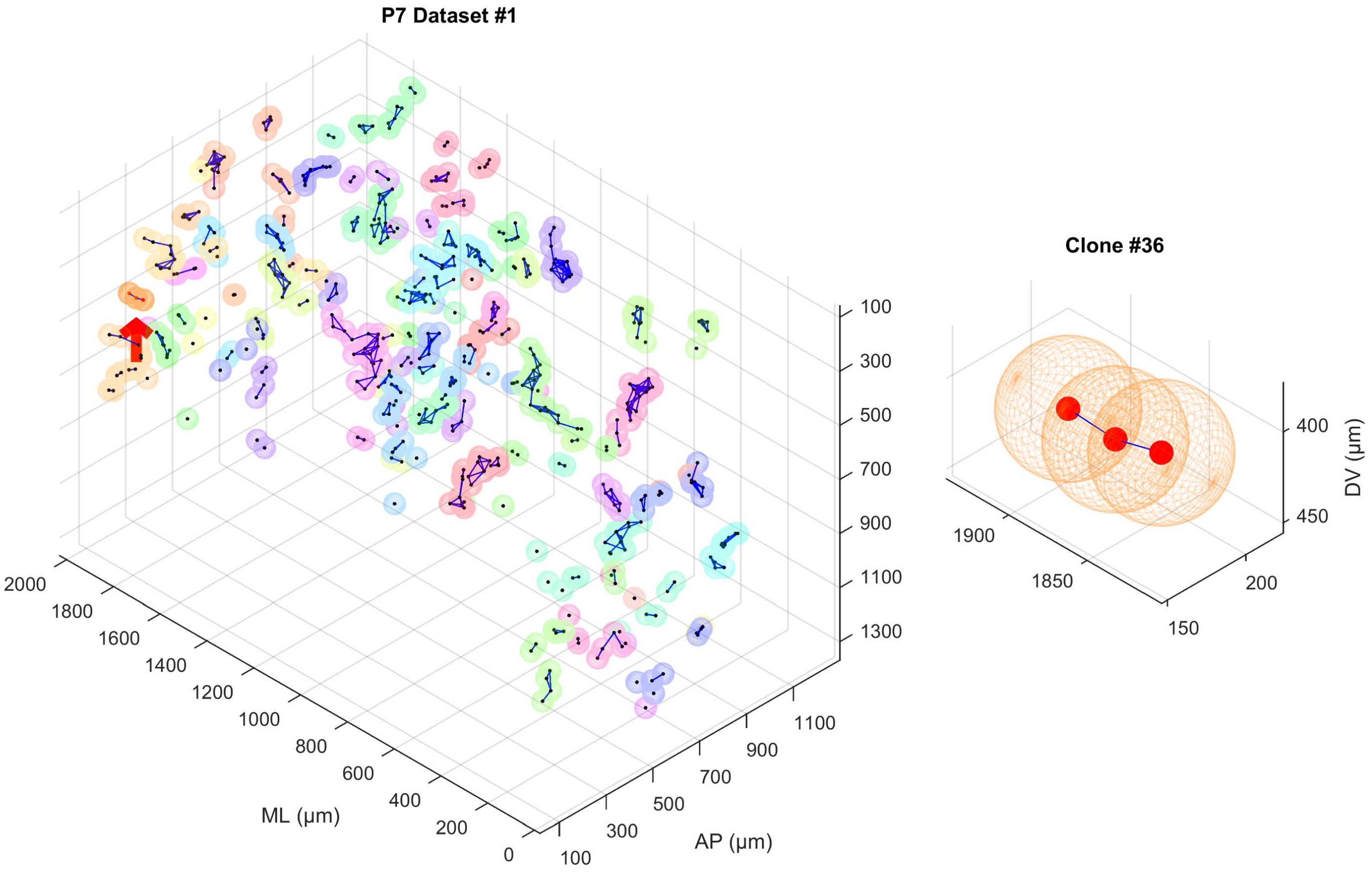

P7 Dataset #1
ML (µm)
2000
1800
1600
1400
1200
1000
800
600
400
200
0
AP (µm)
100
300
500
700
900
1100
100
300
500
700
900
1100
1300
Clone #36
1900
1850
150
200
DV (µm)
400
450

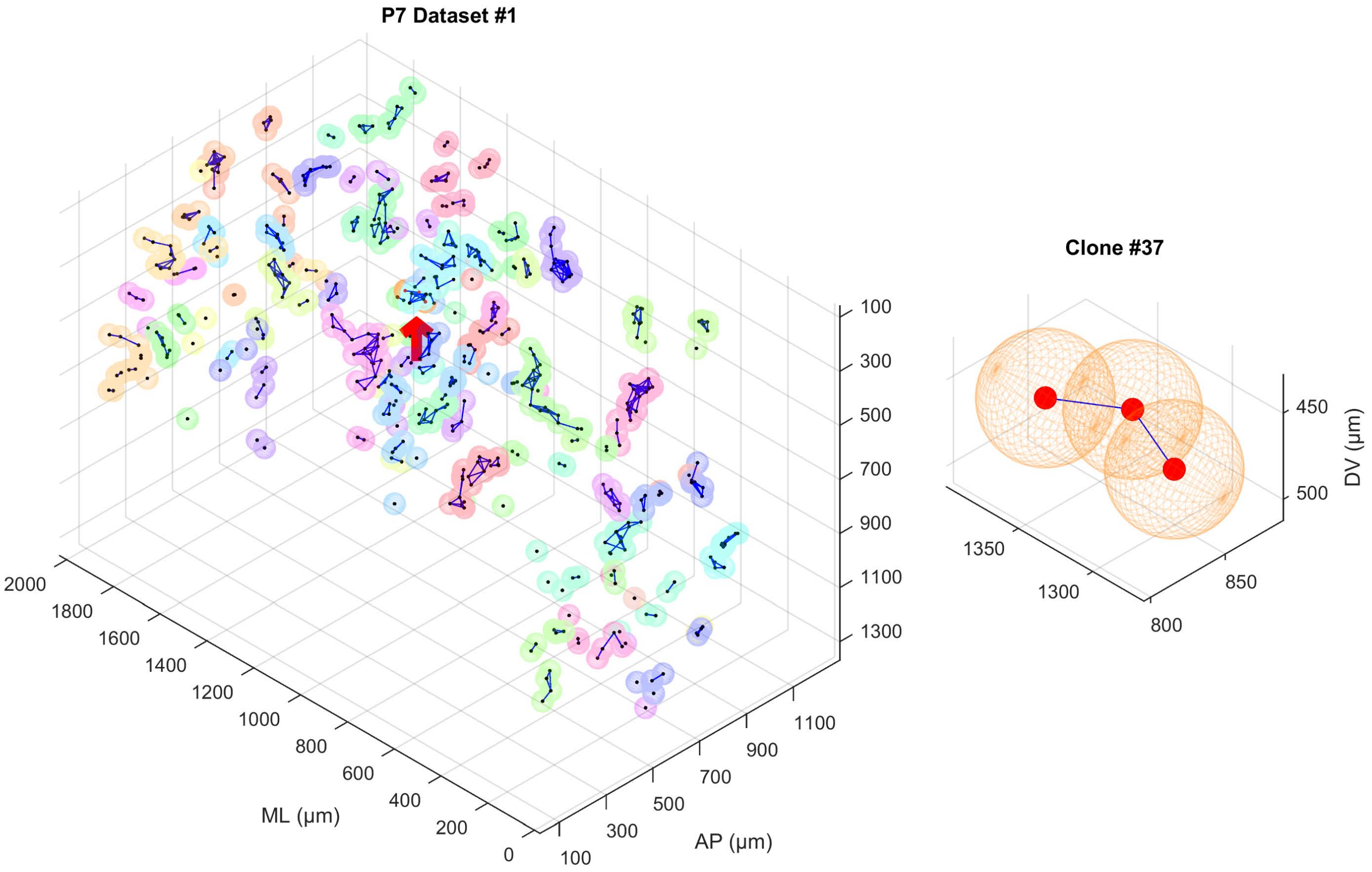
P7 Dataset #1
ML (µm)
2000
1800
1600
1400
1200
1000
800
600
400
200
0
AP (µm)
100
300
500
700
900
1100
100
300
500
700
900
1100
1300
Clone #37
1350
1300
800
850
450
500
DV (µm)

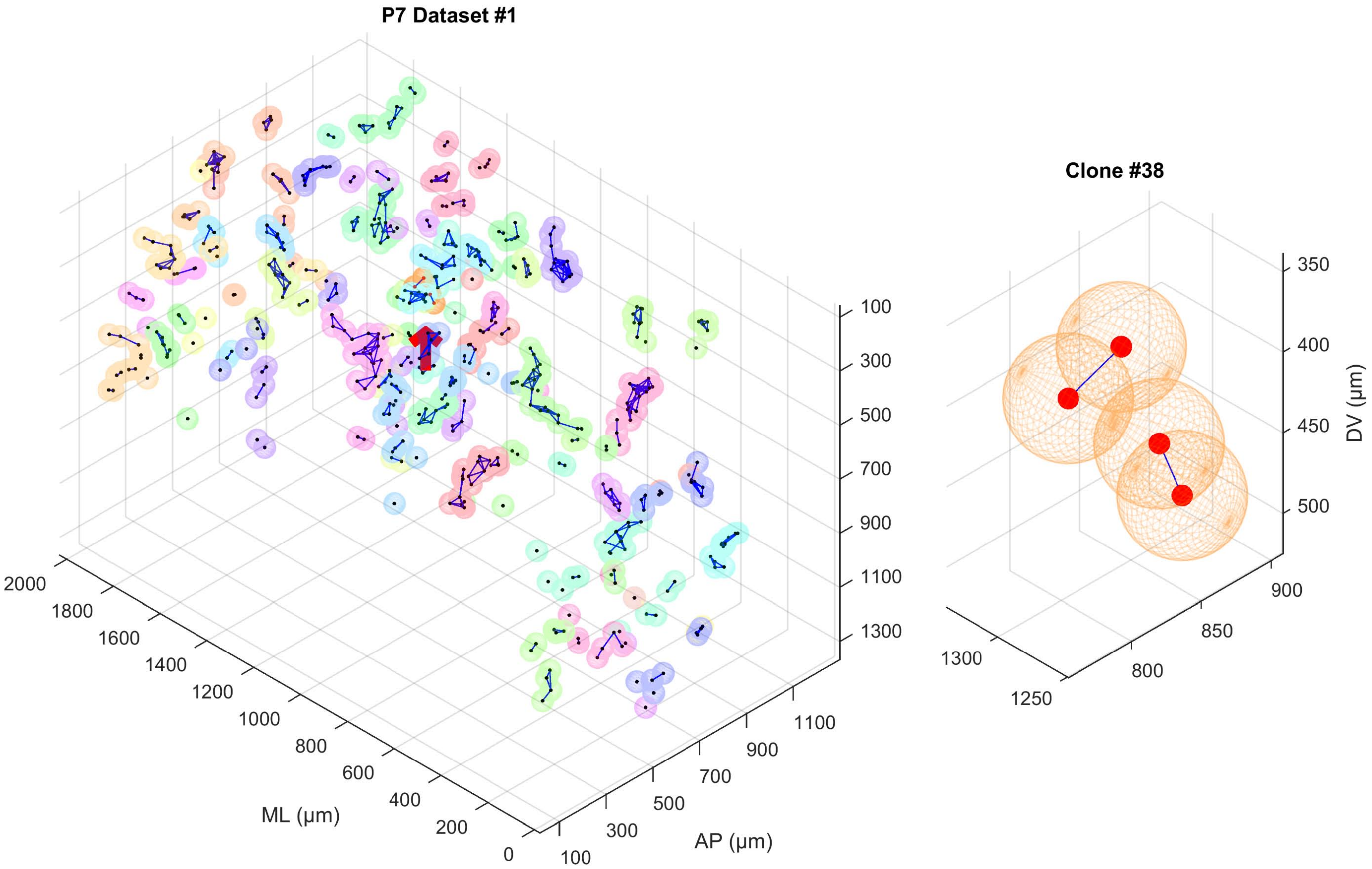
P7 Dataset #1
ML (μm)
2000
1800
1600
1400
1200
1000
800
600
400
200
0
AP (μm)
100
300
500
700
900
1100
100
300
500
700
900
1100
1300
Clone #38
DV (μm)
350
400
450
500
900
850
800
1300
1250

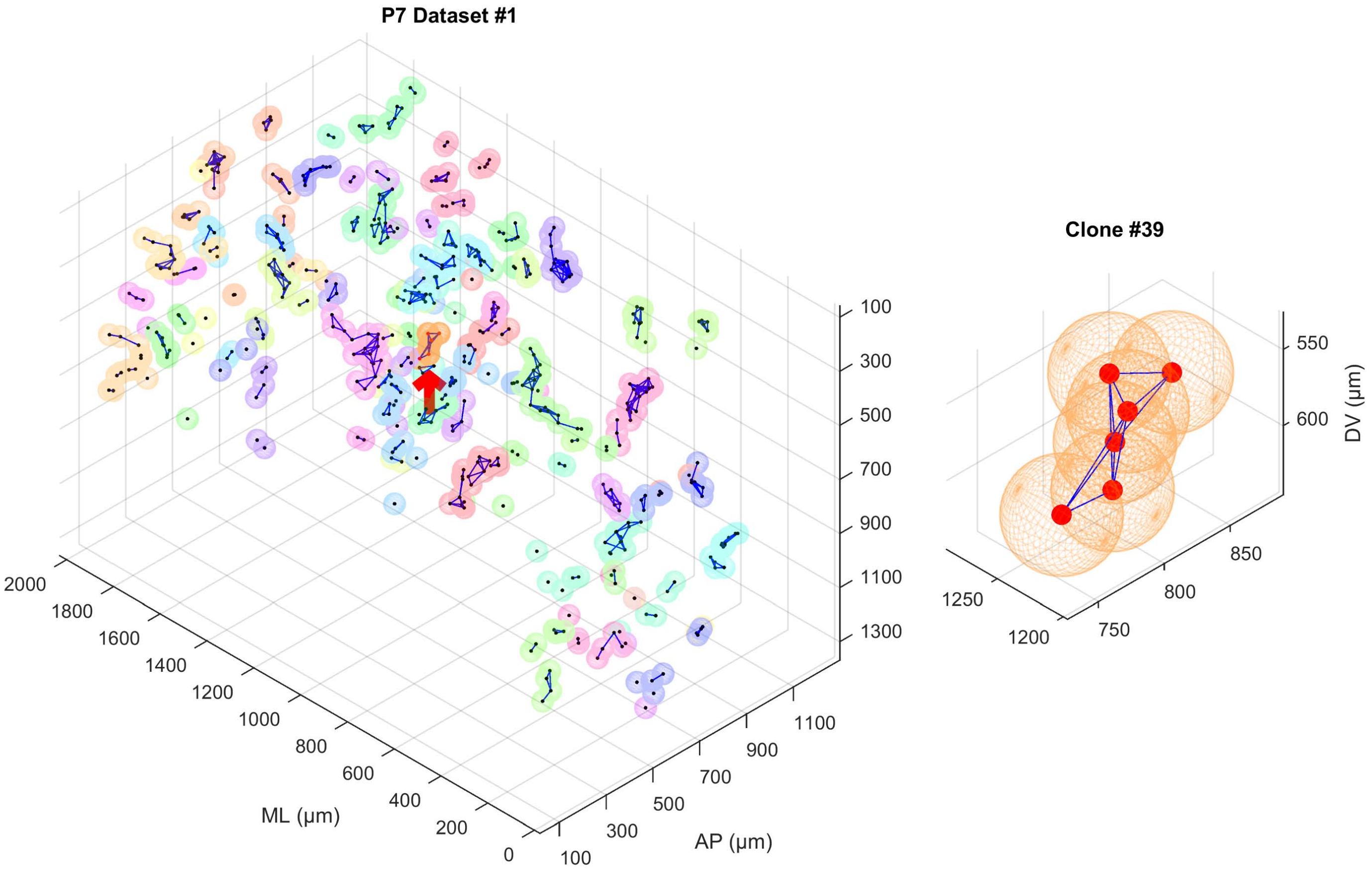

P7 Dataset #1
Clone #39
ML (µm)
AP (µm)
DV (µm)
2000
1800
1600
1400
1200
1000
800
600
400
200
0
100
300
500
700
900
1100
1300
1250
1200
750
800
850
550
600

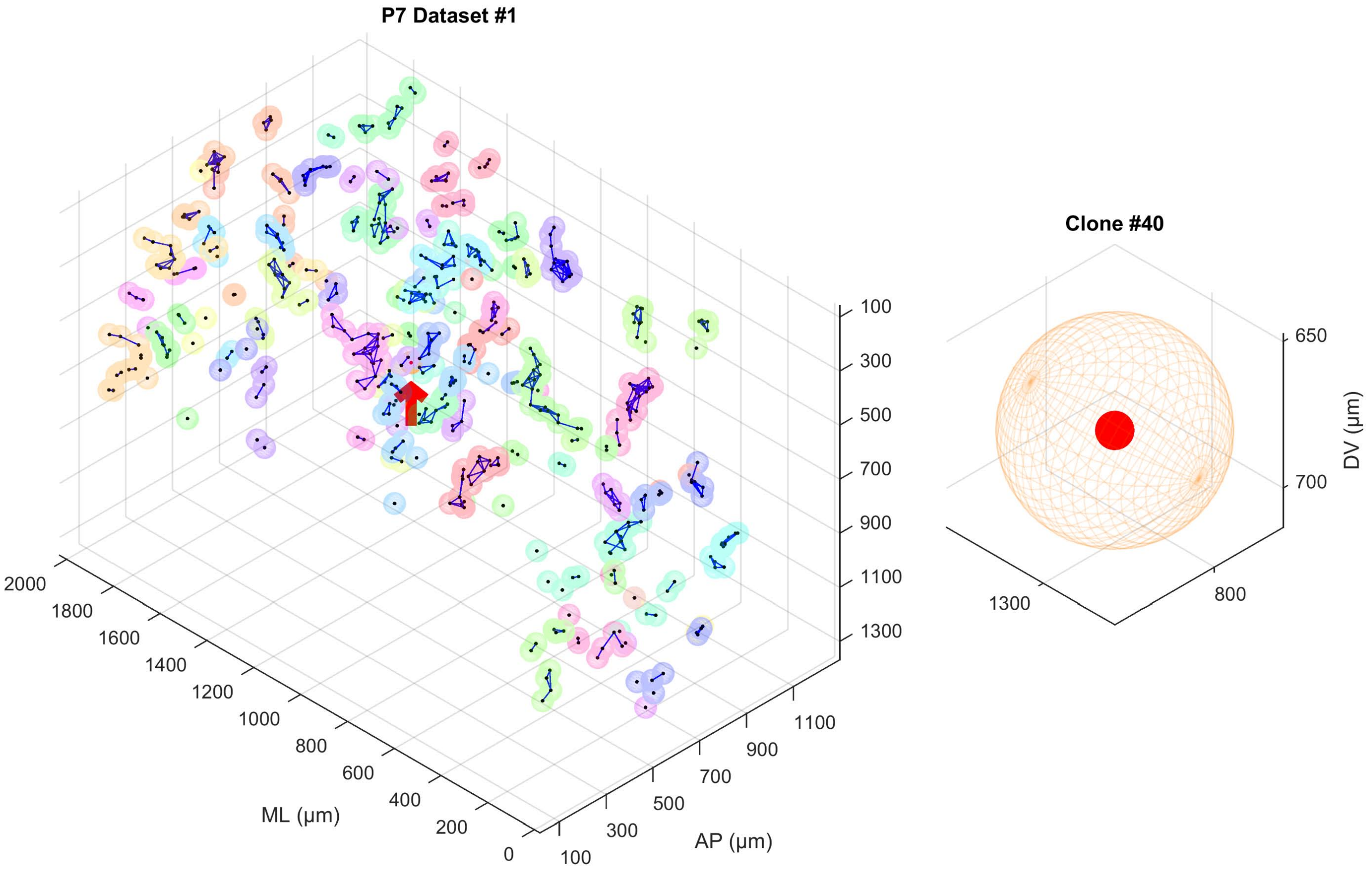
P7 Dataset #1
100
300
500
700
900
1100
1300
2000
1800
1600
1400
1200
1000
800
600
400
200
0
ML (µm)
100
300
500
700
900
1100
AP (µm)
Clone #40
650
700
DV (µm)
1300
800

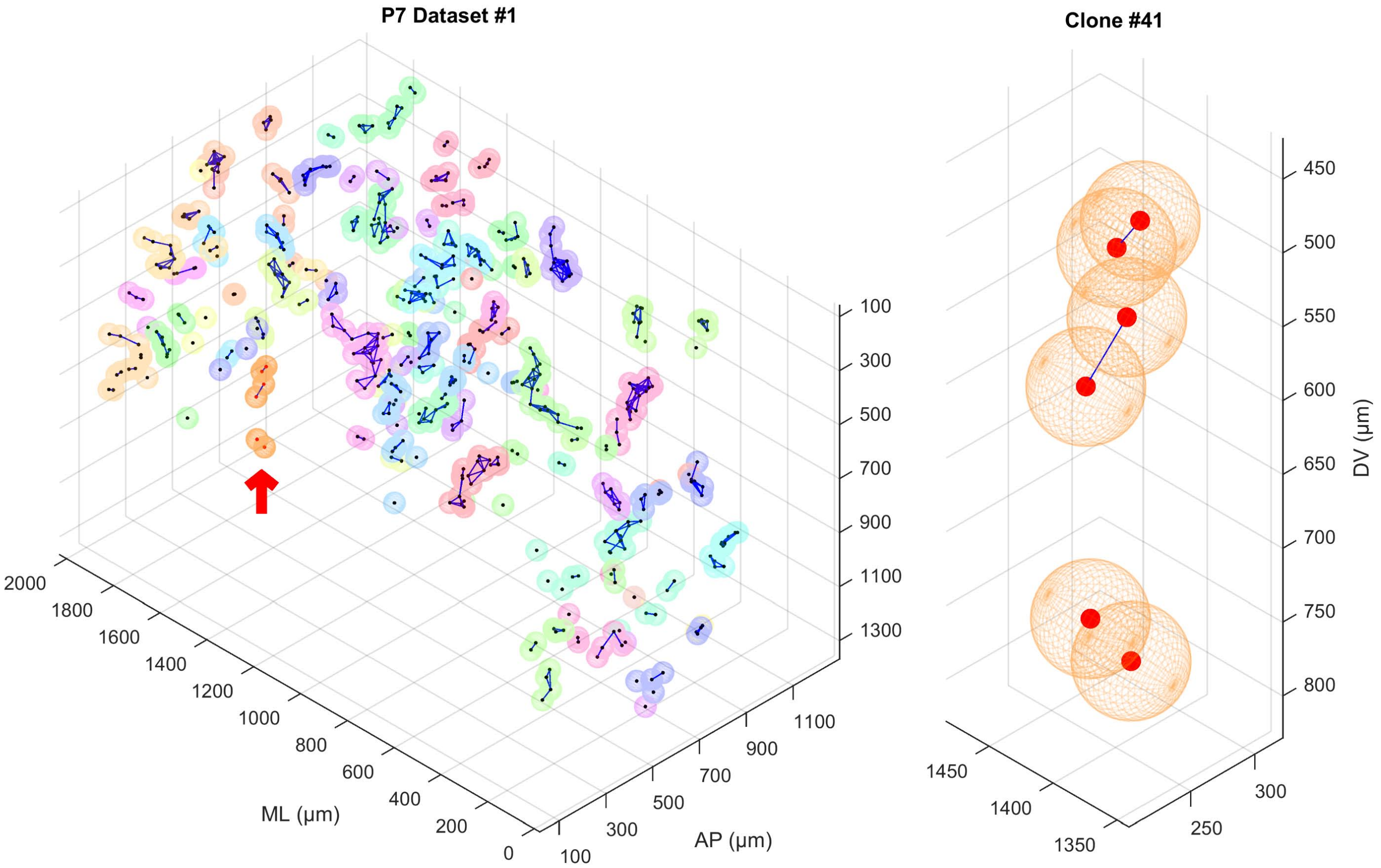

P7 Dataset #1
Clone #41
ML (μm)
AP (μm)
DV (μm)
2000
1800
1600
1400
1200
1000
800
600
400
200
0
100
300
500
700
900
1100
1300
450
500
550
600
650
700
750
800
1450
1400
1350
250
300

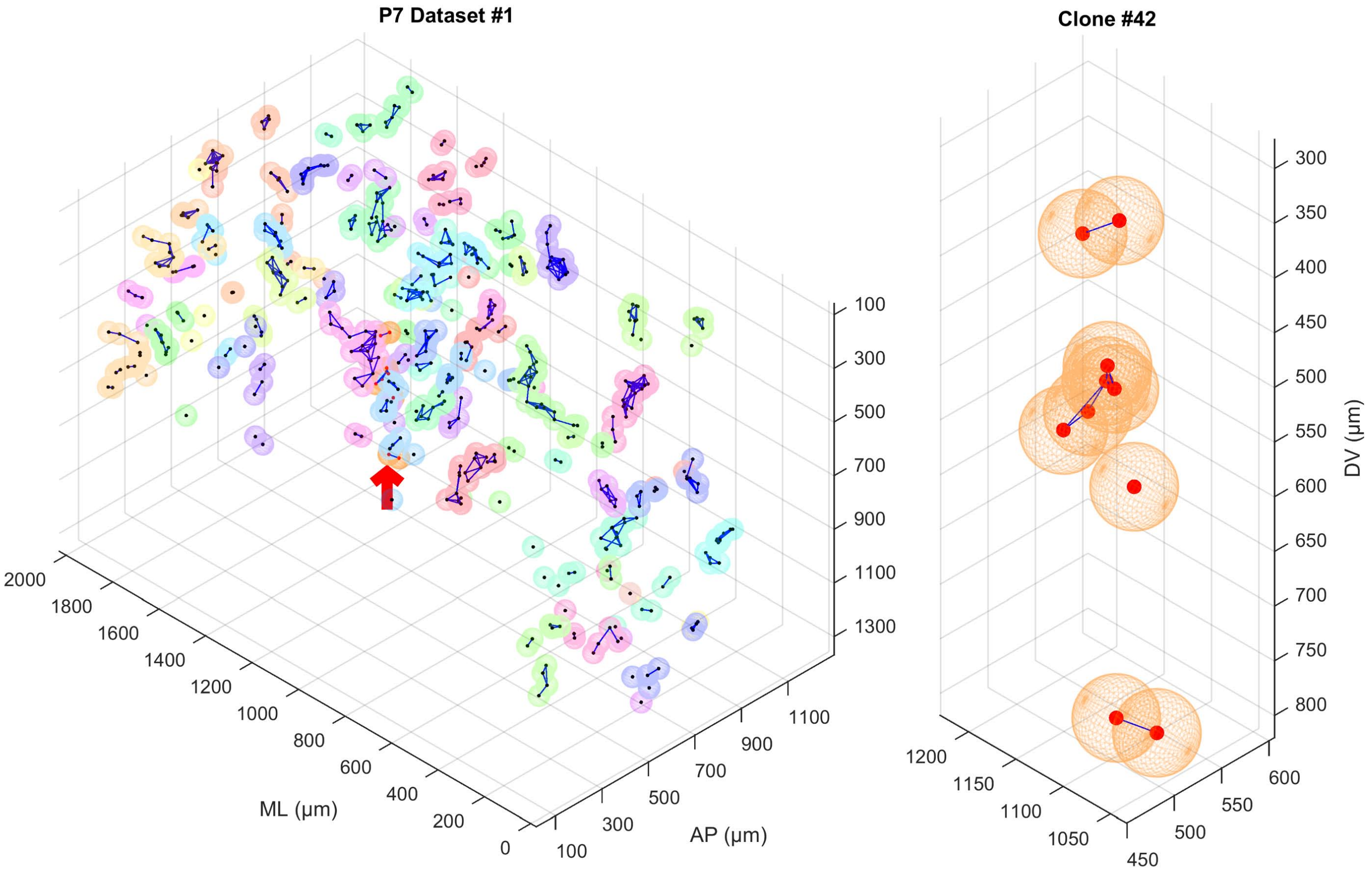

P7 Dataset #1
Clone #42
ML (µm)
AP (µm)
DV (µm)
2000
1800
1600
1400
1200
1000
800
600
400
200
0
100
300
500
700
900
1100
1300
300
350
400
450
500
550
600
650
700
750
800
1200
1150
1100
1050
450
500
550
600

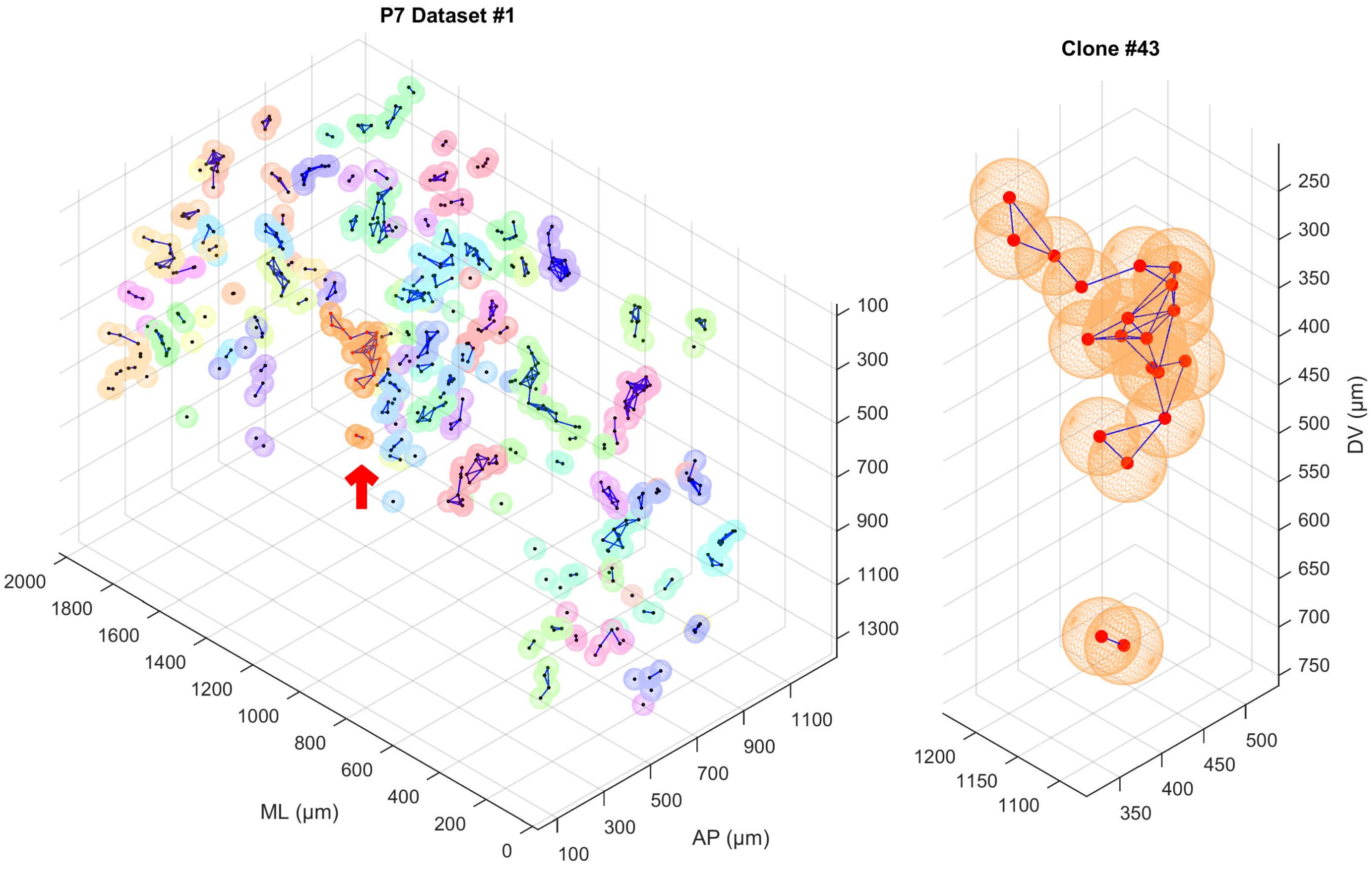

P7 Dataset #1
ML (µm)
2000
1800
1600
1400
1200
1000
800
600
400
200
0
AP (µm)
100
300
500
700
900
1100
100
300
500
700
900
1100
1300
Clone #43
DV (µm)
250
300
350
400
450
500
550
600
650
700
750
1200
1150
1100
350
400
450
500

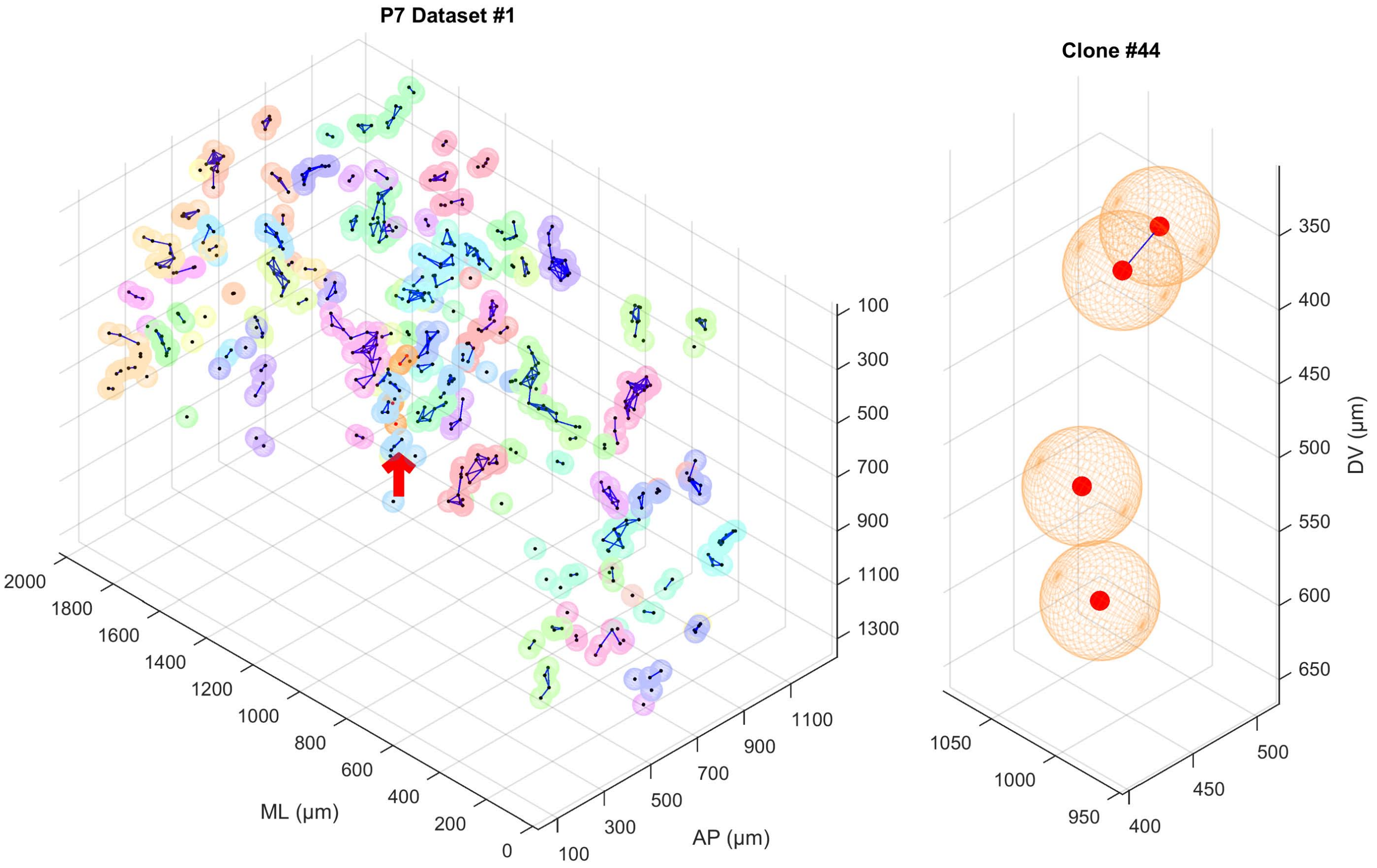

P7 Dataset #1
Clone #44
100
300
500
700
900
1100
1300
2000
1800
1600
1400
1200
1000
800
600
400
200
0
ML (µm)
100
300
500
700
900
1100
AP (µm)
350
400
450
500
550
600
650
DV (µm)
1050
1000
950
400
450
500

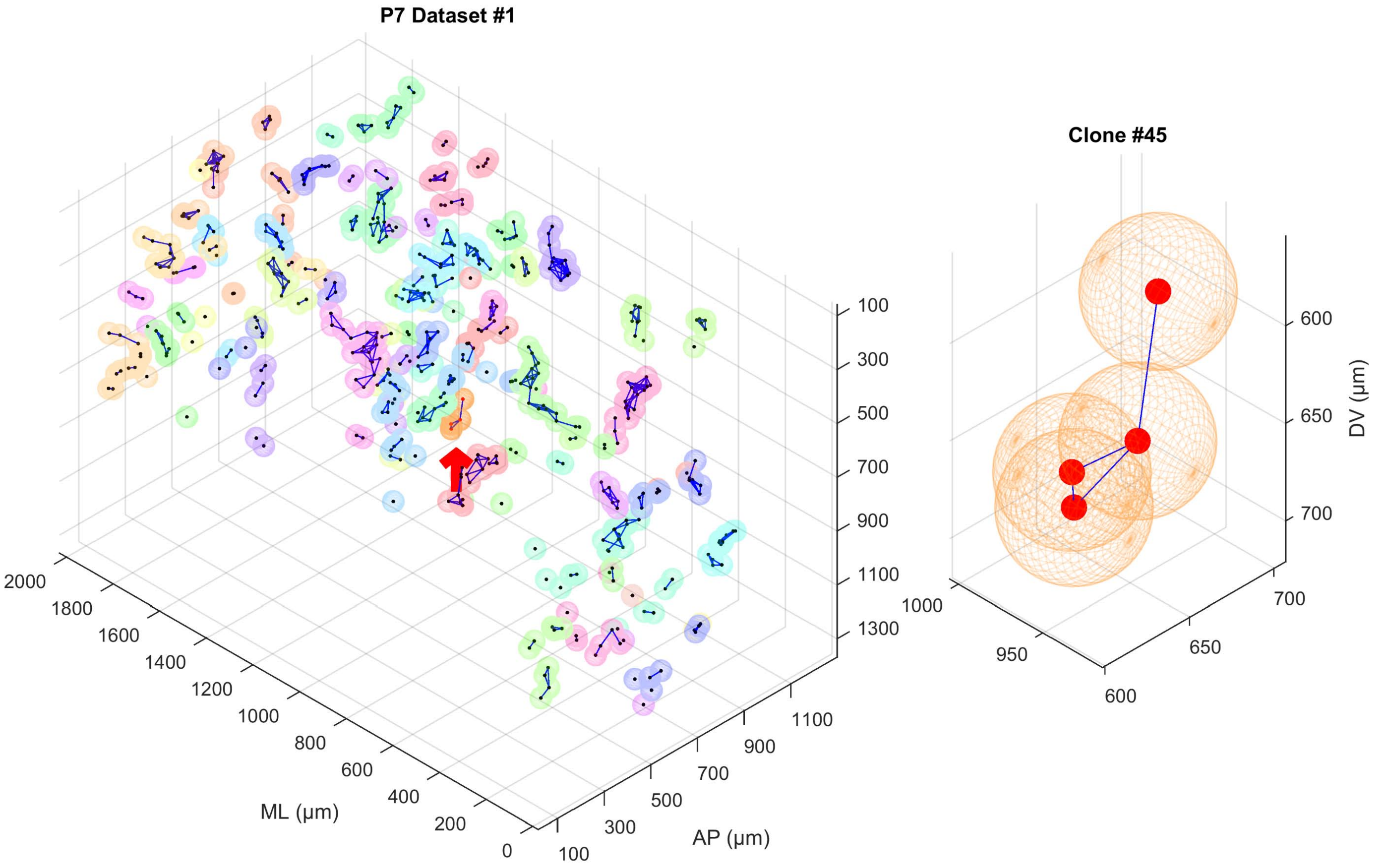
P7 Dataset #1
Clone #45
ML (µm)
AP (µm)
DV (µm)
2000
1800
1600
1400
1200
1000
800
600
400
200
0
100
300
500
700
900
1100
1300
950
650
600
700

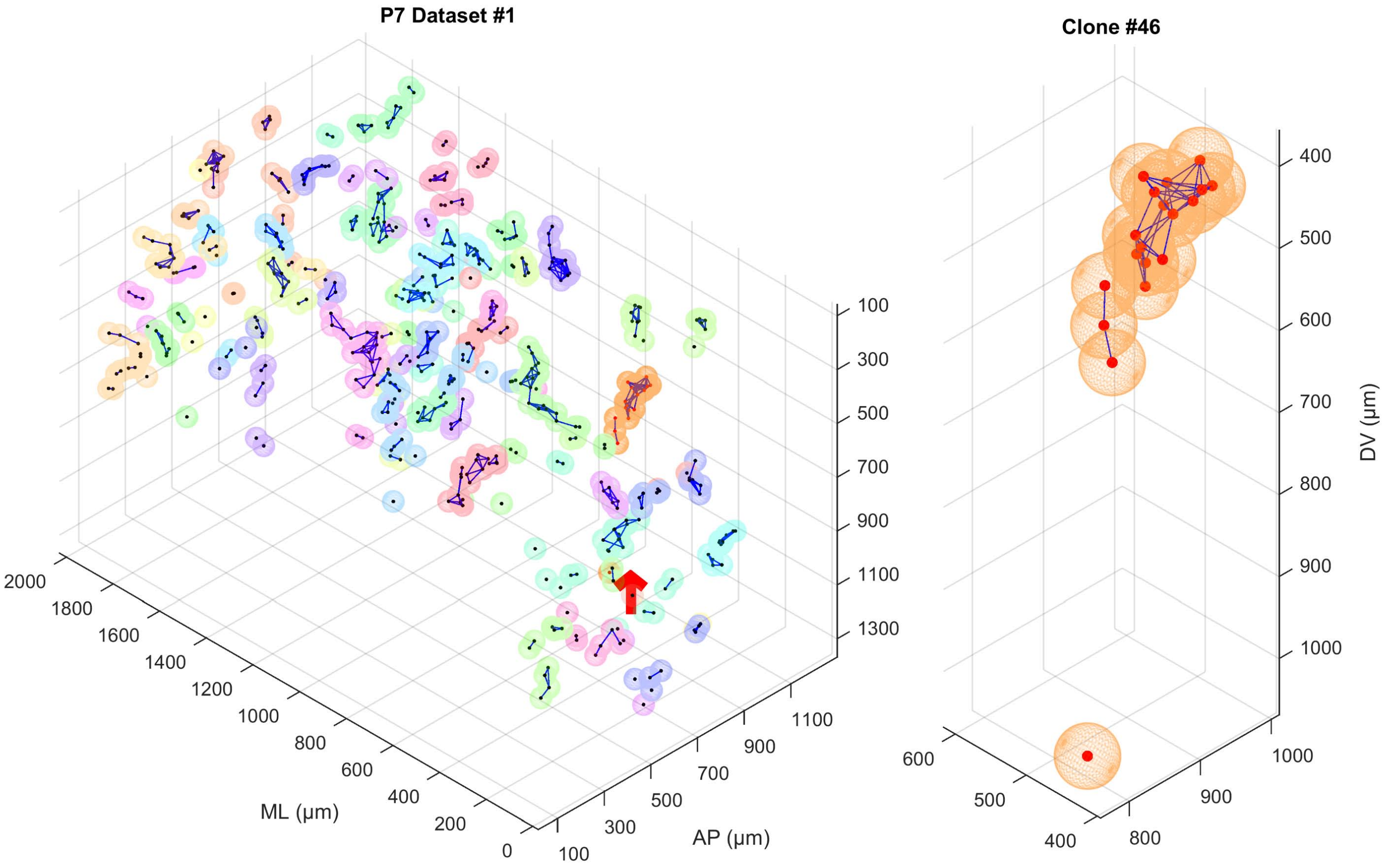

P7 Dataset #1
Clone #46
ML (µm)
AP (µm)
DV (µm)
2000
1800
1600
1400
1200
1000
800
600
400
200
0
100
300
500
700
900
1100
1300

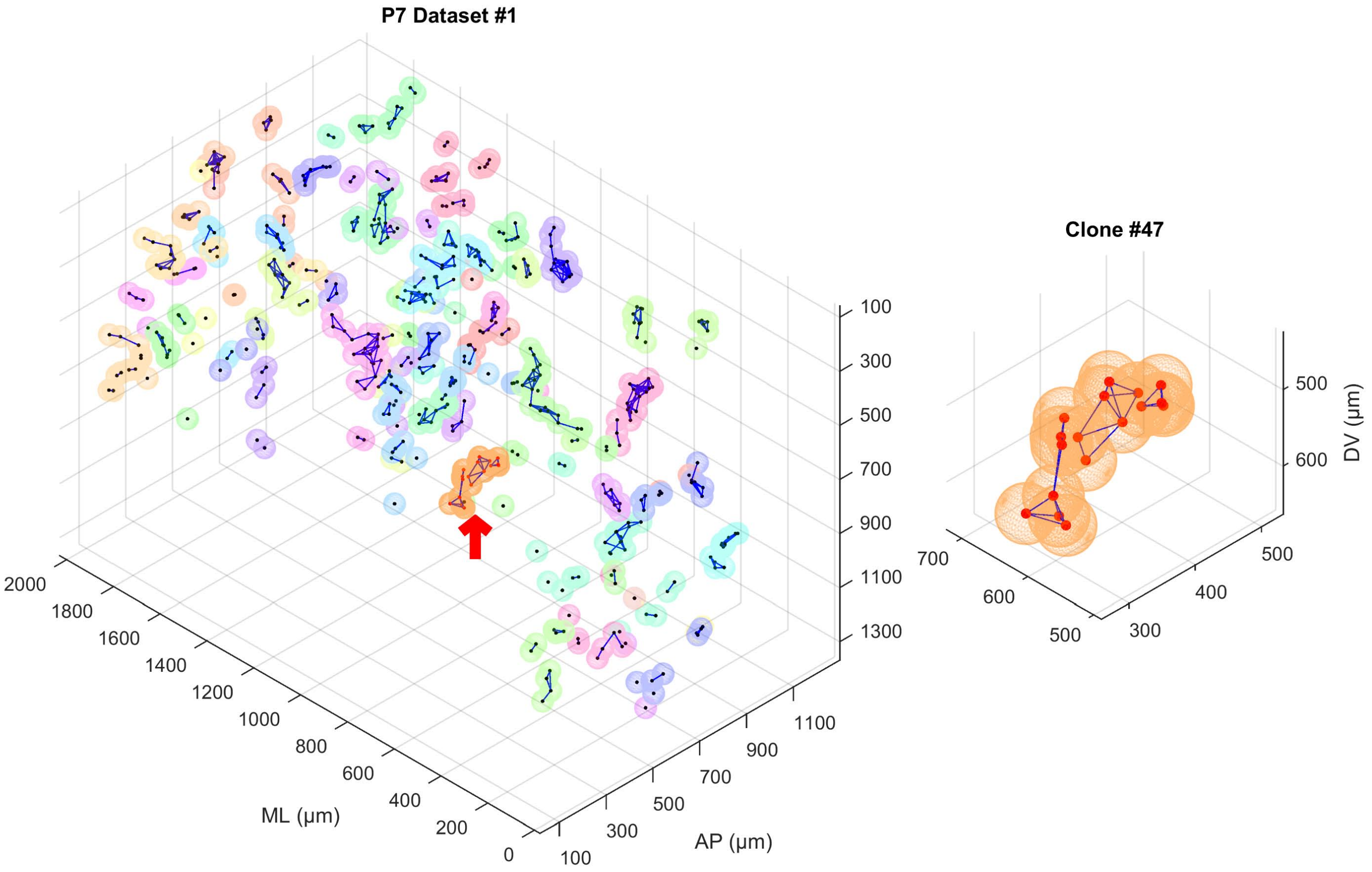

P7 Dataset #1
100
300
500
700
900
1100
1300
2000
1800
1600
1400
1200
1000
800
600
400
200
0
ML (µm)
100
300
500
700
900
1100
AP (µm)
Clone #47
500
600
DV (µm)
700
600
500
300
400
500

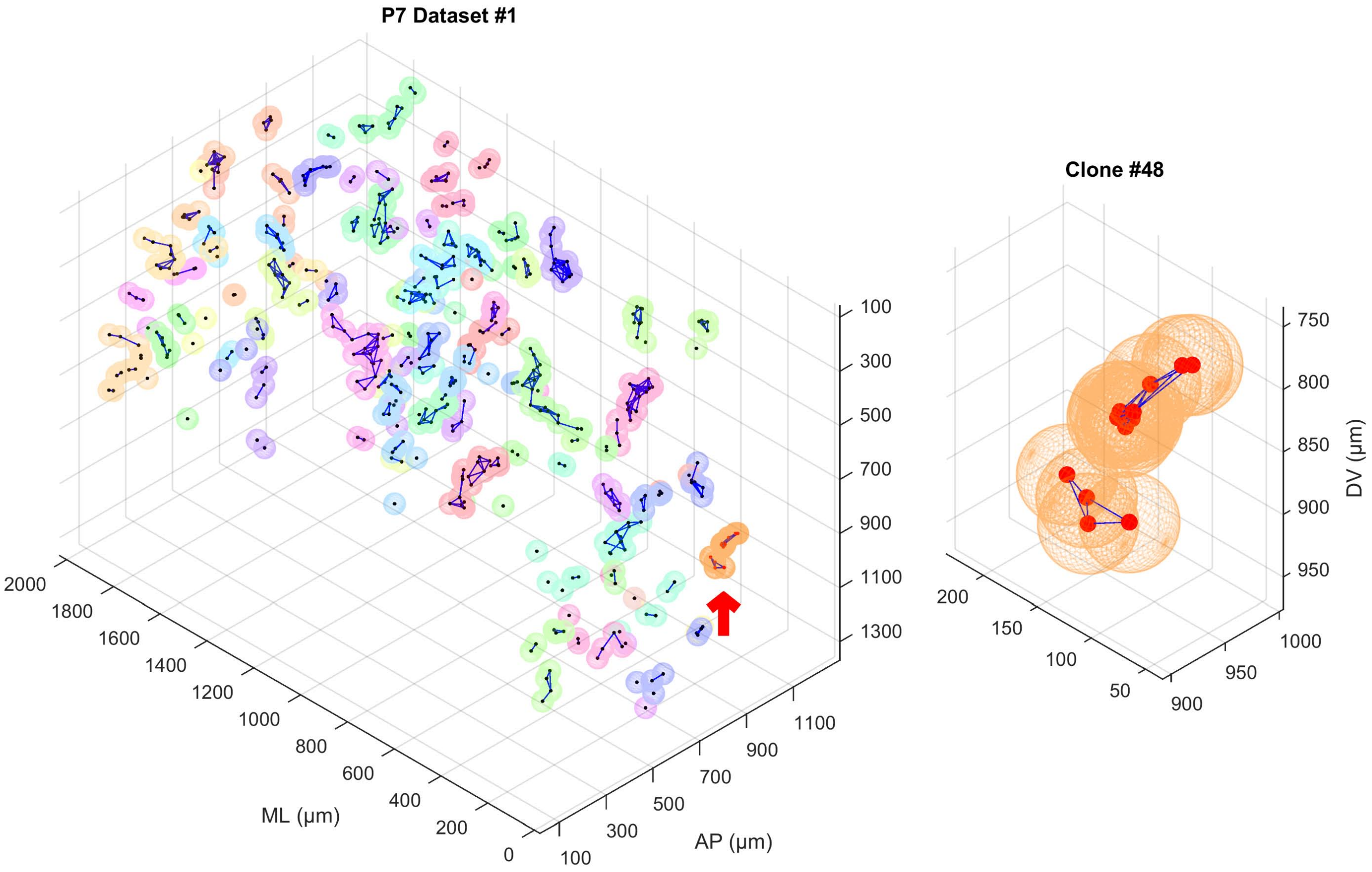
P7 Dataset #1
ML (µm)
AP (µm)
Clone #48
DV (µm)
2000
1800
1600
1400
1200
1000
800
600
400
200
0
100
300
500
700
900
1100
1300
750
800
850
900
950
1000
50
150
200

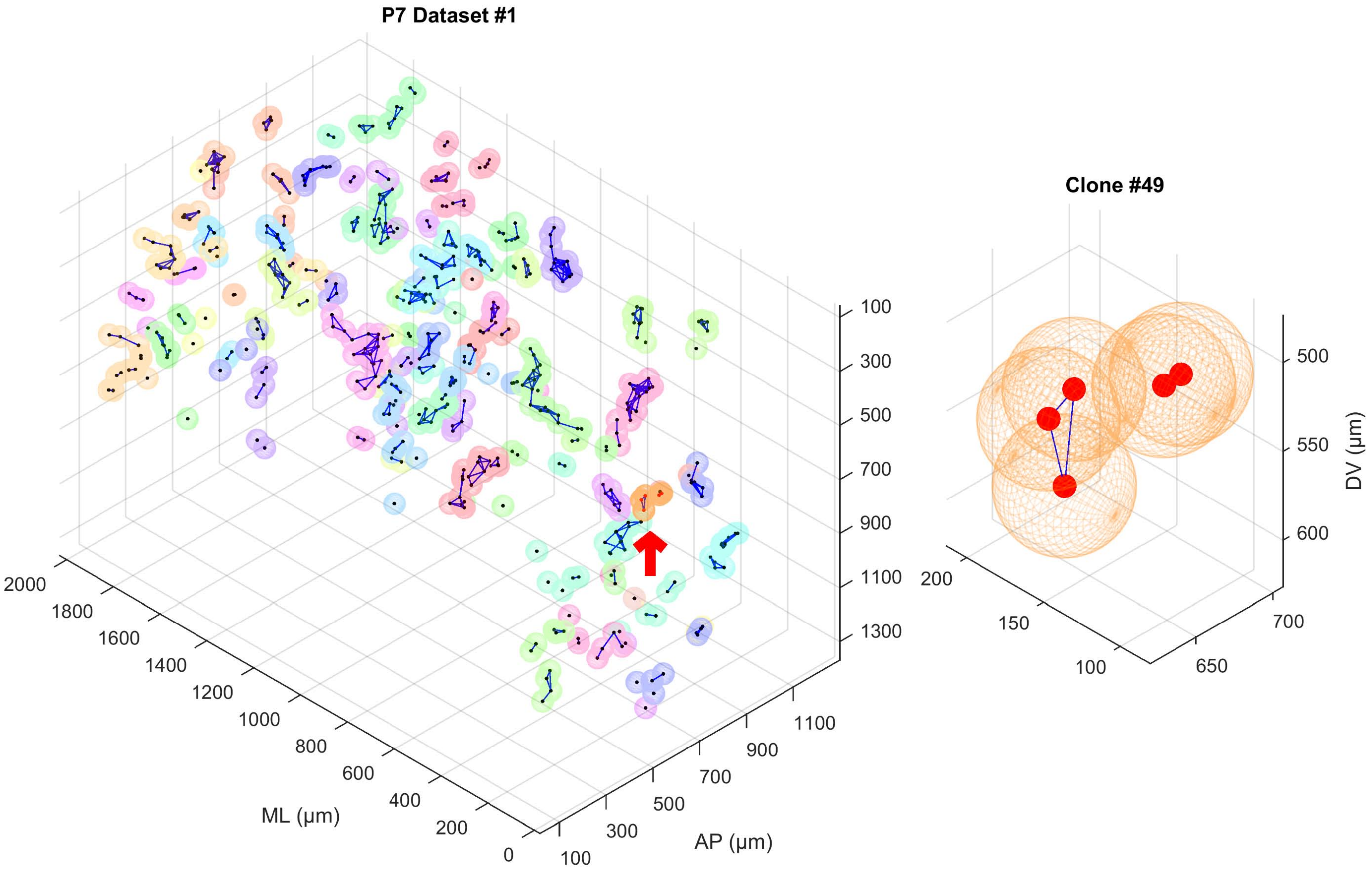

P7 Dataset #1
ML (µm)
2000
1800
1600
1400
1200
1000
800
600
400
200
0
AP (µm)
100
300
500
700
900
1100
100
300
500
700
900
1100
1300
Clone #49
DV (µm)
500
550
600
200
150
100
650
700

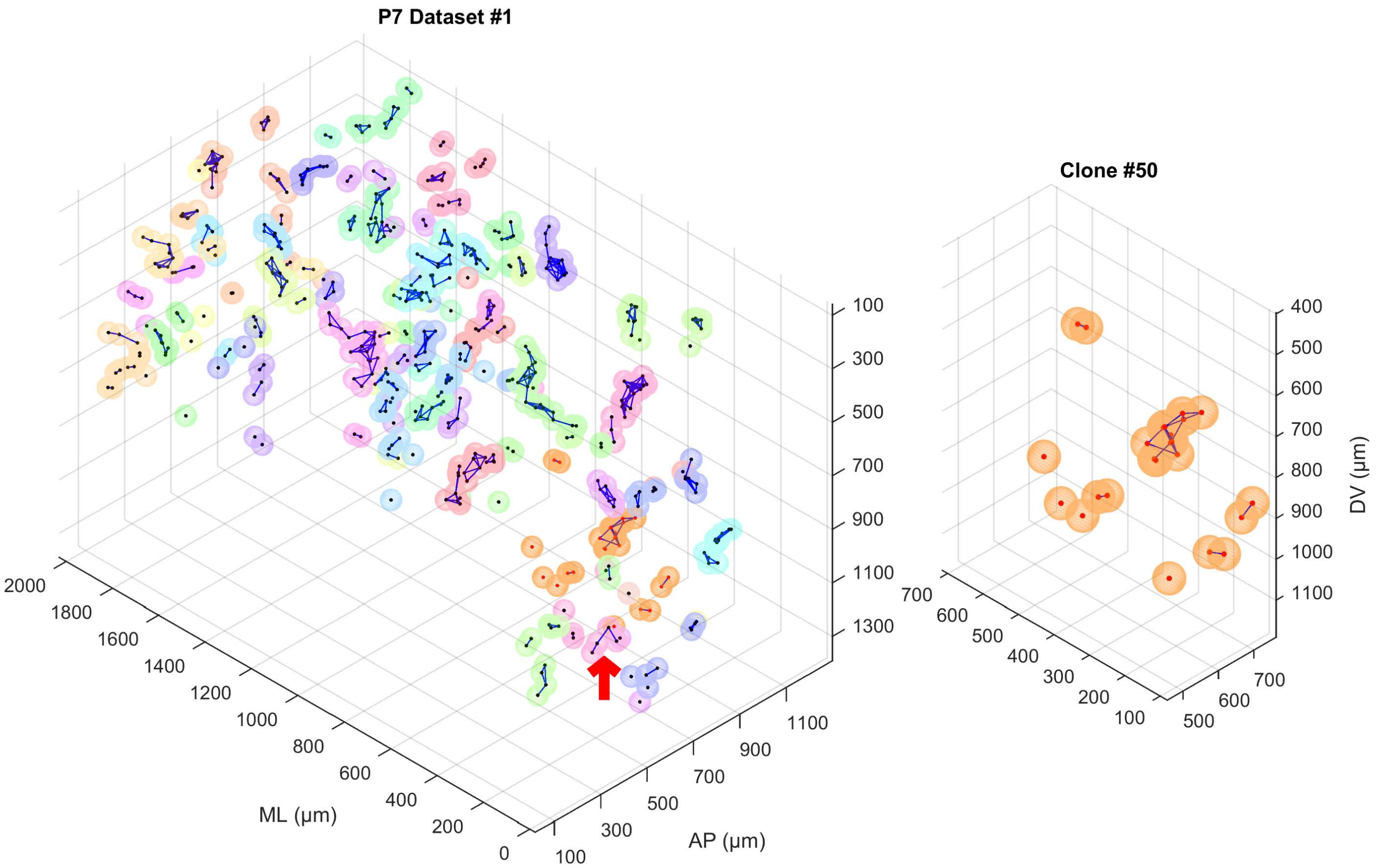
P7 Dataset #1
Clone #50
100
300
500
700
900
1100
1300
2000
1800
1600
1400
1200
1000
800
600
400
200
0
ML (μm)
100
300
500
700
900
1100
AP (μm)
400
500
600
700
800
900
1000
1100
DV (μm)
700
600
500
400
300
200
100
500
600
700

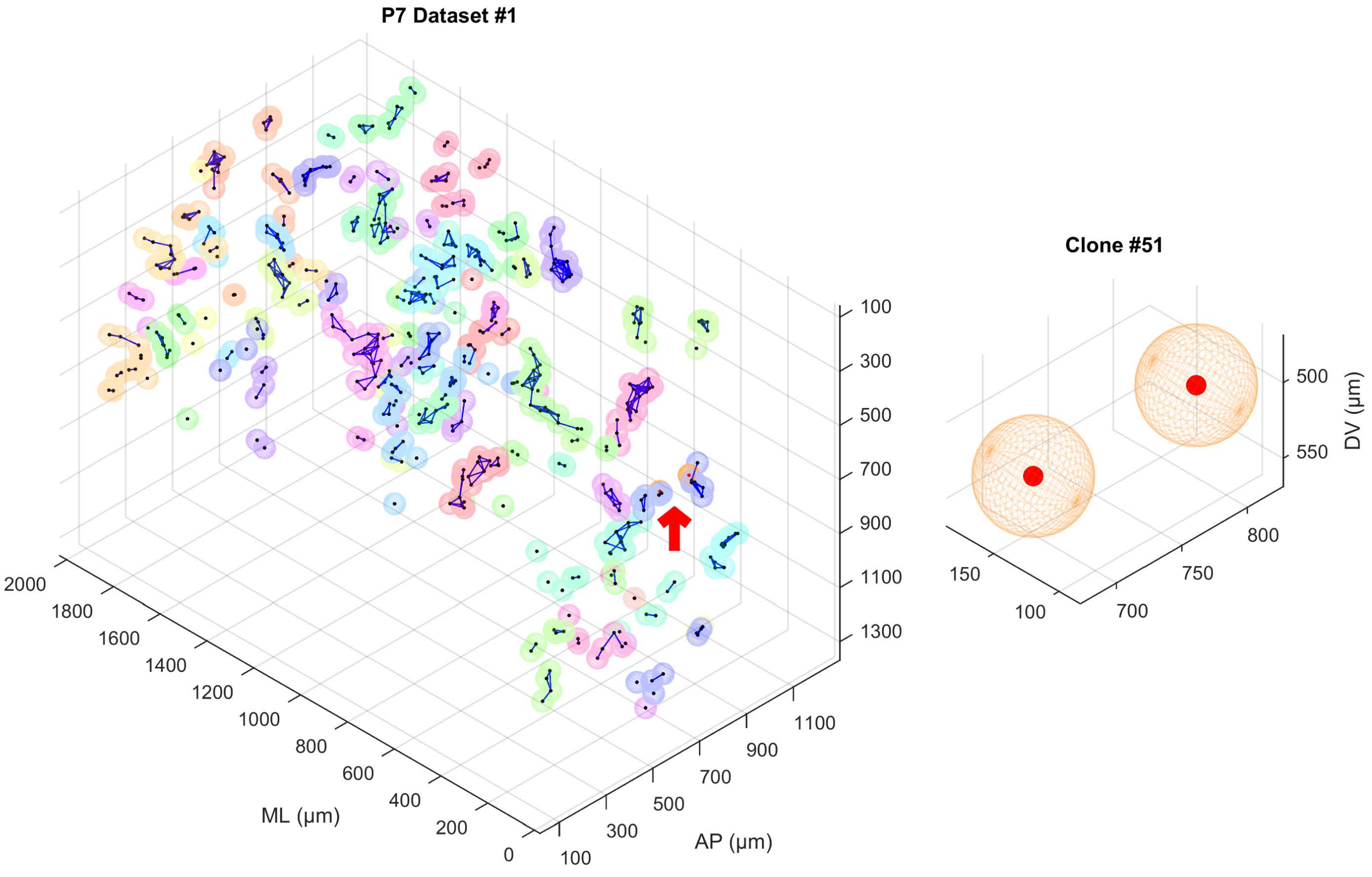

P7 Dataset #1
ML (µm)
AP (µm)
2000
1800
1600
1400
1200
1000
800
600
400
200
0
100
300
500
700
900
1100
1300
Clone #51
DV (µm)
500
550
150
100
700
750
800

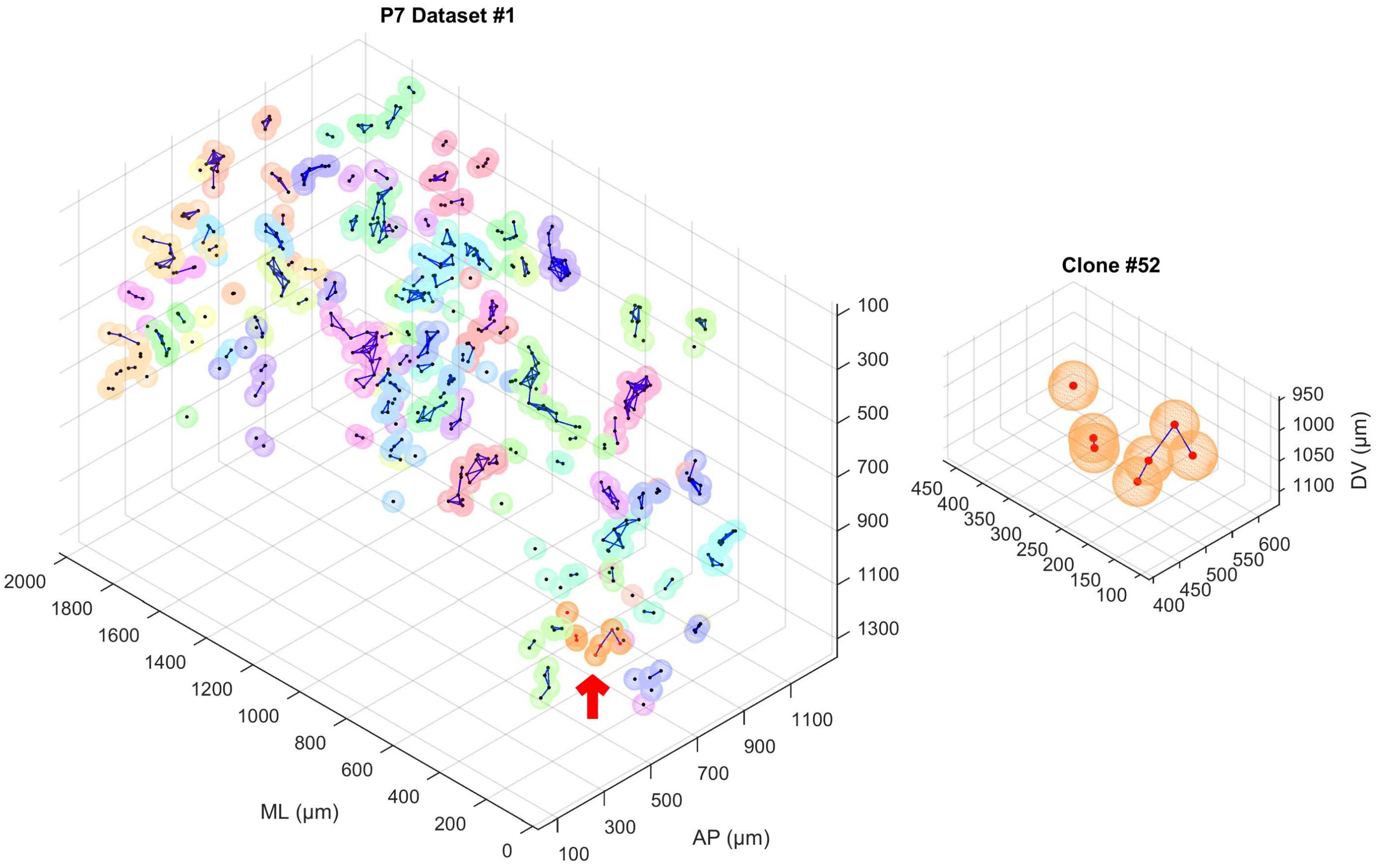
P7 Dataset #1
100
300
500
700
900
1100
1300
2000
1800
1600
1400
1200
1000
800
600
400
200
0
ML (µm)
100
300
500
700
900
1100
AP (µm)
Clone #52
950
1000
1050
1100
DV (µm)
450
400
350
300
250
200
150
100
400
450
500
550
600

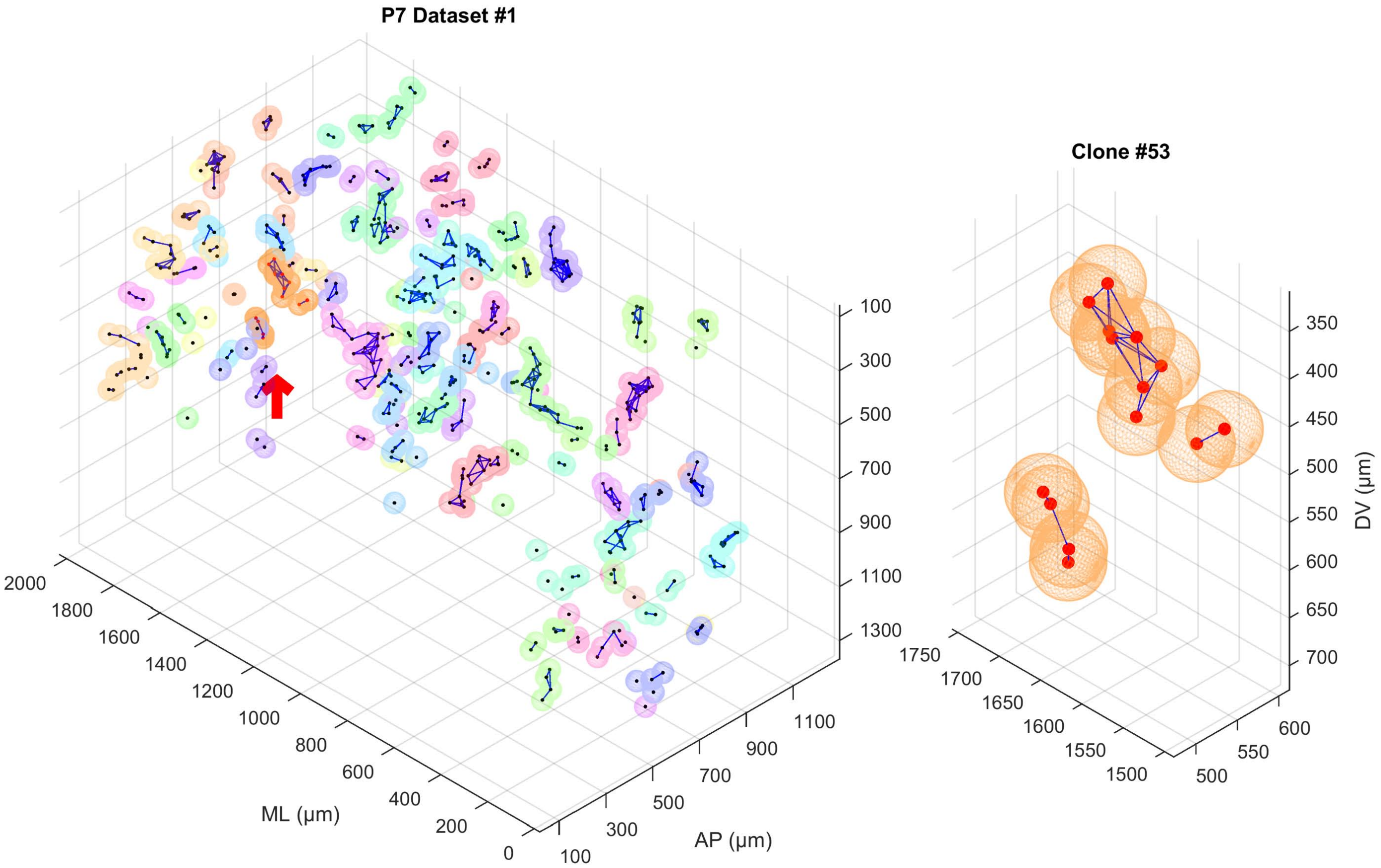

P7 Dataset #1
ML (µm)
AP (µm)
100
300
500
700
900
1100
1300
2000
1800
1600
1400
1200
1000
800
600
400
200
0
100
300
500
700
900
1100
Clone #53
DV (µm)
350
400
450
500
550
600
650
700
1750
1700
1650
1600
1550
1500
500
550
600

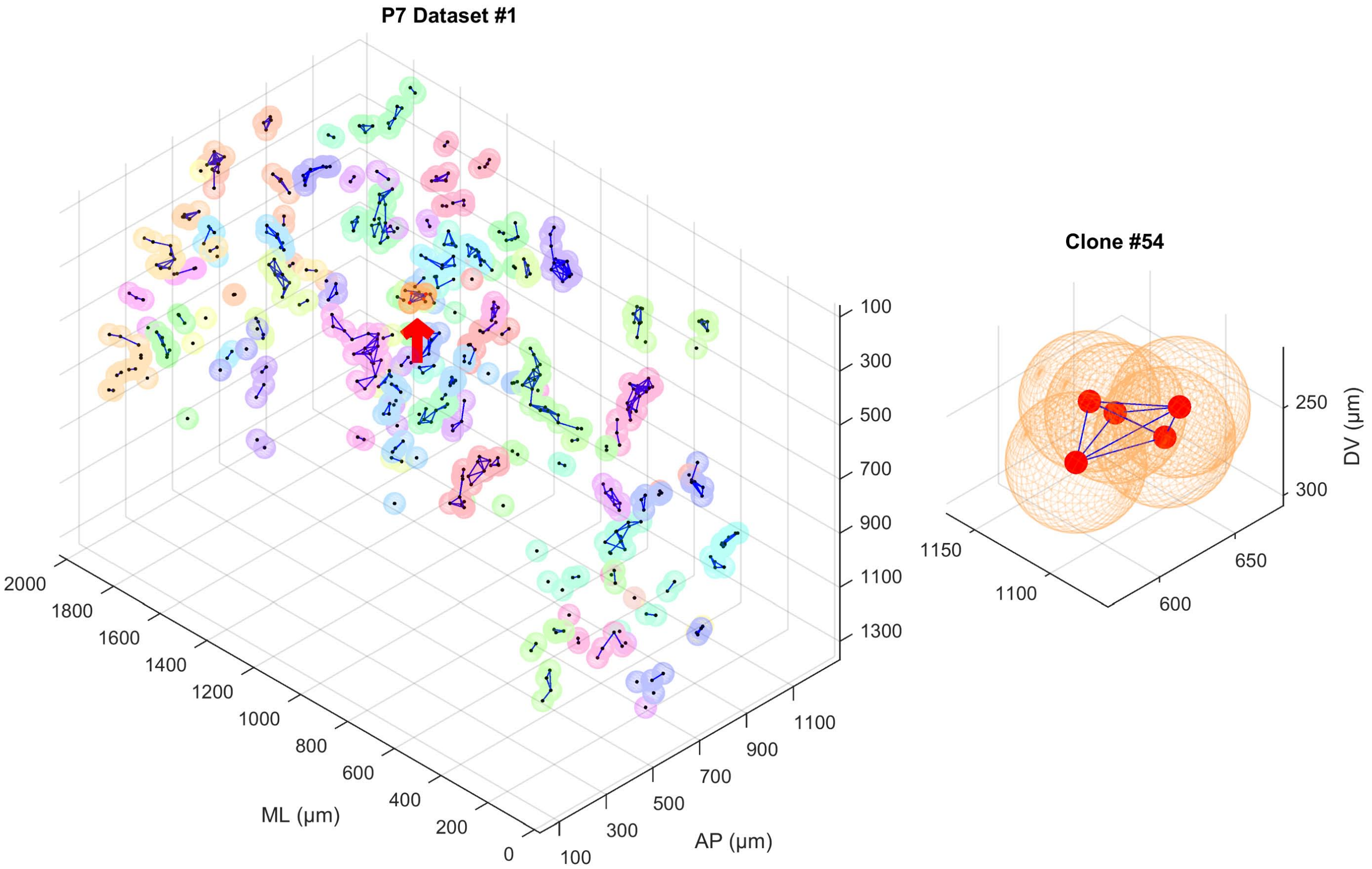

P7 Dataset #1
100
300
500
700
900
1100
1300
2000
1800
1600
1400
1200
1000
800
600
400
200
0
ML (µm)
100
300
500
700
900
1100
AP (µm)
Clone #54
250
300
DV (µm)
1150
1100
600
650

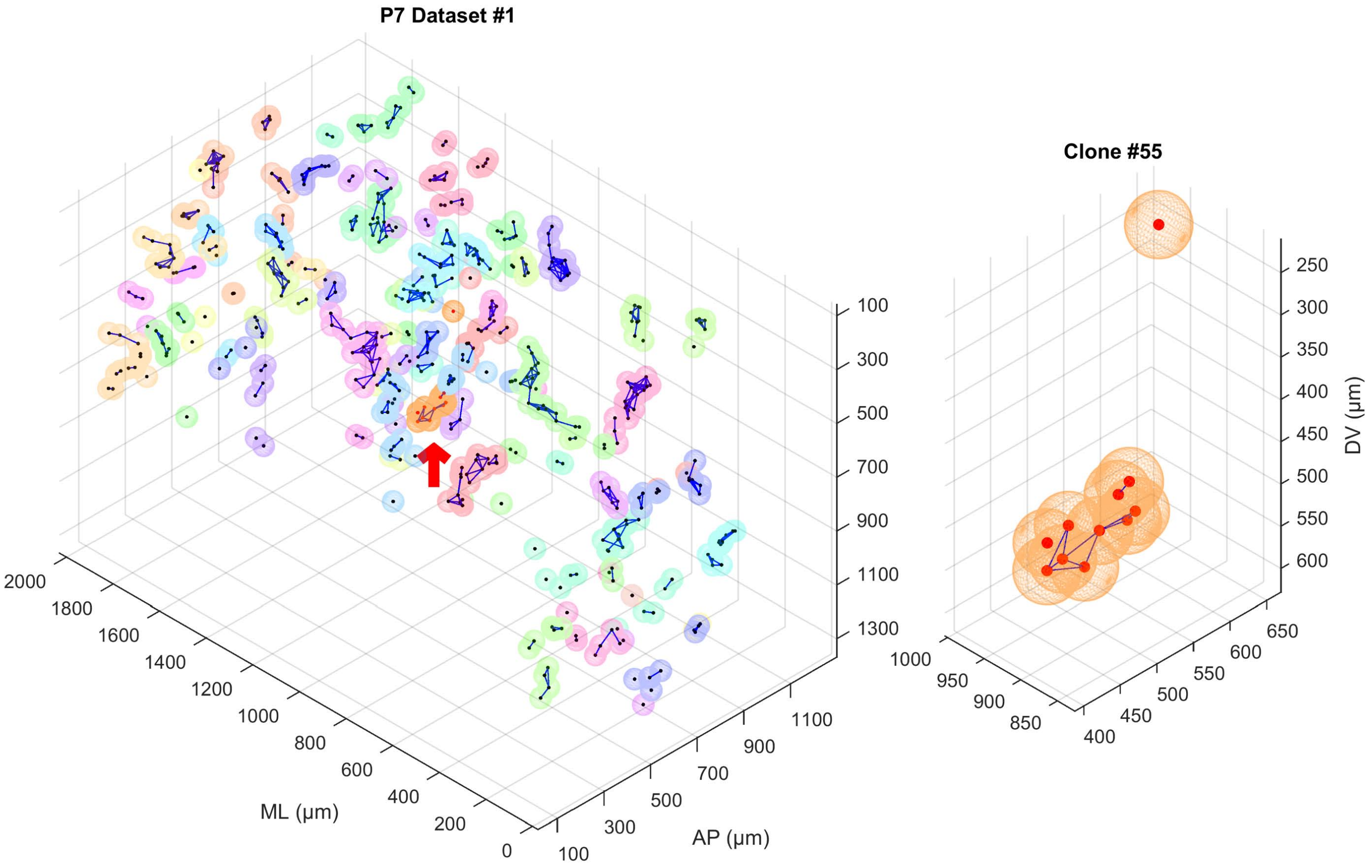
P7 Dataset #1
Clone #55
ML (µm)
AP (µm)
DV (µm)
2000
1800
1600
1400
1200
1000
800
600
400
200
0
100
300
500
700
900
1100
1300
250
300
350
400
450
500
550
600
650
850
900
950
1000

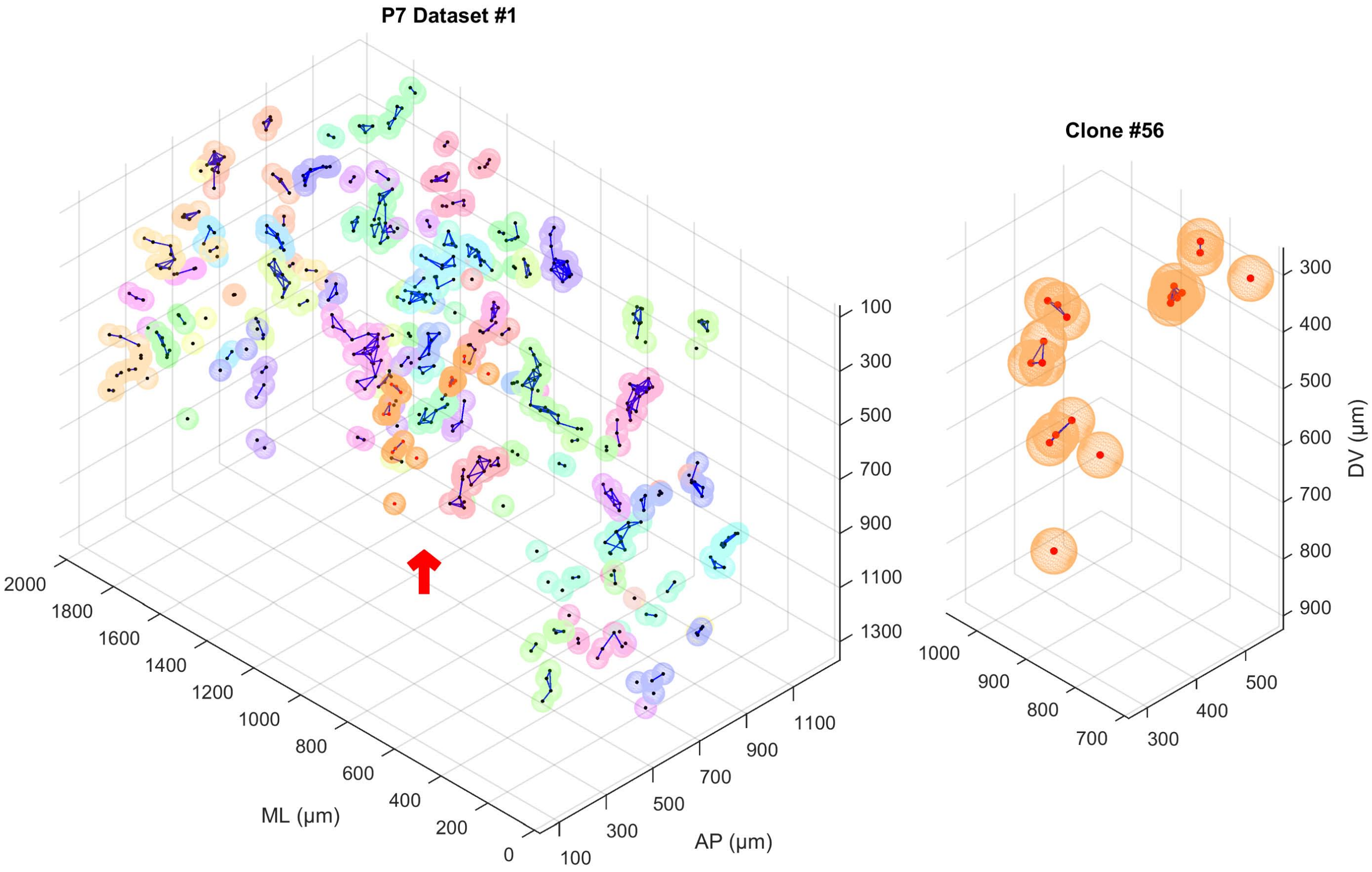

P7 Dataset #1
100
300
500
700
900
1100
1300
2000
1800
1600
1400
1200
1000
800
600
400
200
0
ML (μm)
100
300
500
700
900
1100
AP (μm)
Clone #56
300
400
500
600
700
800
900
DV (μm)
1000
900
800
700
300
400
500

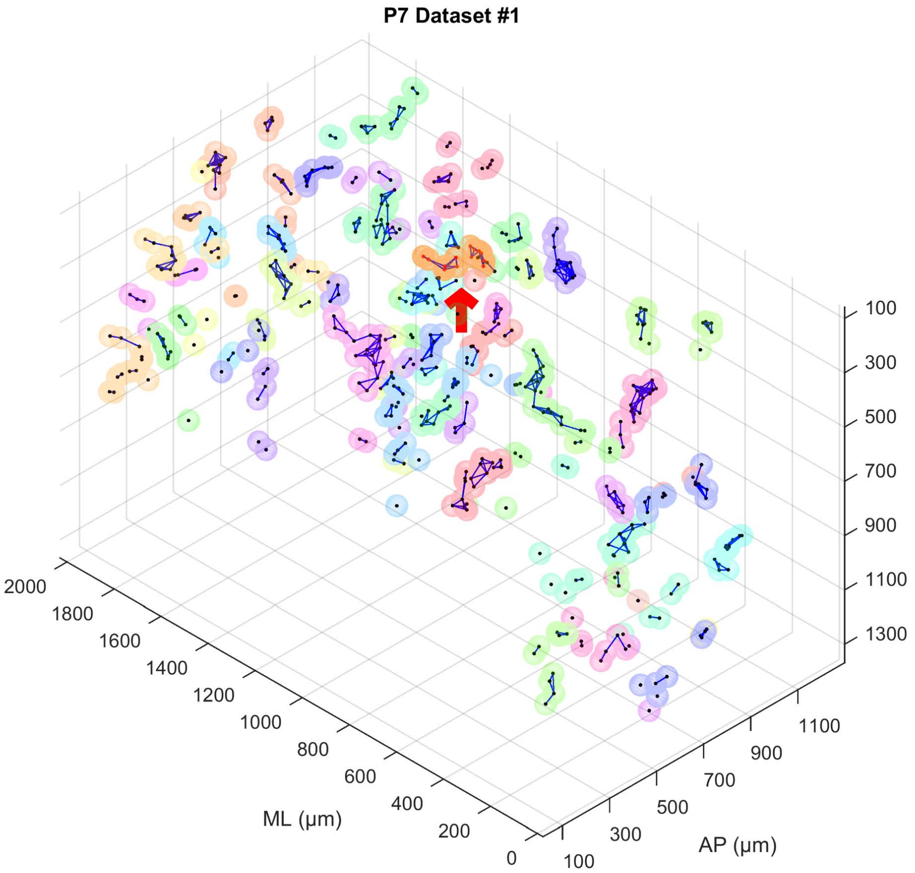
P7 Dataset #1
100
300
500
700
900
1100
1300
2000
1800
1600
1400
1200
1000
800
600
400
200
0
ML (µm)
100
300
500
700
900
1100
AP (µm)

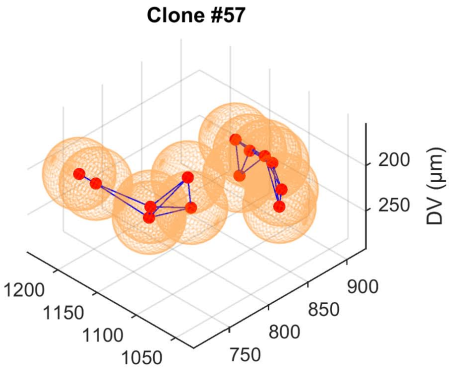
Clone #57
200
250
DV (µm)
1200
1150
1100
1050
900
850
800
750

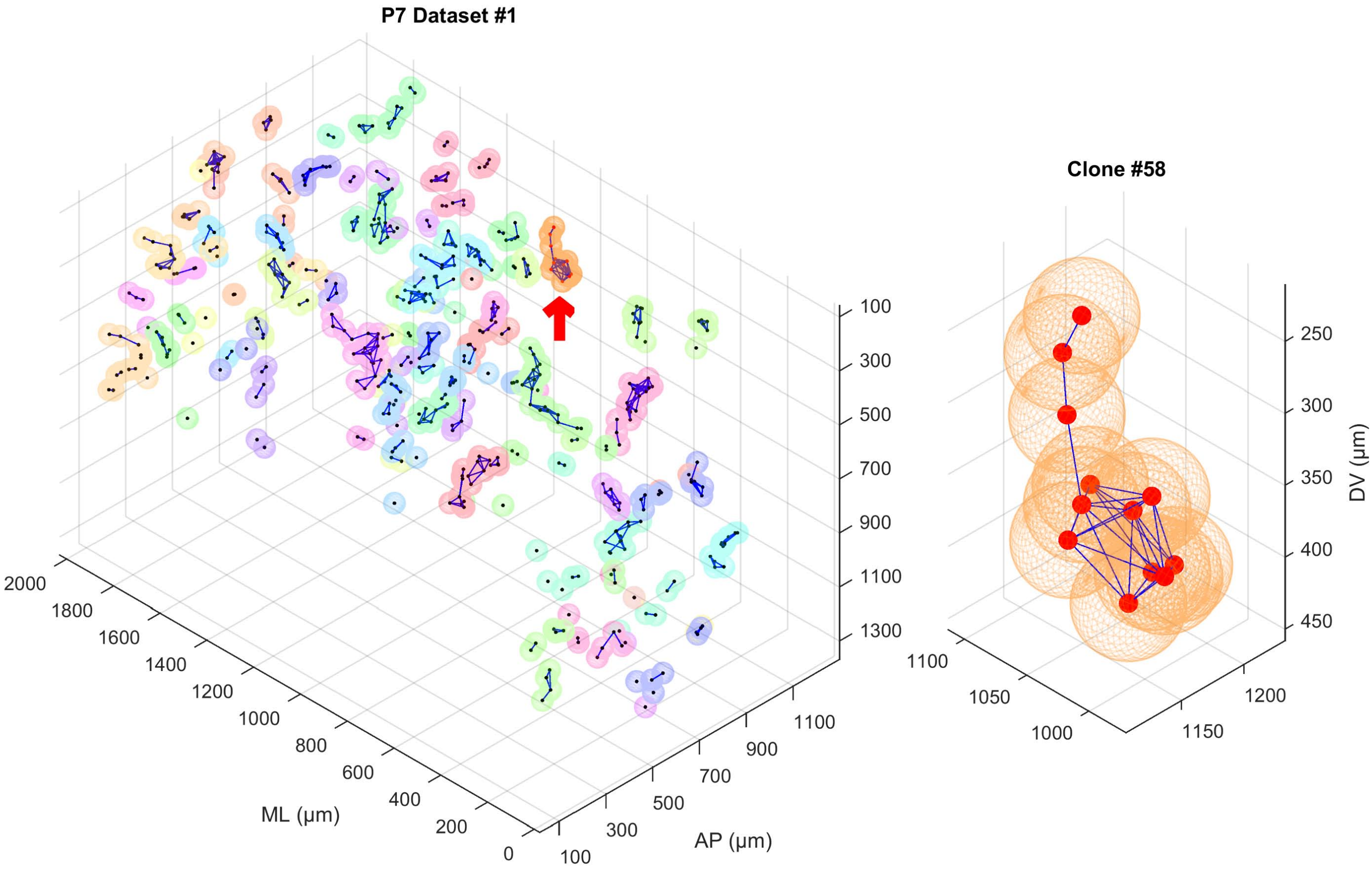

P7 Dataset #1
100
300
500
700
900
1100
1300
2000
1800
1600
1400
1200
1000
800
600
400
200
0
ML (μm)
100
300
500
700
900
1100
AP (μm)
Clone #58
250
300
350
400
450
DV (μm)
1100
1050
1000
1150
1200

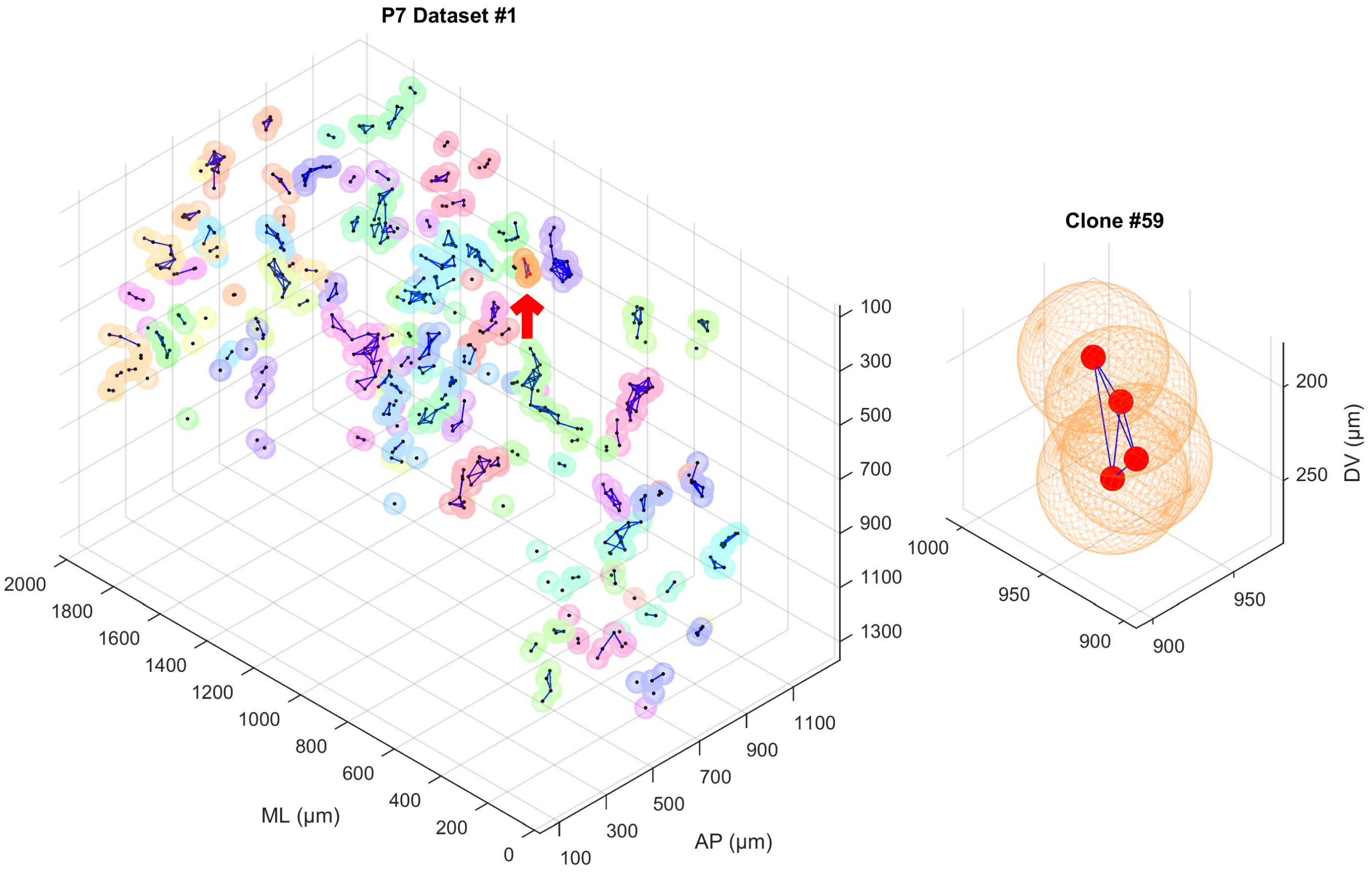

P7 Dataset #1
Clone #59
ML (µm)
AP (µm)
DV (µm)
2000
1800
1600
1400
1200
1000
800
600
400
200
0
100
300
500
700
900
1100
1300
950
900
250
200

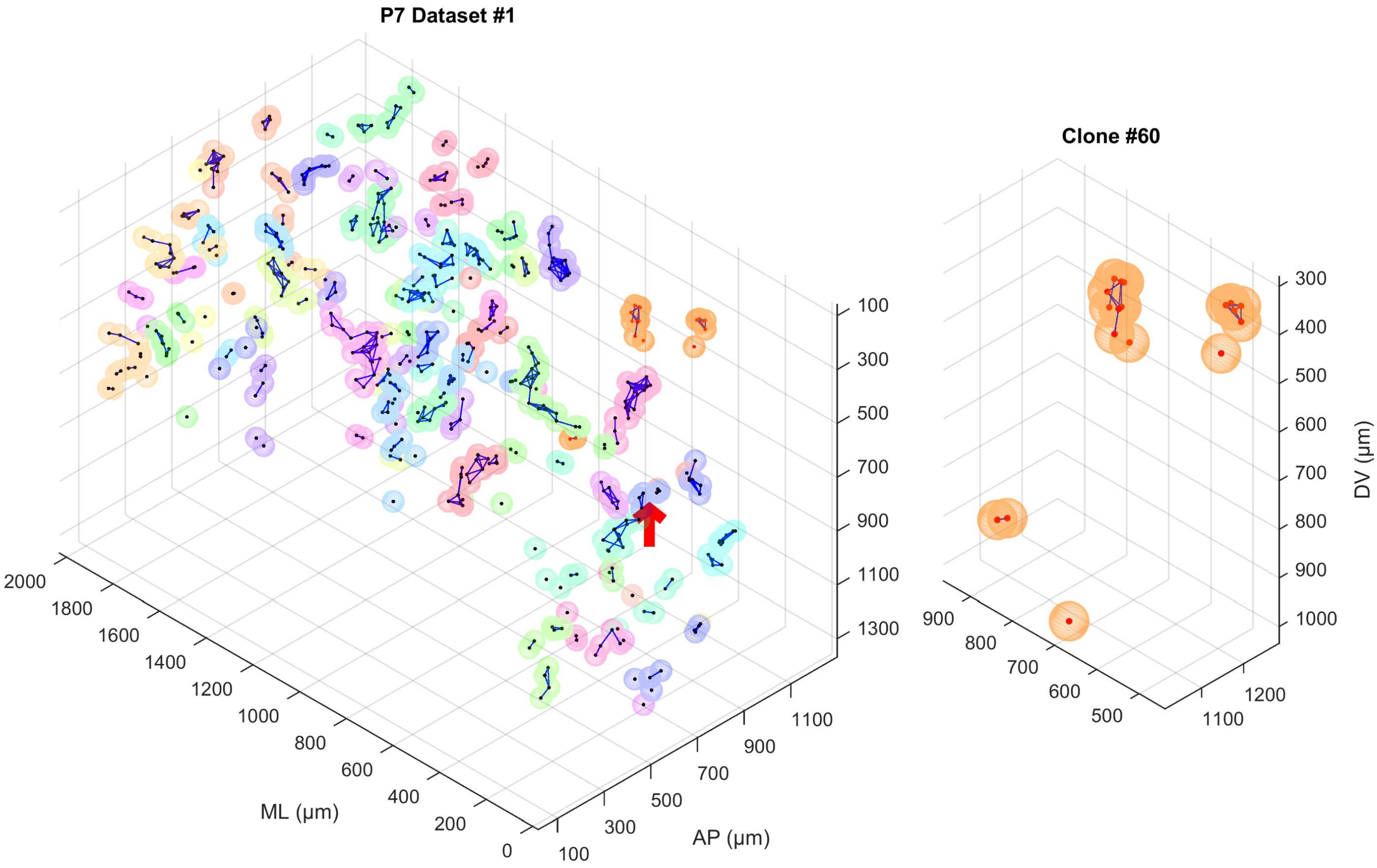

P7 Dataset #1
ML (µm)
AP (µm)
2000
1800
1600
1400
1200
1000
800
600
400
200
0
100
300
500
700
900
1100
1300
Clone #60
DV (µm)
300
400
500
600
700
800
900
1000
1100
1200

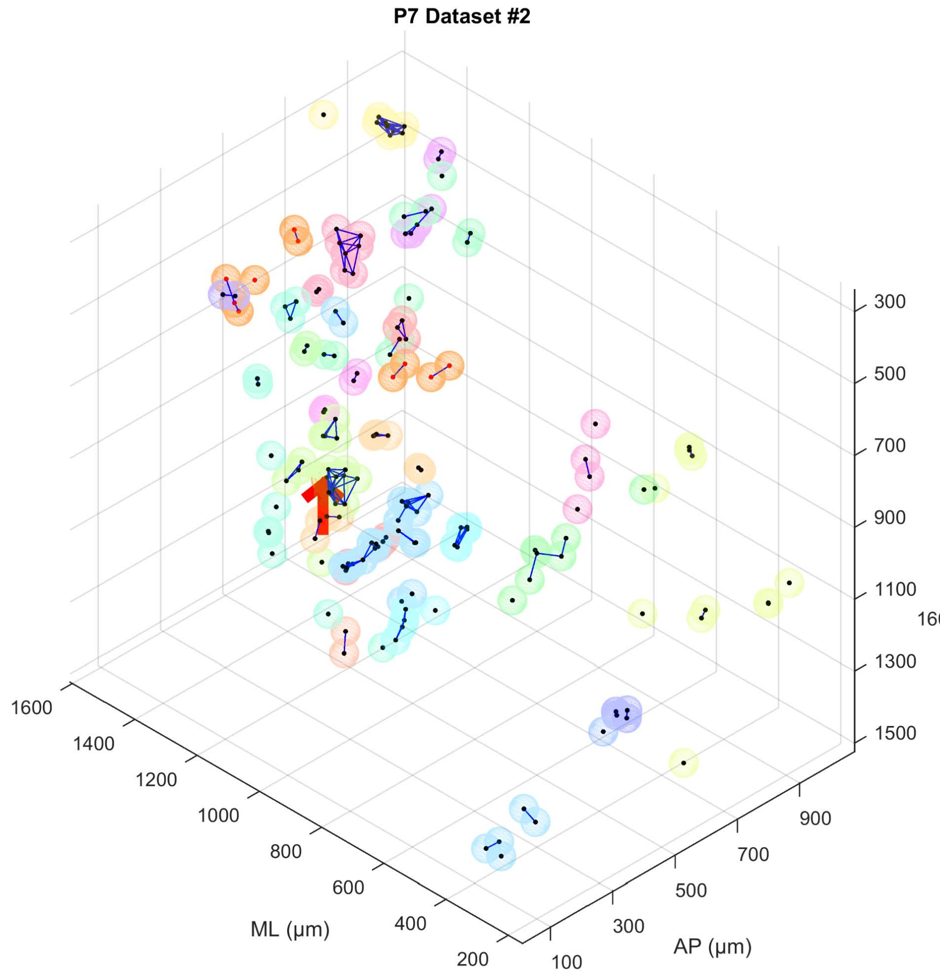
P7 Dataset #2
1
300
500
700
900
1100
1300
1500
1600
1400
1200
1000
800
600
400
200
ML (µm)
100
300
500
700
900
AP (µm)

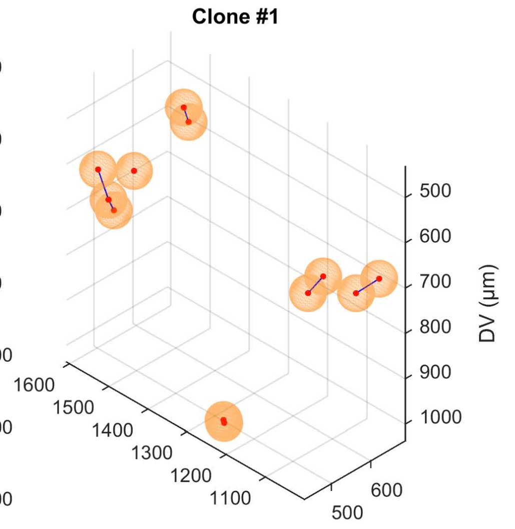
Clone #1
500
600
700
800
900
1000
DV (µm)
1600
1500
1400
1300
1200
1100
500
600

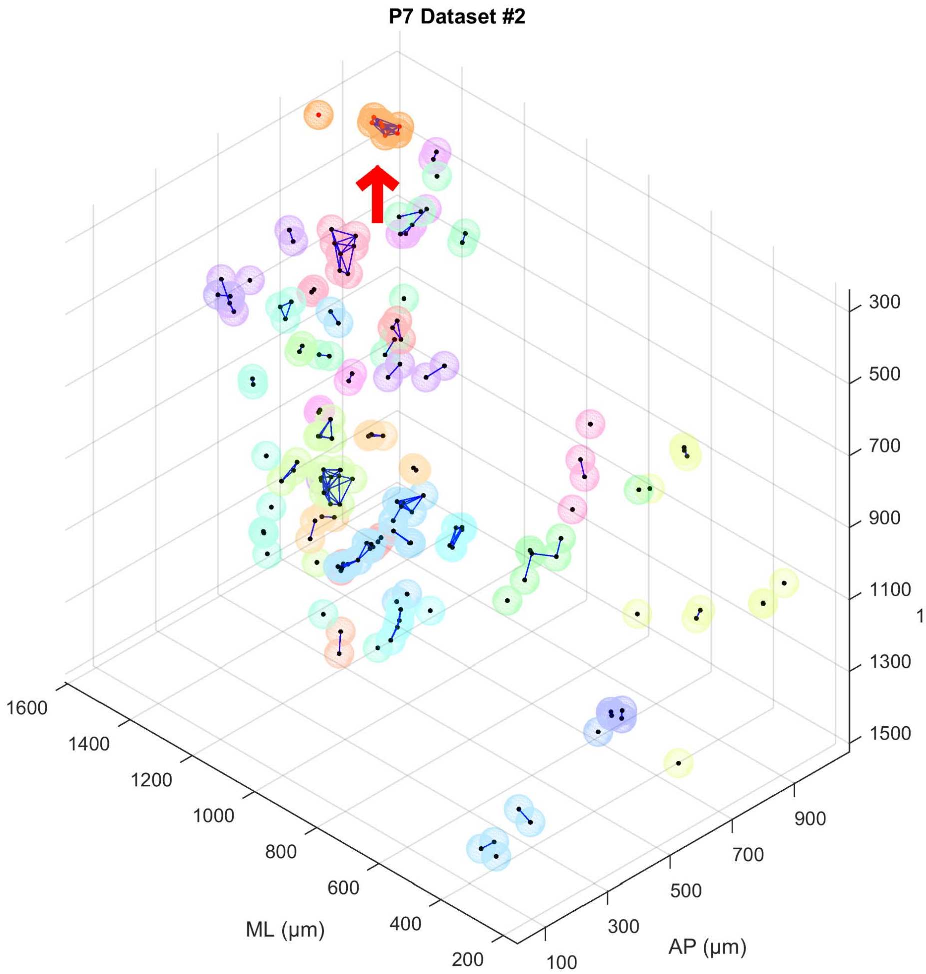

P7 Dataset #2
300
500
700
900
1100
1300
1500
1600
1400
1200
1000
800
600
400
200
ML (µm)
100
300
500
700
900
AP (µm)

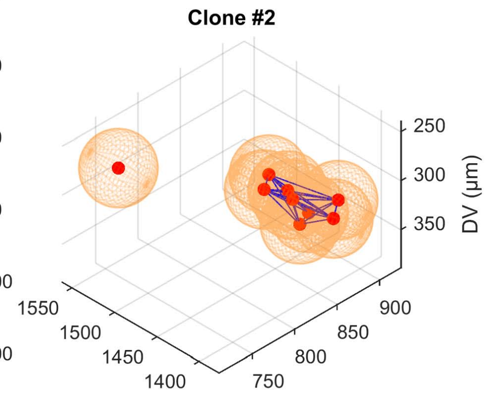

Clone #2
250
300
350
DV (µm)
1550
1500
1450
1400
750
800
850
900

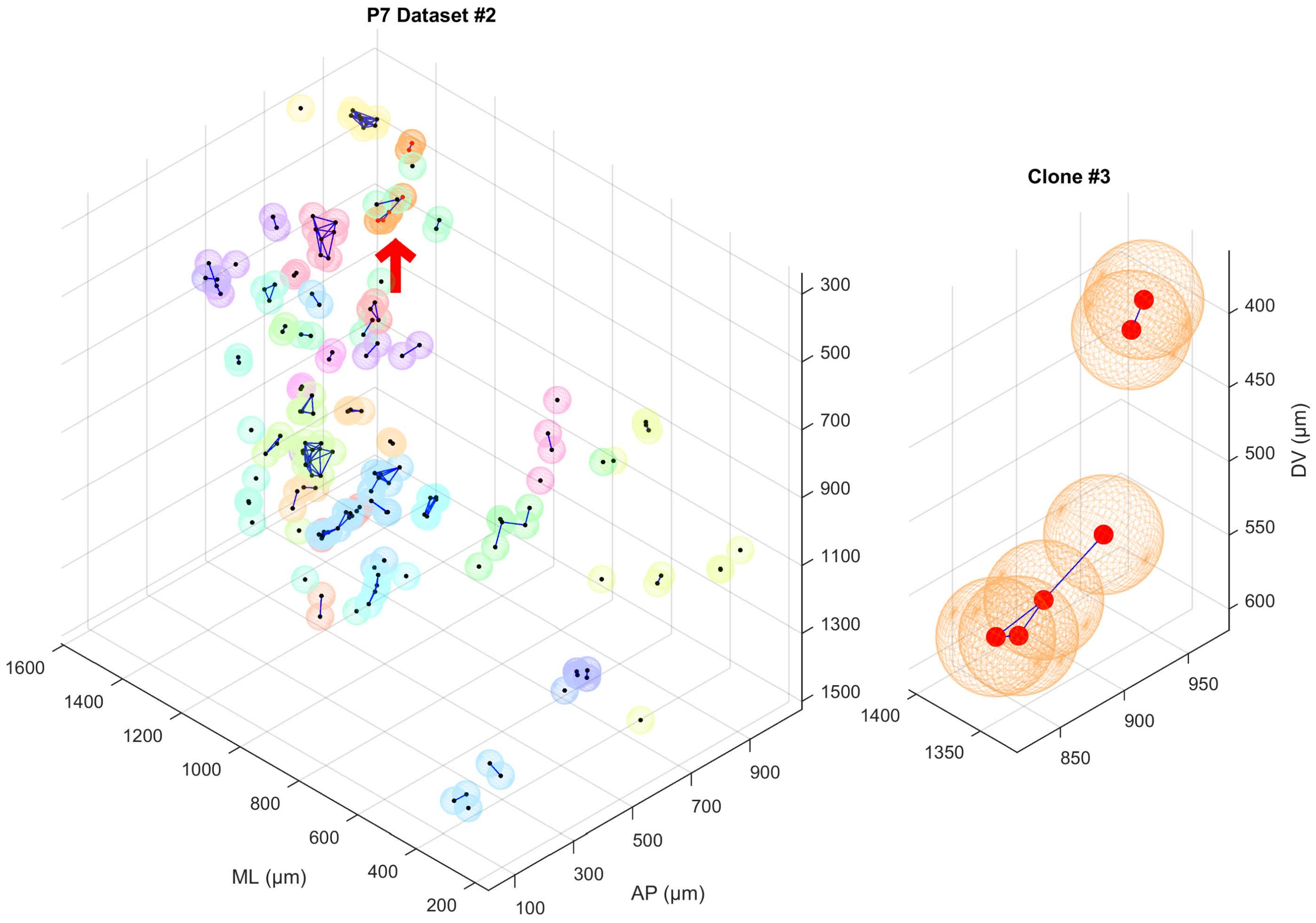

P7 Dataset #2
300
500
700
900
1100
1300
1500
1600
1400
1200
1000
800
600
400
200
ML (µm)
100
300
500
700
900
AP (µm)
Clone #3
400
450
500
550
600
DV (µm)
1400
1350
850
900
950

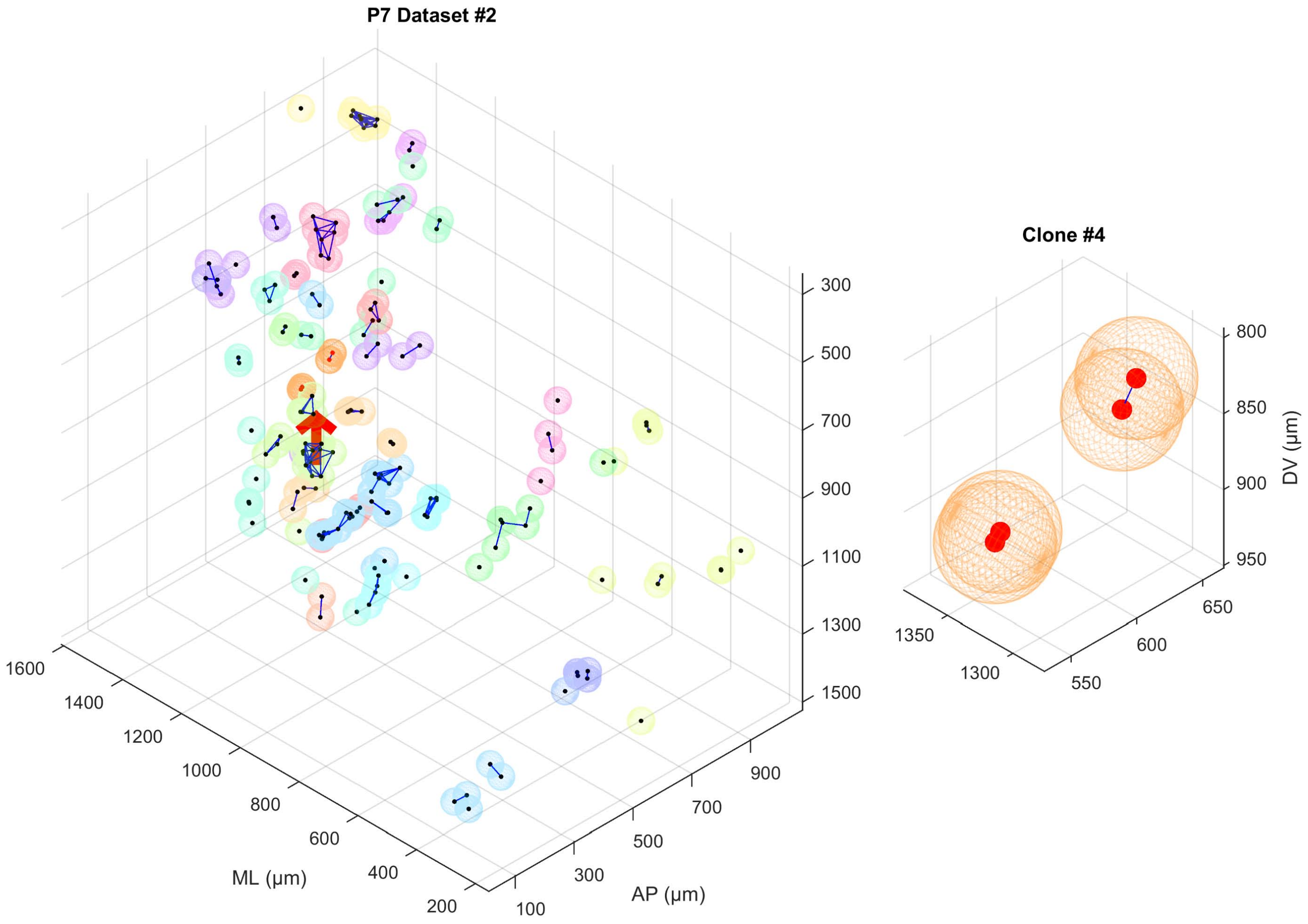
P7 Dataset #2
300
500
700
900
1100
1300
1500
1600
1400
1200
1000
800
600
400
200
ML (µm)
100
300
500
700
900
AP (µm)
Clone #4
800
850
900
950
DV (µm)
1350
1300
550
600
650

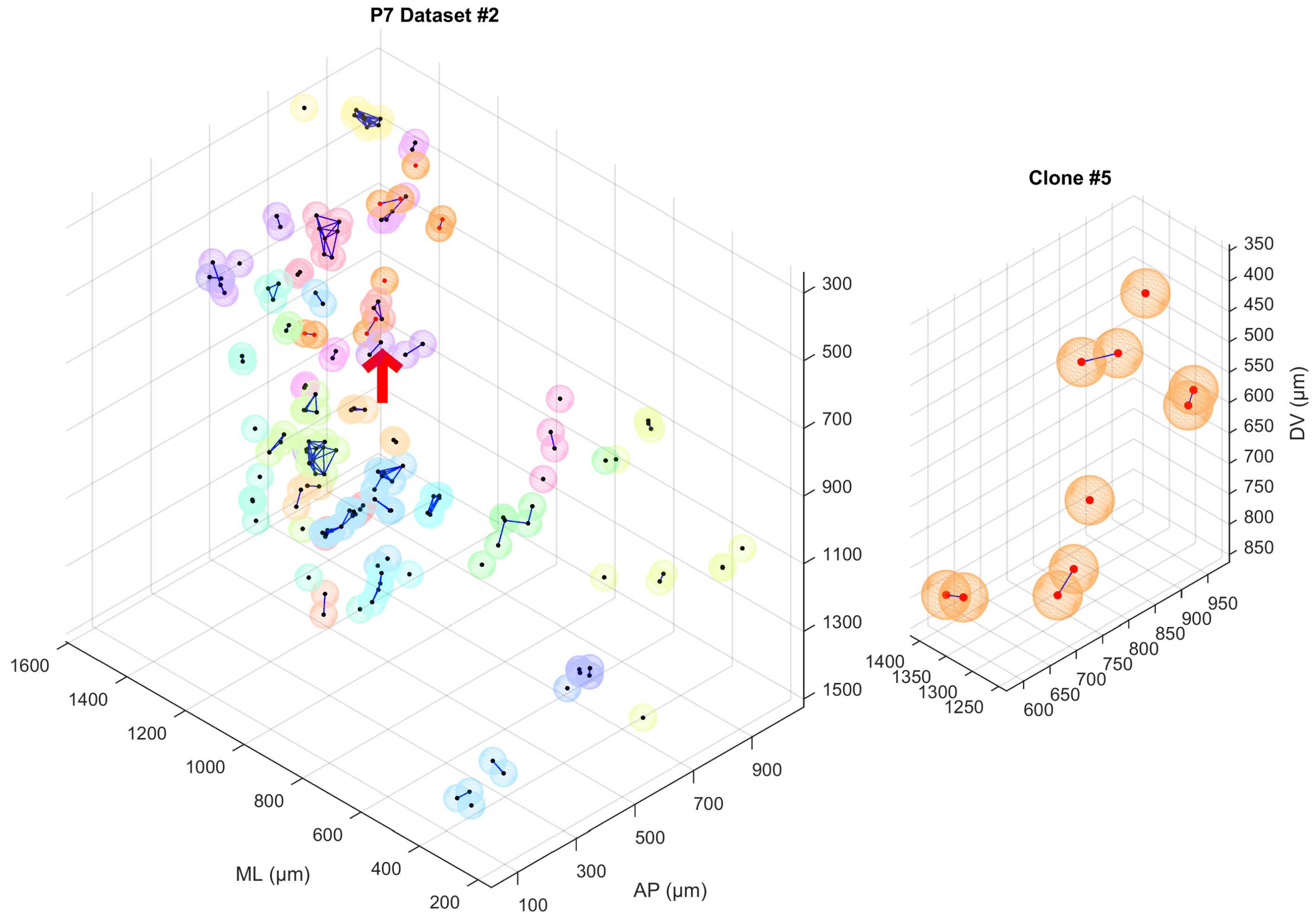
P7 Dataset #2
300
500
700
900
1100
1300
1500
1600
1400
1200
1000
800
600
400
200
ML (µm)
100
300
500
700
900
AP (µm)
Clone #5
350
400
450
500
550
600
650
700
750
800
850
DV (µm)
1400
1350
1300
1250
600
650
700
750
800
850
900
950

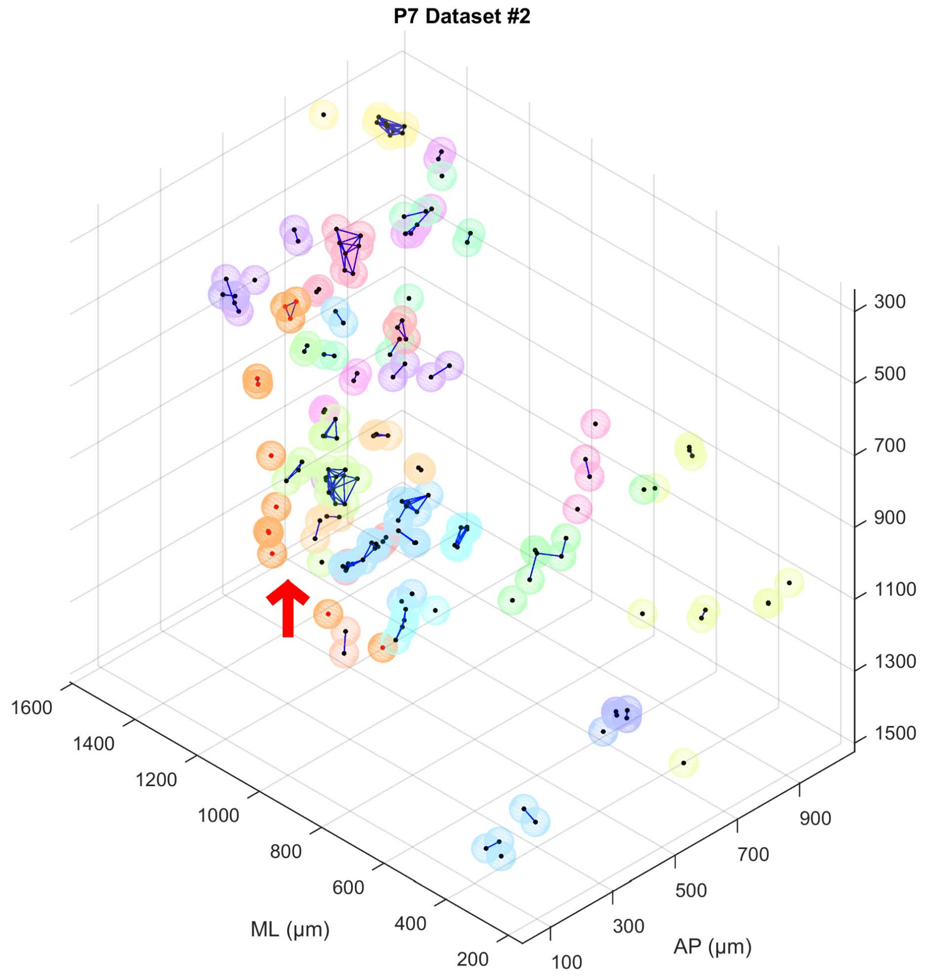
P7 Dataset #2
ML (µm)
1600
1400
1200
1000
800
600
400
200
AP (µm)
100
300
500
700
900
300
500
700
900
1100
1300
1500

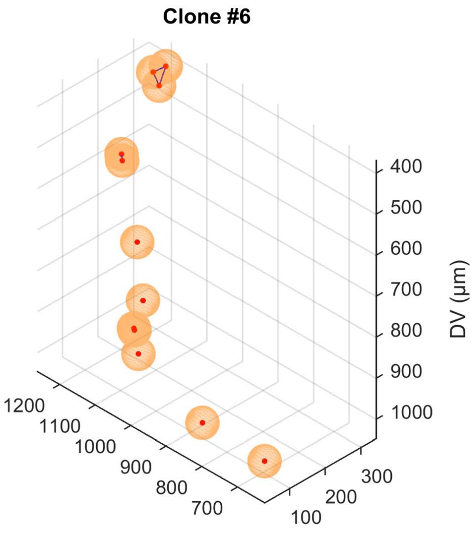
Clone #6
DV (µm)
400
500
600
700
800
900
1000
1200
1100
1000
900
800
700
100
200
300

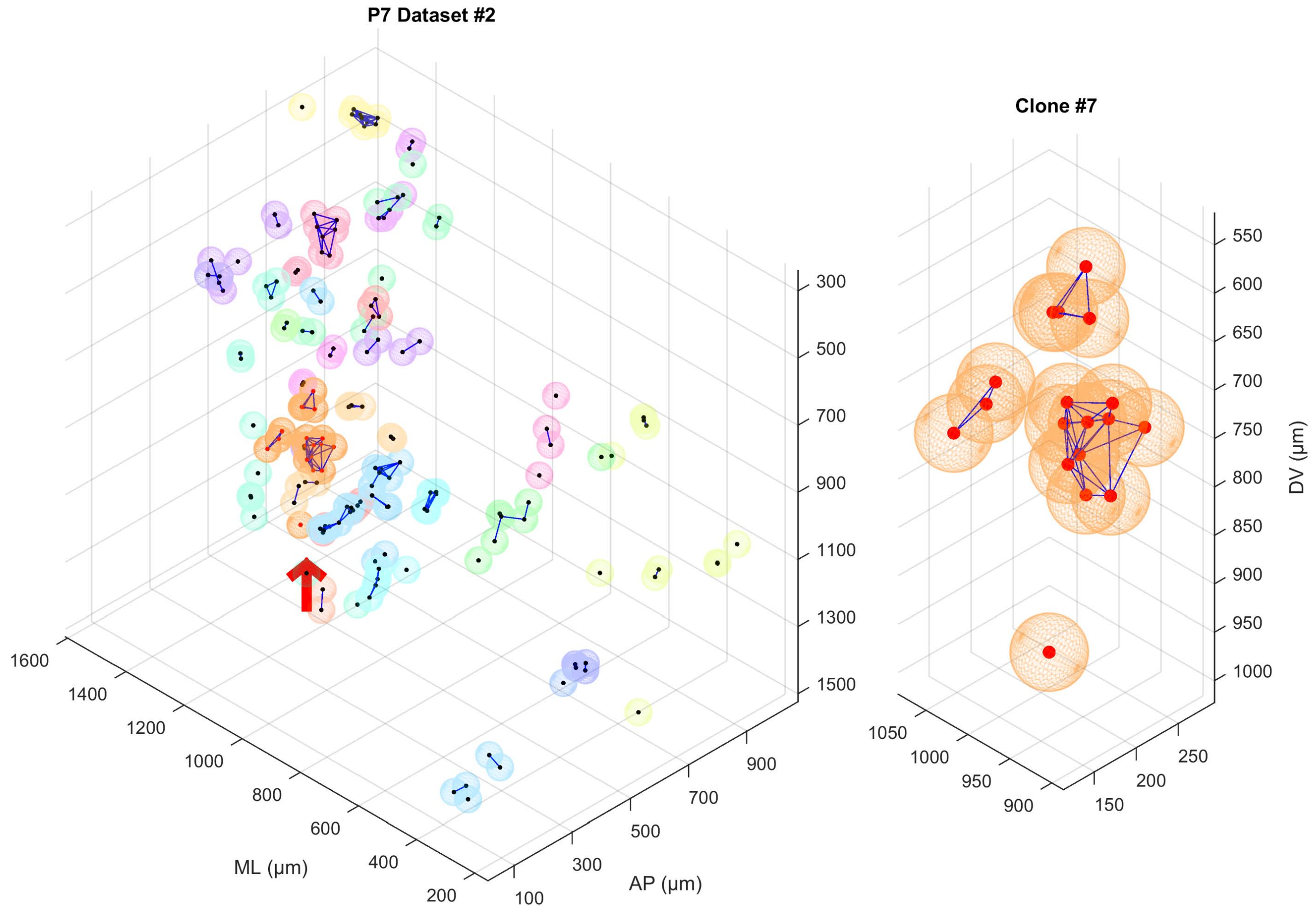
P7 Dataset #2
ML (μm)
AP (μm)
1600
1400
1200
1000
800
600
400
200
100
300
500
700
900
300
500
700
900
1100
1300
1500
Clone #7
DV (μm)
550
600
650
700
750
800
850
900
950
1000
1050
1000
950
900
150
200
250

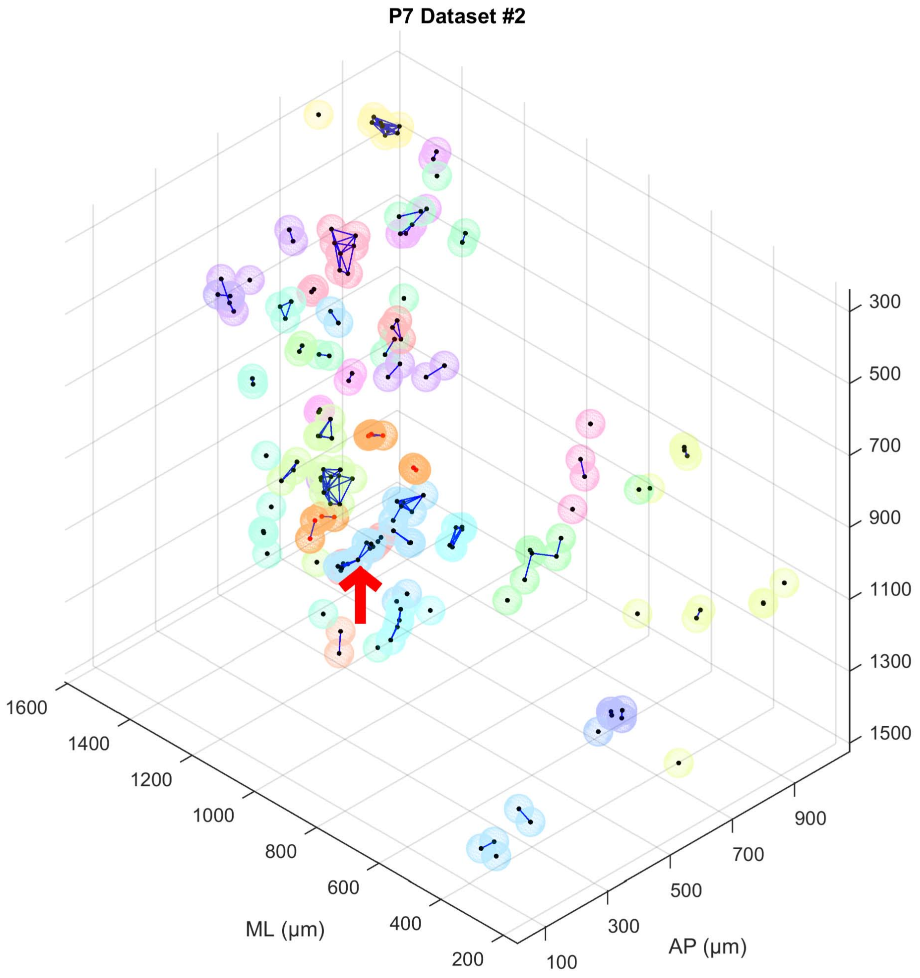
P7 Dataset #2
300
500
700
900
1100
1300
1500
1600
1400
1200
1000
800
600
400
200
ML (µm)
100
300
500
700
900
AP (µm)

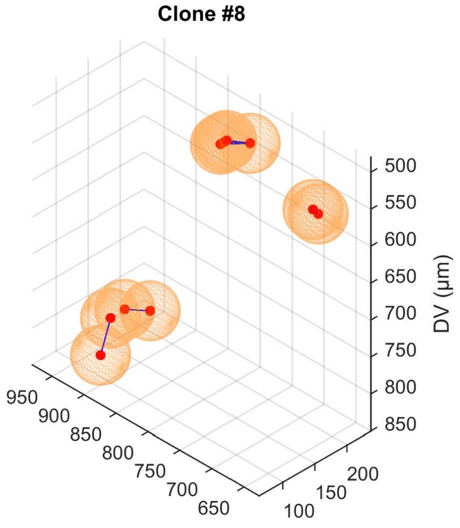
Clone #8
500
550
600
650
700
750
800
850
DV (µm)
950
900
850
800
750
700
650
100
150
200

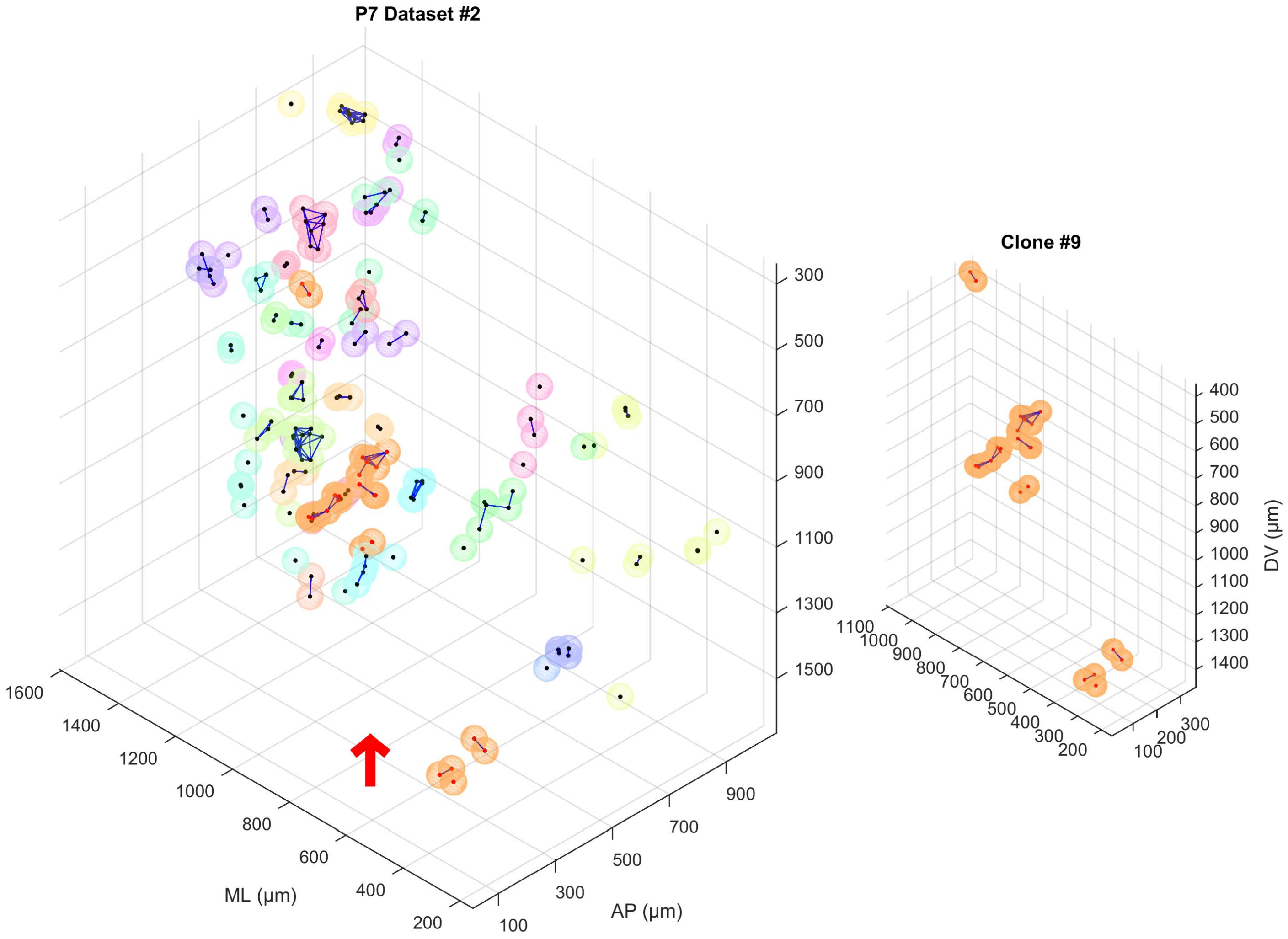

P7 Dataset #2
300
500
700
900
1100
1300
1500
1600
1400
1200
1000
800
600
400
200
ML (µm)
100
300
500
700
900
AP (µm)
Clone #9
400
500
600
700
800
900
1000
1100
1200
1300
1400
DV (µm)
1100
1000
900
800
700
600
500
400
300
200
100
200
300

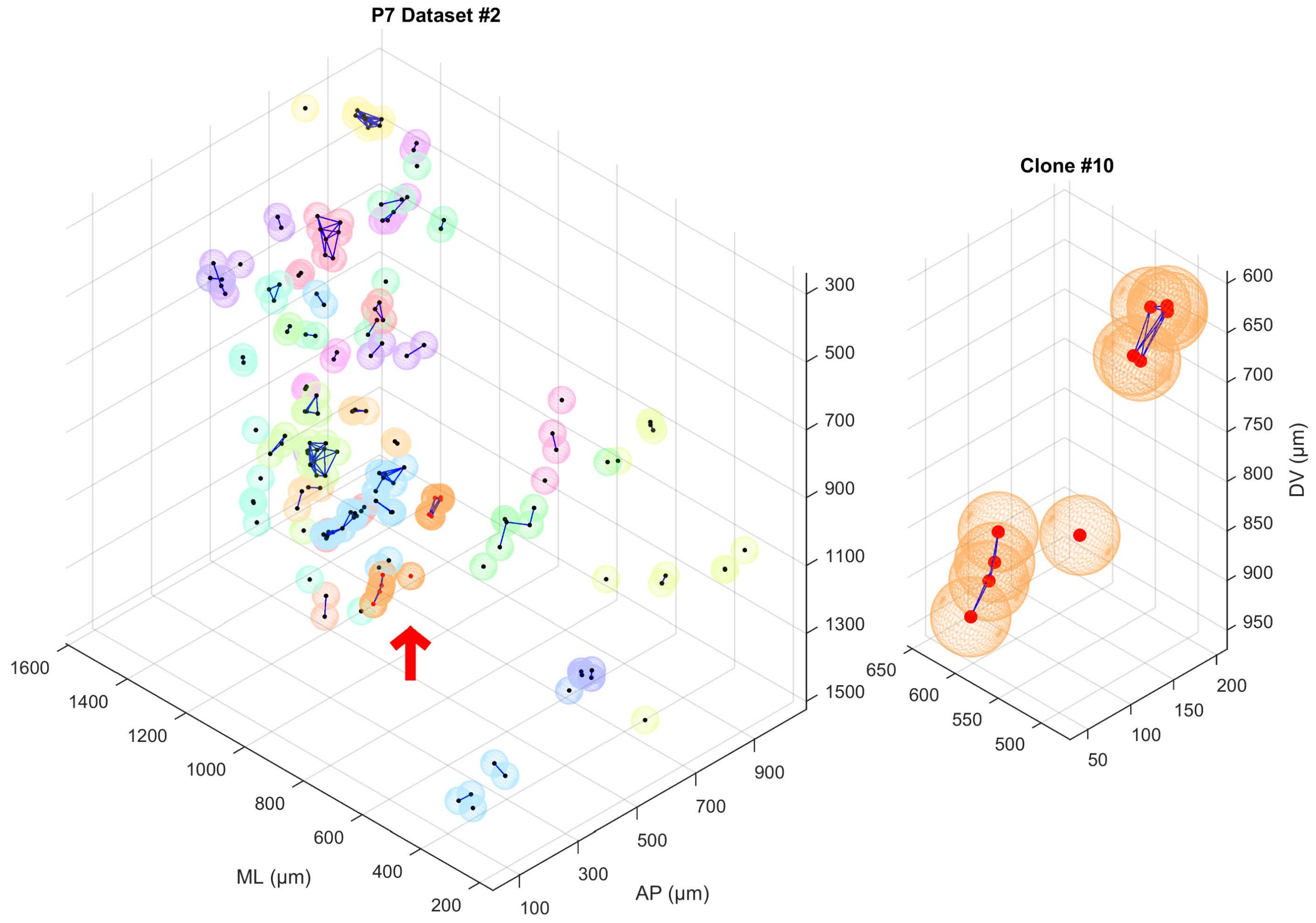

P7 Dataset #2
ML (µm)
1600
1400
1200
1000
800
600
400
200
AP (µm)
100
300
500
700
900
300
500
700
900
1100
1300
1500
Clone #10
DV (µm)
600
650
700
750
800
850
900
950
650
600
550
500
50
100
150
200

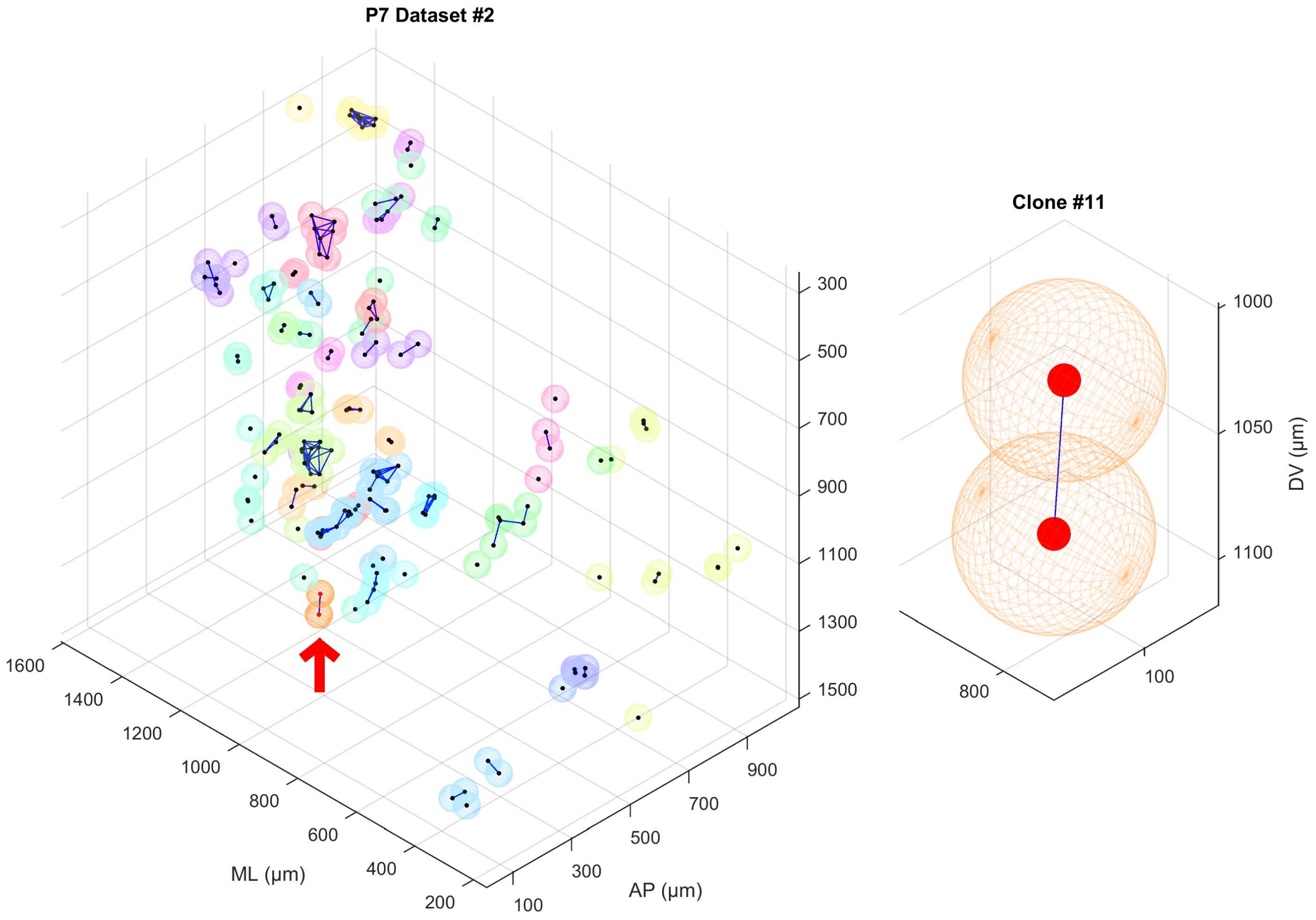

P7 Dataset #2
ML (µm)
1600
1400
1200
1000
800
600
400
200
AP (µm)
100
300
500
700
900
300
500
700
900
1100
1300
1500
Clone #11
DV (µm)
1000
1050
1100
800
100

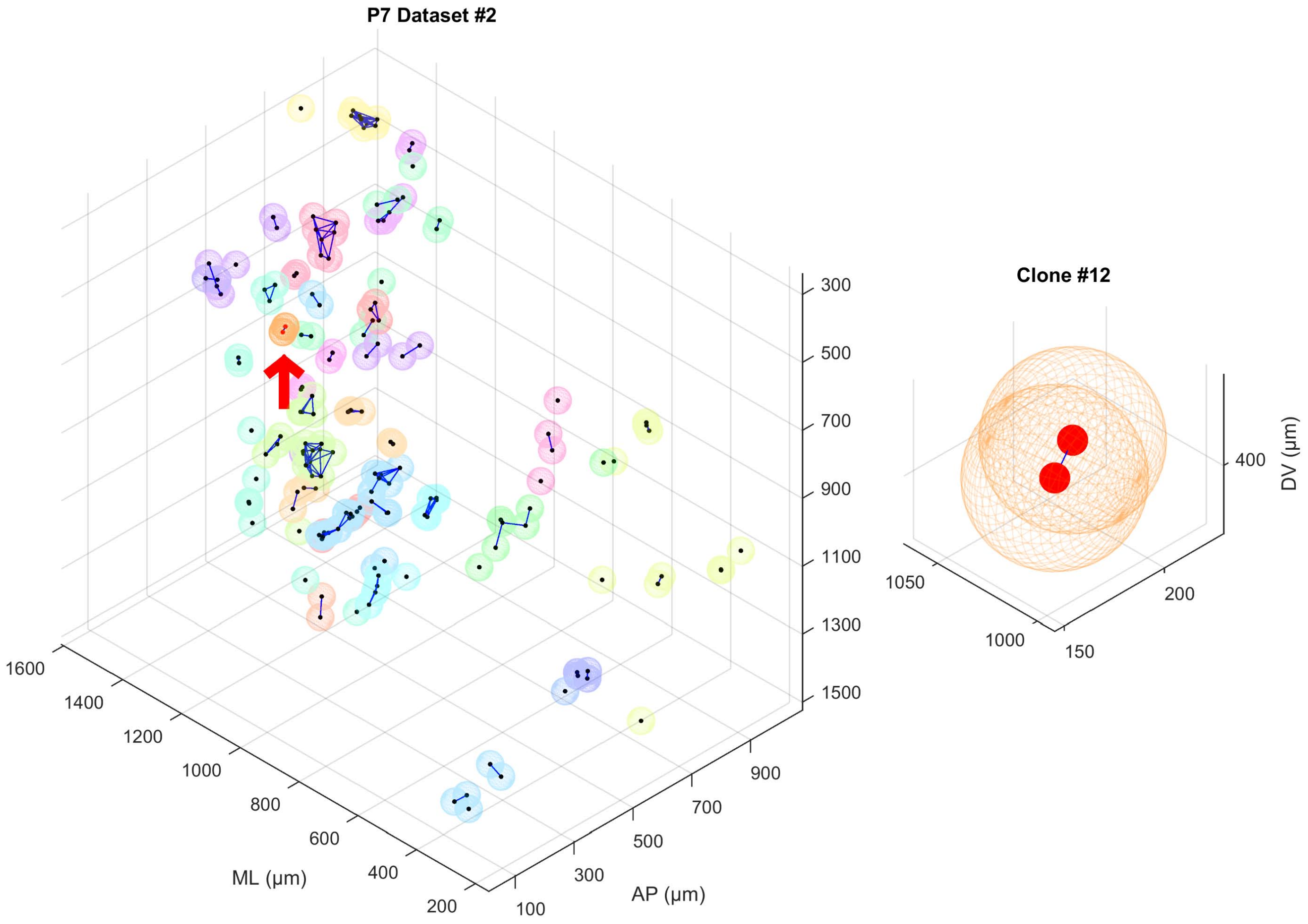

P7 Dataset #2
300
500
700
900
1100
1300
1500
1600
1400
1200
1000
800
600
400
200
ML (µm)
100
300
500
700
900
AP (µm)
Clone #12
400
DV (µm)
1050
1000
150
200

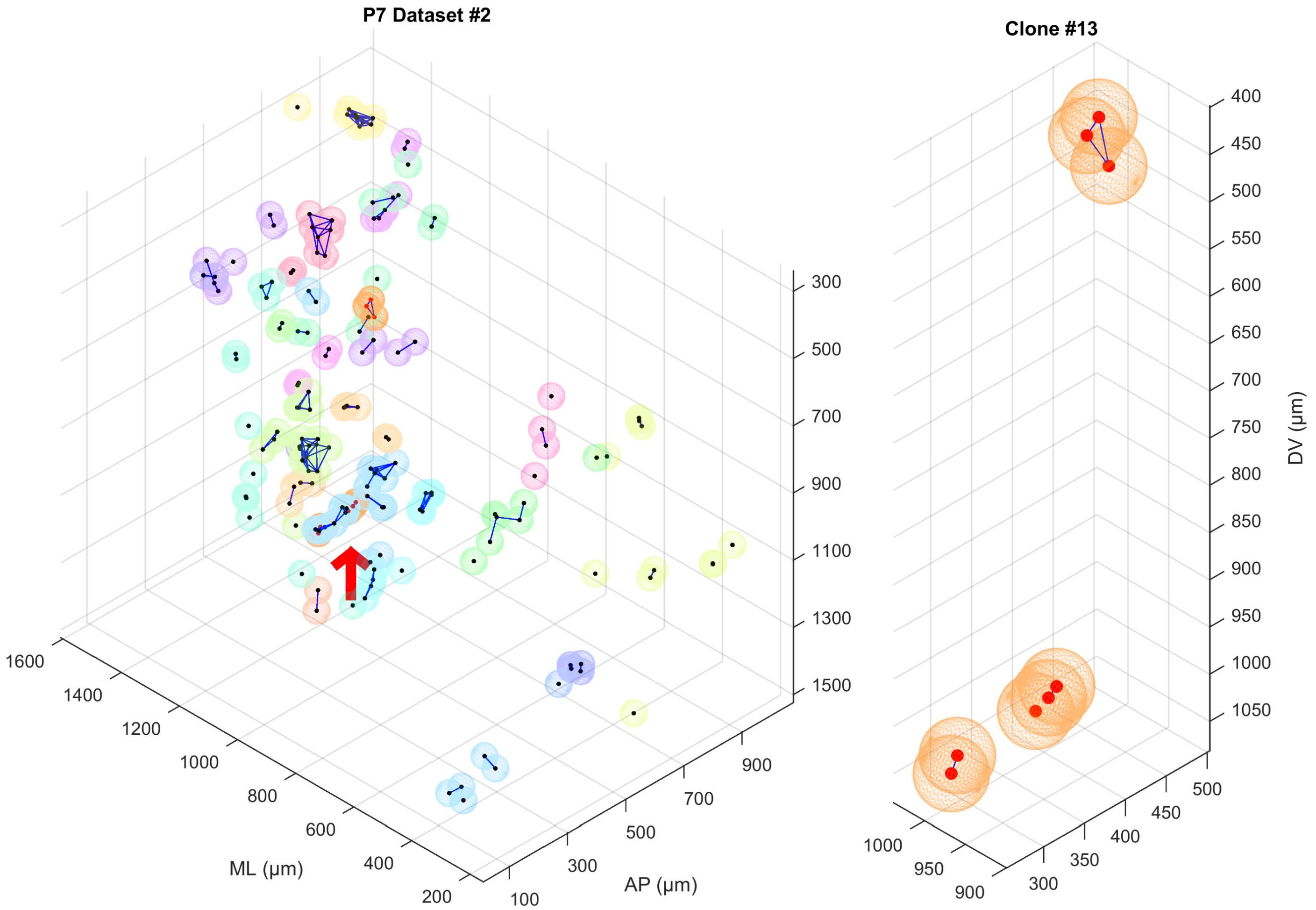

P7 Dataset #2
ML (µm)
AP (µm)
1600
1400
1200
1000
800
600
400
200
100
300
500
700
900
300
500
700
900
1100
1300
1500
Clone #13
DV (µm)
400
450
500
550
600
650
700
750
800
850
900
950
1000
1050
1000
950
900
300
350
400
450
500

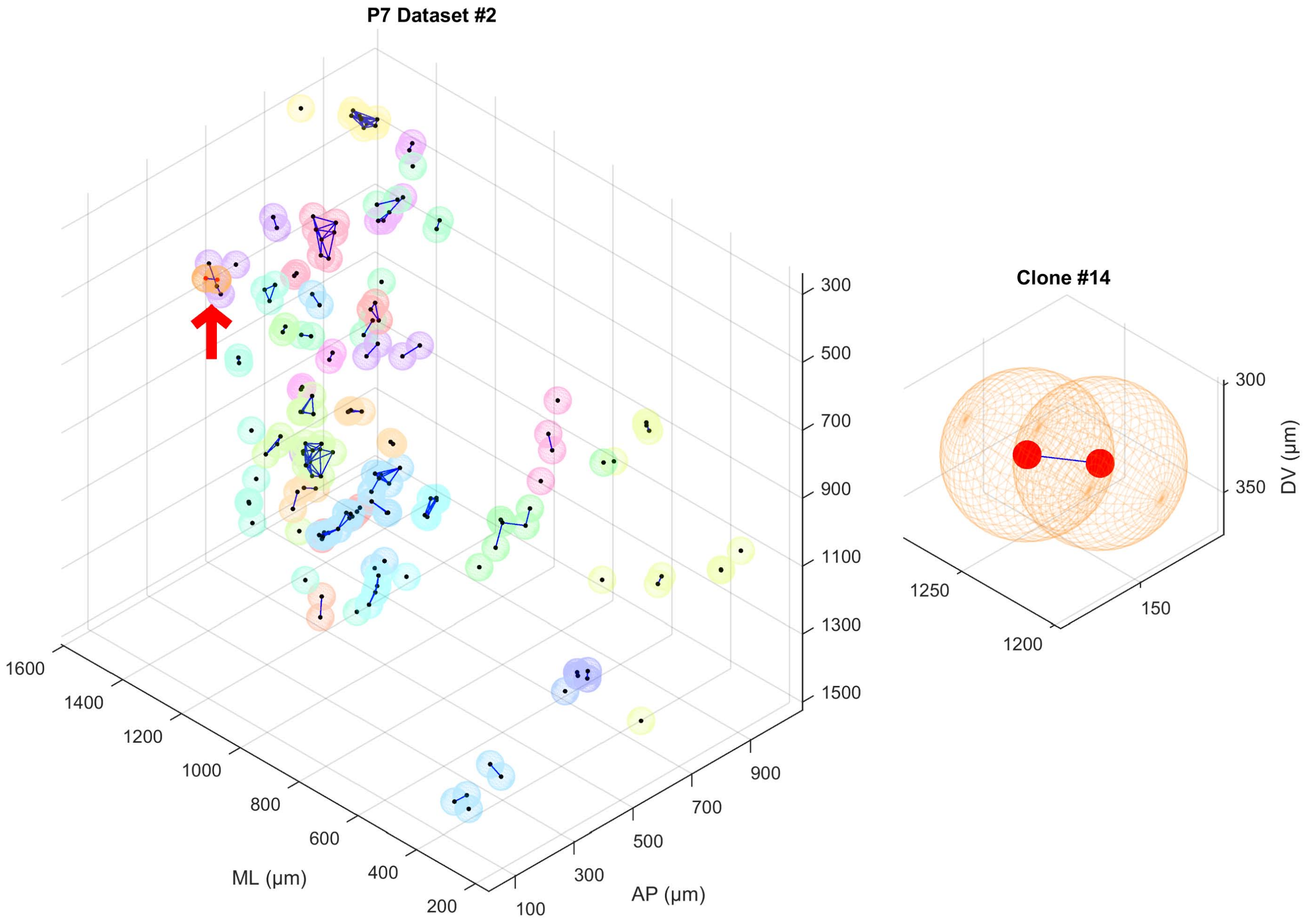

P7 Dataset #2
300
500
700
900
1100
1300
1500
1600
1400
1200
1000
800
600
400
200
ML (µm)
100
300
500
700
900
AP (µm)
Clone #14
300
350
DV (µm)
1250
1200
150

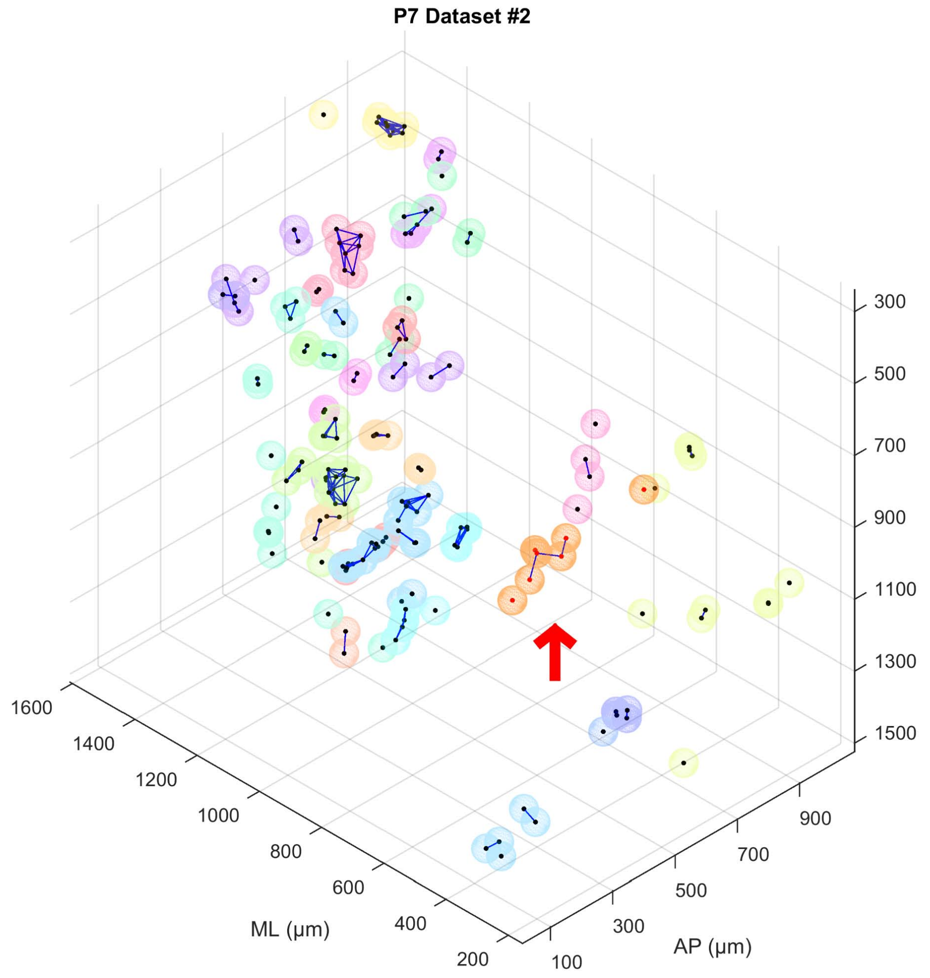

P7 Dataset #2
300
500
700
900
1100
1300
1500
1600
1400
1200
1000
800
600
400
200
ML (µm)
100
300
500
700
900
AP (µm)

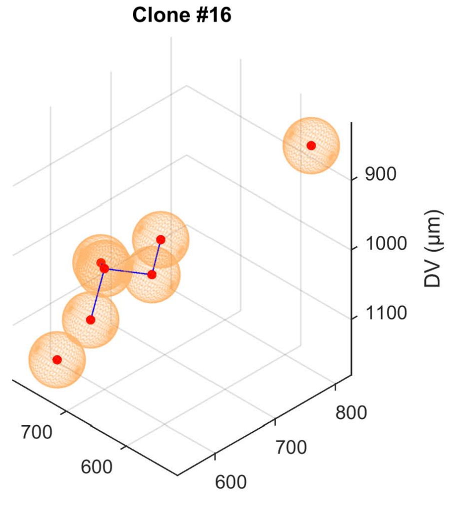

Clone #16
900
1000
1100
DV (µm)
700
600
800
700
600

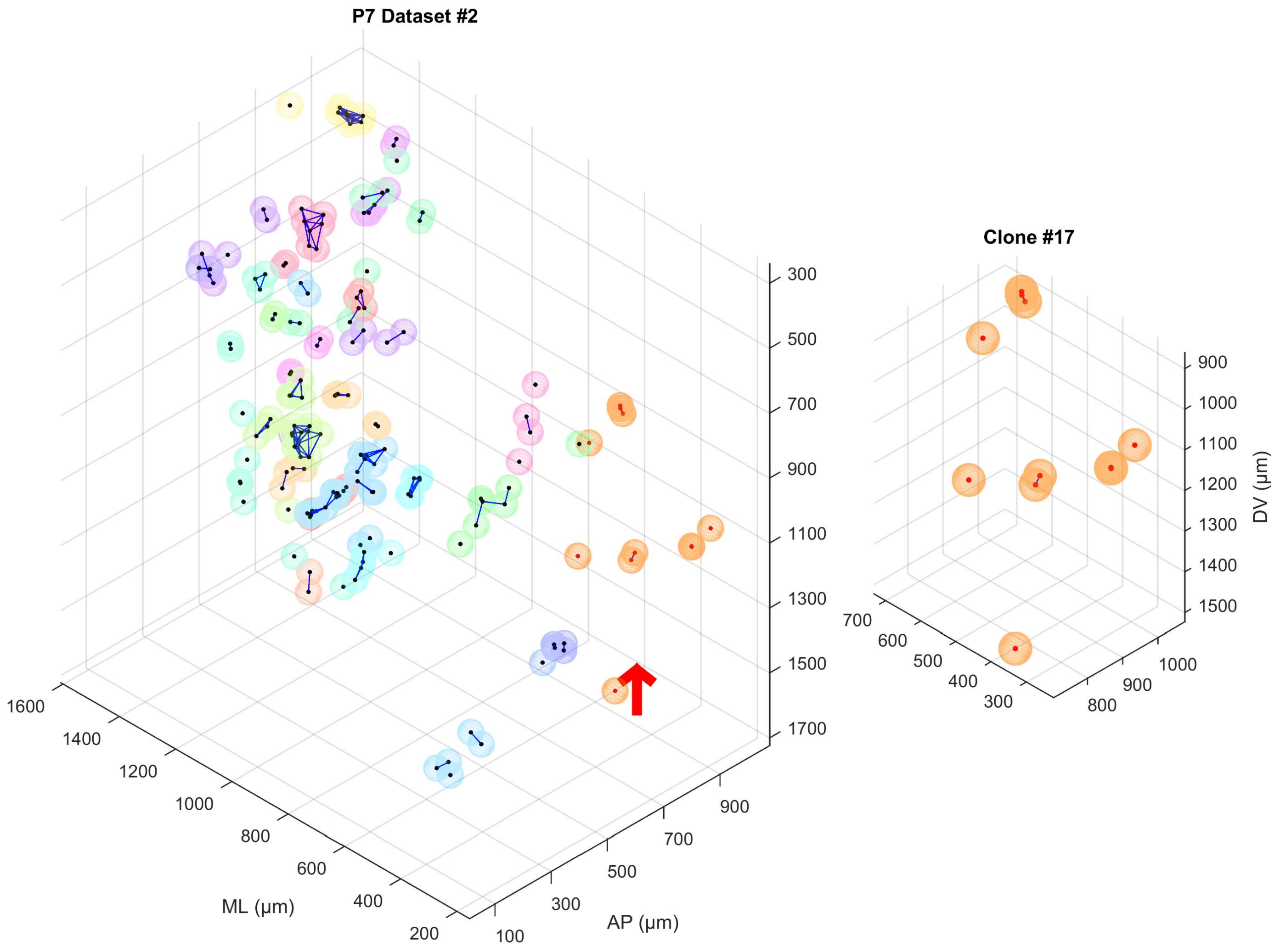
P7 Dataset #2
ML (µm)
1600
1400
1200
1000
800
600
400
200
AP (µm)
100
300
500
700
900
300
500
700
900
1100
1300
1500
1700
Clone #17
DV (µm)
900
1000
1100
1200
1300
1400
1500
700
600
500
400
300
800
900
1000

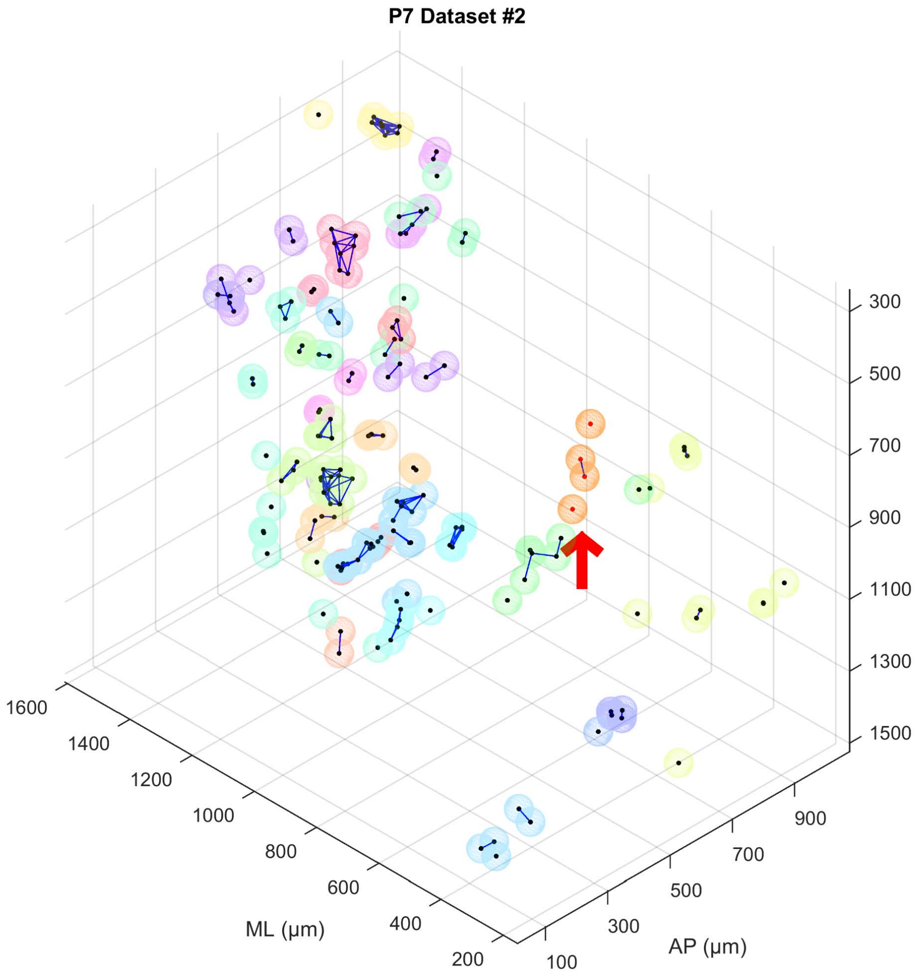
P7 Dataset #2
300
500
700
900
1100
1300
1500
1600
1400
1200
1000
800
600
400
200
ML (µm)
100
300
500
700
900
AP (µm)

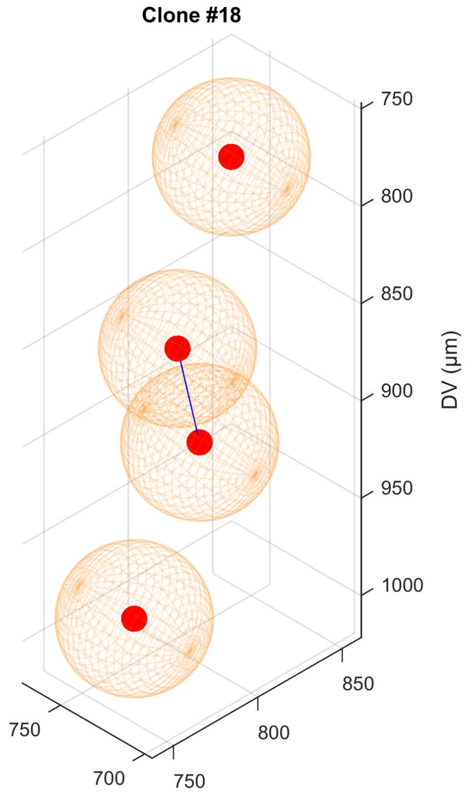
Clone #18
750
800
850
900
950
1000
DV (µm)
750
700
750
800
850

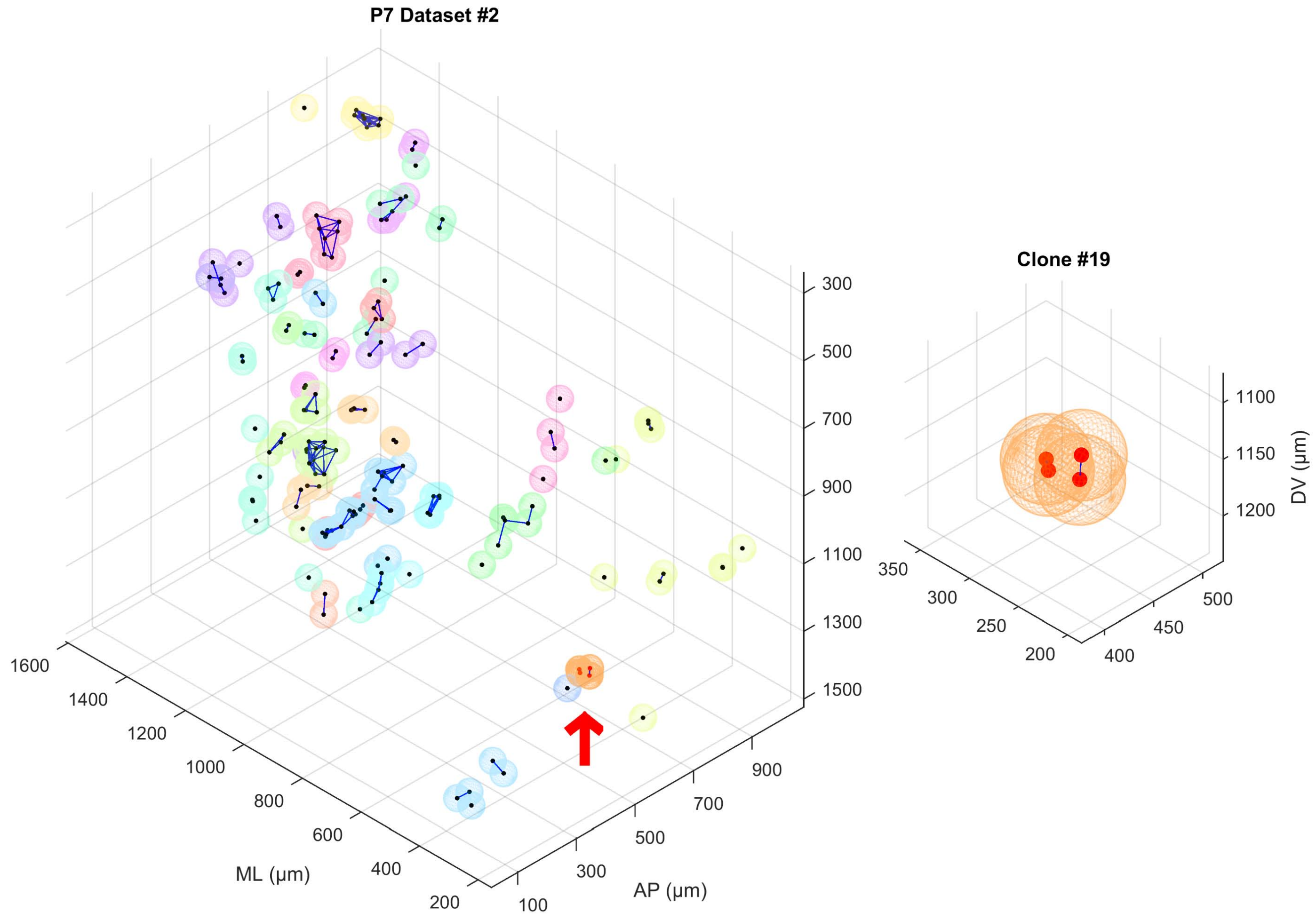

P7 Dataset #2
300
500
700
900
1100
1300
1500
1600
1400
1200
1000
800
600
400
200
ML (µm)
100
300
500
700
900
AP (µm)
Clone #19
1100
1150
1200
DV (µm)
350
300
250
200
400
450
500

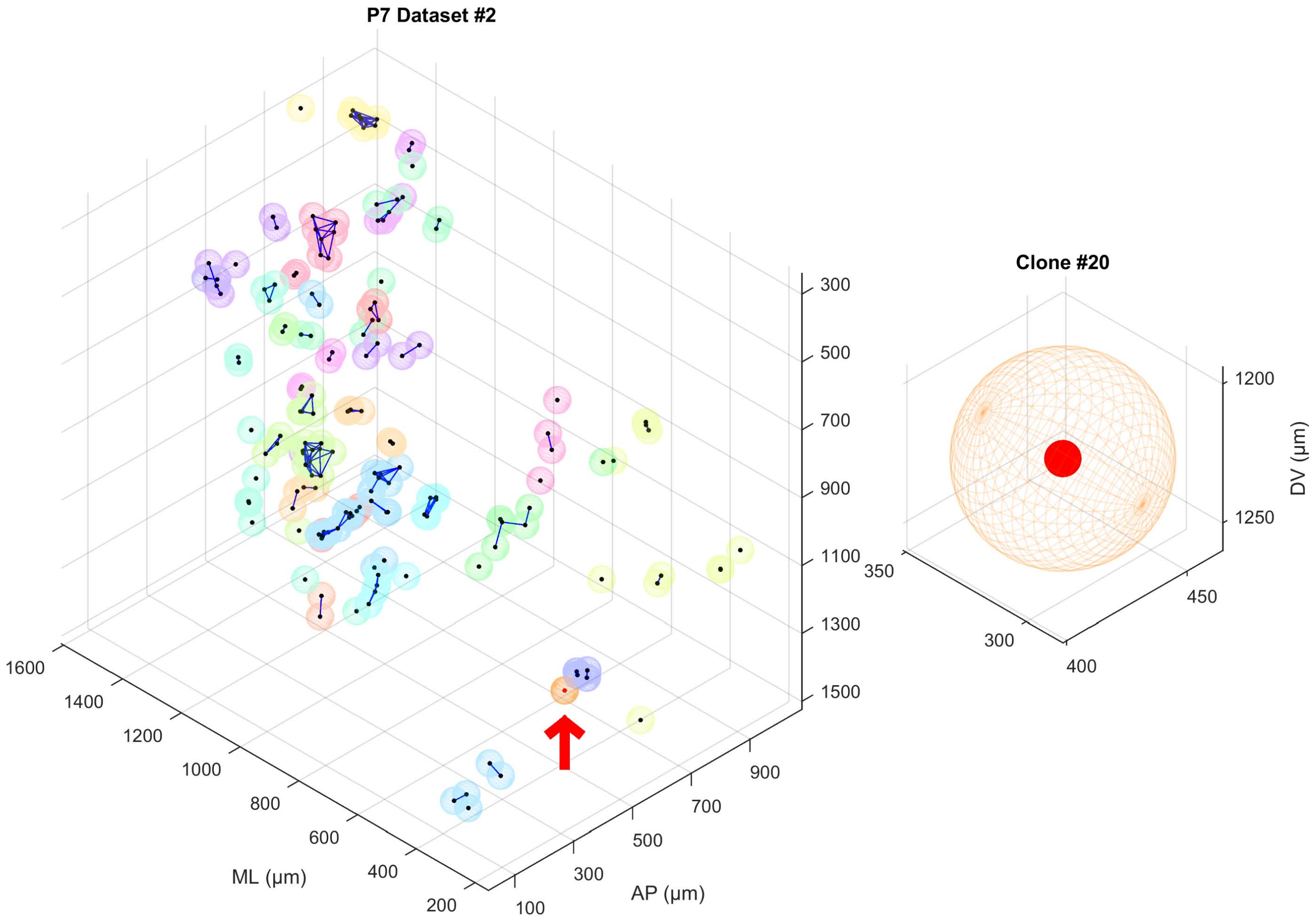
P7 Dataset #2
300
500
700
900
1100
1300
1500
1600
1400
1200
1000
800
600
400
200
ML (µm)
100
300
500
700
900
AP (µm)
Clone #20
1200
1250
DV (µm)
350
300
400
450

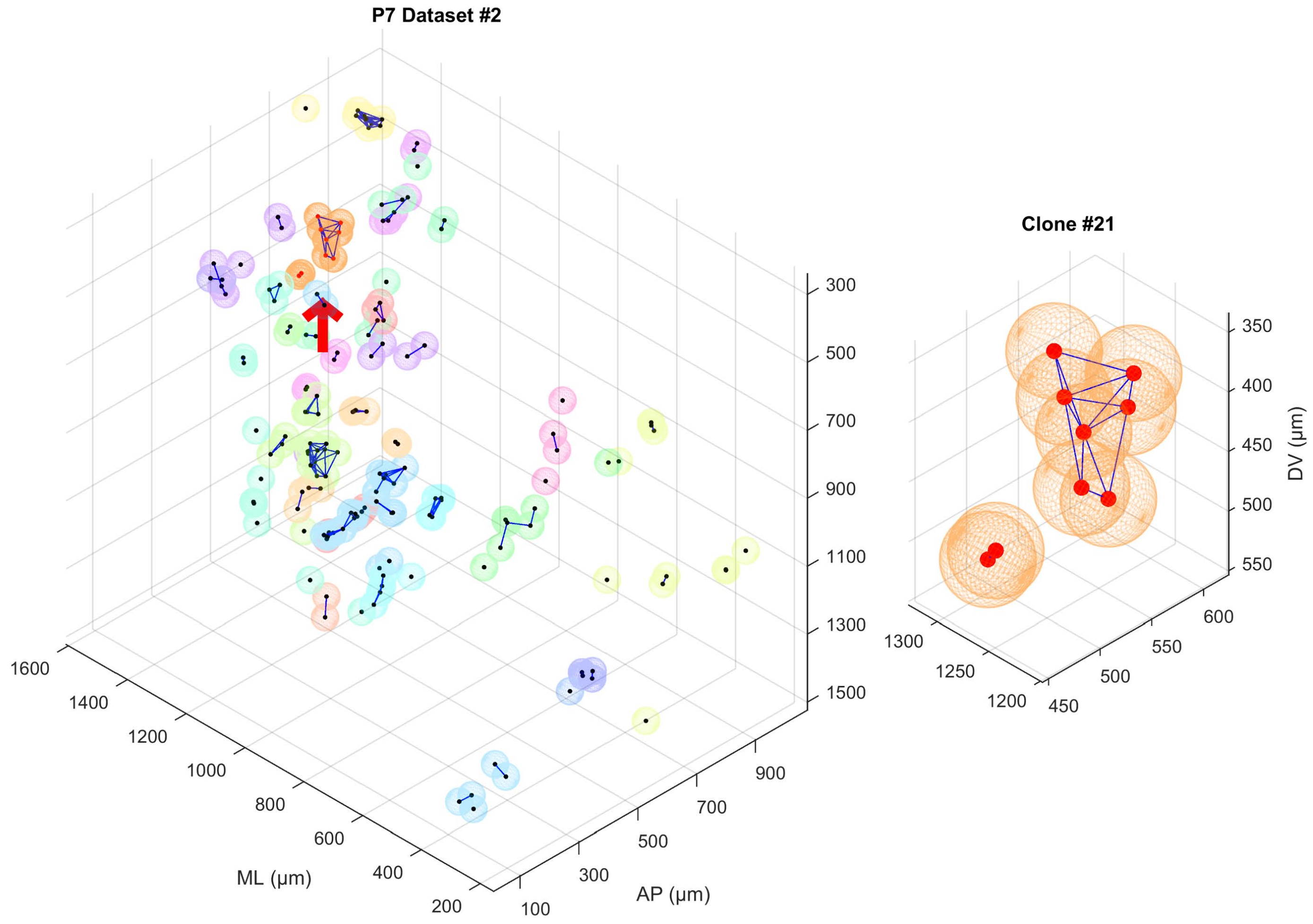

P7 Dataset #2
ML (μm)
AP (μm)
1600
1400
1200
1000
800
600
400
200
100
300
500
700
900
300
500
700
900
1100
1300
1500
Clone #21
DV (μm)
350
400
450
500
550
1300
1250
1200
450
500
550
600

P21 Clones

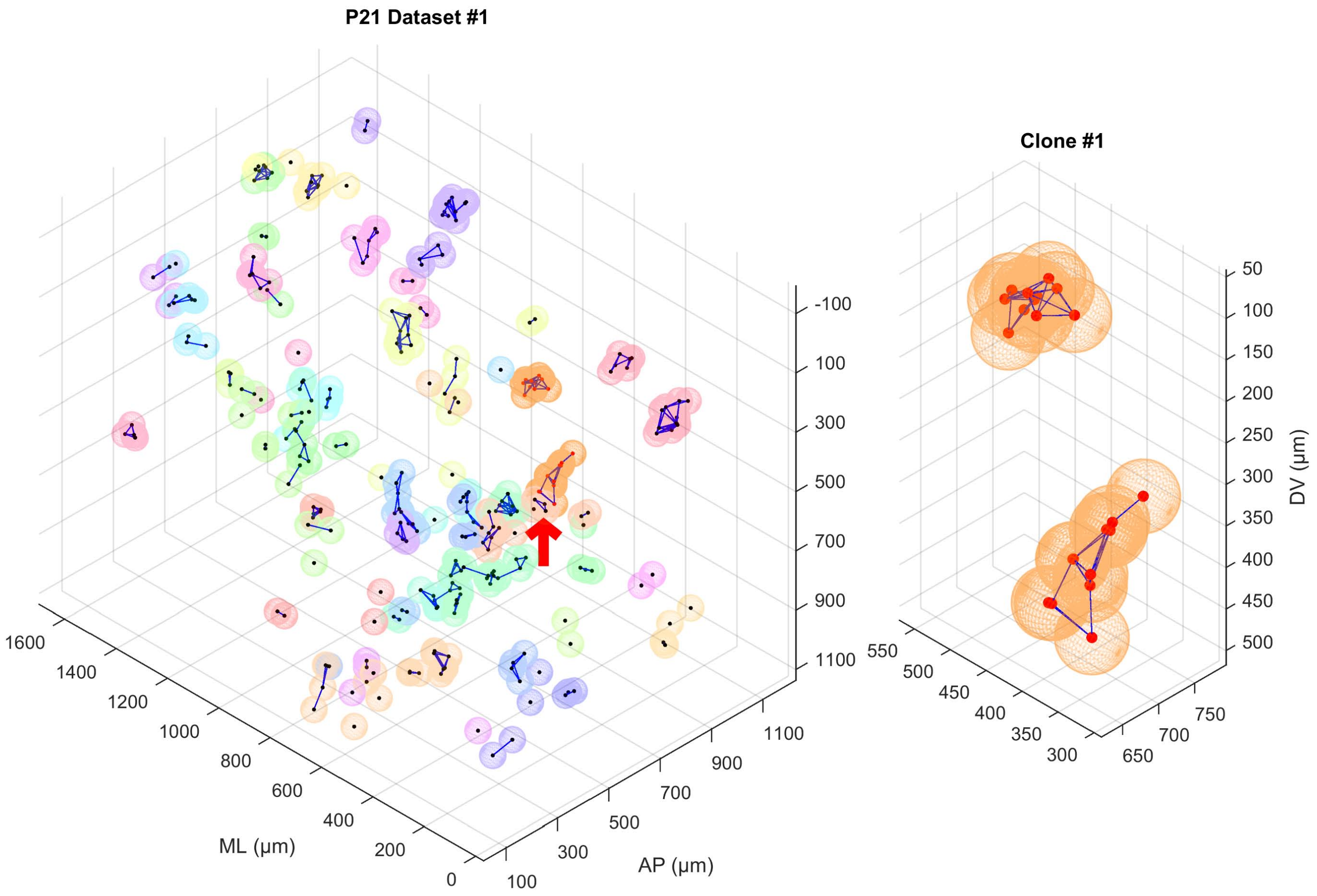

P21 Dataset #1
-100
100
300
500
700
900
1100
1600
1400
1200
1000
800
600
400
200
0
ML (µm)
100
300
500
700
900
1100
AP (µm)
Clone #1
50
100
150
200
250
300
350
400
450
500
DV (µm)
550
500
450
400
350
300
650
700
750

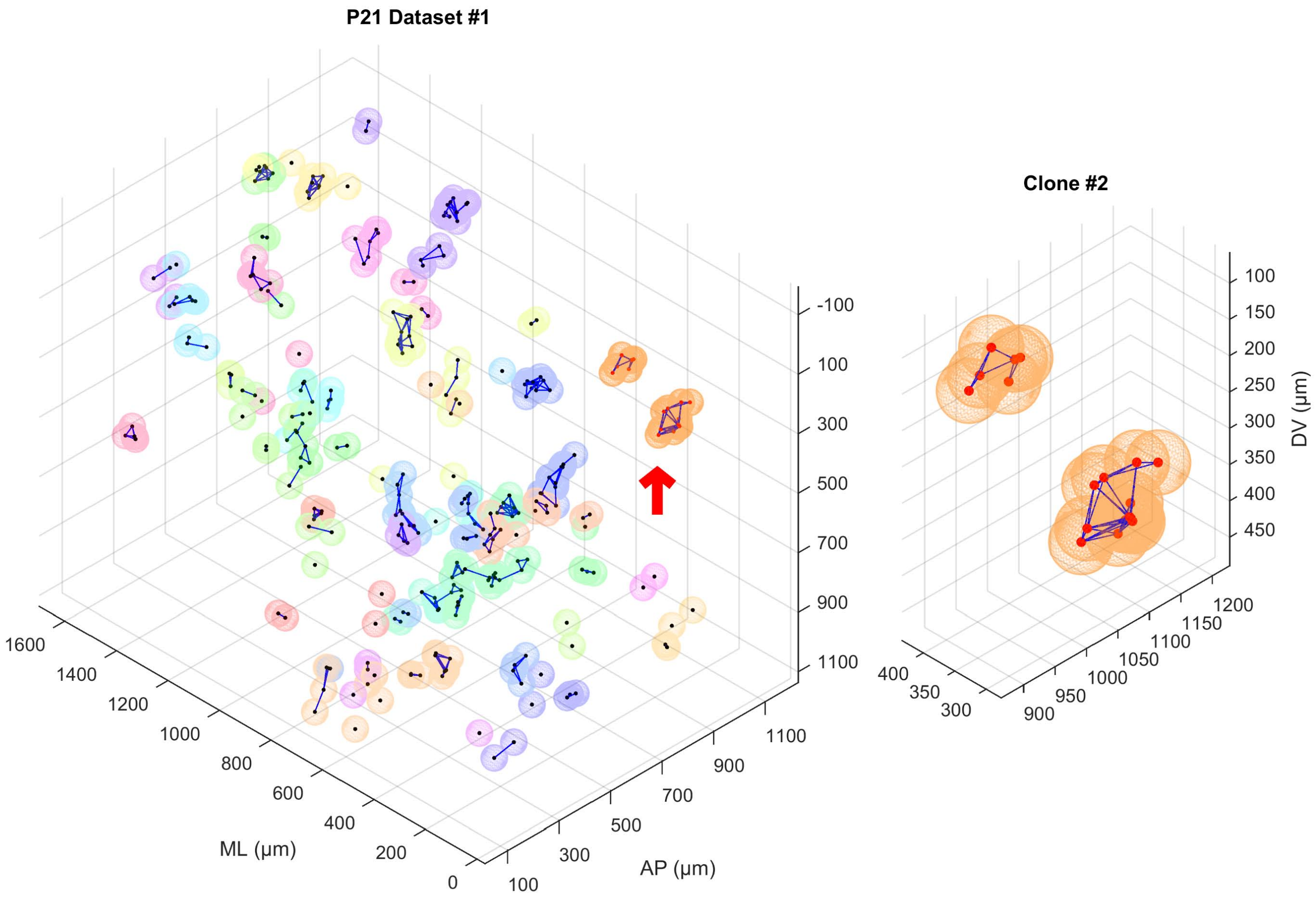
P21 Dataset #1
ML (µm)
1600
1400
1200
1000
800
600
400
200
0
AP (µm)
100
300
500
700
900
1100
-100
100
300
500
700
900
1100
Clone #2
DV (µm)
100
150
200
250
300
350
400
450
400
350
300
900
950
1000
1050
1100
1150
1200

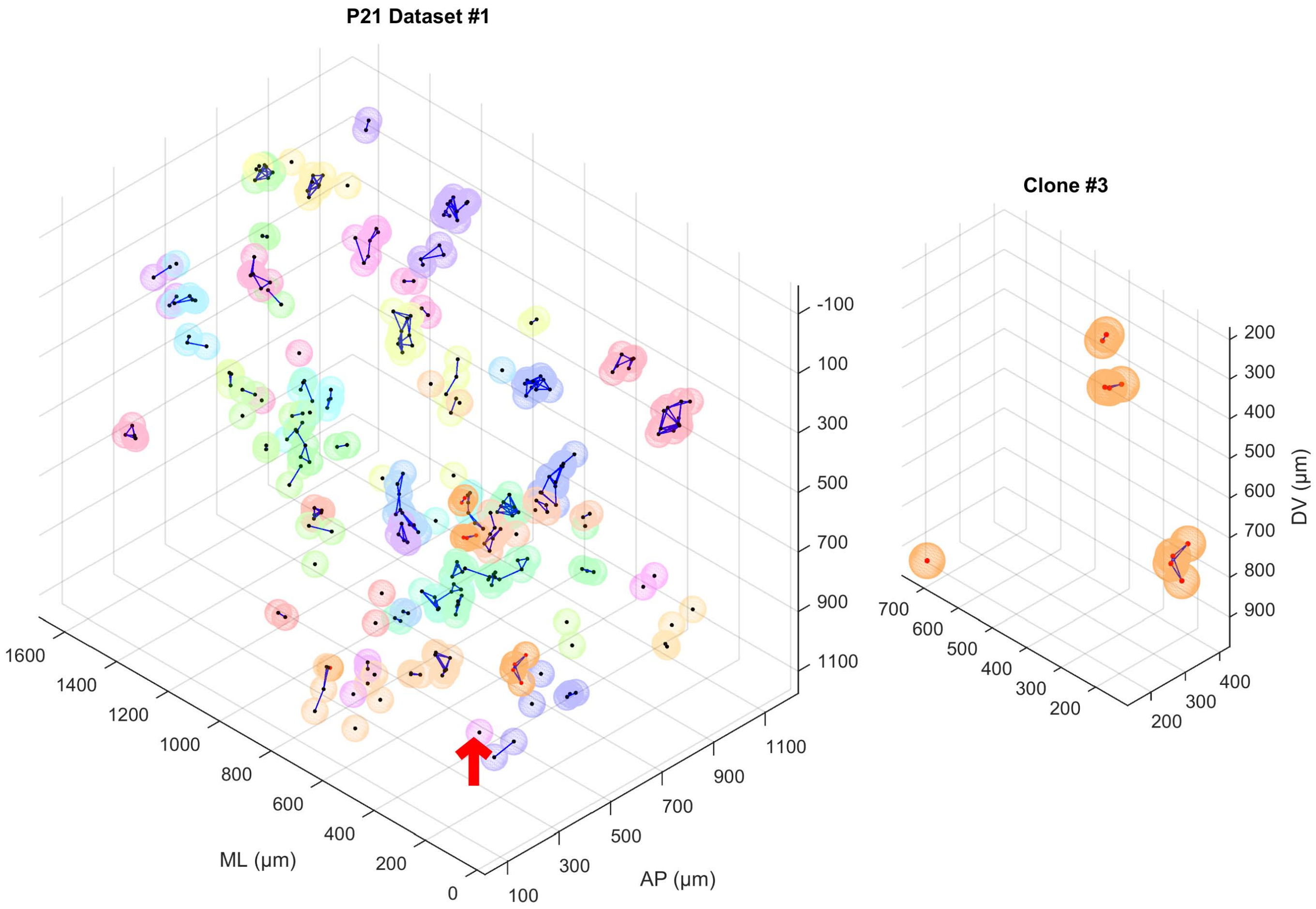
P21 Dataset #1
-100
100
300
500
700
900
1100
1600
1400
1200
1000
800
600
400
200
0
ML (µm)
100
300
500
700
900
1100
AP (µm)
Clone #3
200
300
400
500
600
700
800
900
DV (µm)
700
600
500
400
300
200
200
300
400

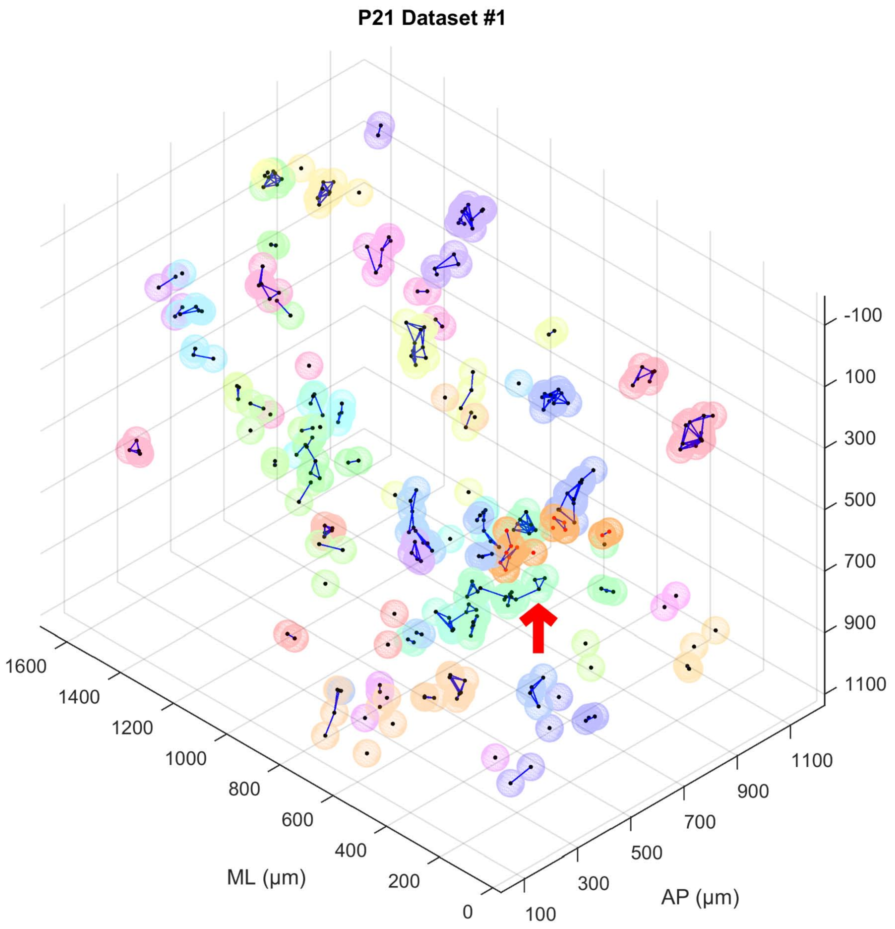
P21 Dataset #1
-100
100
300
500
700
900
1100
1600
1400
1200
1000
800
600
400
200
0
ML (µm)
100
300
500
700
900
1100
AP (µm)

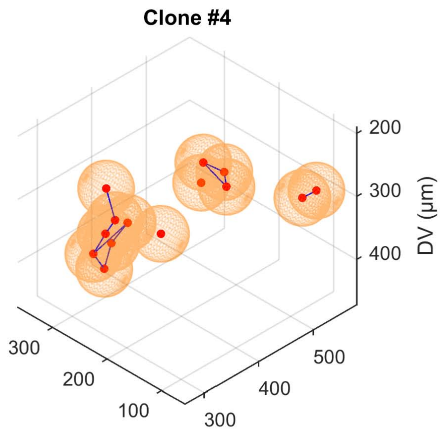
Clone #4
200
300
400
DV (µm)
300
200
100
300
400
500

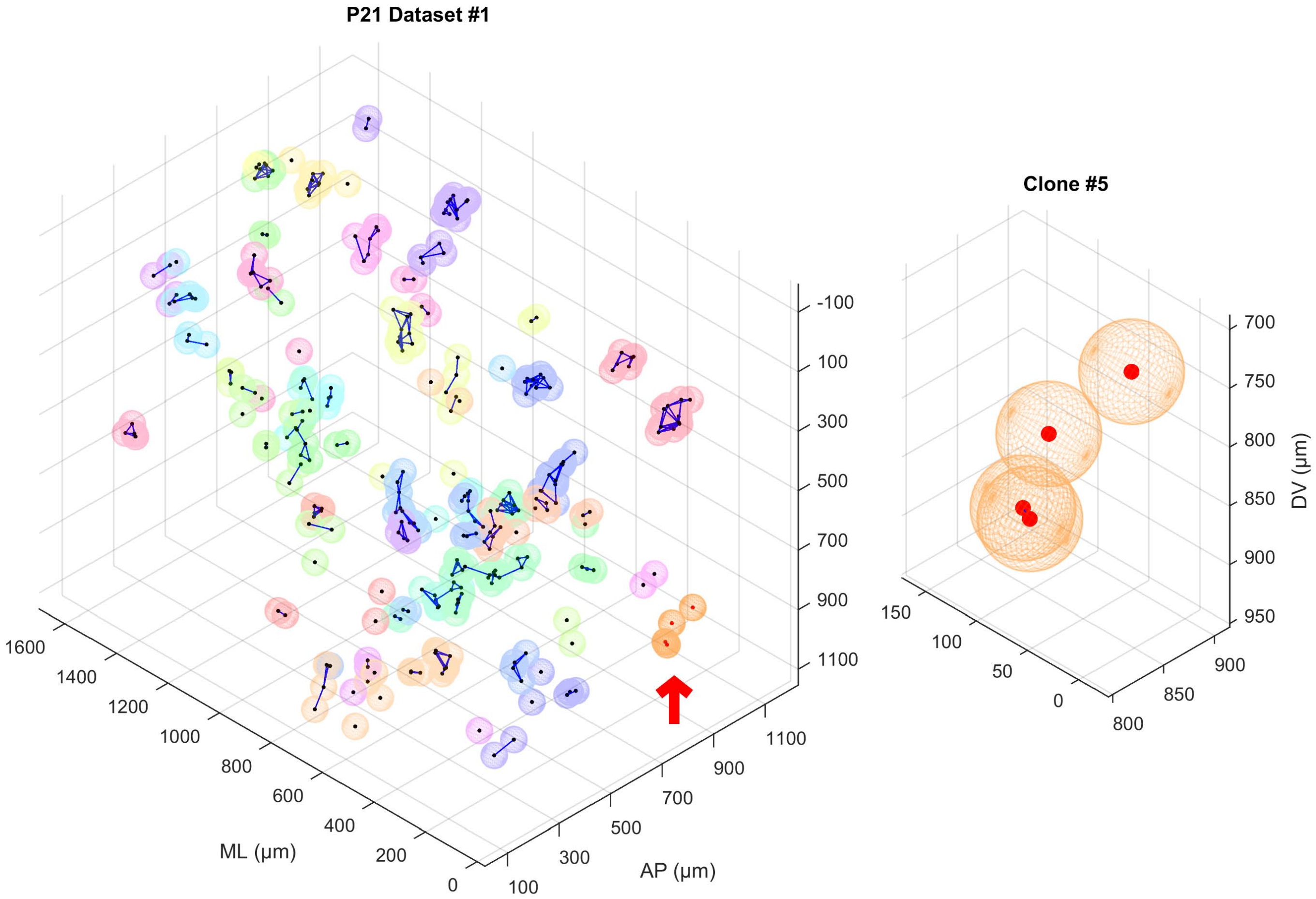
P21 Dataset #1
Clone #5
ML (μm)
AP (μm)
DV (μm)
1600
1400
1200
1000
800
600
400
200
0
100
300
500
700
900
1100
-100
100
300
500
700
900
1100
150
100
50
0
800
850
900
700
750
800
850
900
950

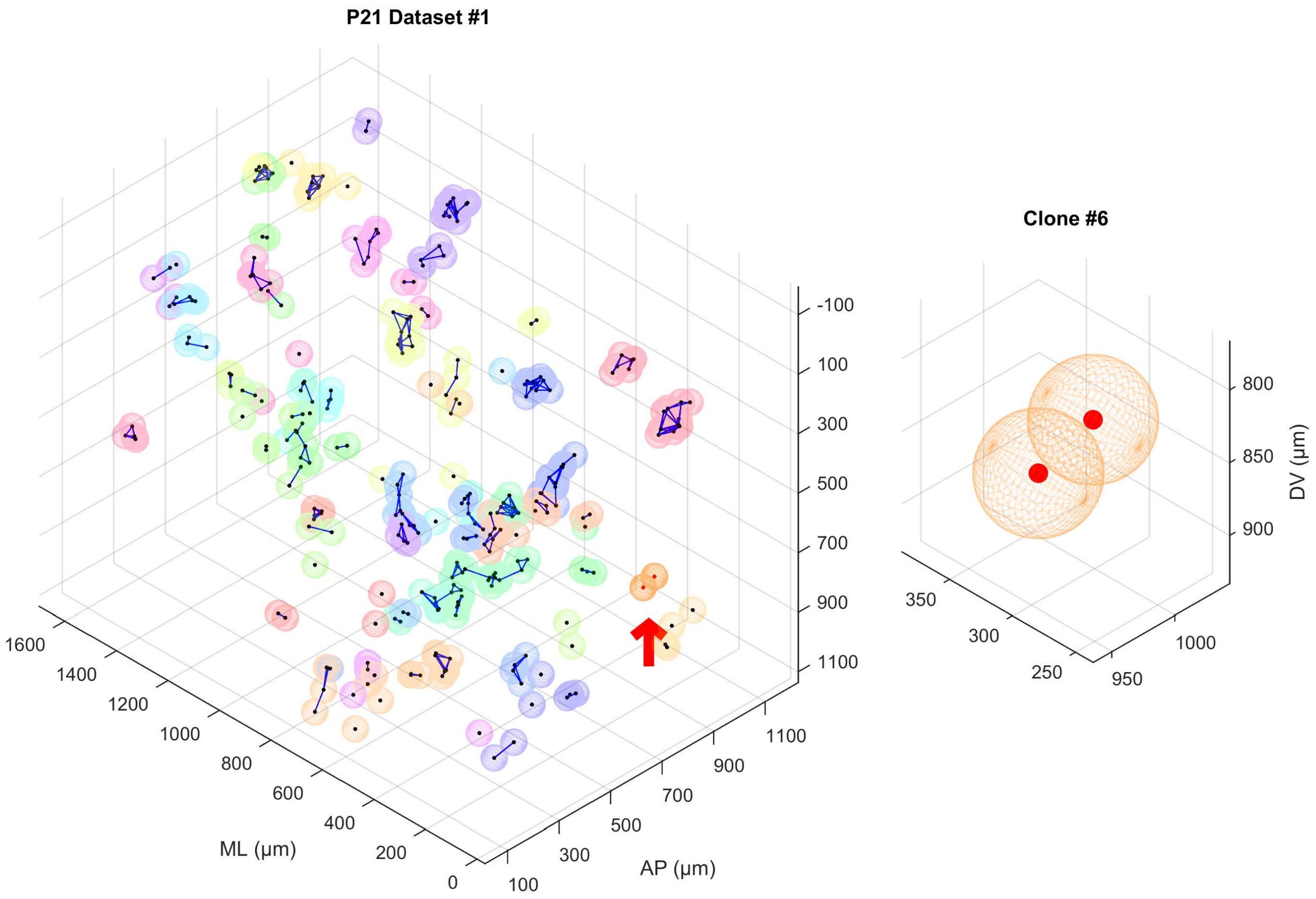
P21 Dataset #1
Clone #6
-100
100
300
500
700
900
1100
1600
1400
1200
1000
800
600
400
200
0
ML (µm)
100
300
500
700
900
1100
AP (µm)
800
850
900
DV (µm)
350
300
250
950
1000

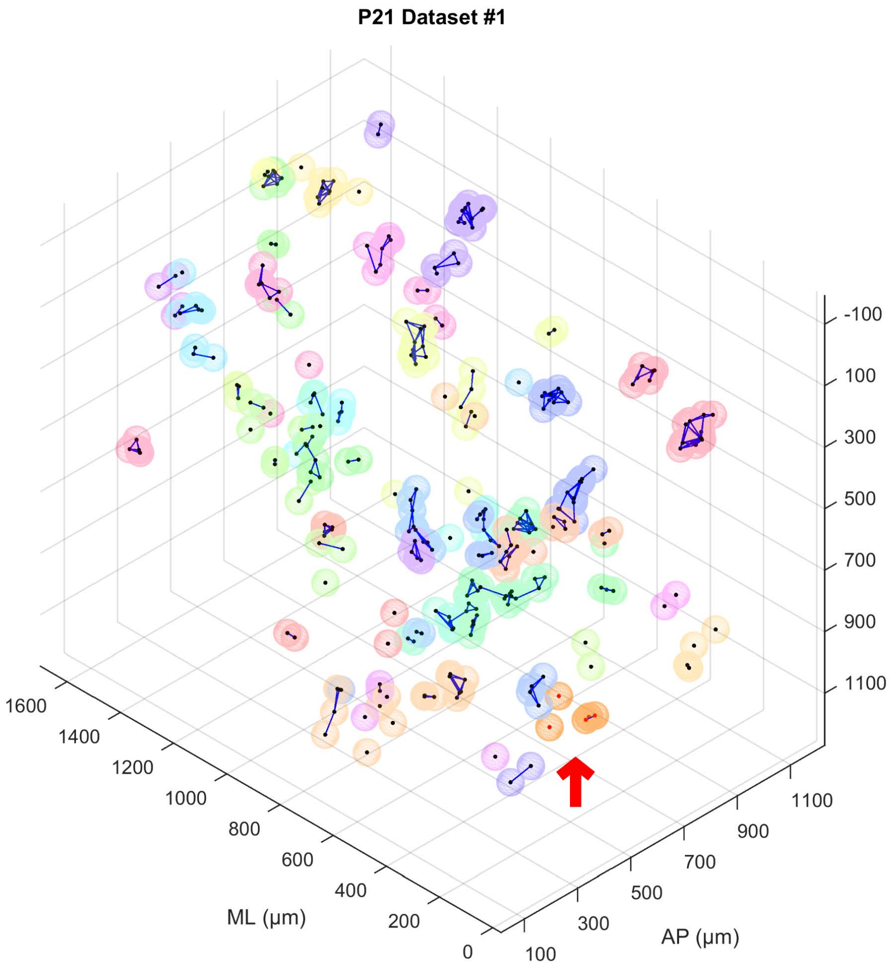
P21 Dataset #1
-100
100
300
500
700
900
1100
1600
1400
1200
1000
800
600
400
200
0
ML (µm)
100
300
500
700
900
1100
AP (µm)

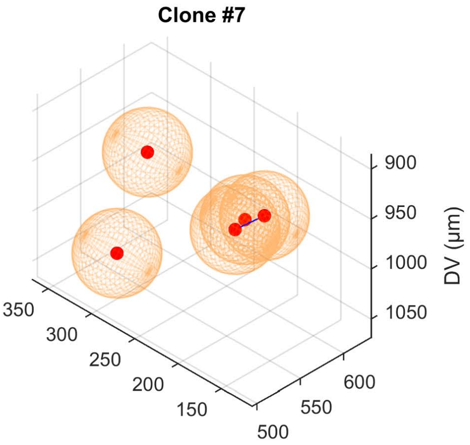
Clone #7
900
950
1000
1050
DV (µm)
350
300
250
200
150
500
550
600

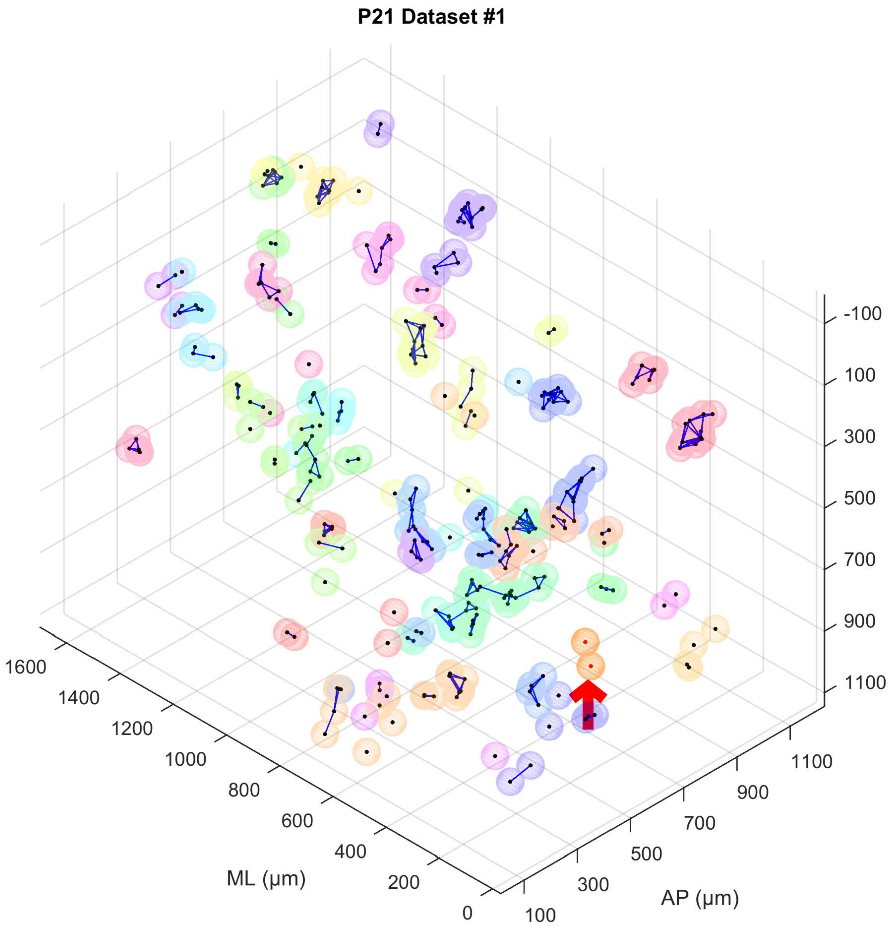

P21 Dataset #1
-100
100
300
500
700
900
1100
1600
1400
1200
1000
800
600
400
200
0
ML (µm)
100
300
500
700
900
1100
AP (µm)

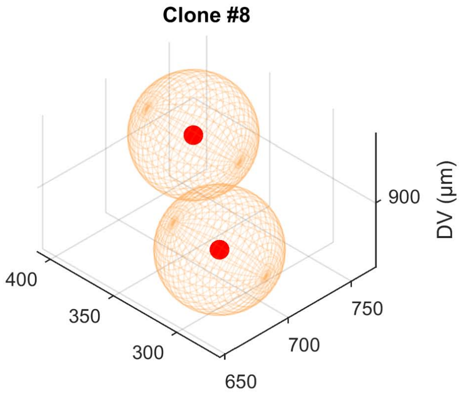

Clone #8
900
DV (µm)
400
350
300
650
700
750

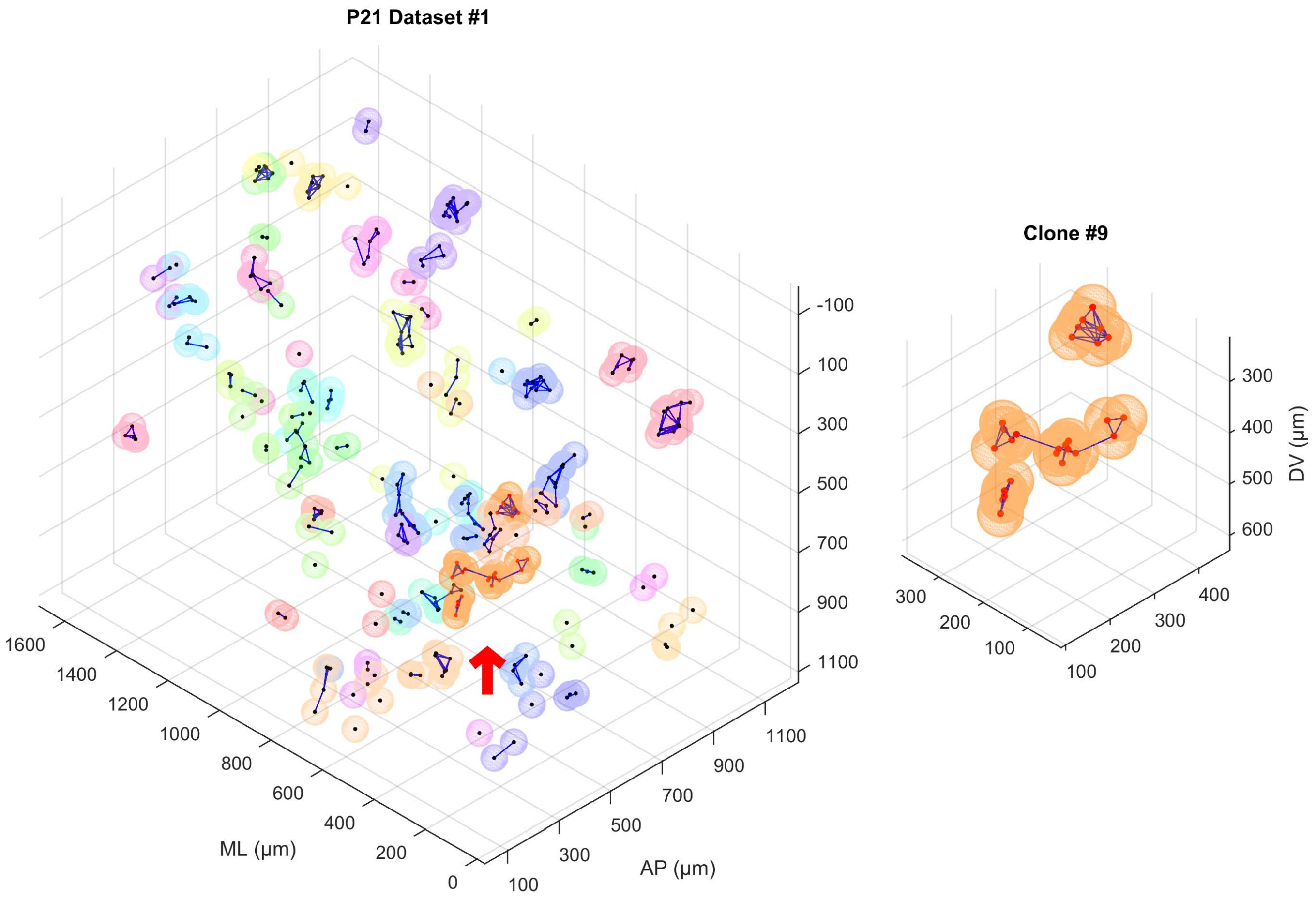

P21 Dataset #1
Clone #9
ML (µm)
AP (µm)
DV (µm)
-100
100
300
500
700
900
1100
1600
1400
1200
1000
800
600
400
200
0
100
300
500
700
900
1100
300
400
500
600
300
200
100
100
200
300
400

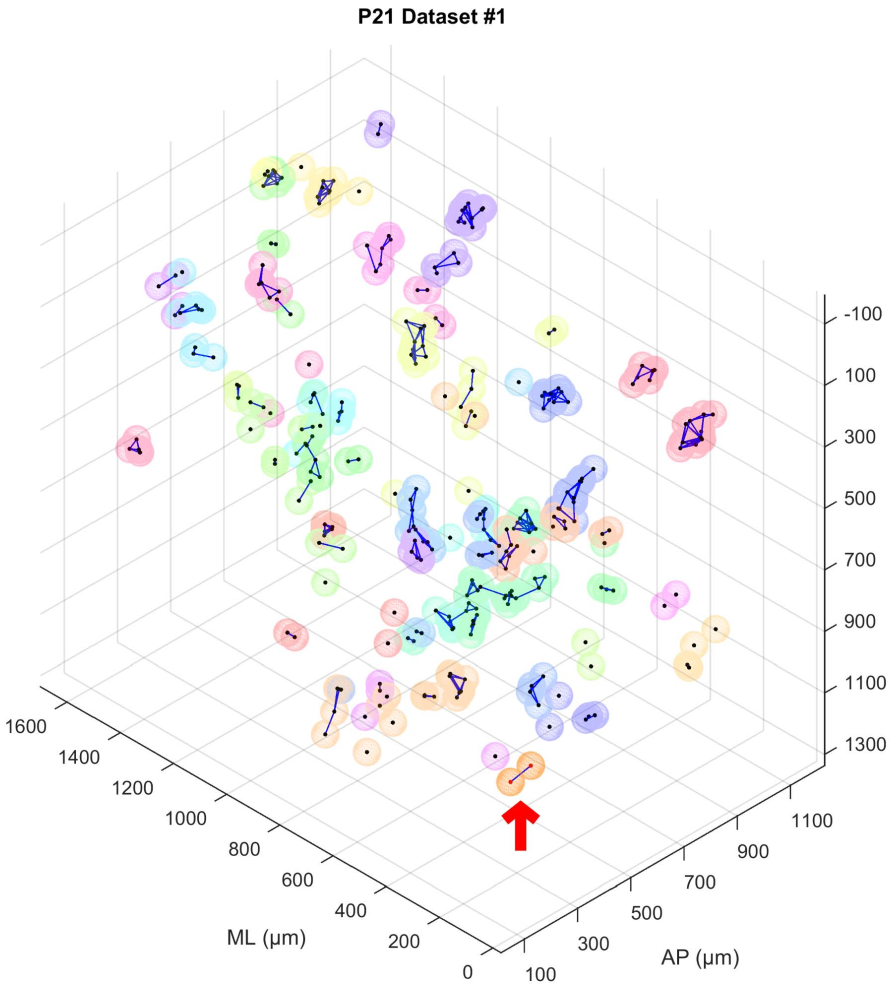

P21 Dataset #1
-100
100
300
500
700
900
1100
1300
1600
1400
1200
1000
800
600
400
200
0
ML (µm)
100
300
500
700
900
1100
AP (µm)

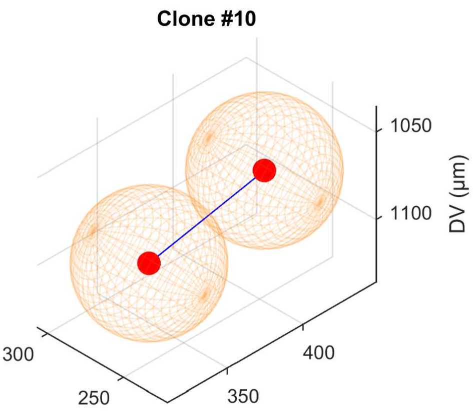

Clone #10
1050
1100
DV (µm)
300
250
350
400

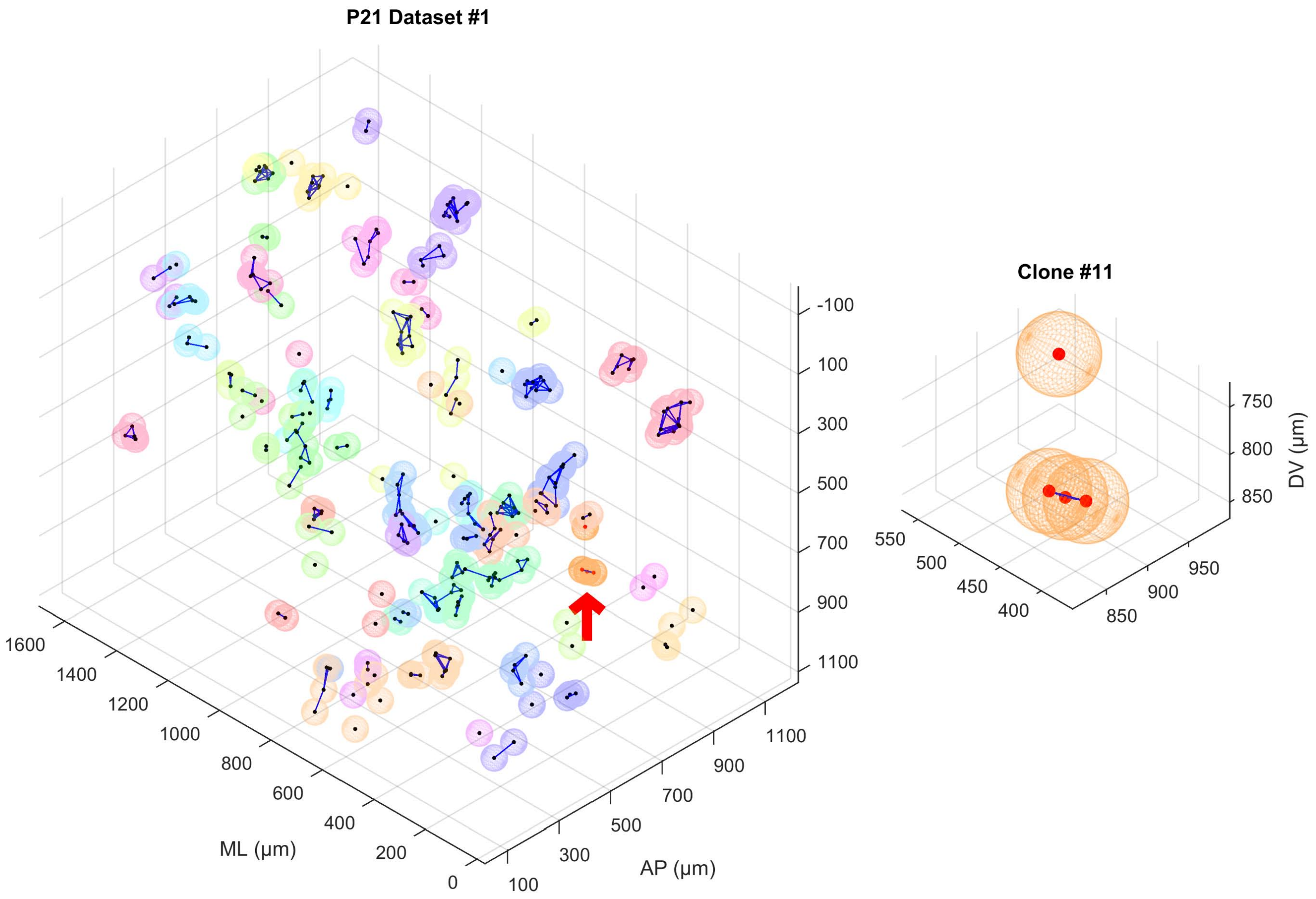

P21 Dataset #1
ML (µm)
1600
1400
1200
1000
800
600
400
200
0
AP (µm)
100
300
500
700
900
1100
-100
100
300
500
700
900
1100
Clone #11
750
800
850
DV (µm)
550
500
450
400
850
900
950

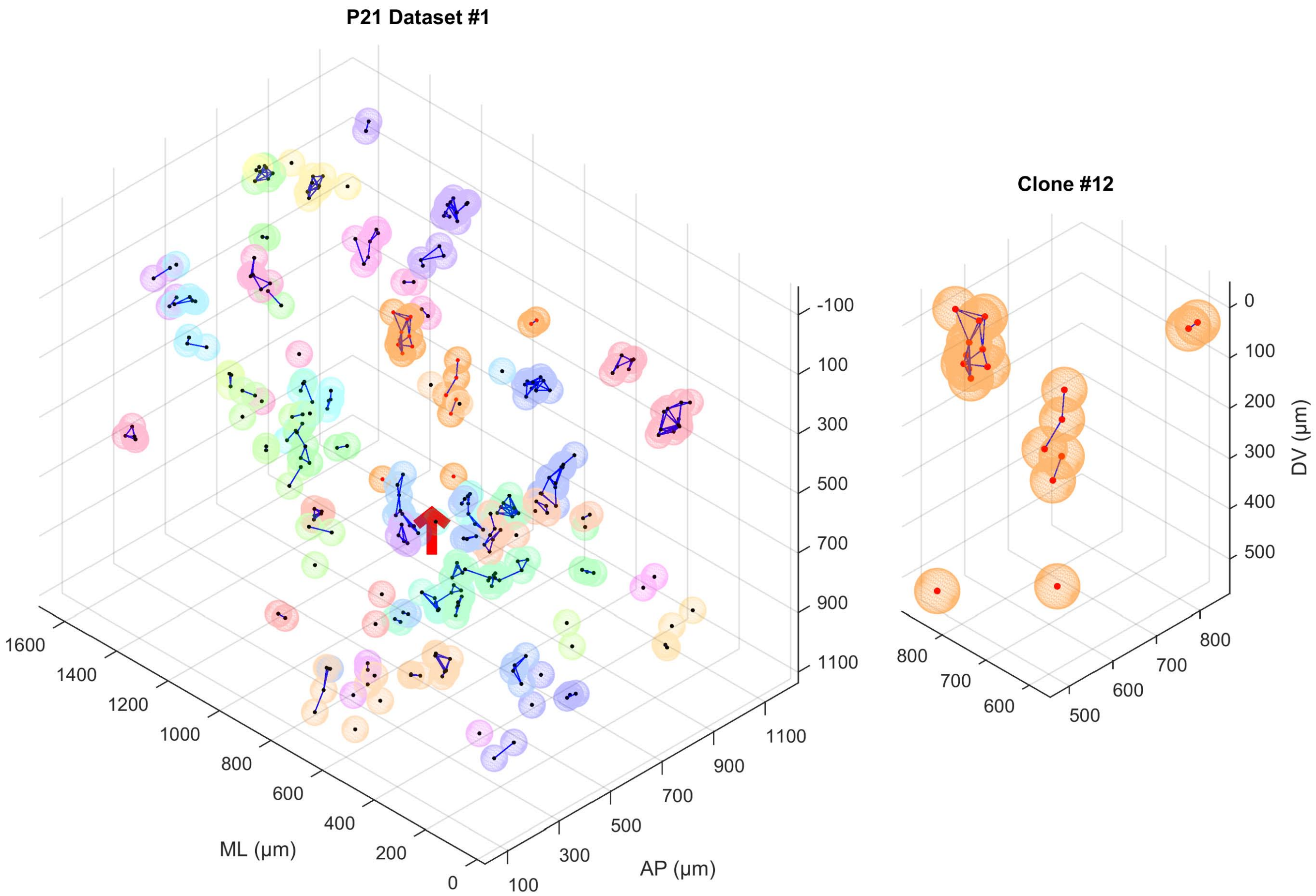

P21 Dataset #1
Clone #12
ML (μm)
AP (μm)
DV (μm)
1600
1400
1200
1000
800
600
400
200
0
100
300
500
700
900
1100
-100
100
300
500
700
900
1100
0
100
200
300
400
500
800
700
600
500
600
700
800

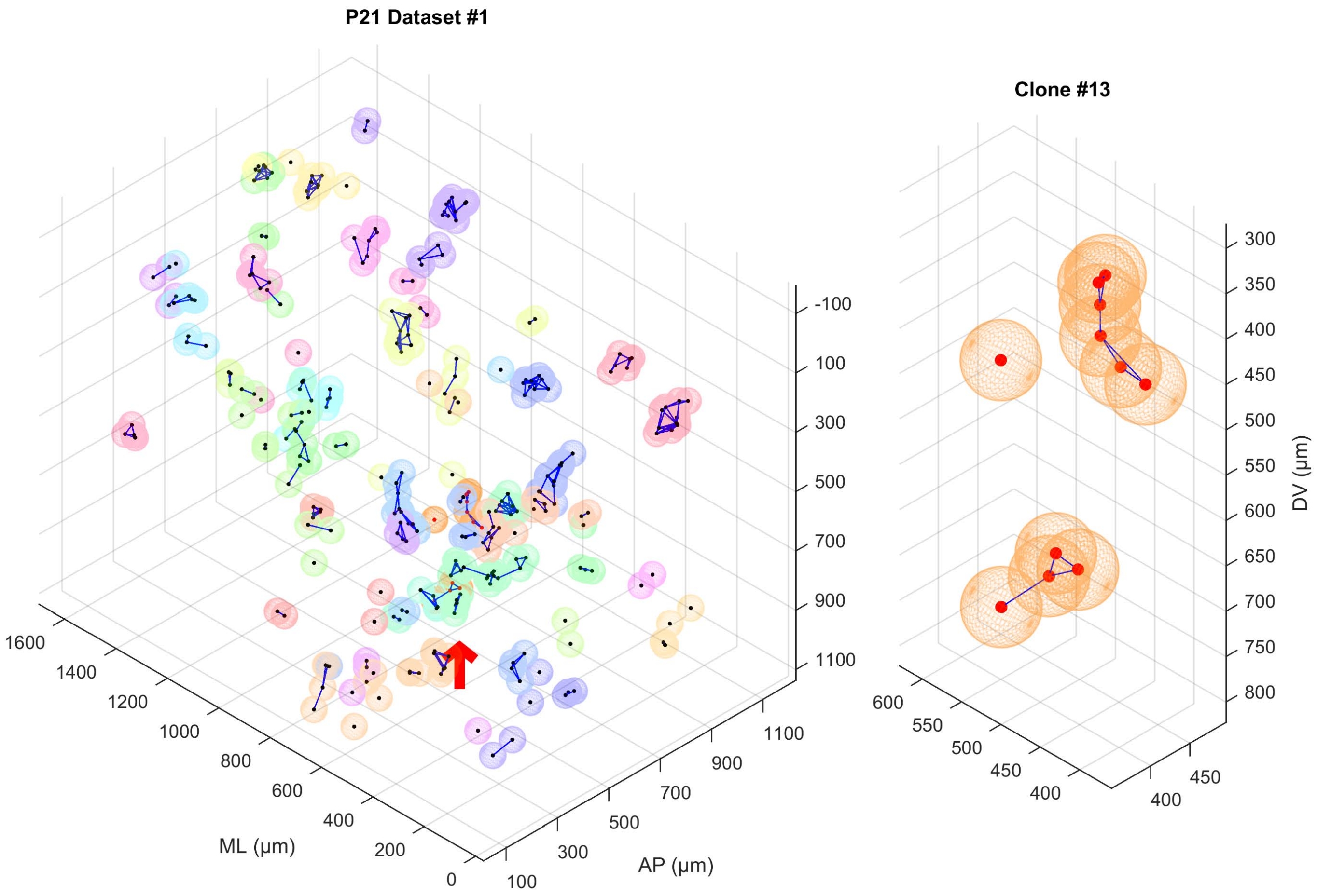

P21 Dataset #1
ML (µm)
AP (µm)
1600
1400
1200
1000
800
600
400
200
0
100
300
500
700
900
1100
-100
100
300
500
700
900
1100
Clone #13
DV (µm)
300
350
400
450
500
550
600
650
700
750
800
600
550
500
450
400
400
450

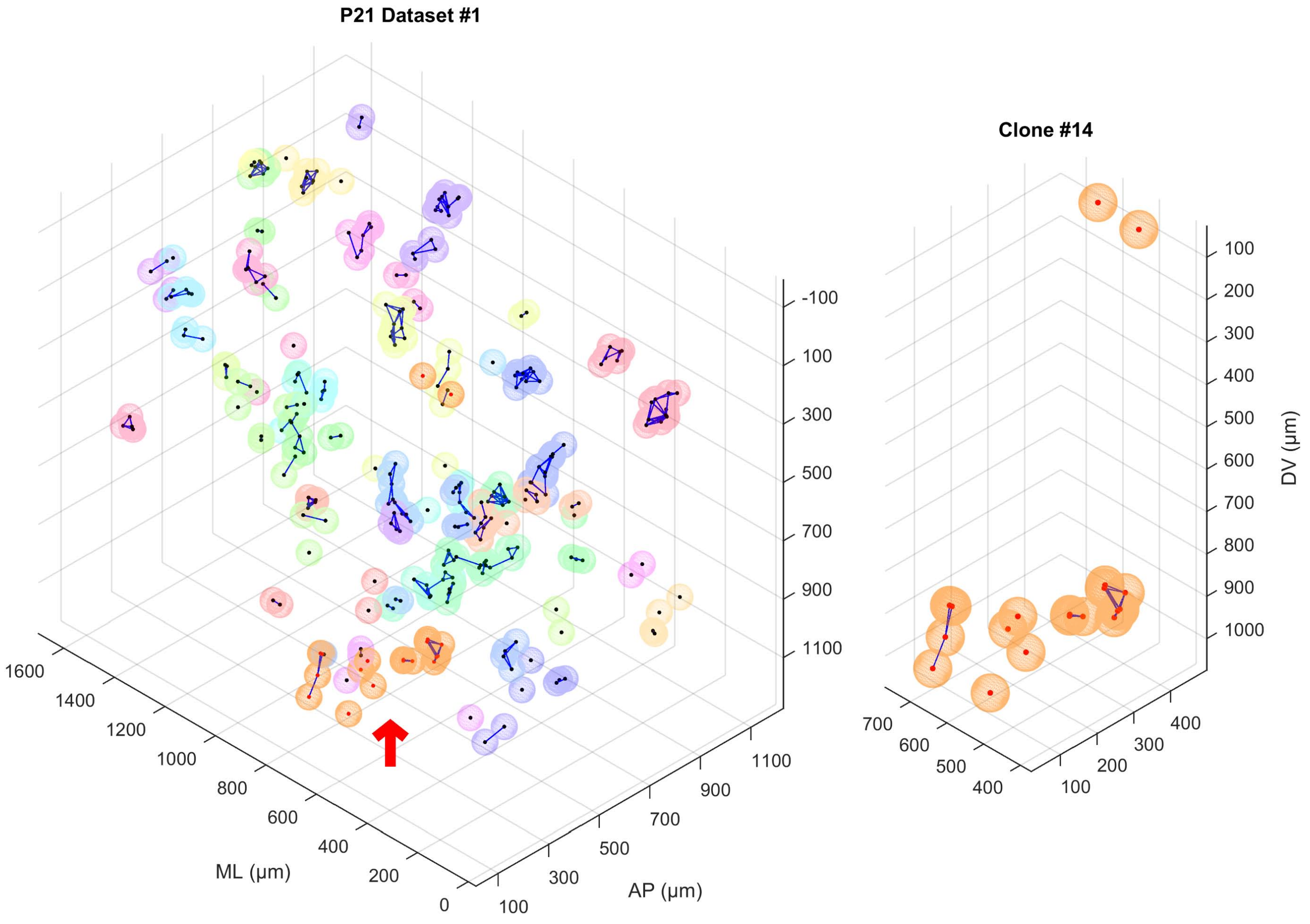

P21 Dataset #1
ML (µm)
AP (µm)
1600
1400
1200
1000
800
600
400
200
0
100
300
500
700
900
1100
-100
100
300
500
700
900
1100
Clone #14
DV (µm)
100
200
300
400
500
600
700
800
900
1000
700
600
500
400
100
200
300
400

P21 Dataset #1

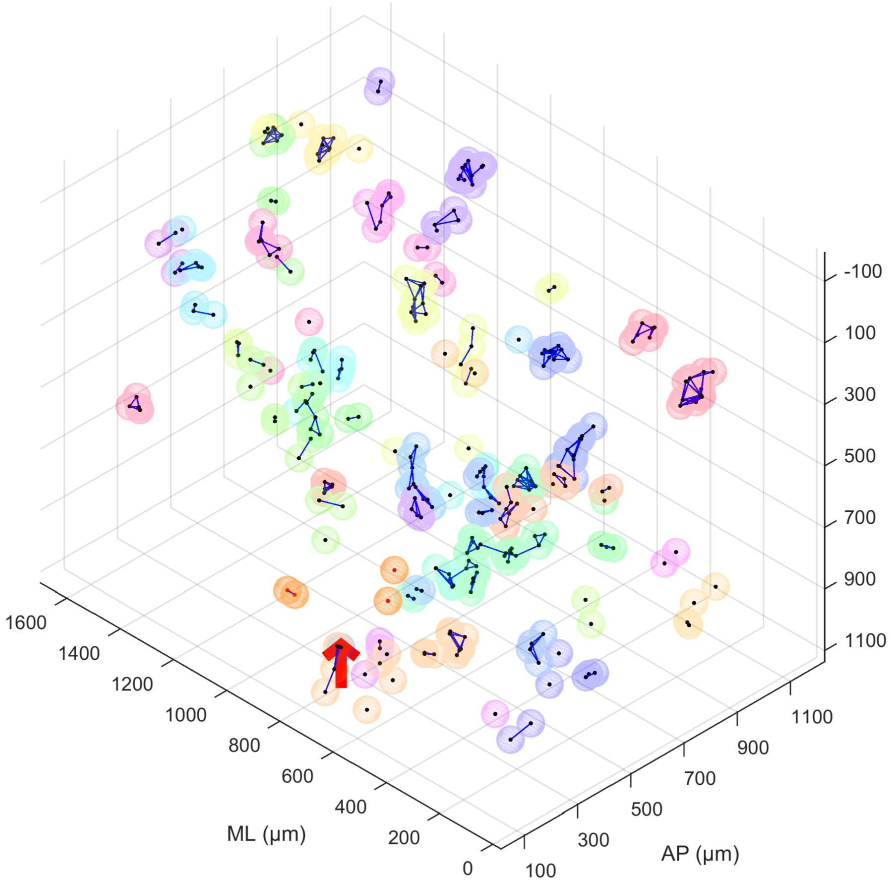

Clone #15

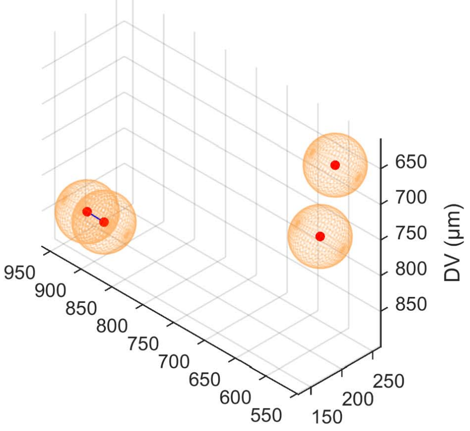

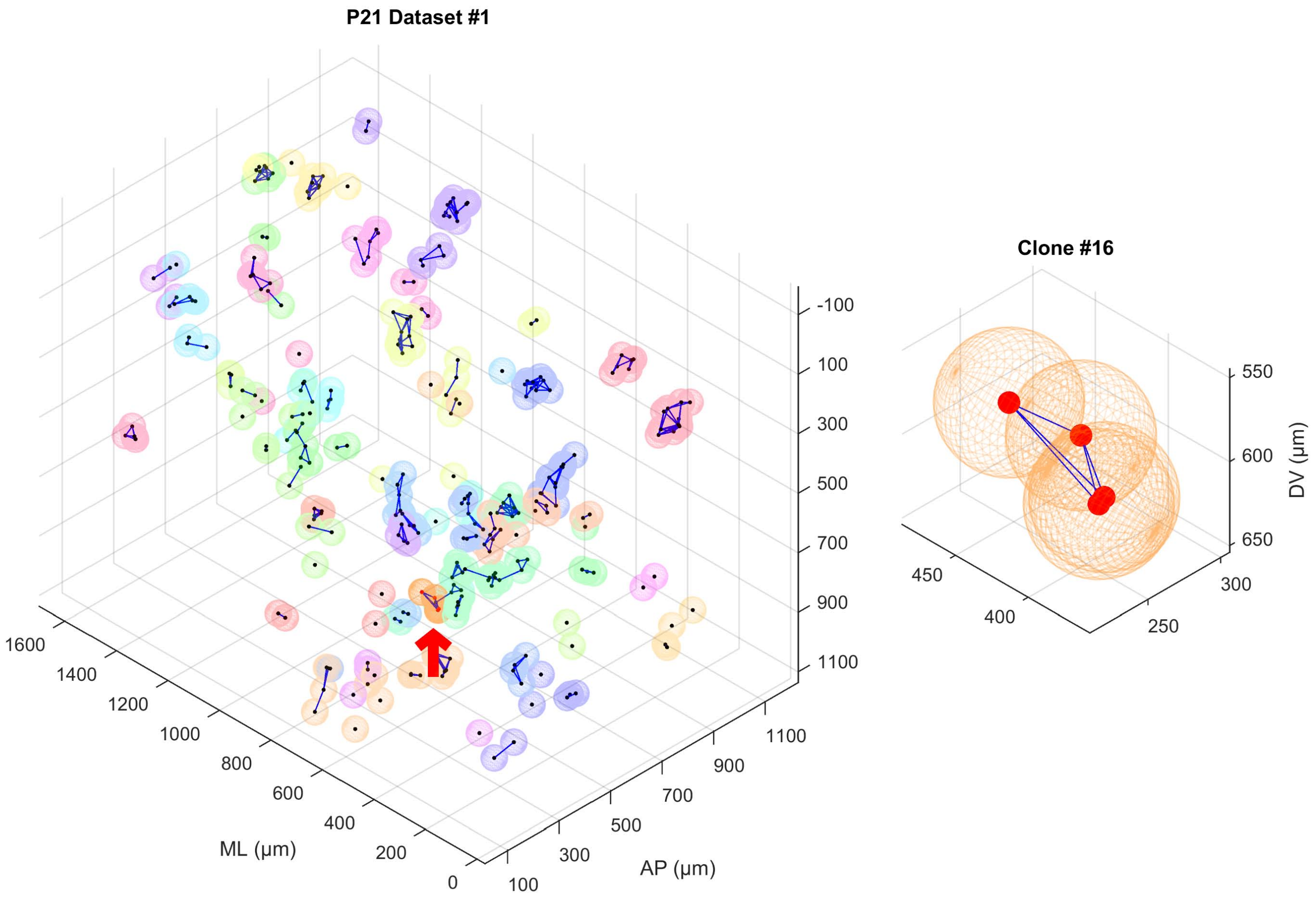

P21 Dataset #1
-100
100
300
500
700
900
1100
1600
1400
1200
1000
800
600
400
200
0
ML (µm)
100
300
500
700
900
1100
AP (µm)
Clone #16
550
600
650
DV (µm)
450
400
300
250

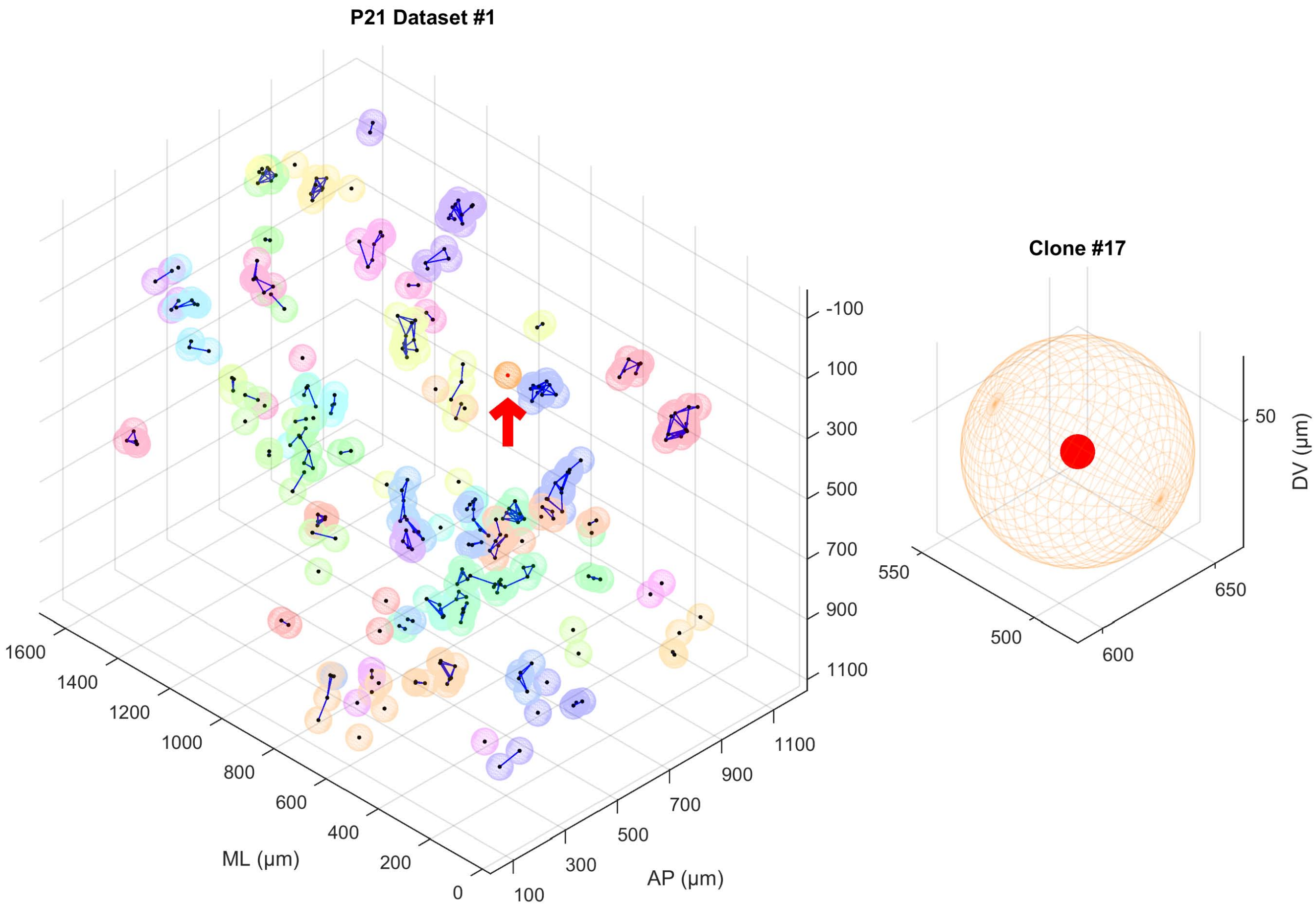
P21 Dataset #1
ML (µm)
1600
1400
1200
1000
800
600
400
200
0
AP (µm)
100
300
500
700
900
1100
-100
100
300
500
700
900
1100
Clone #17
DV (µm)
50
550
500
600
650

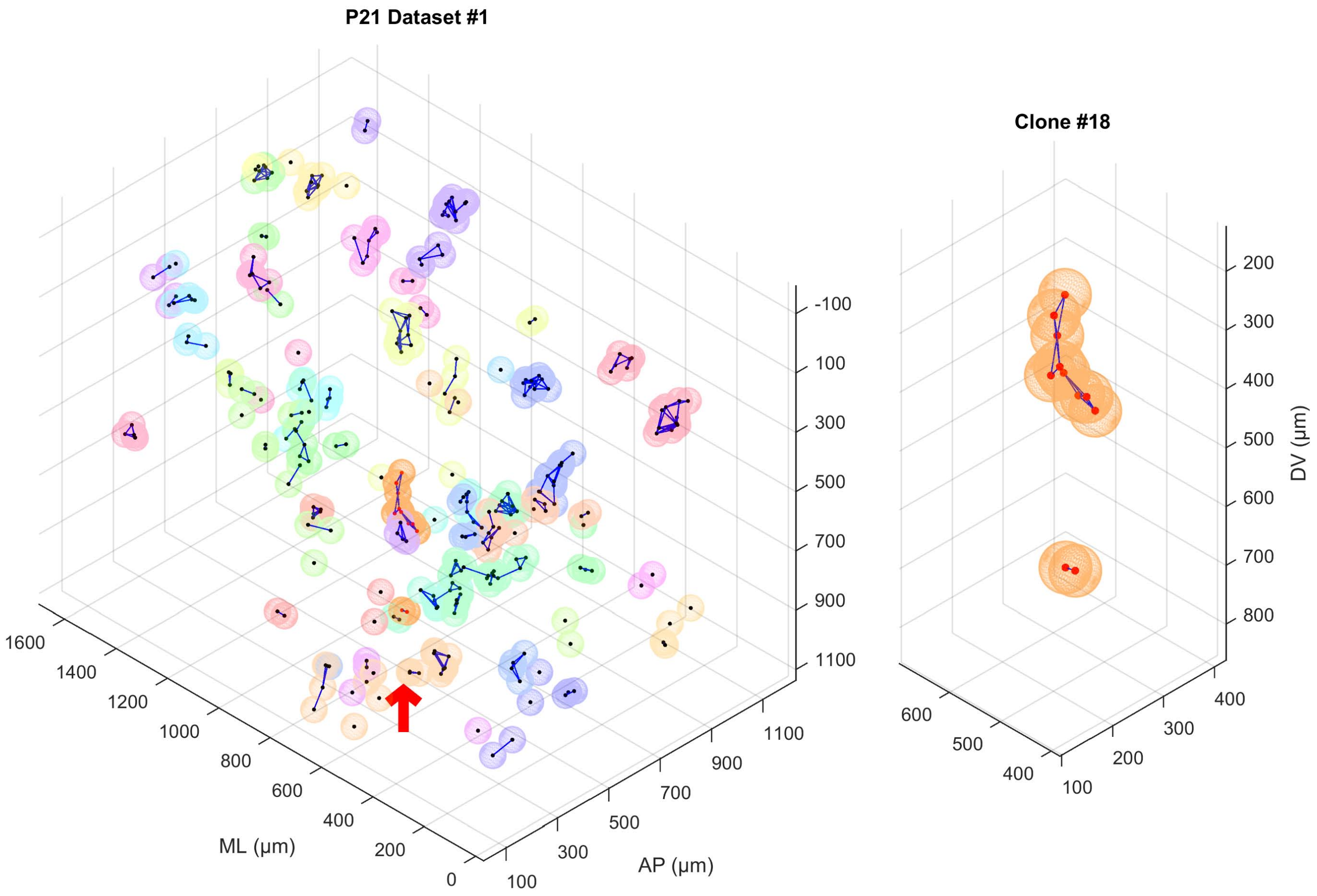

P21 Dataset #1
Clone #18
ML (µm)
AP (µm)
DV (µm)
1600
1400
1200
1000
800
600
400
200
0
100
300
500
700
900
1100
-100
100
300
500
700
900
1100
200
300
400
500
600
700
800
600
500
400
100
200
300
400

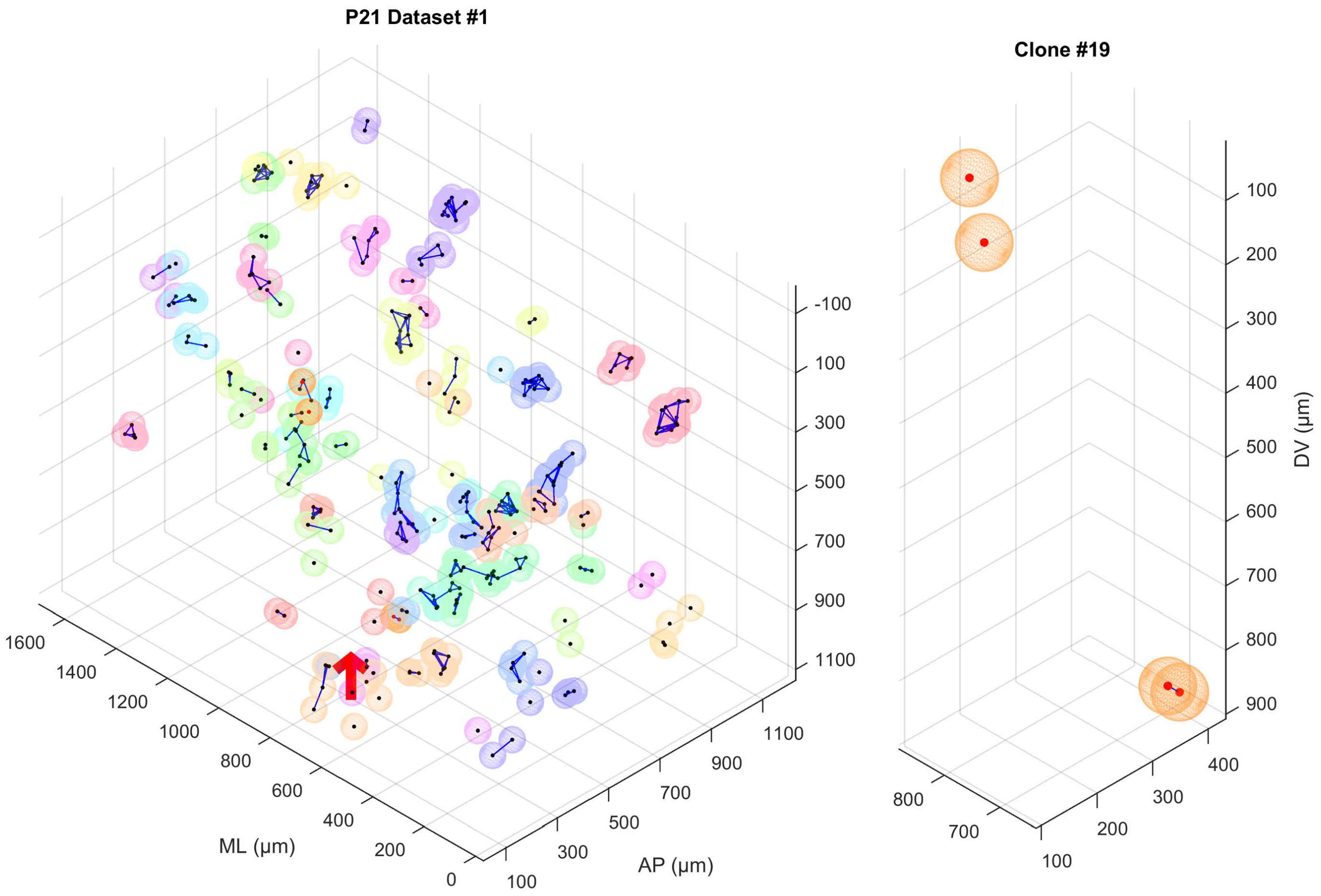

P21 Dataset #1
ML (μm)
AP (μm)
1600
1400
1200
1000
800
600
400
200
0
100
300
500
700
900
1100
-100
100
300
500
700
900
1100
Clone #19
DV (μm)
100
200
300
400
500
600
700
800
900
800
700
100
200
300
400

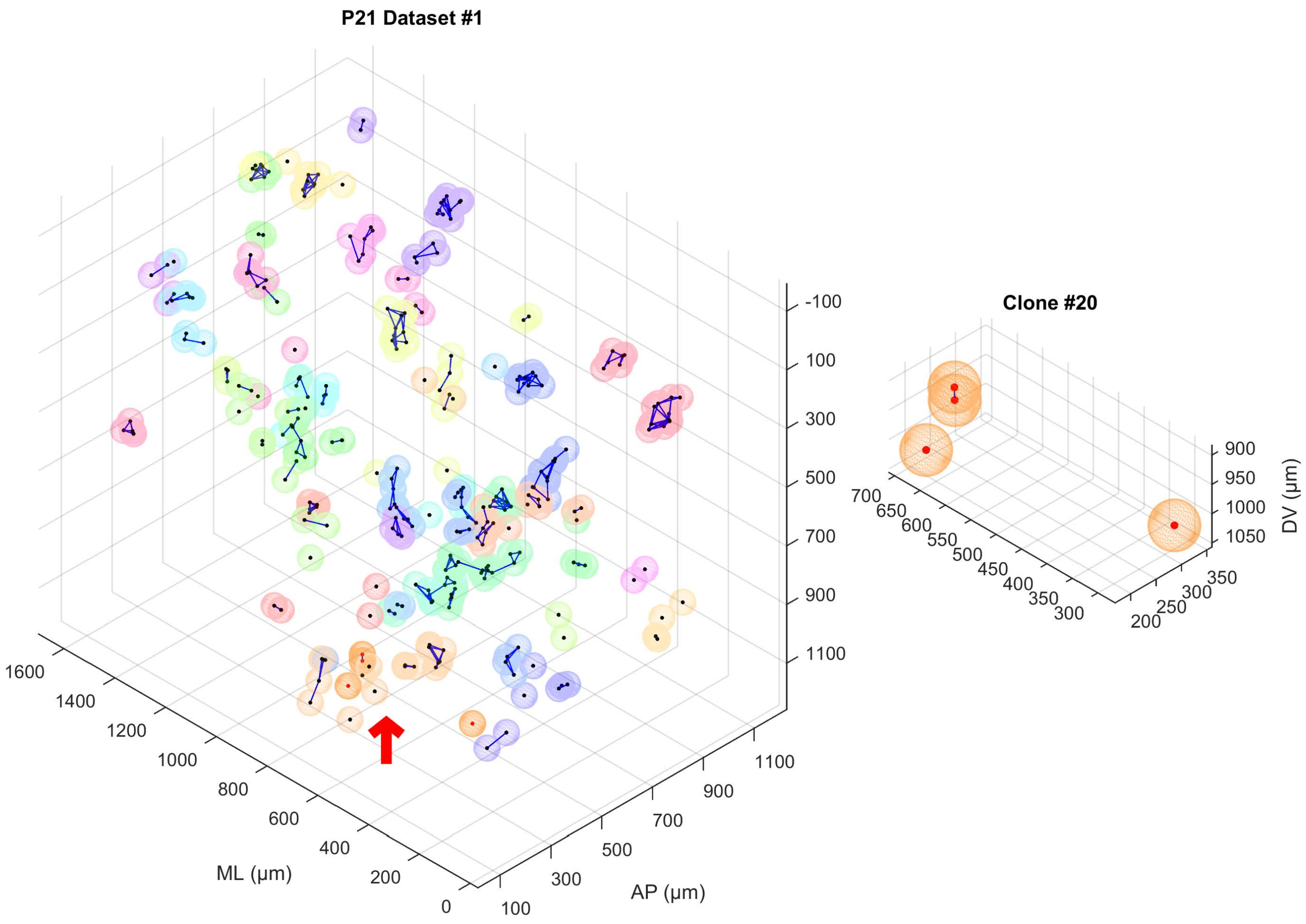
P21 Dataset #1
-100
100
300
500
700
900
1100
1600
1400
1200
1000
800
600
400
200
0
ML (µm)
100
300
500
700
900
1100
AP (µm)
Clone #20
900
950
1000
1050
DV (µm)
700
650
600
550
500
450
400
350
300
200
250
300
350

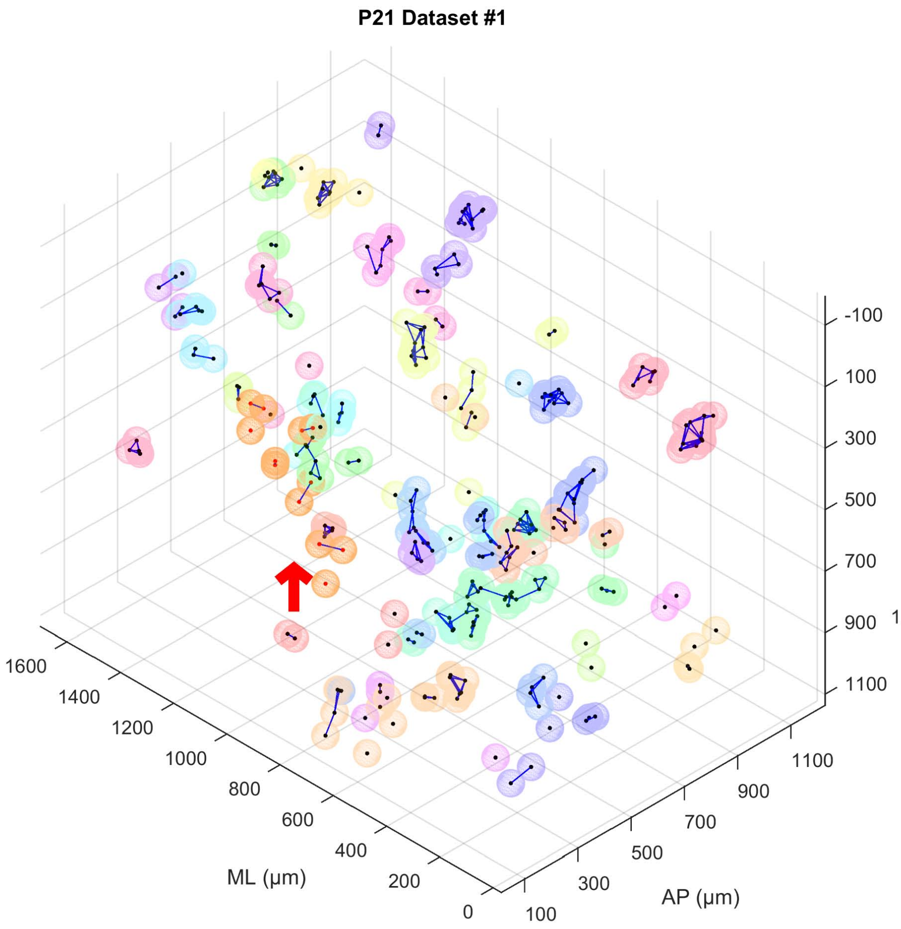
P21 Dataset #1
-100
100
300
500
700
900
1100
1600
1400
1200
1000
800
600
400
200
0
ML (µm)
100
300
500
700
900
1100
AP (µm)

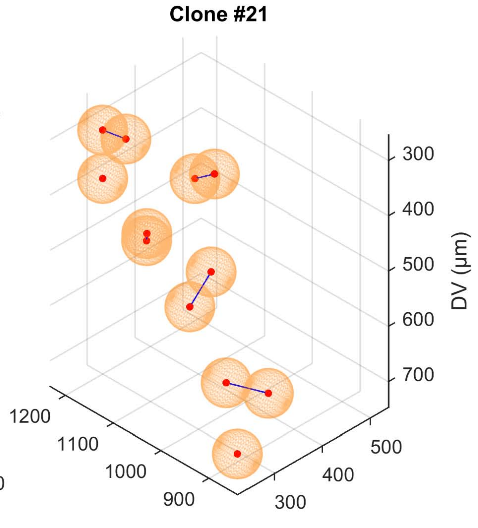
Clone #21
300
400
500
600
700
DV (µm)
1200
1100
1000
900
300
400
500

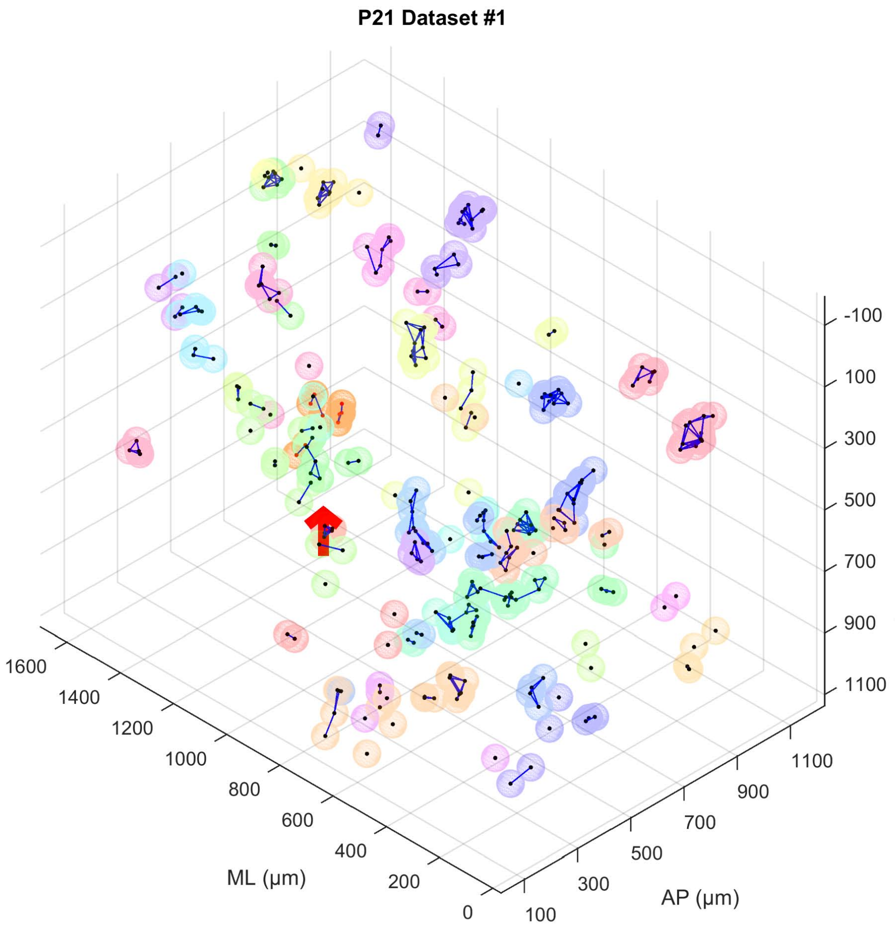
P21 Dataset #1
ML (µm)
AP (µm)
1600
1400
1200
1000
800
600
400
200
0
100
300
500
700
900
1100
-100
100
300
500
700
900
1100

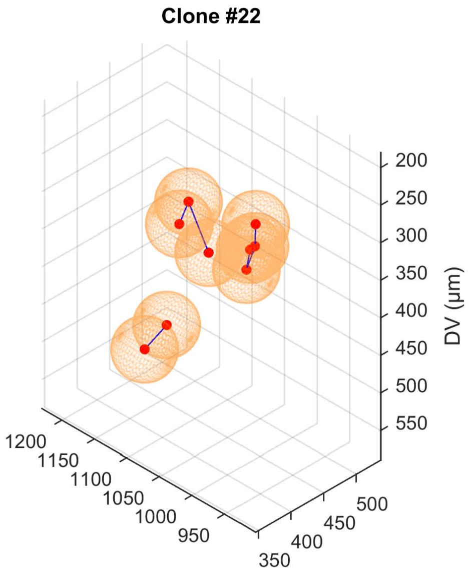
Clone #22
DV (µm)
200
250
300
350
400
450
500
550
1200
1150
1100
1050
1000
950
350
400
450
500

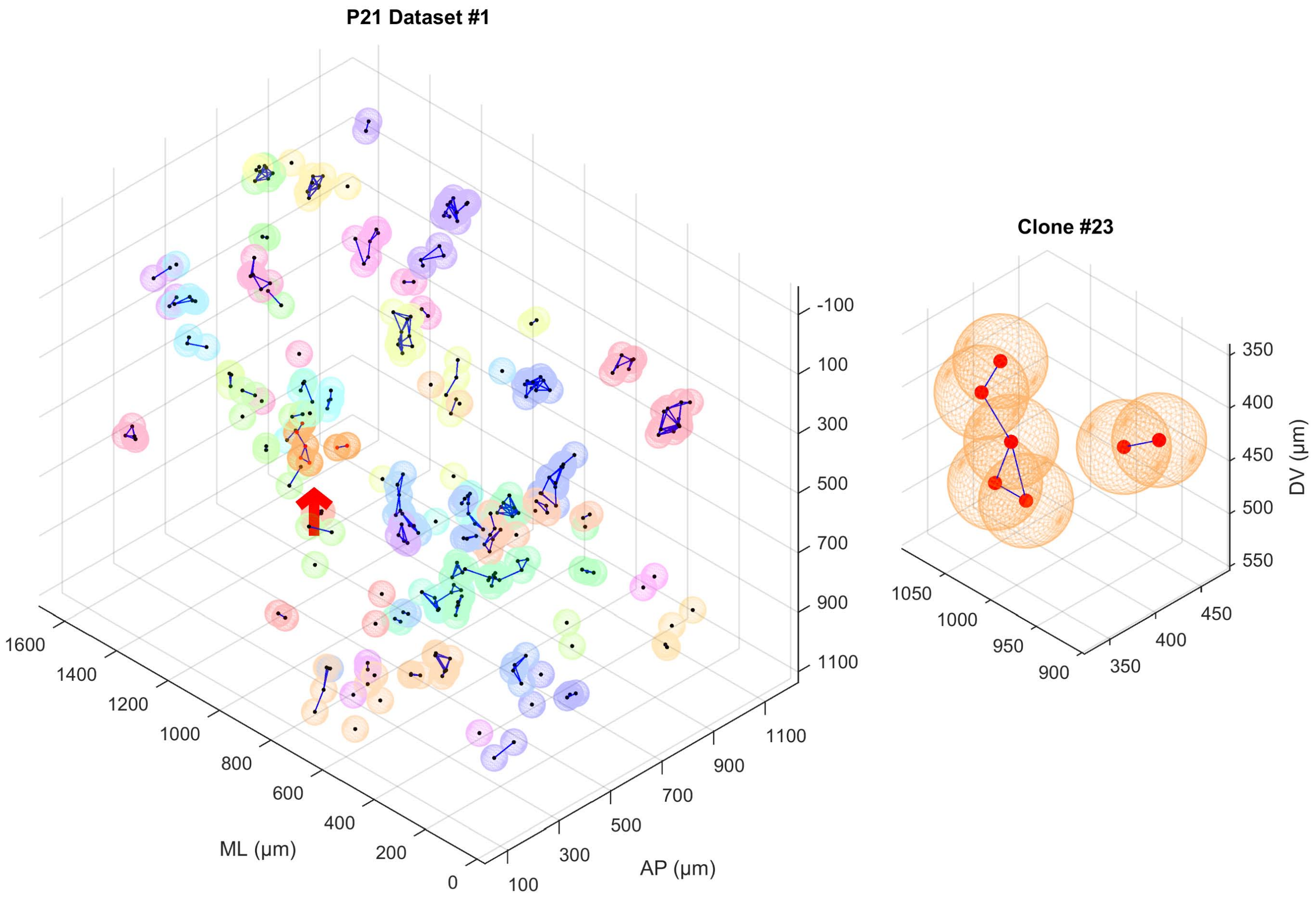

P21 Dataset #1
-100
100
300
500
700
900
1100
1600
1400
1200
1000
800
600
400
200
0
ML (μm)
100
300
500
700
900
1100
AP (μm)
Clone #23
350
400
450
500
550
DV (μm)
1050
1000
950
900
350
400
450

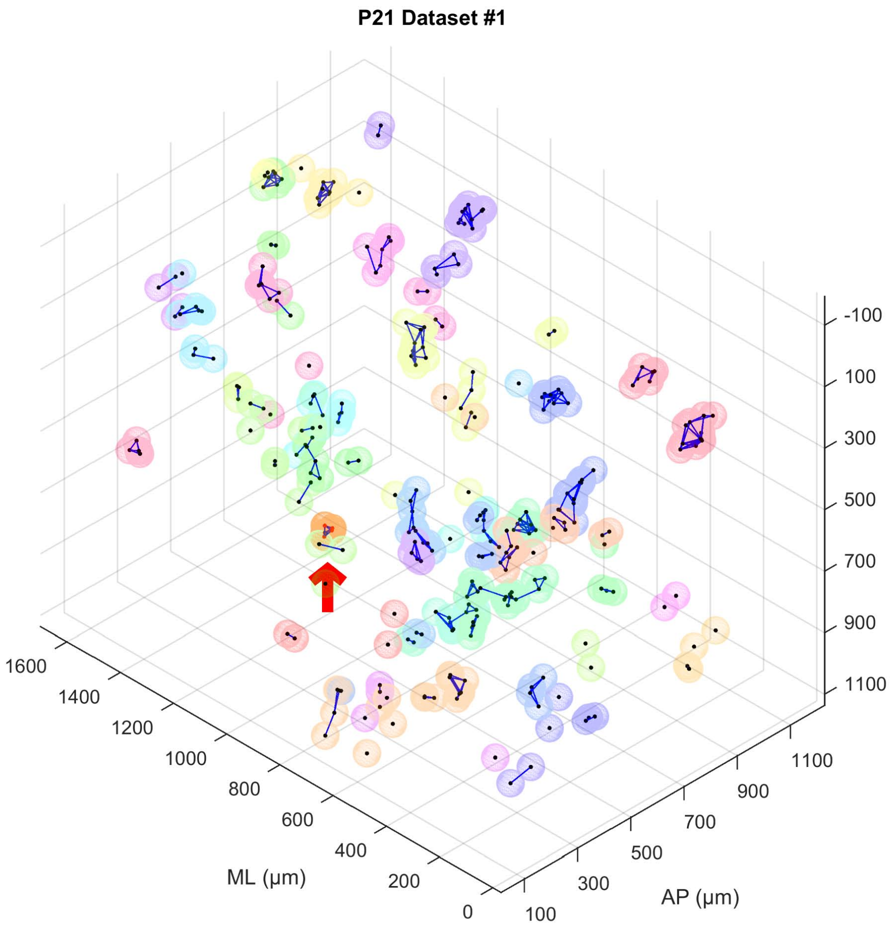
P21 Dataset #1
-100
100
300
500
700
900
1100
1600
1400
1200
1000
800
600
400
200
0
ML (μm)
100
300
500
700
900
1100
AP (μm)

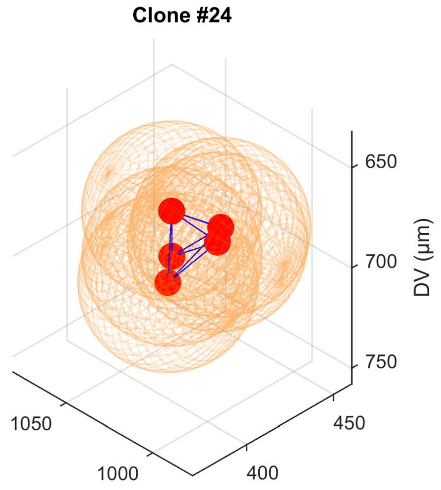
Clone #24
650
700
750
DV (μm)
1050
1000
450
400

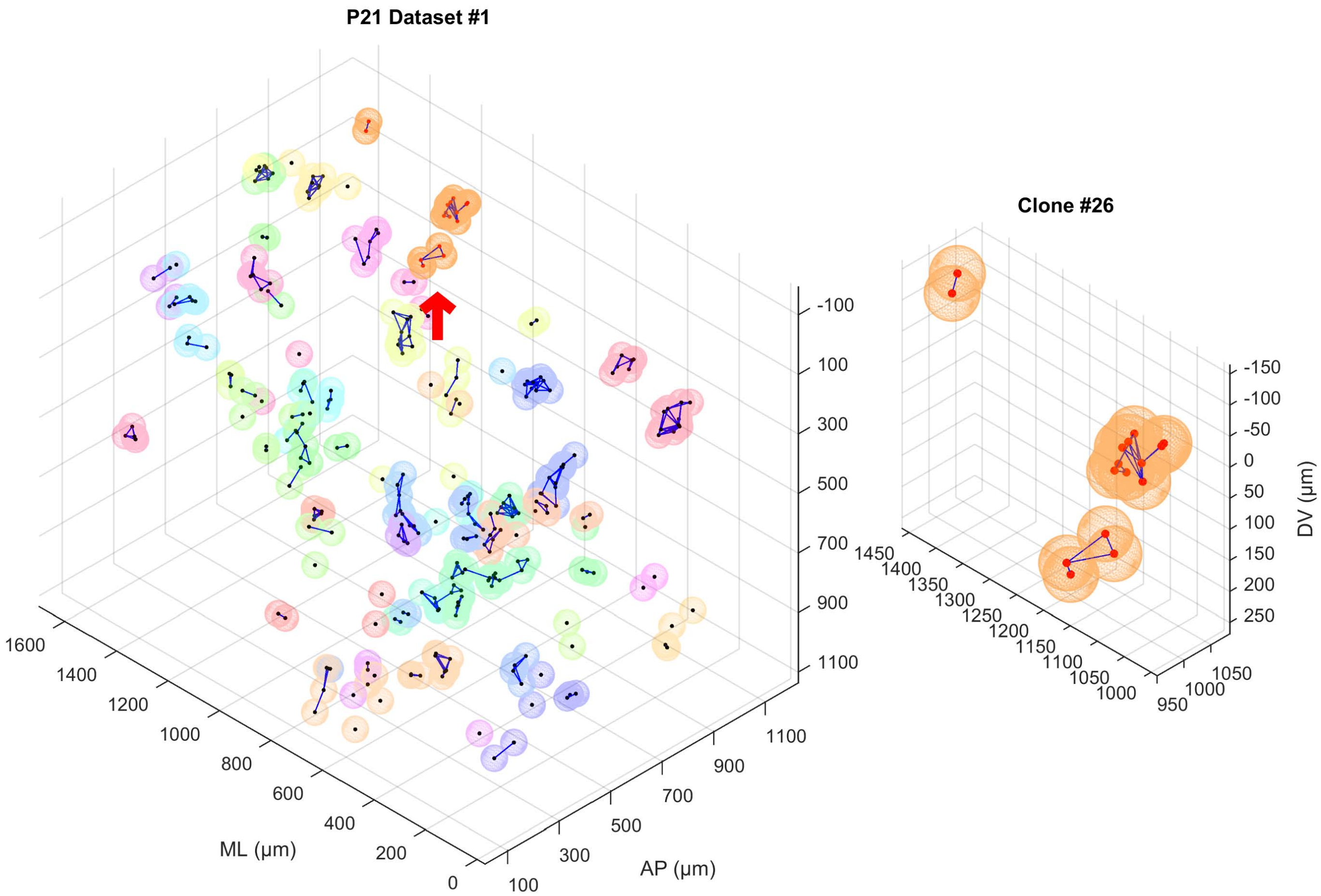
P21 Dataset #1
ML (µm)
1600
1400
1200
1000
800
600
400
200
0
AP (µm)
100
300
500
700
900
1100
-100
100
300
500
700
900
1100
Clone #26
1450
1400
1350
1300
1250
1200
1150
1100
1050
1000
950
1000
1050
-150
-100
-50
0
50
100
150
200
250
DV (µm)

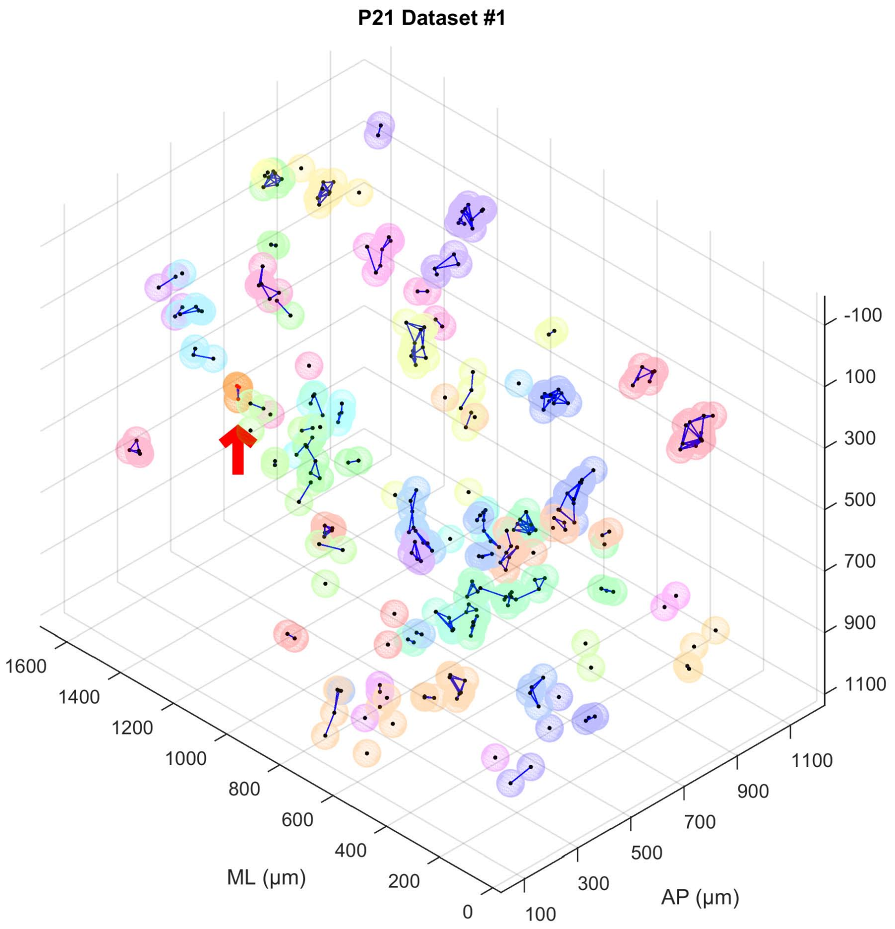
P21 Dataset #1
-100
100
300
500
700
900
1100
1600
1400
1200
1000
800
600
400
200
0
ML (μm)
100
300
500
700
900
1100
AP (μm)
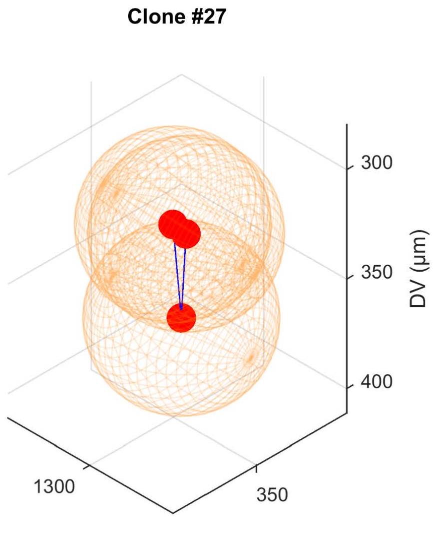
Clone #27
300
350
400
DV (μm)
1300
350

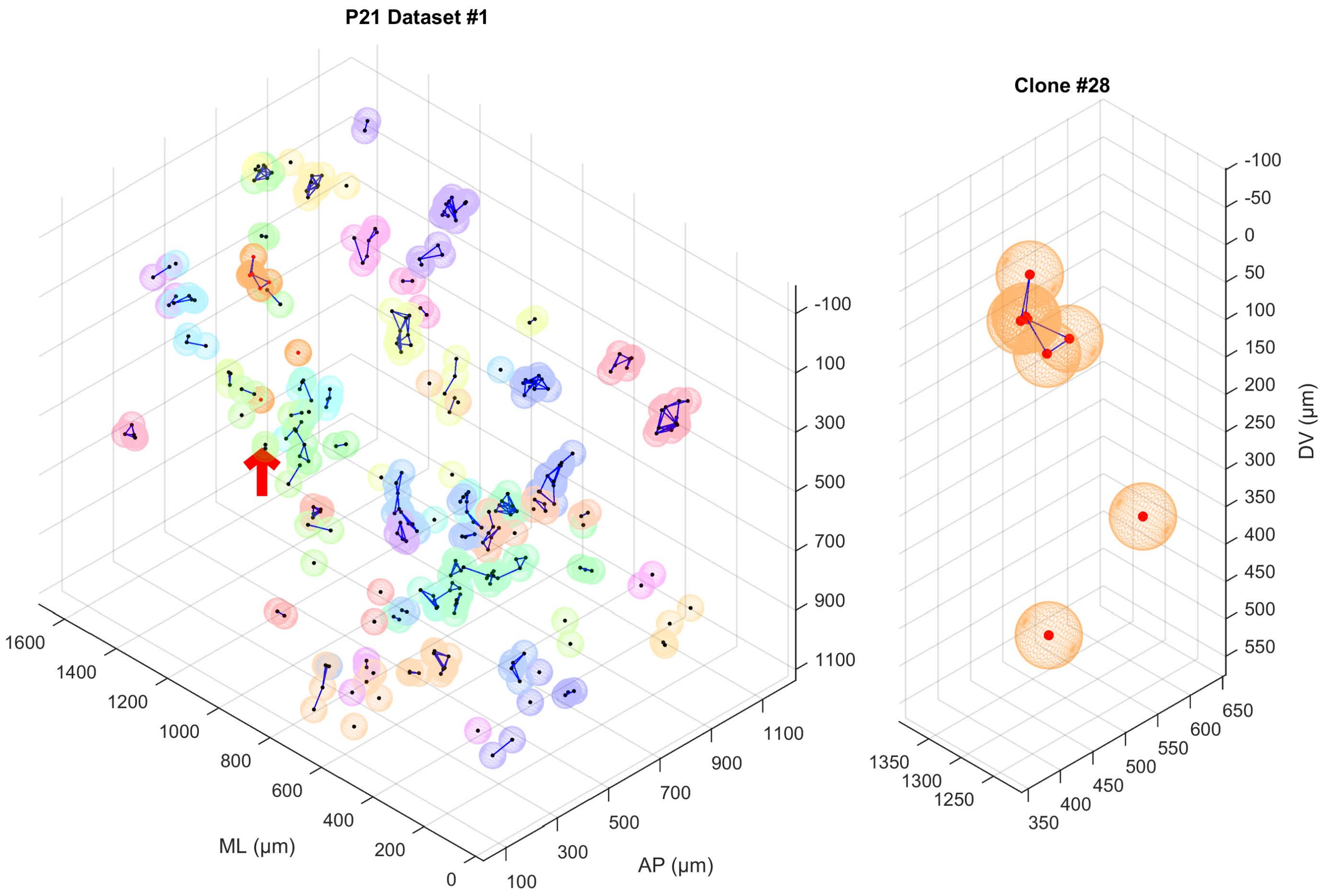

P21 Dataset #1
Clone #28
ML (µm)
AP (µm)
DV (µm)
1600
1400
1200
1000
800
600
400
200
0
100
300
500
700
900
1100
-100
100
300
500
700
900
1100
-100
-50
0
50
100
150
200
250
300
350
400
450
500
550
1350
1300
1250
350
400
450
500
550
600
650

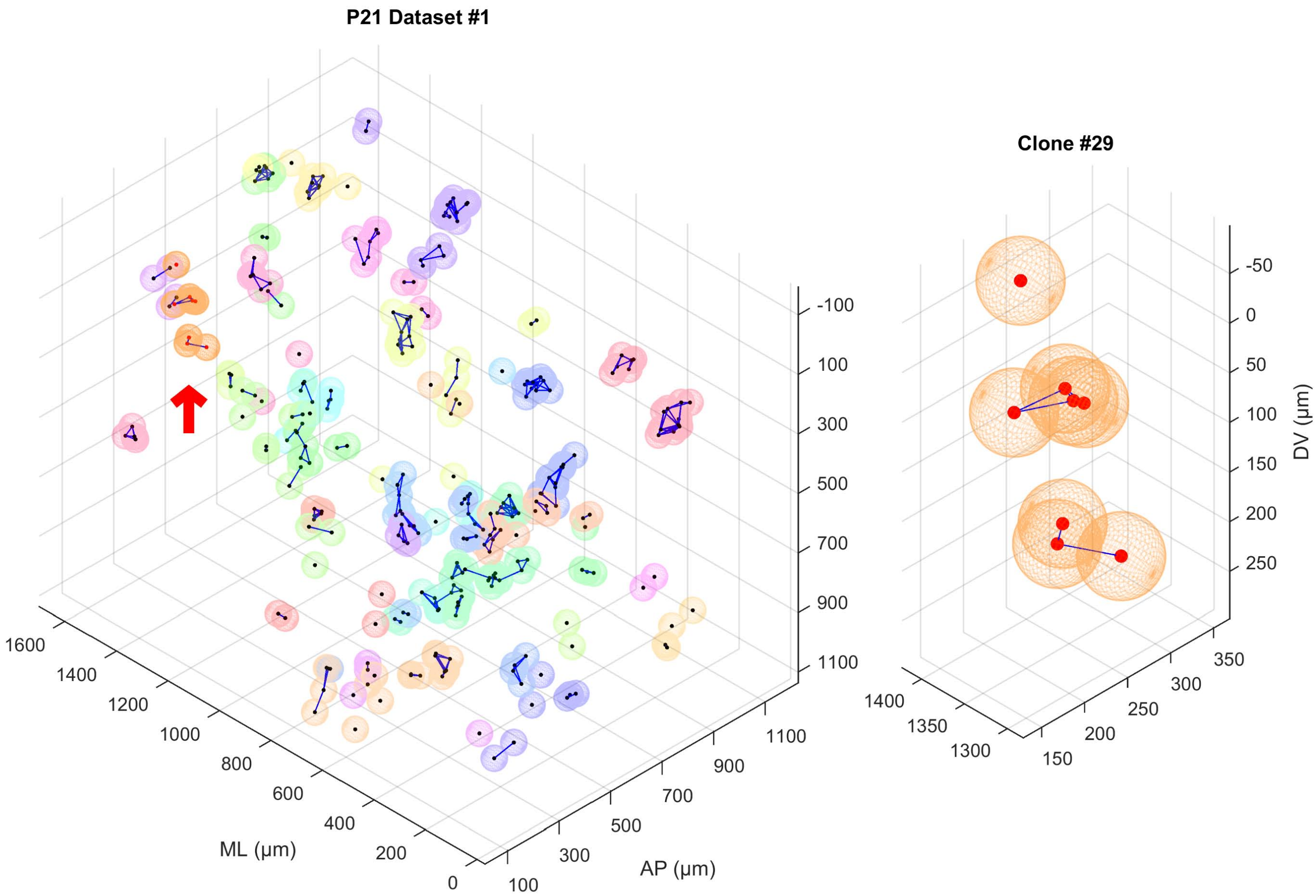
P21 Dataset #1
ML (µm)
AP (µm)
1600
1400
1200
1000
800
600
400
200
0
100
300
500
700
900
1100
-100
100
300
500
700
900
1100
Clone #29
DV (µm)
-50
0
50
100
150
200
250
1400
1350
1300
150
200
250
300
350

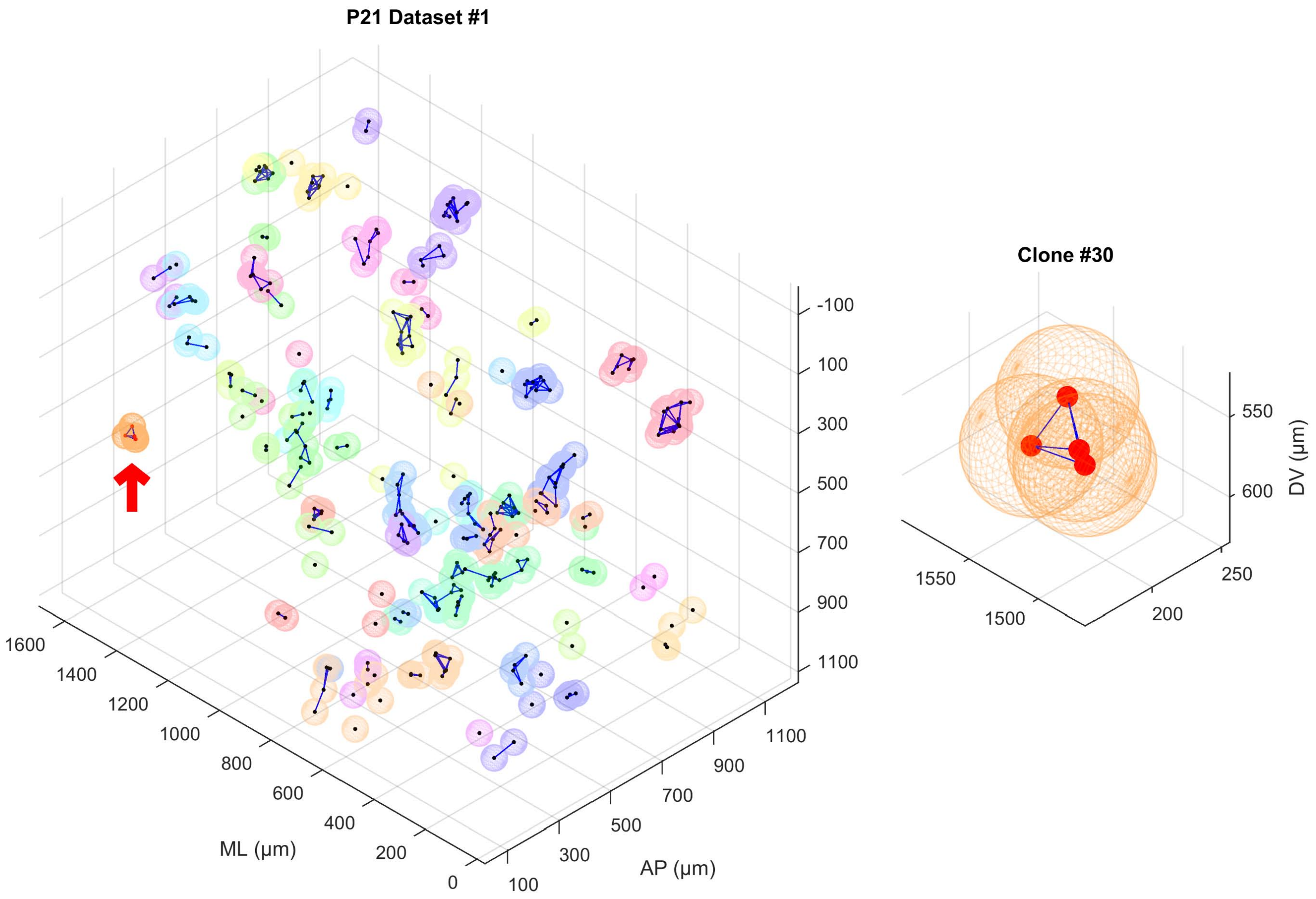
P21 Dataset #1
ML (µm)
AP (µm)
1600
1400
1200
1000
800
600
400
200
0
100
300
500
700
900
1100
-100
100
300
500
700
900
1100
Clone #30
DV (µm)
550
600
1550
1500
200
250

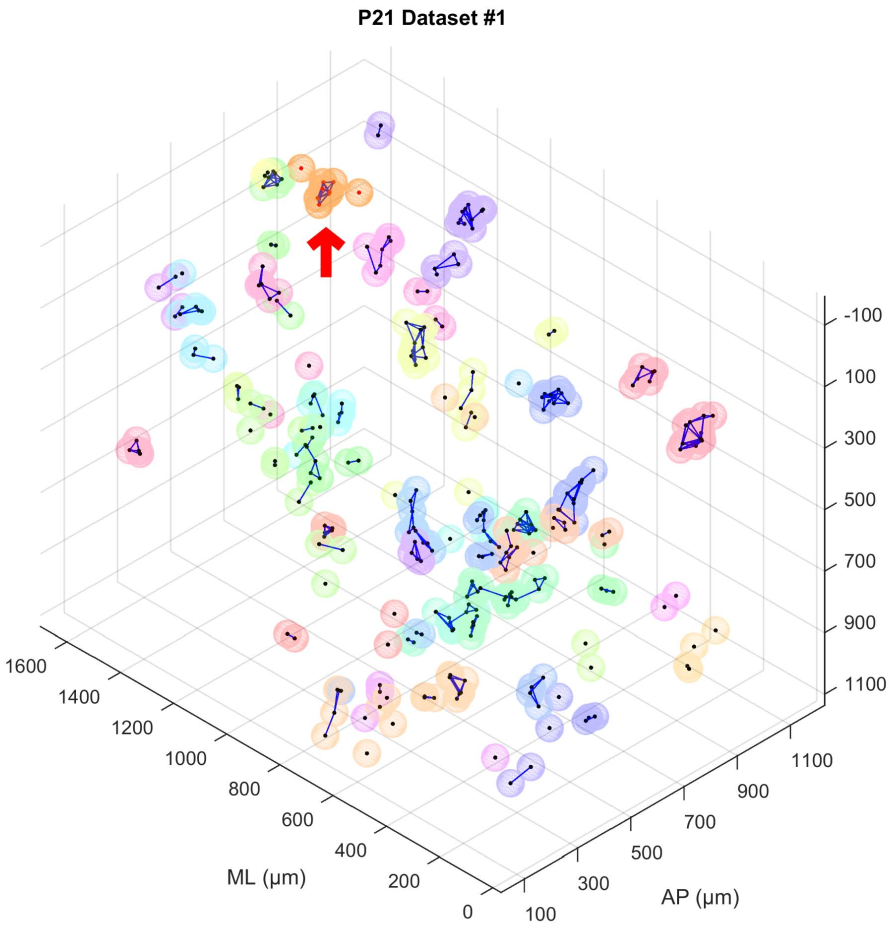

P21 Dataset #1
-100
100
300
500
700
900
1100
1600
1400
1200
1000
800
600
400
200
0
ML (µm)
100
300
500
700
900
1100
AP (µm)

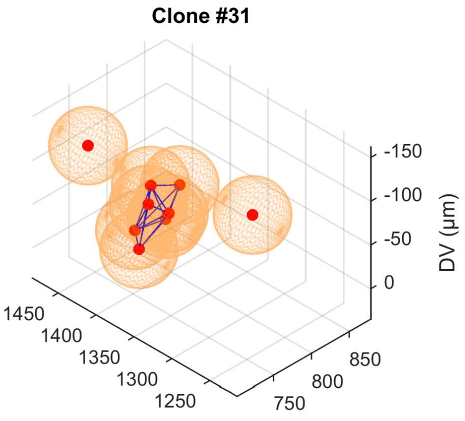

Clone #31
-150
-100
-50
0
DV (µm)
1450
1400
1350
1300
1250
750
800
850

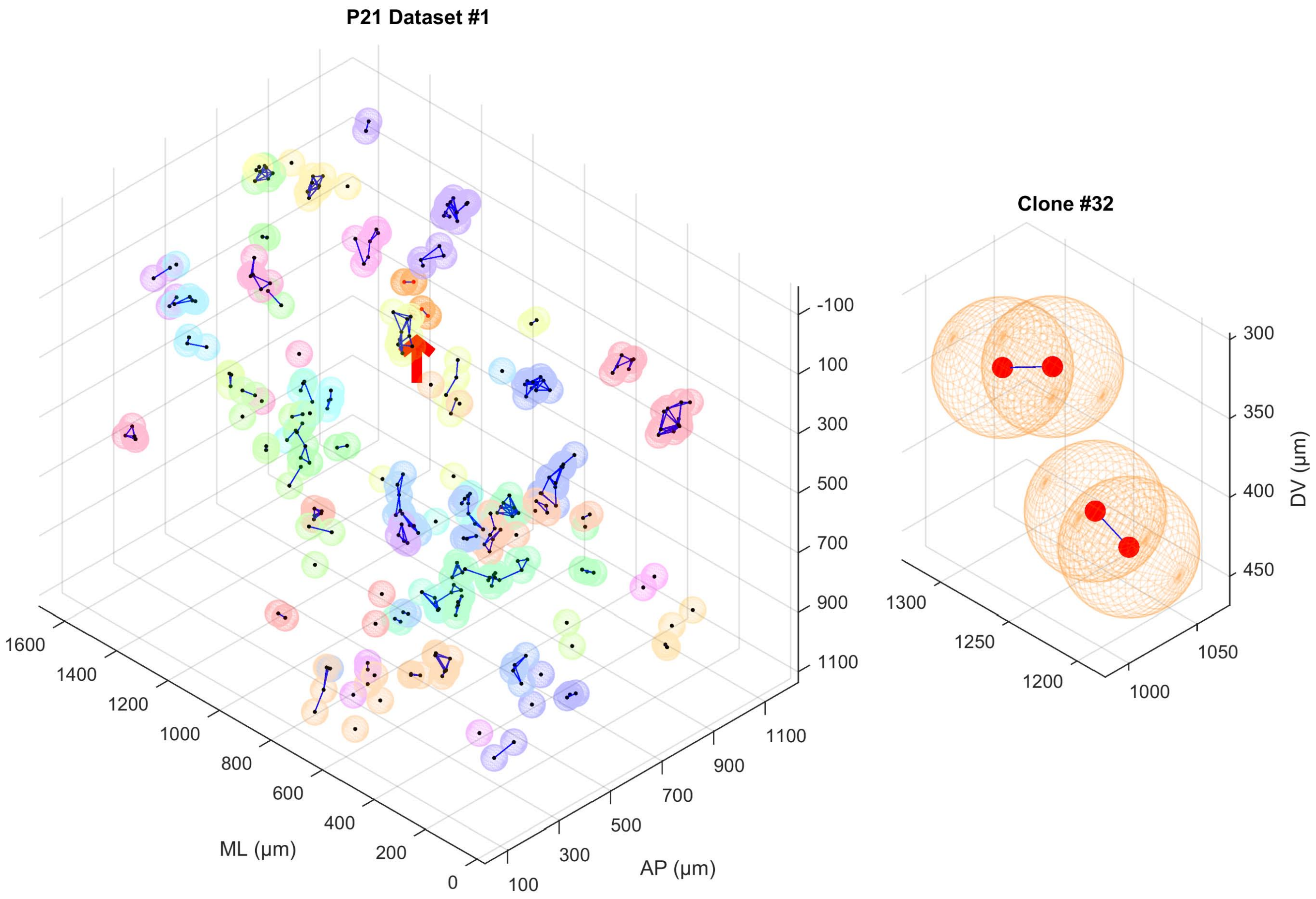

P21 Dataset #1
-100
100
300
500
700
900
1100
1600
1400
1200
1000
800
600
400
200
0
ML (µm)
100
300
500
700
900
1100
AP (µm)
Clone #32
300
350
400
450
DV (µm)
1300
1250
1200
1000
1050

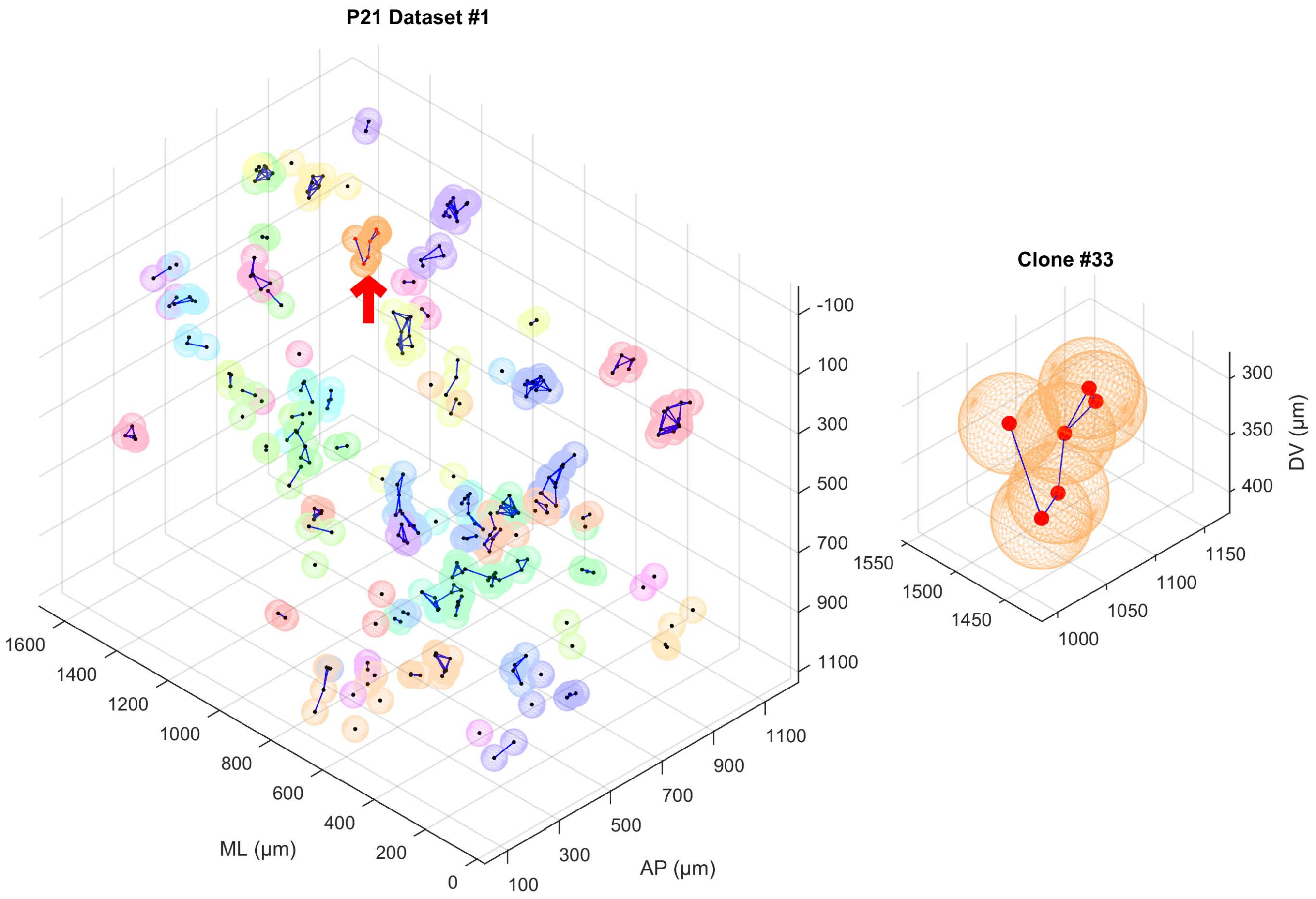
P21 Dataset #1
-100
100
300
500
700
900
1100
1600
1400
1200
1000
800
600
400
200
0
ML (μm)
100
300
500
700
900
1100
AP (μm)
Clone #33
300
350
400
DV (μm)
1550
1500
1450
1000
1050
1100
1150

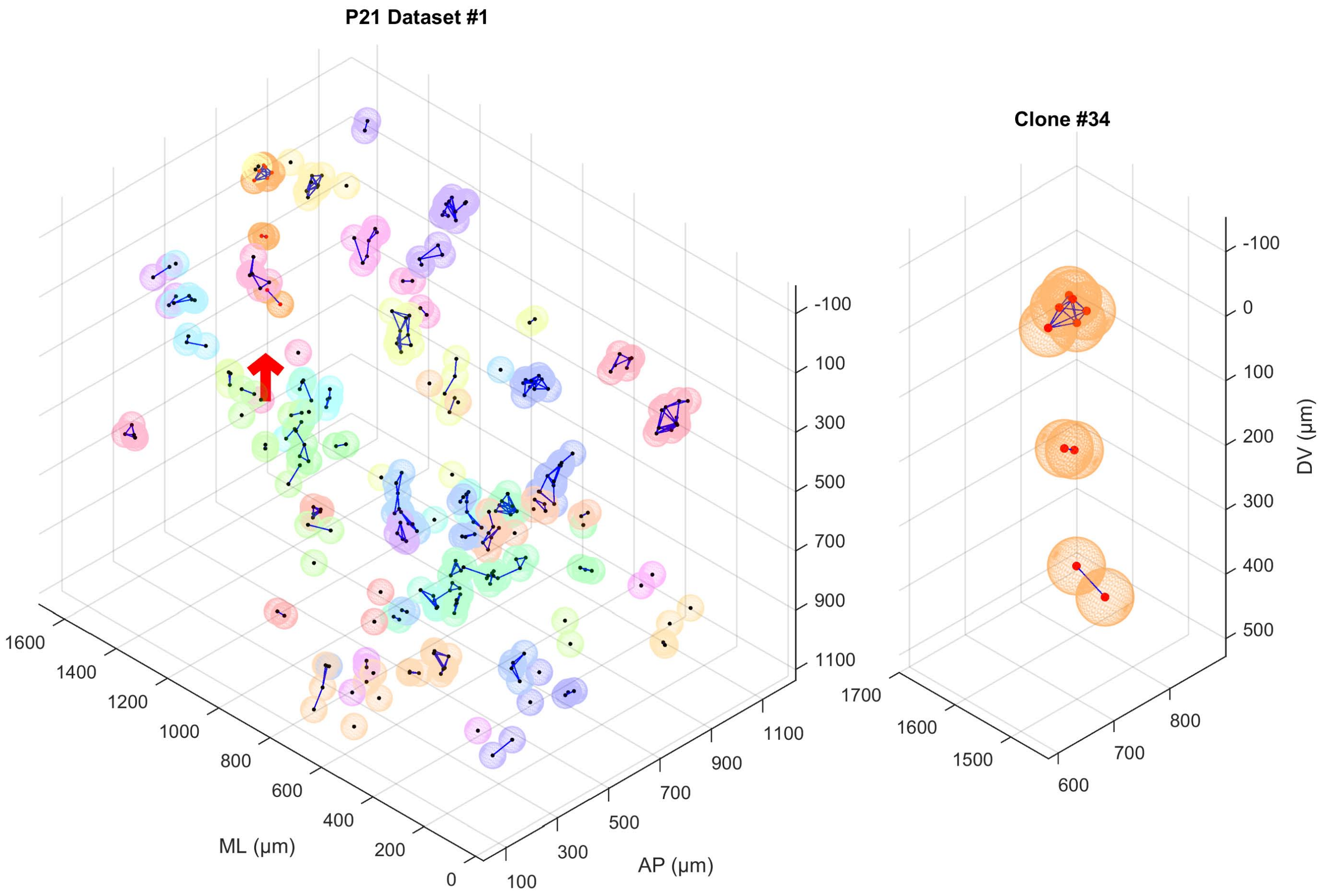

P21 Dataset #1
-100
100
300
500
700
900
1100
1600
1400
1200
1000
800
600
400
200
0
ML (µm)
100
300
500
700
900
1100
AP (µm)
Clone #34
-100
0
100
200
300
400
500
DV (µm)
1700
1600
1500
600
700
800

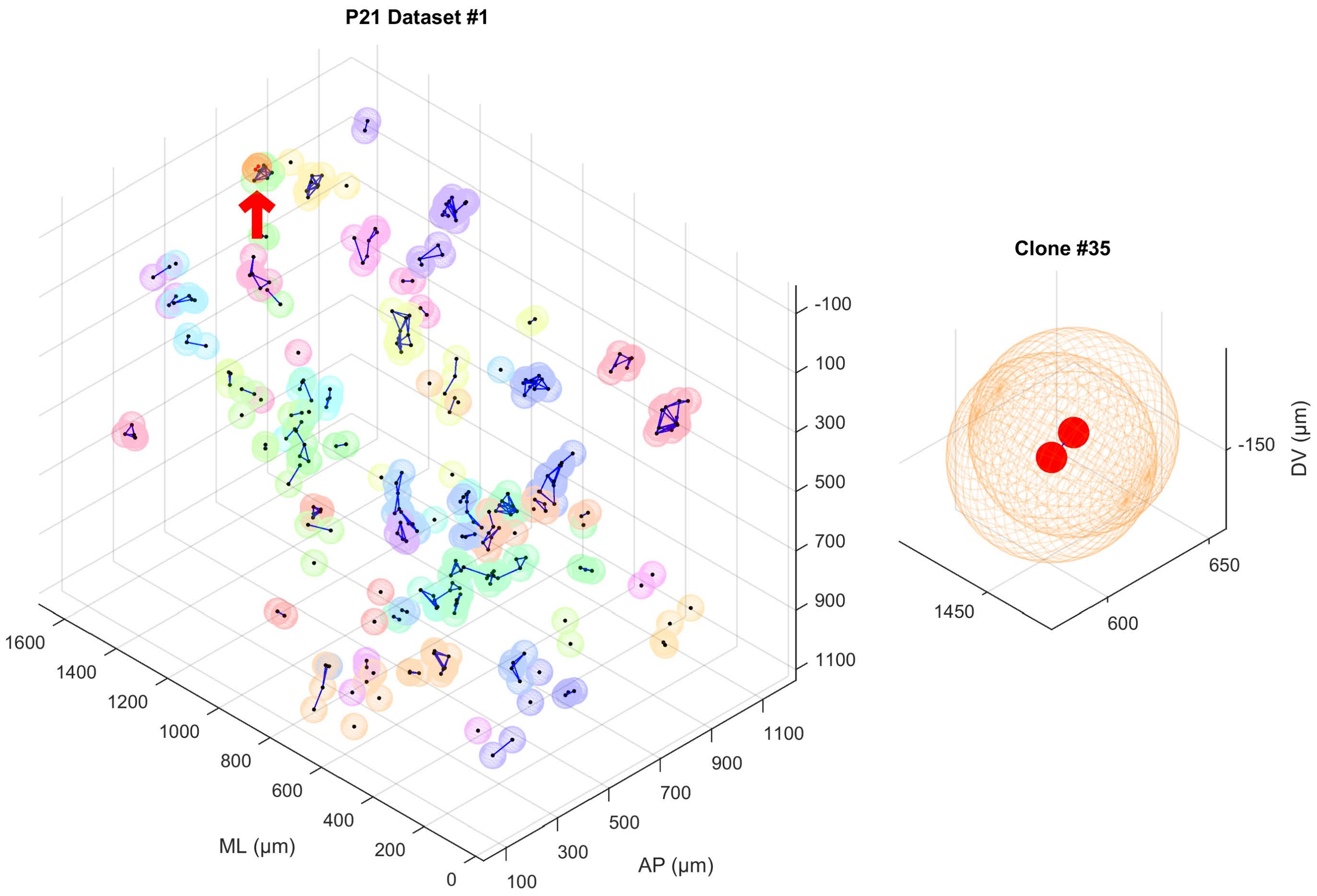

P21 Dataset #1
ML (µm)
AP (µm)
1600
1400
1200
1000
800
600
400
200
0
100
300
500
700
900
1100
-100
100
300
500
700
900
1100
Clone #35
DV (µm)
-150
1450
600
650

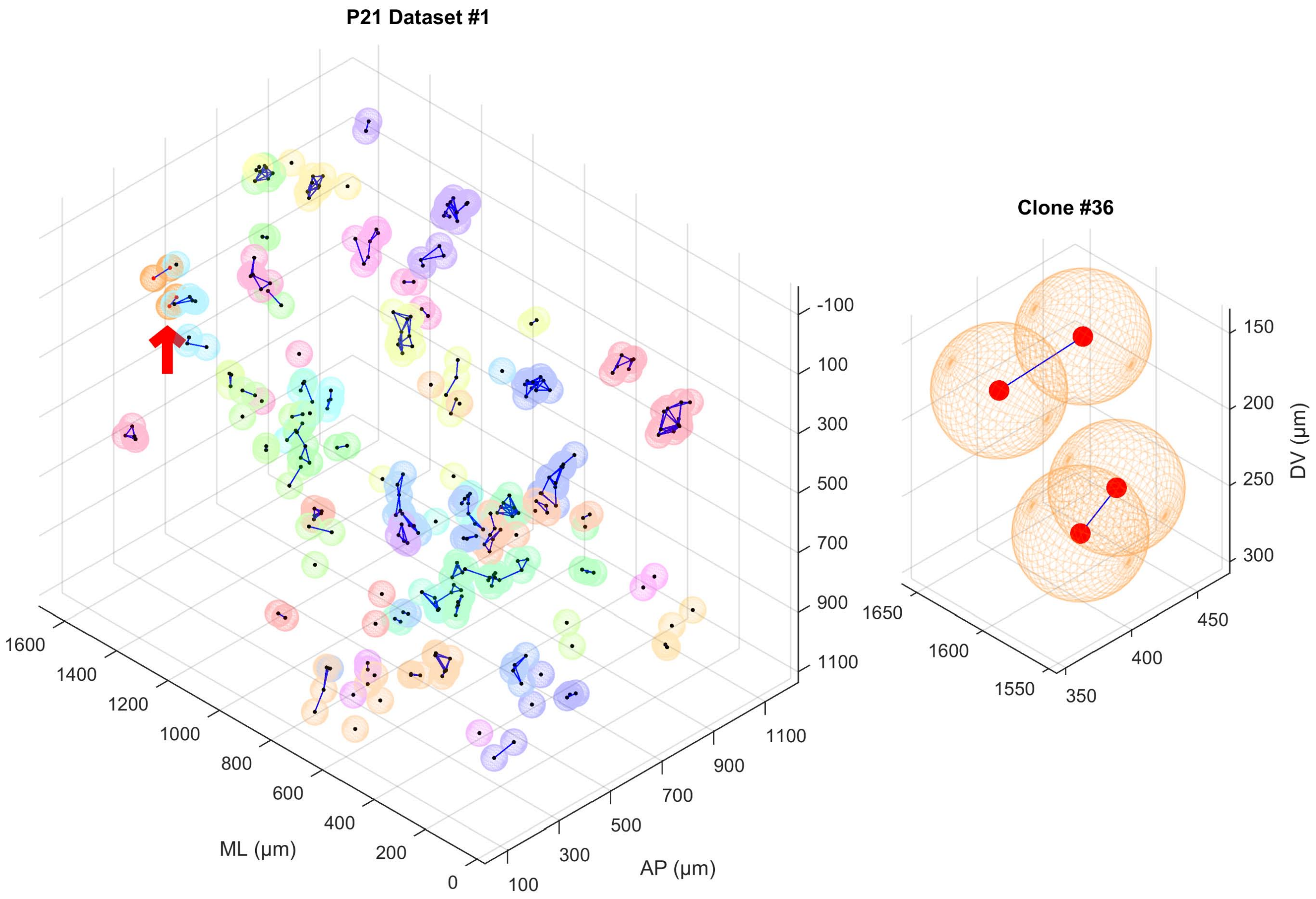

P21 Dataset #1
ML (µm)
AP (µm)
1600
1400
1200
1000
800
600
400
200
0
100
300
500
700
900
1100
-100
100
300
500
700
900
1100
Clone #36
DV (µm)
150
200
250
300
1650
1600
1550
350
400
450

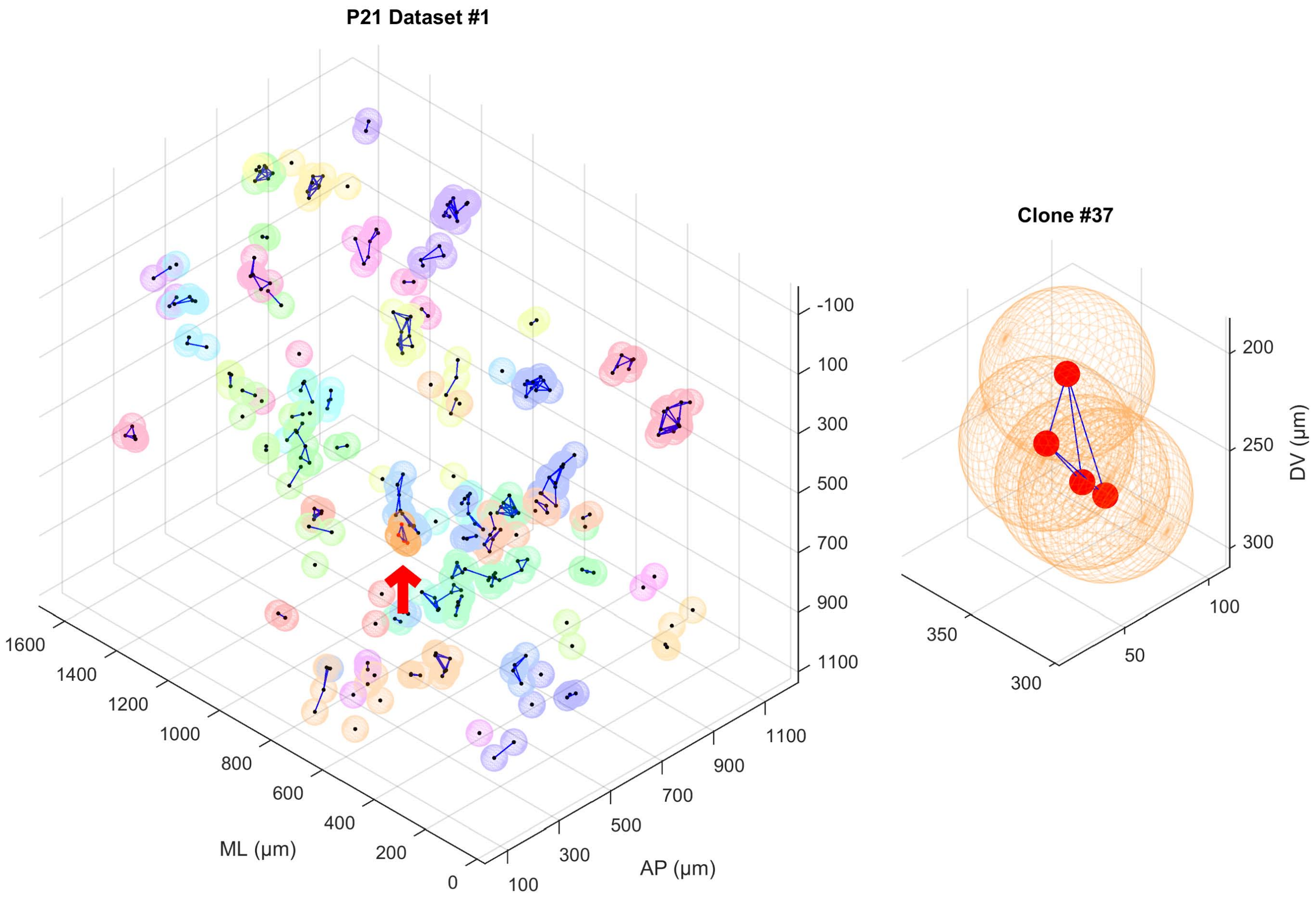

P21 Dataset #1
-100
100
300
500
700
900
1100
1600
1400
1200
1000
800
600
400
200
0
ML (µm)
100
300
500
700
900
1100
AP (µm)
Clone #37
200
250
300
DV (µm)
350
300
50
100

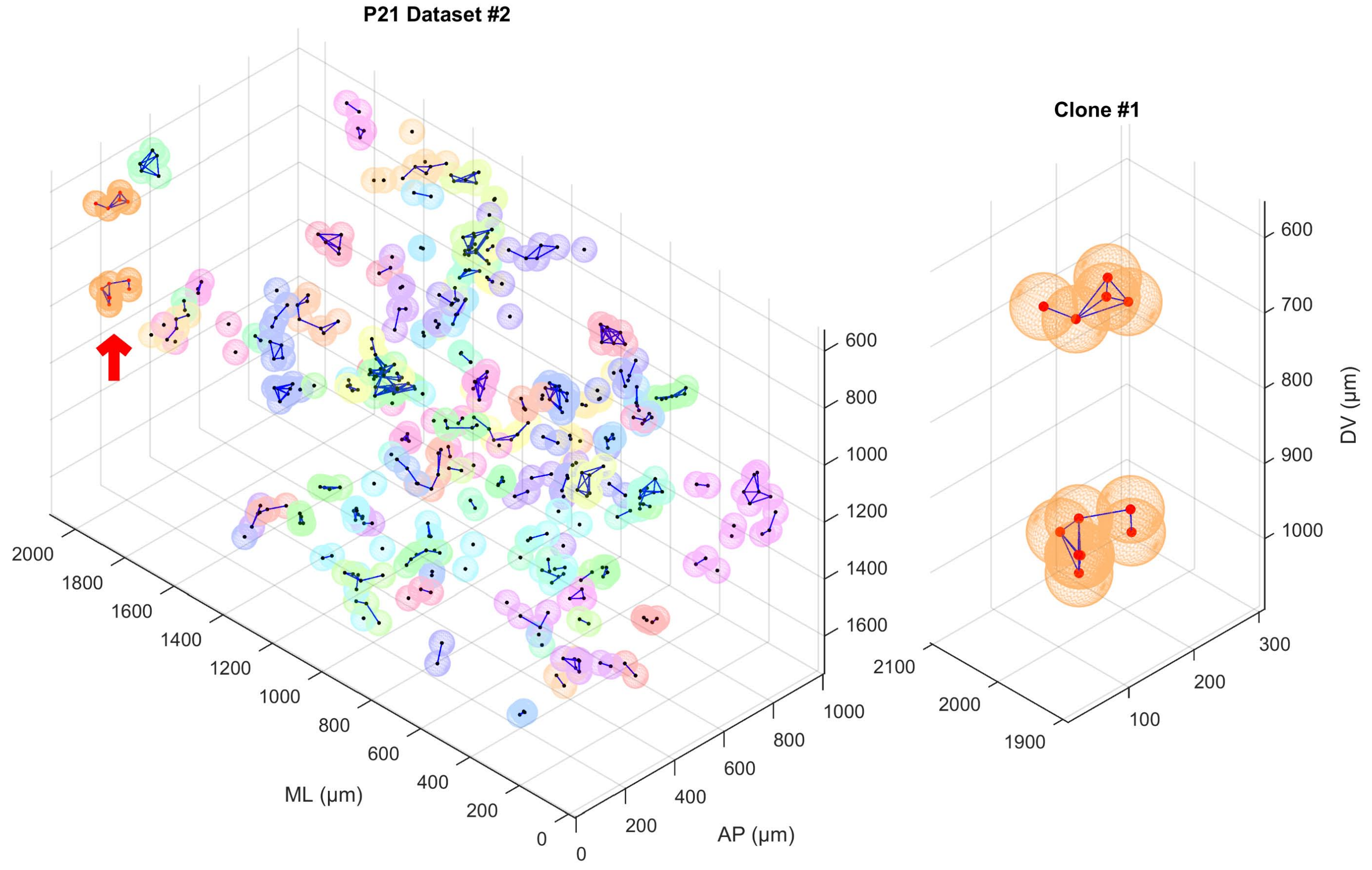
P21 Dataset #2
ML (µm)
2000
1800
1600
1400
1200
1000
800
600
400
200
0
AP (µm)
0
200
400
600
800
1000
600
800
1000
1200
1400
1600
Clone #1
DV (µm)
600
700
800
900
1000
2100
2000
1900
100
200
300

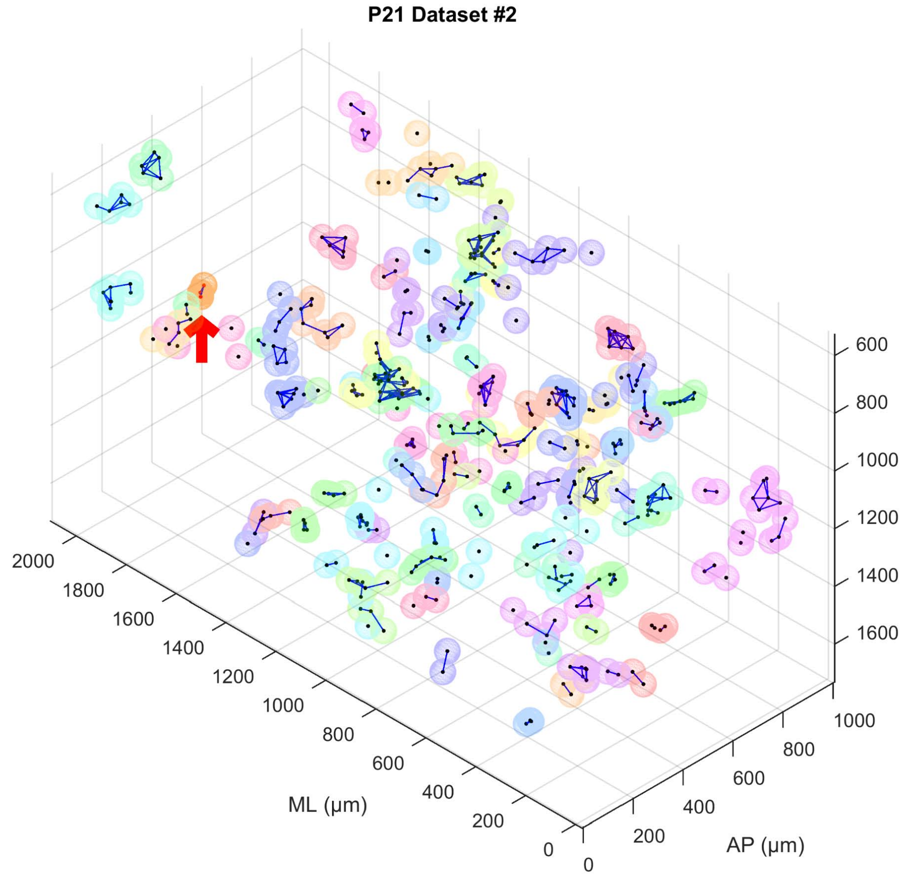
P21 Dataset #2
600
800
1000
1200
1400
1600
2000
1800
1600
1400
1200
1000
800
600
400
200
0
ML (µm)
0
200
400
600
800
1000
AP (µm)

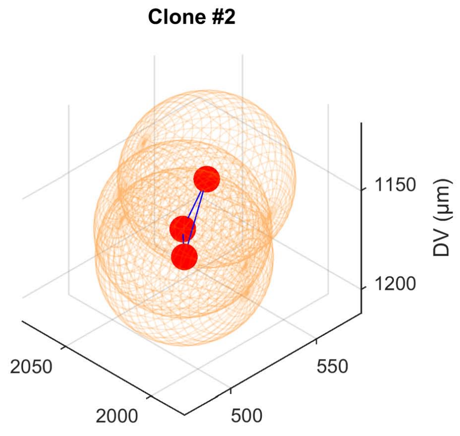
Clone #2
1150
1200
DV (µm)
2050
2000
500
550

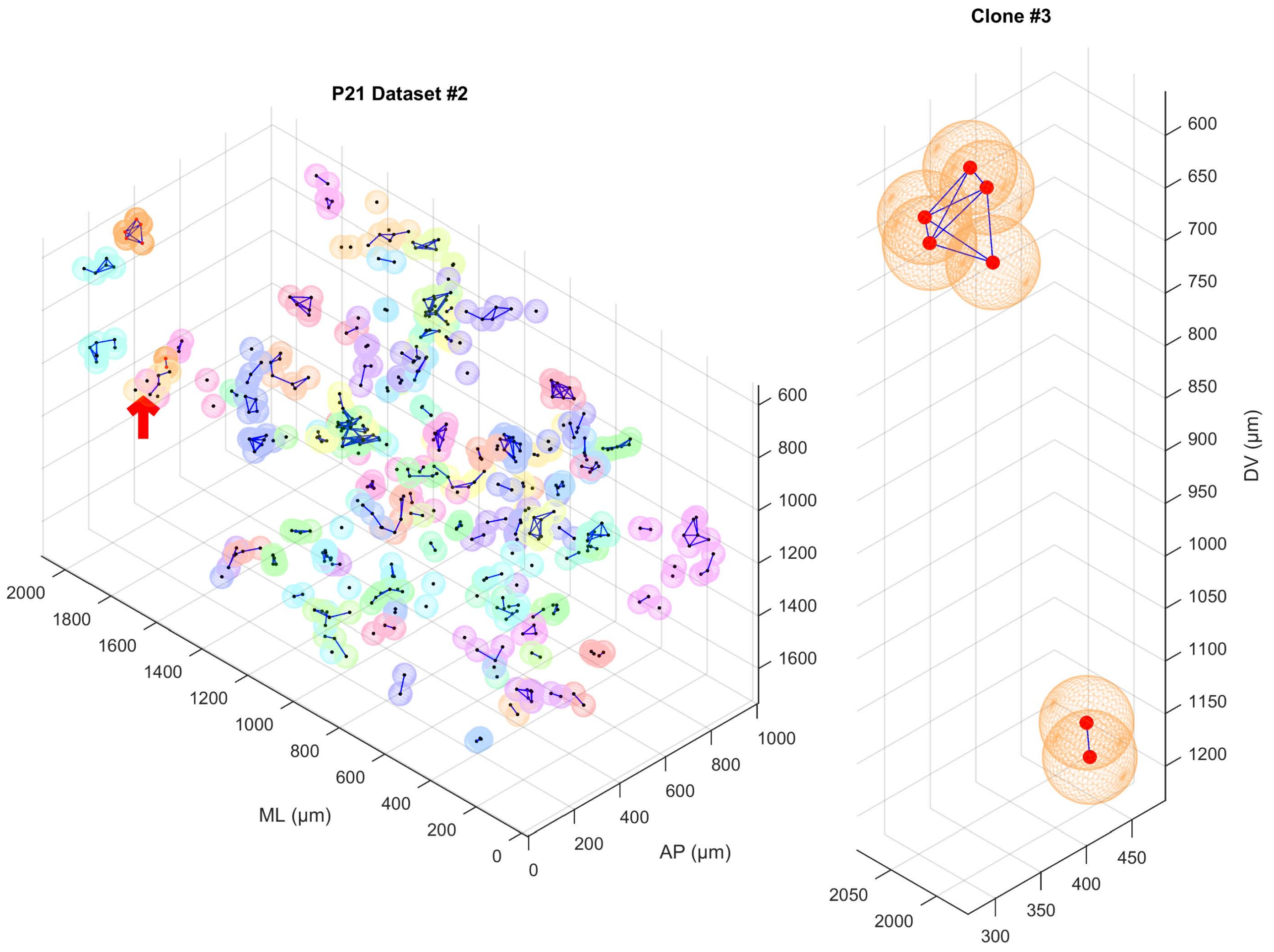
P21 Dataset #2
ML (µm)
AP (µm)
2000
1800
1600
1400
1200
1000
800
600
400
200
0
0
200
400
600
800
1000
600
800
1000
1200
1400
1600
Clone #3
DV (µm)
600
650
700
750
800
850
900
950
1000
1050
1100
1150
1200
2050
2000
300
350
400
450

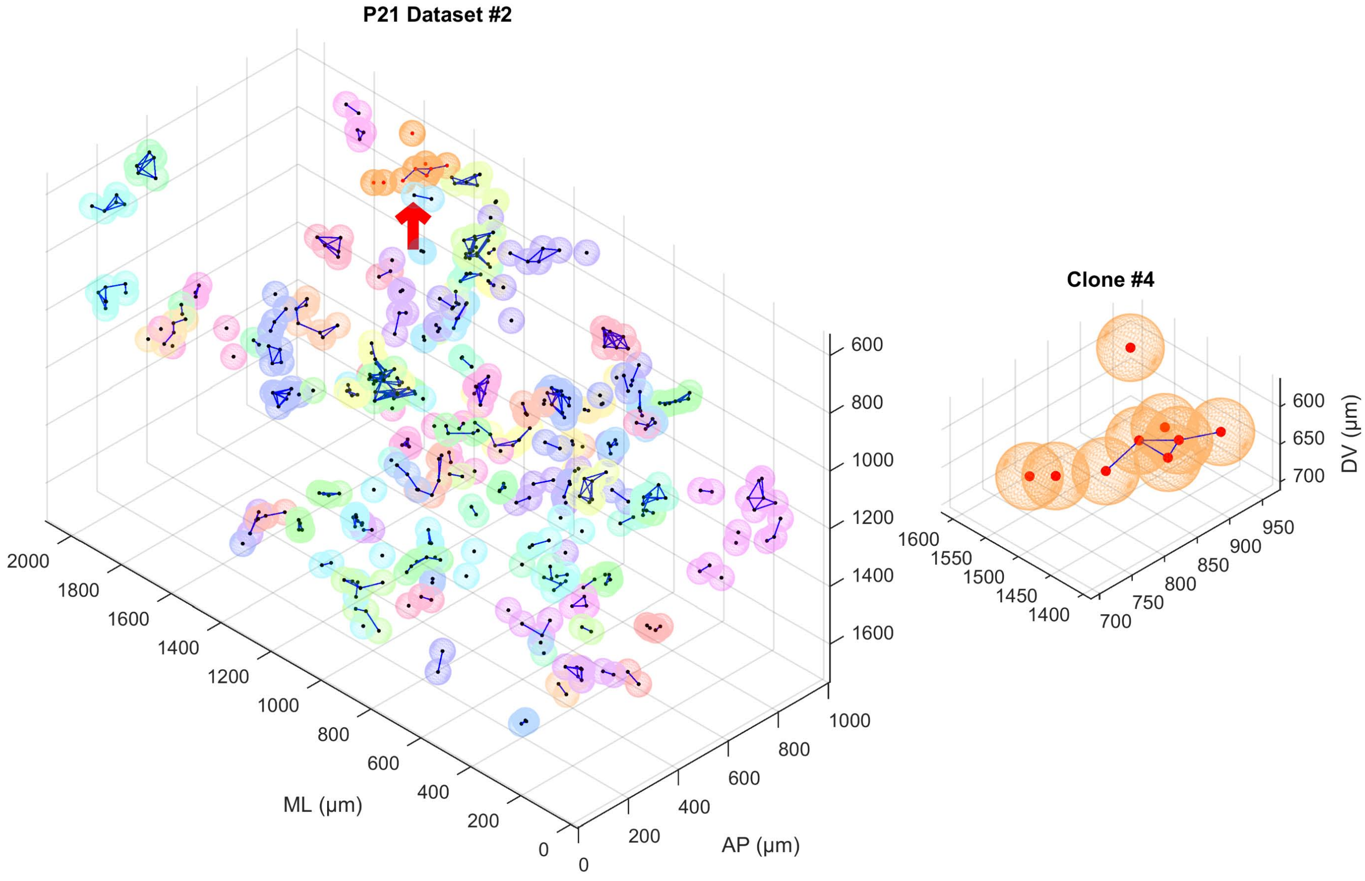
P21 Dataset #2
ML (µm)
AP (µm)
2000
1800
1600
1400
1200
1000
800
600
400
200
0
200
400
600
800
1000
600
800
1000
1200
1400
1600
Clone #4
DV (µm)
600
650
700
1600
1550
1500
1450
1400
700
750
800
850
900
950

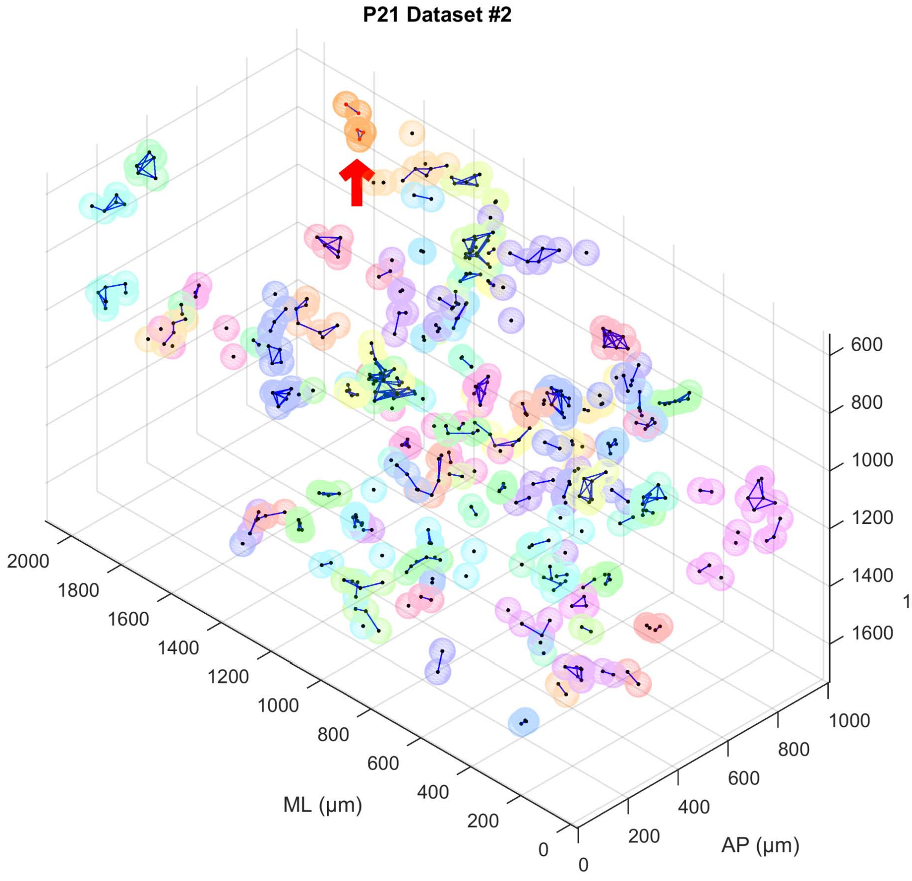

P21 Dataset #2
600
800
1000
1200
1400
1600
2000
1800
1600
1400
1200
1000
800
600
400
200
0
ML (µm)
0
200
400
600
800
1000
AP (µm)

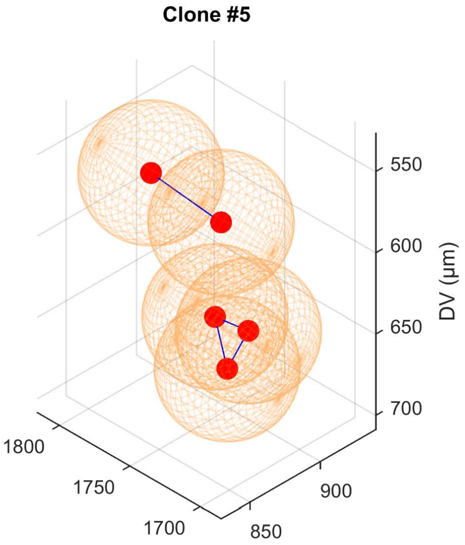

Clone #5
550
600
650
700
DV (µm)
1800
1750
1700
850
900

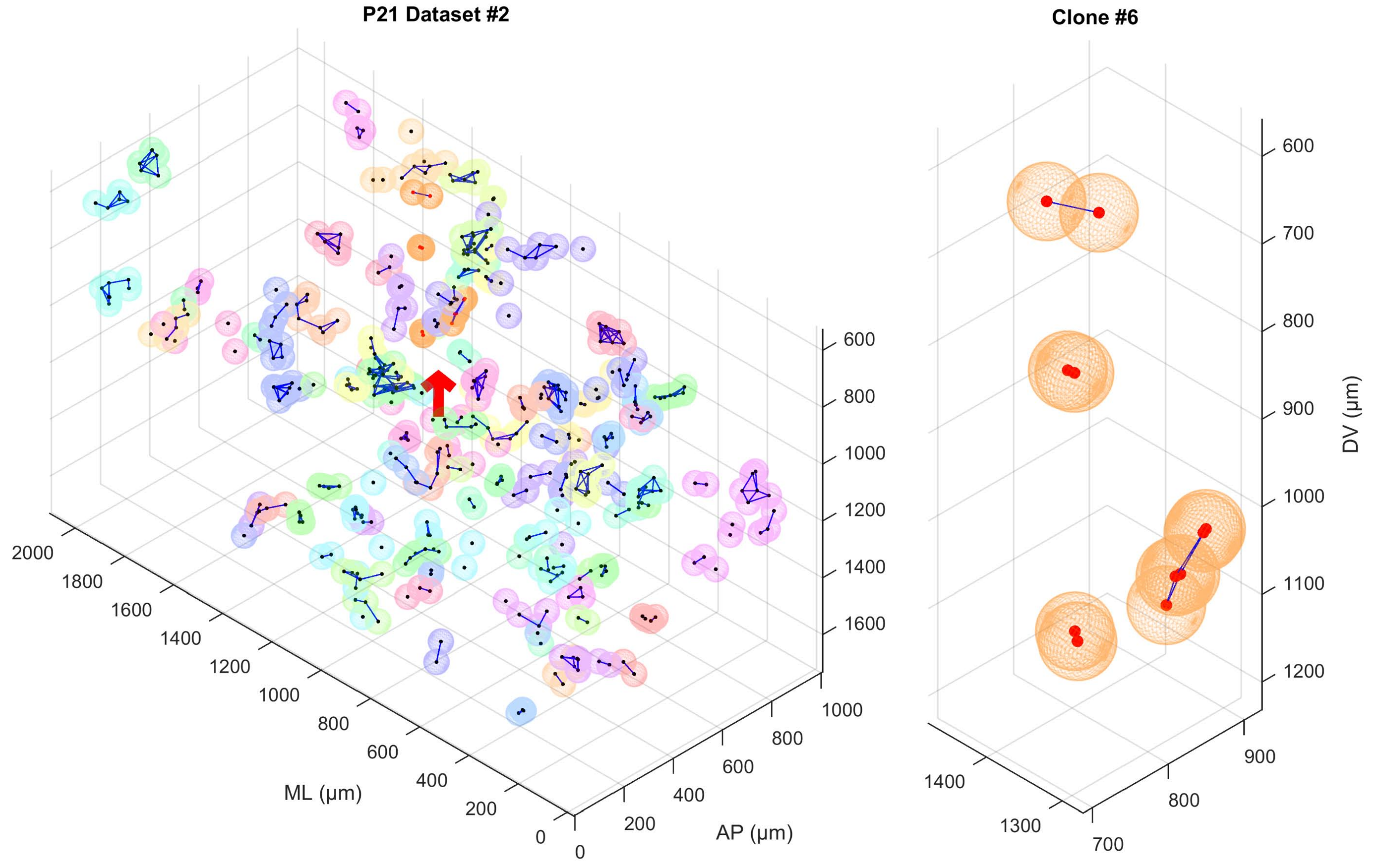

P21 Dataset #2
600
800
1000
1200
1400
1600
2000
1800
1600
1400
1200
1000
800
600
400
200
0
ML (μm)
0
200
400
600
800
1000
AP (μm)
Clone #6
600
700
800
900
1000
1100
1200
DV (μm)
1400
1300
700
800
900

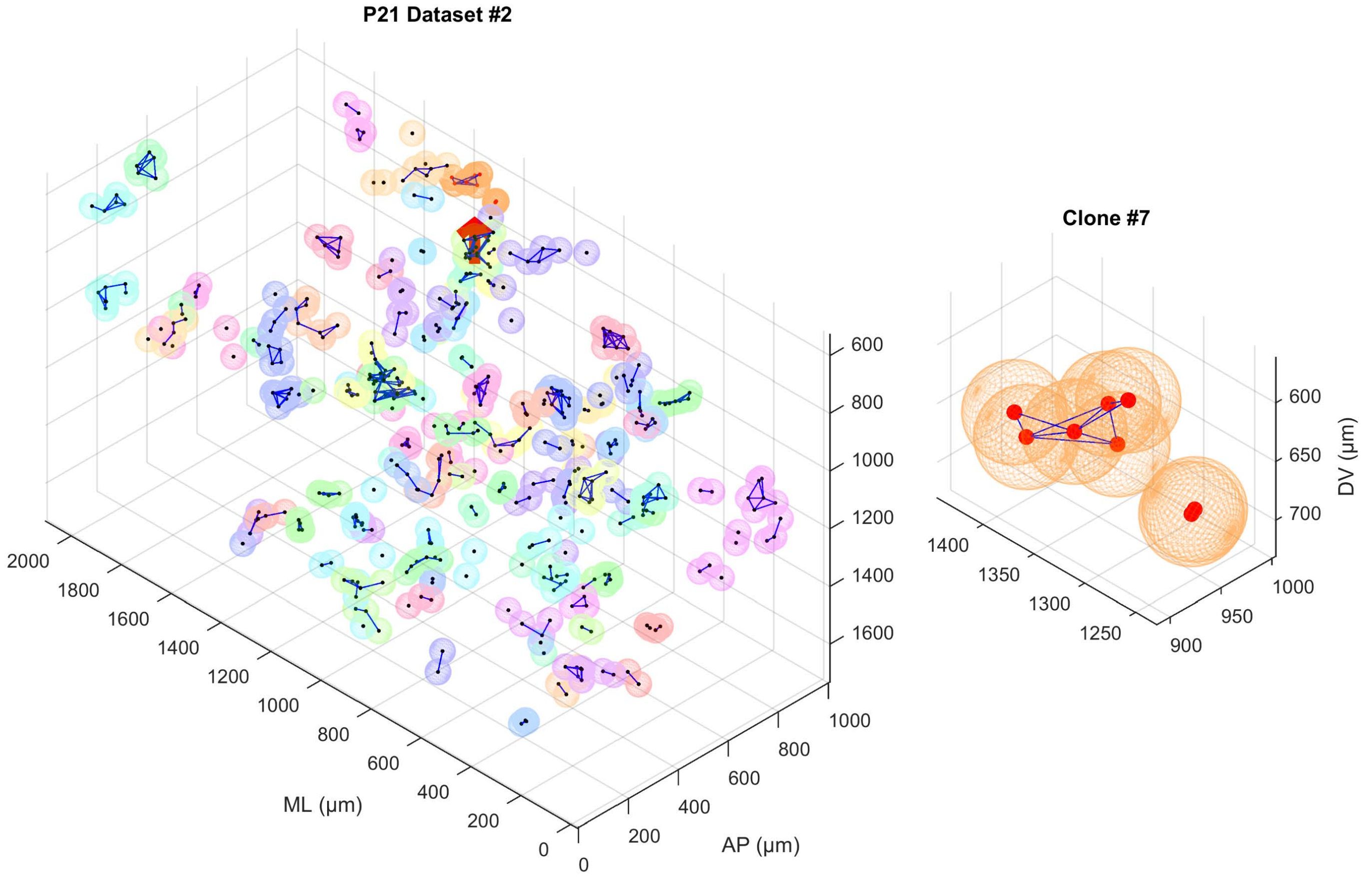

P21 Dataset #2
ML (µm)
AP (µm)
2000
1800
1600
1400
1200
1000
800
600
400
200
0
0
200
400
600
800
1000
600
800
1000
1200
1400
1600
Clone #7
DV (µm)
1400
1350
1300
1250
900
950
1000
600
650
700

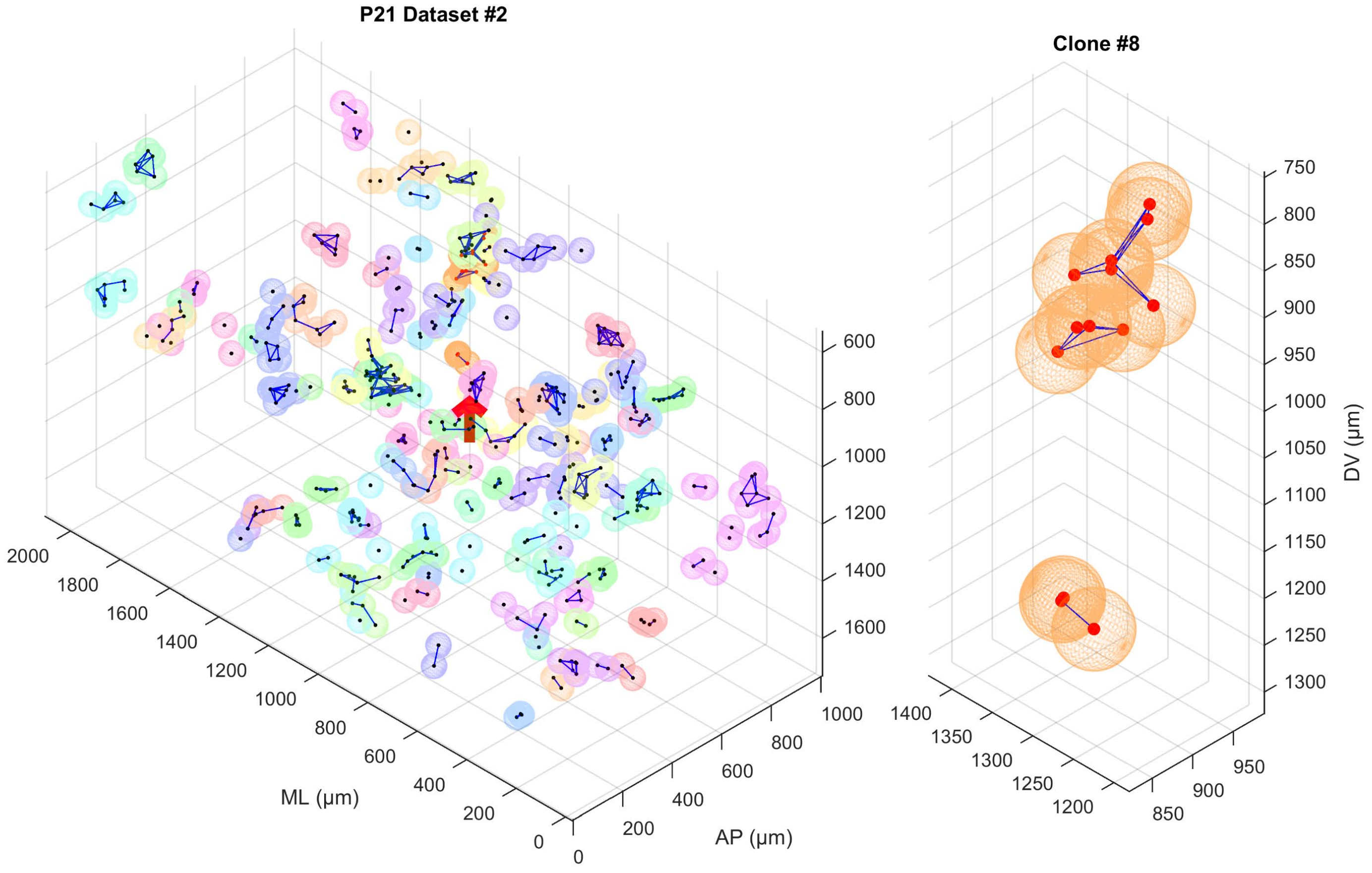

P21 Dataset #2
Clone #8
ML (μm)
AP (μm)
DV (μm)
2000
1800
1600
1400
1200
1000
800
600
400
200
0
600
800
1000
1200
1400
1600
750
800
850
900
950
1000
1050
1100
1150
1200
1250
1300
1400
1350
1300
1250
1200
850
900
950

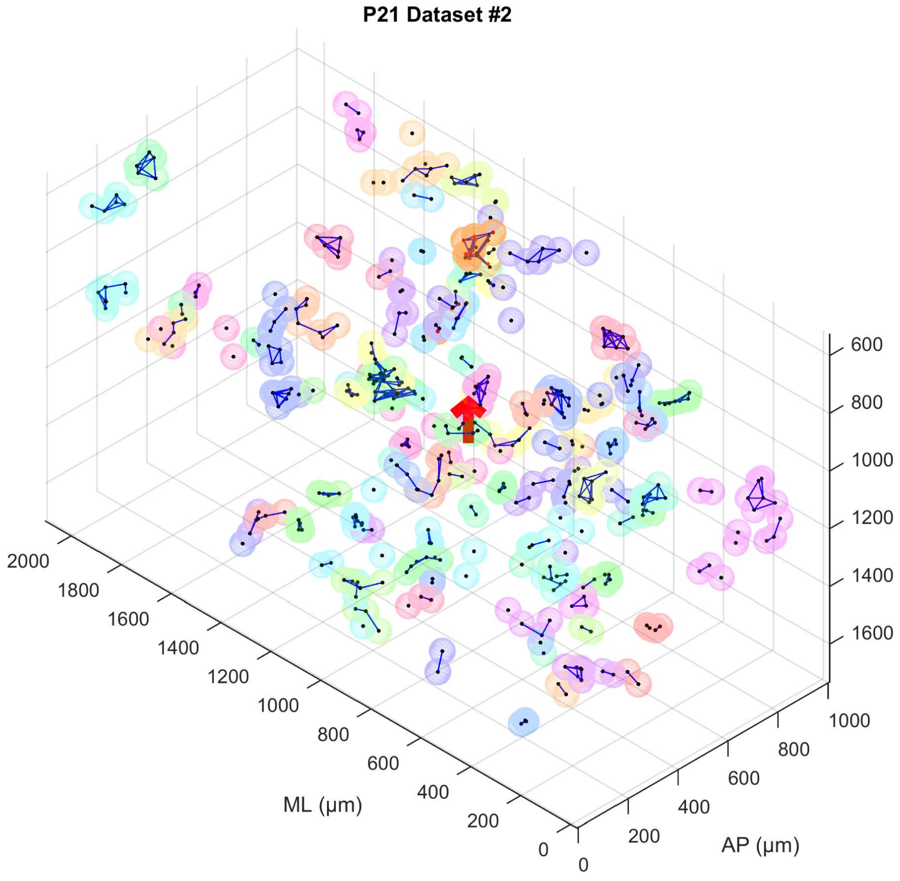
P21 Dataset #2
600
800
1000
1200
1400
1600
2000
1800
1600
1400
1200
1000
800
600
400
200
0
ML (μm)
0
200
400
600
800
1000
AP (μm)

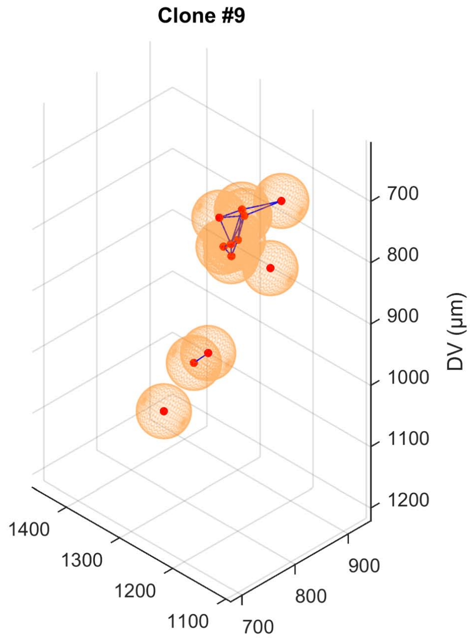
Clone #9
700
800
900
1000
1100
1200
DV (μm)
1400
1300
1200
1100
700
800
900

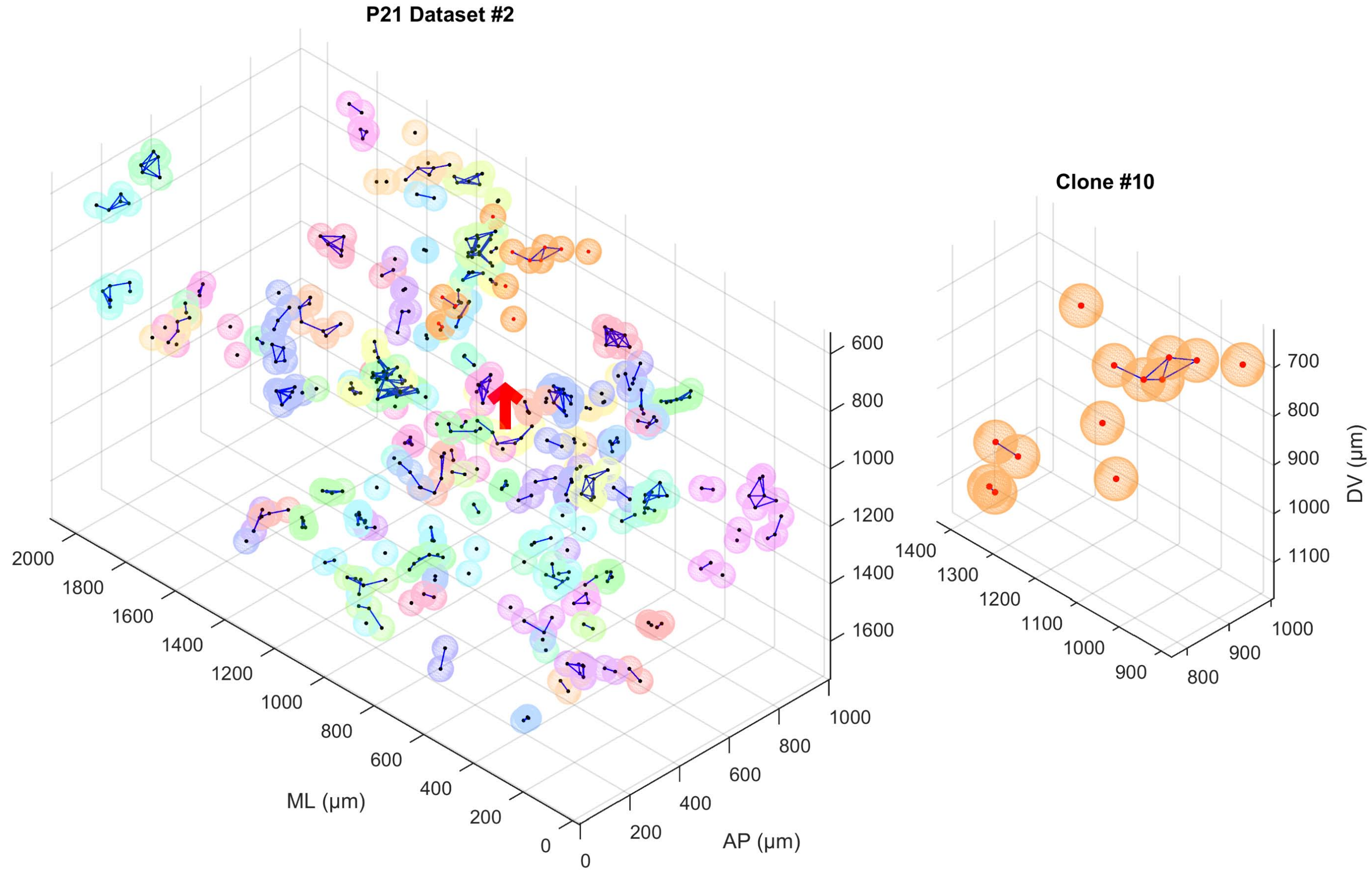

P21 Dataset #2
ML (µm)
AP (µm)
2000
1800
1600
1400
1200
1000
800
600
400
200
0
0
200
400
600
800
1000
600
800
1000
1200
1400
1600
Clone #10
DV (µm)
1400
1300
1200
1100
1000
900
800
900
1000
700
800
900
1000
1100

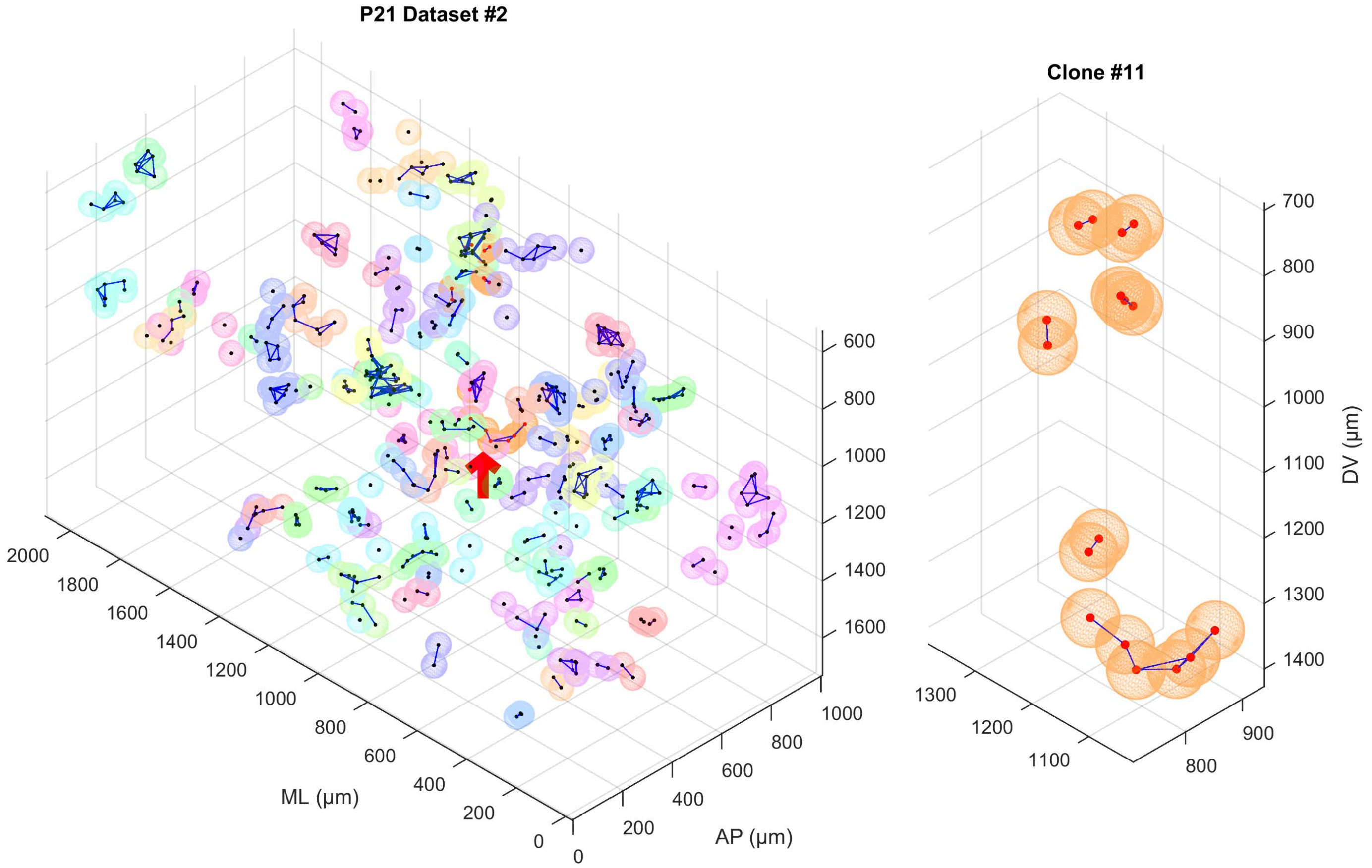

P21 Dataset #2
Clone #11
ML (μm)
AP (μm)
DV (μm)
2000
1800
1600
1400
1200
1000
800
600
400
200
0
0
200
400
600
800
1000
600
800
1000
1200
1400
1600
1300
1200
1100
800
900
700
800
900
1000
1100
1200
1300
1400

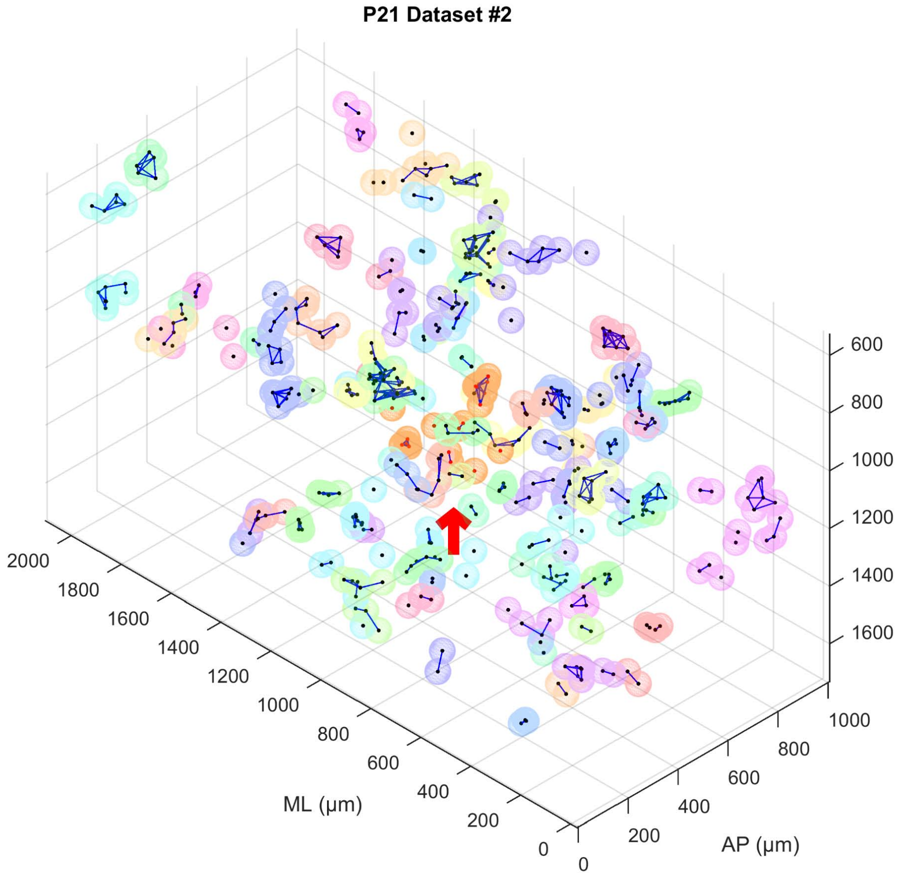
P21 Dataset #2
600
800
1000
1200
1400
1600
2000
1800
1600
1400
1200
1000
800
600
400
200
0
ML (µm)
0
200
400
600
800
1000
AP (µm)

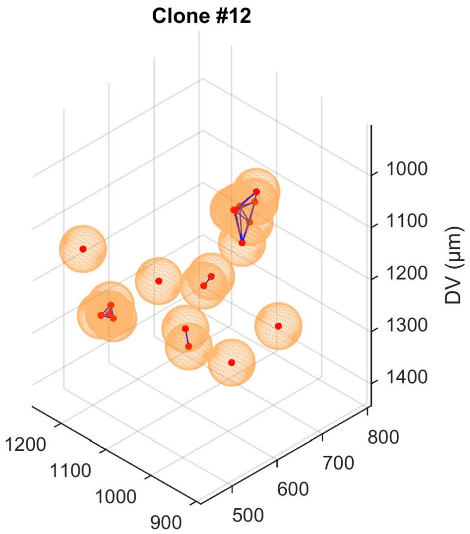
Clone #12
1000
1100
1200
1300
1400
DV (µm)
1200
1100
1000
900
500
600
700
800

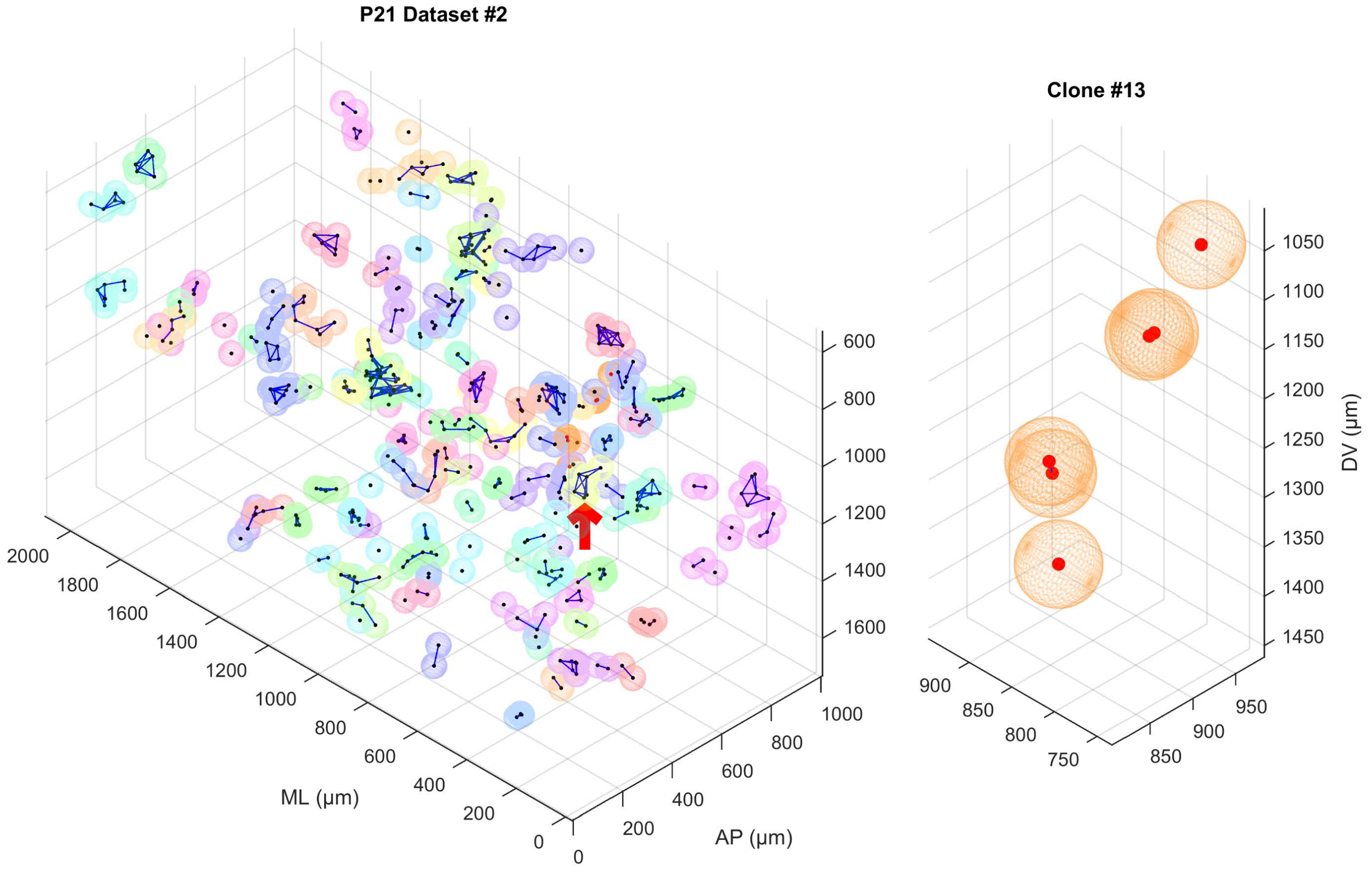

P21 Dataset #2
ML (µm)
AP (µm)
2000
1800
1600
1400
1200
1000
800
600
400
200
0
0
200
400
600
800
1000
600
800
1000
1200
1400
1600
Clone #13
DV (µm)
1050
1100
1150
1200
1250
1300
1350
1400
1450
900
850
800
750
850
900
950

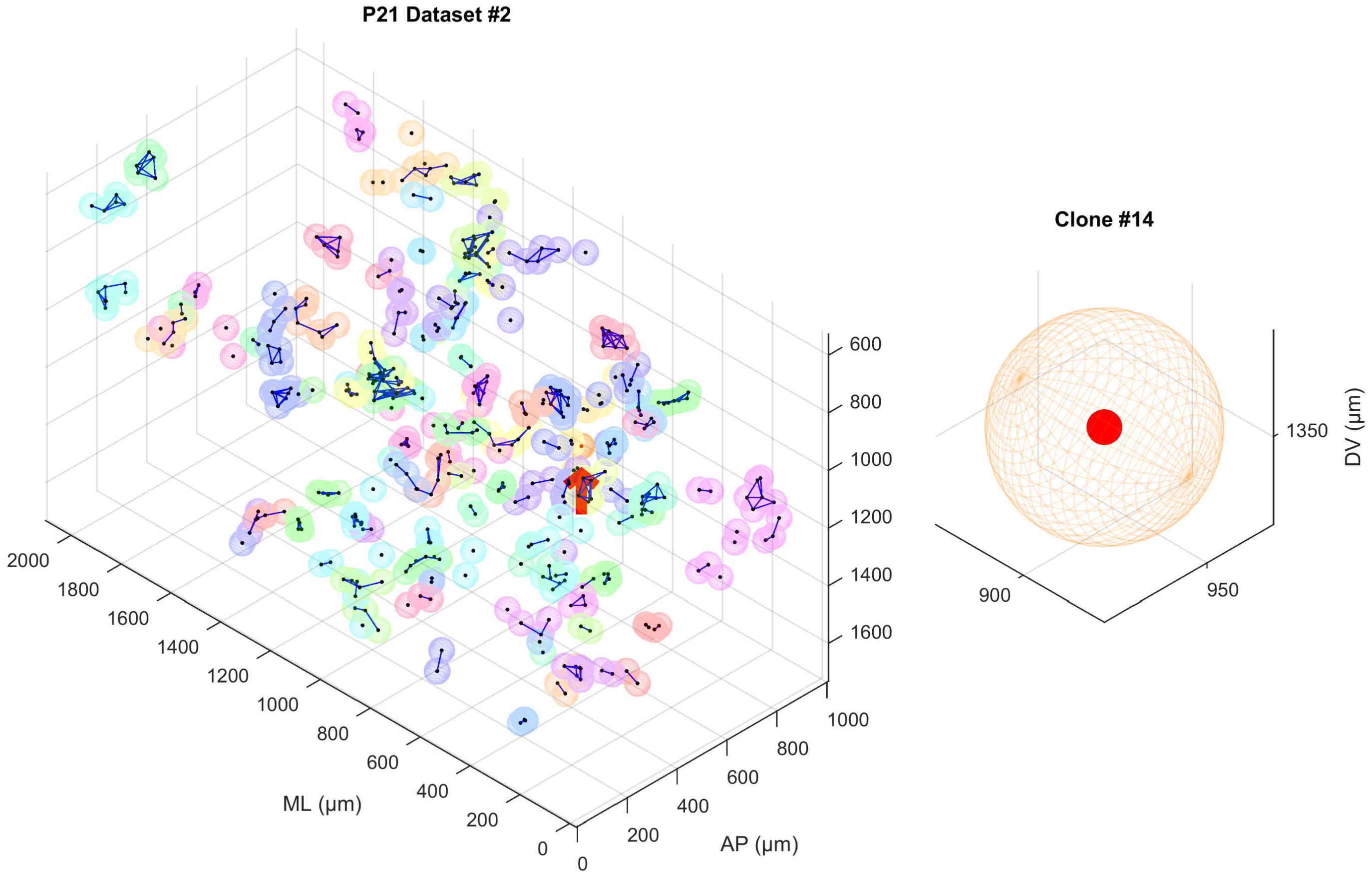

P21 Dataset #2
600
800
1000
1200
1400
1600
2000
1800
1600
1400
1200
1000
800
600
400
200
0
ML (µm)
0
200
400
600
800
1000
AP (µm)
Clone #14
1350
DV (µm)
900
950

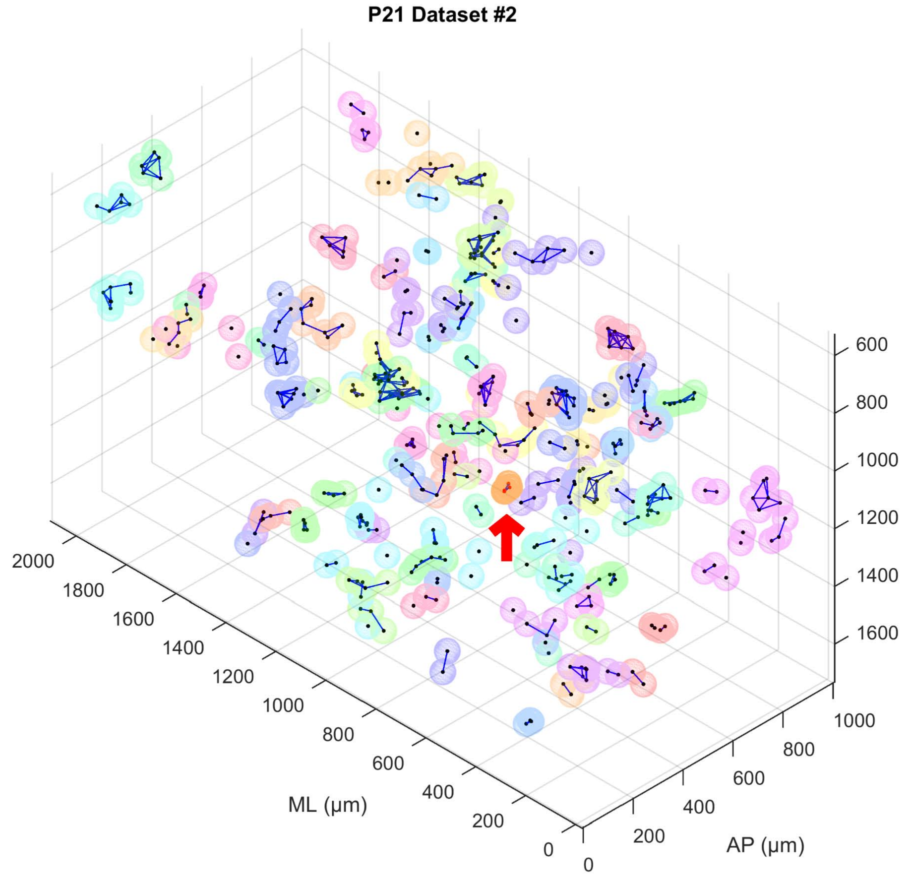
P21 Dataset #2
600
800
1000
1200
1400
1600
2000
1800
1600
1400
1200
1000
800
600
400
200
0
ML (µm)
0
200
400
600
800
1000
AP (µm)

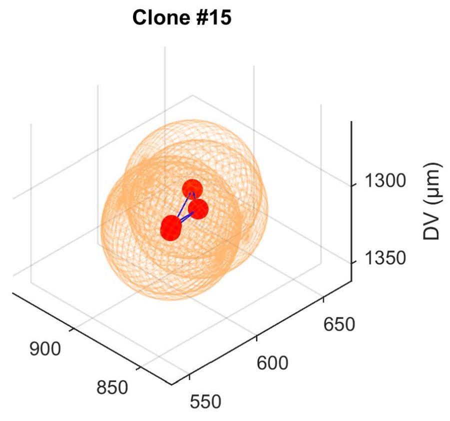
Clone #15
1300
1350
DV (µm)
900
850
550
600
650

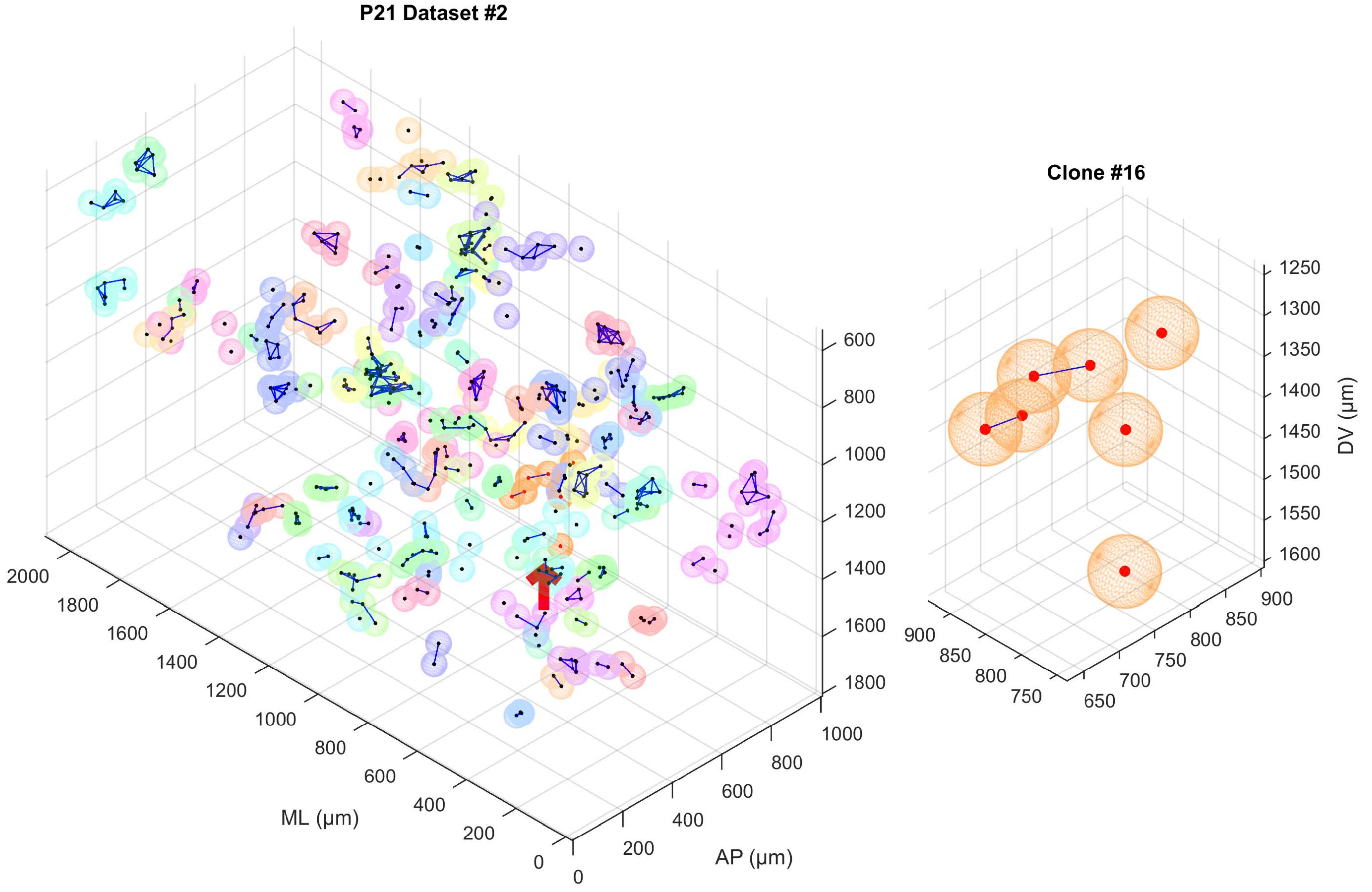

P21 Dataset #2
600
800
1000
1200
1400
1600
1800
2000
1800
1600
1400
1200
1000
800
600
400
200
0
ML (µm)
0
200
400
600
800
1000
AP (µm)
Clone #16
1250
1300
1350
1400
1450
1500
1550
1600
DV (µm)
900
850
800
750
650
700
750
800
850
900

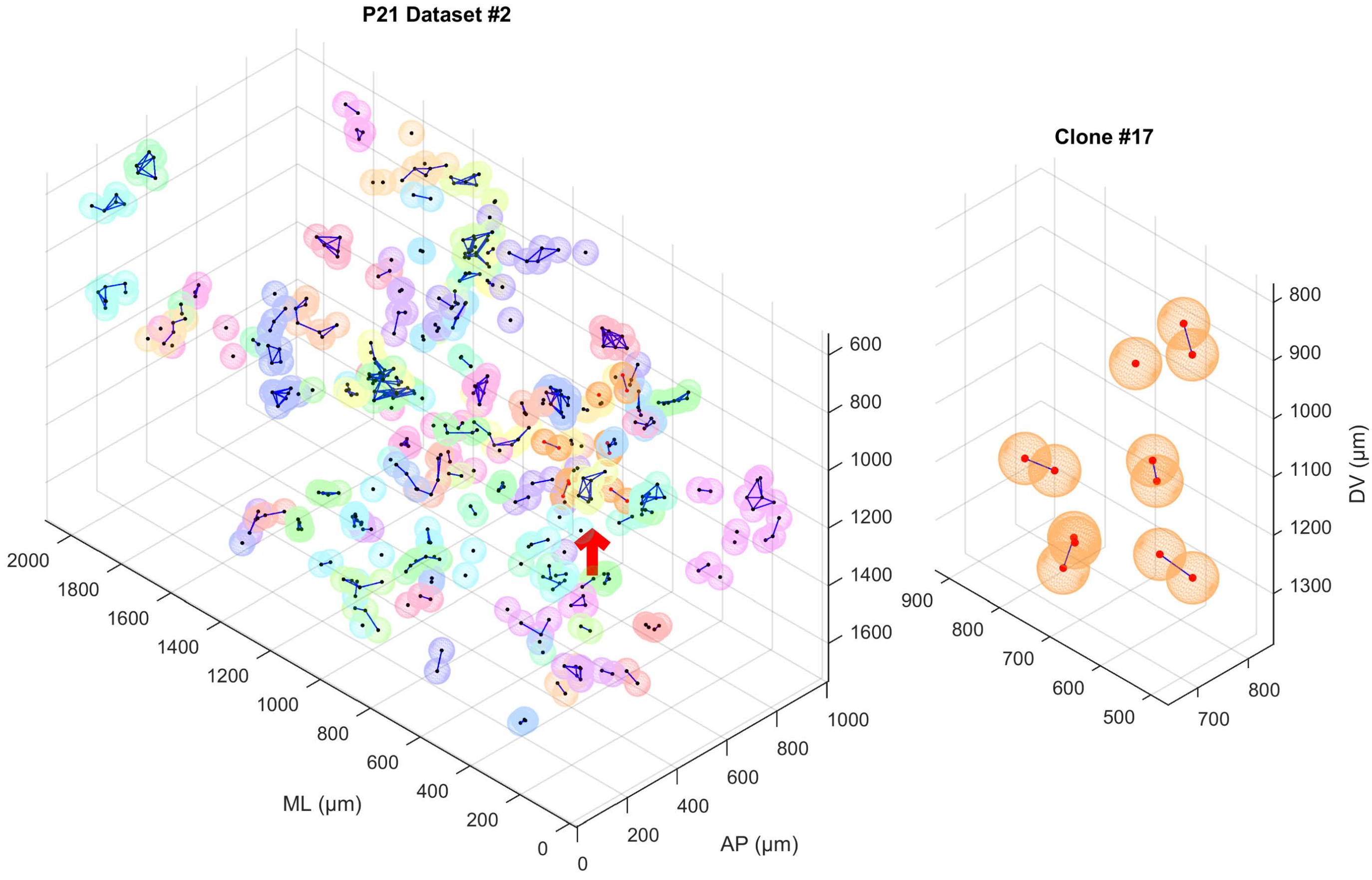

P21 Dataset #2
Clone #17
ML (µm)
AP (µm)
DV (µm)
2000
1800
1600
1400
1200
1000
800
600
400
200
0

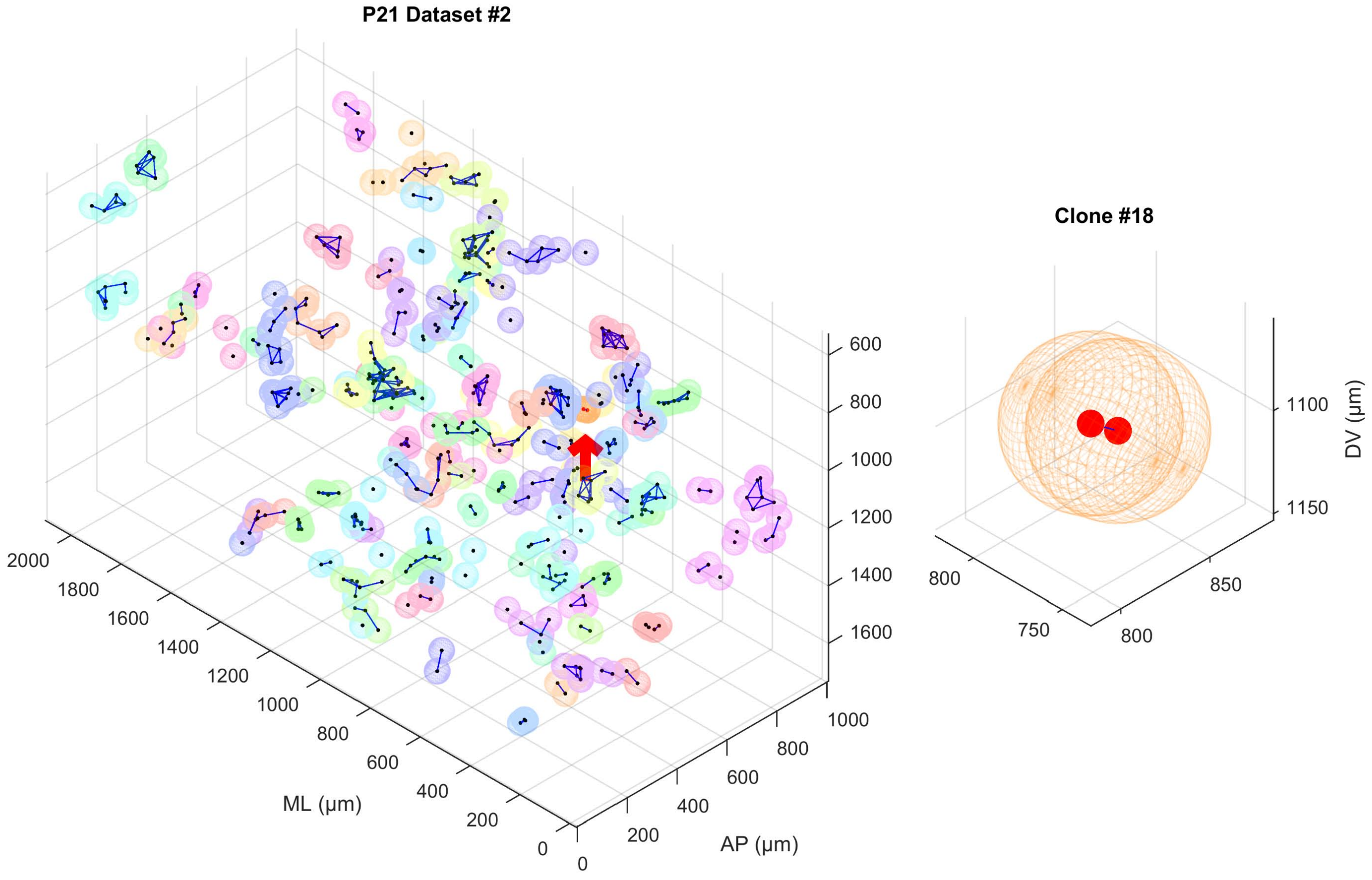
P21 Dataset #2
ML (µm)
AP (µm)
2000
1800
1600
1400
1200
1000
800
600
400
200
0
0
200
400
600
800
1000
600
800
1000
1200
1400
1600
Clone #18
DV (µm)
1100
1150
800
750
800
850

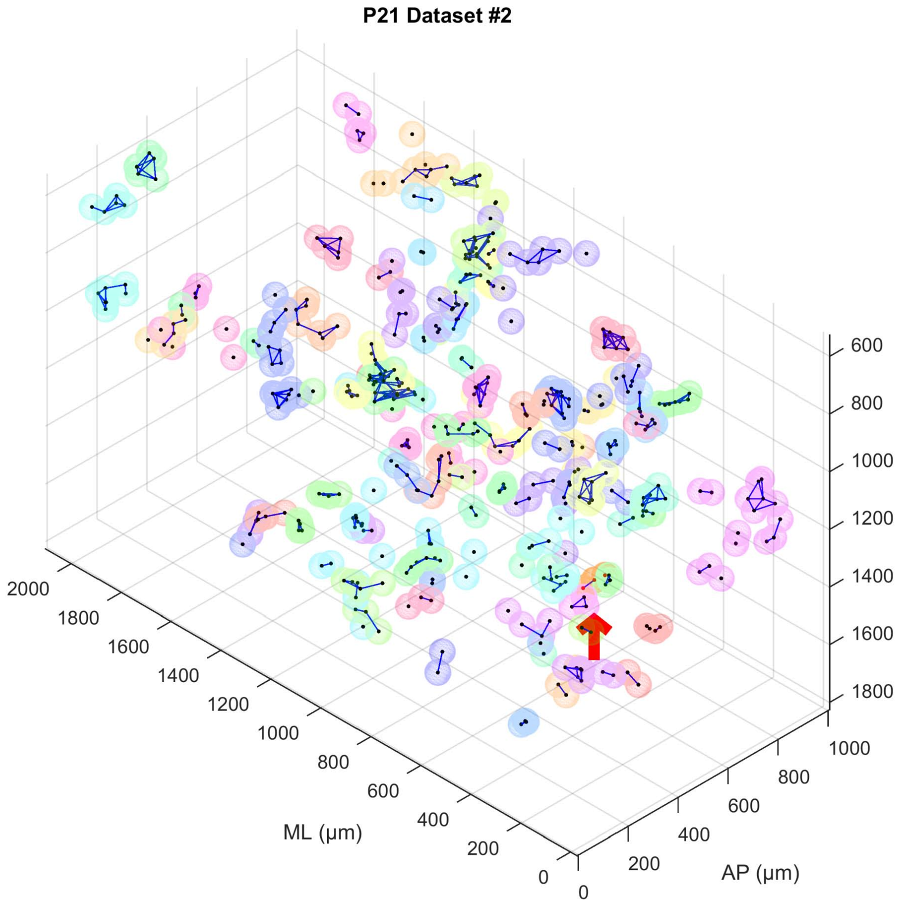
P21 Dataset #2
ML (µm)
AP (µm)
2000
1800
1600
1400
1200
1000
800
600
400
200
0
0
200
400
600
800
1000
600
800
1000
1200
1400
1600
1800

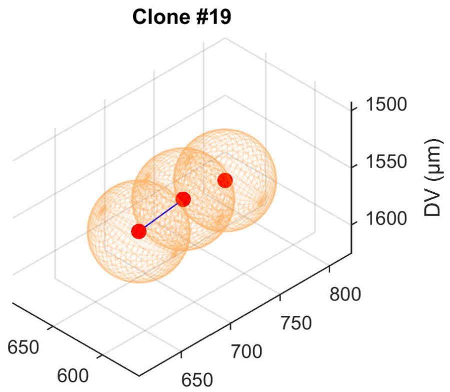
Clone #19
DV (µm)
1500
1550
1600
650
600
650
700
750
800

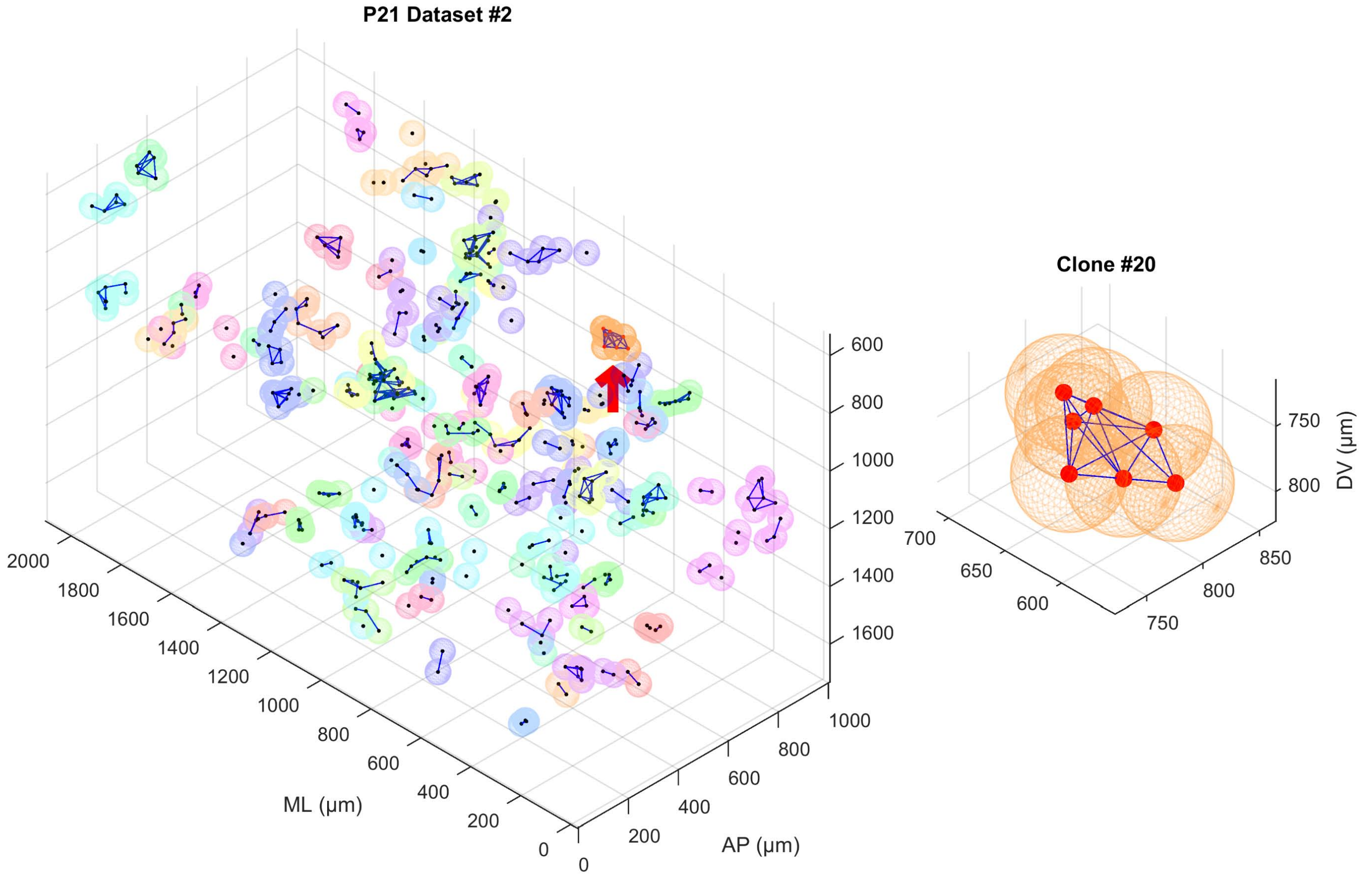

P21 Dataset #2
ML (µm)
2000
1800
1600
1400
1200
1000
800
600
400
200
0
AP (µm)
0
200
400
600
800
1000
600
800
1000
1200
1400
1600
Clone #20
700
650
600
750
800
850
750
800
DV (µm)

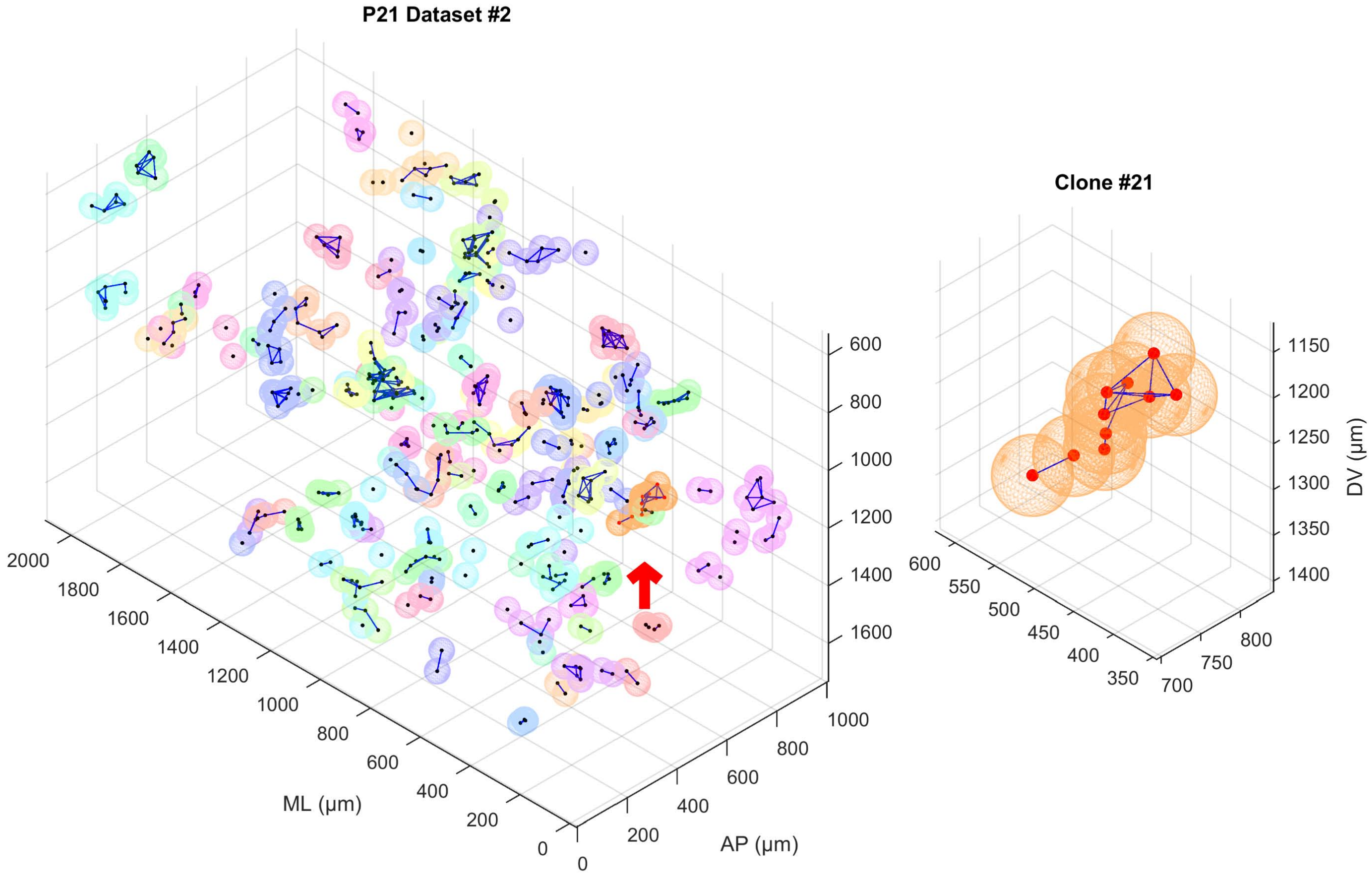

P21 Dataset #2
ML (µm)
AP (µm)
2000
1800
1600
1400
1200
1000
800
600
400
200
0
0
200
400
600
800
1000
600
800
1000
1200
1400
1600
Clone #21
DV (µm)
1150
1200
1250
1300
1350
1400
600
550
500
450
400
350
700
750
800

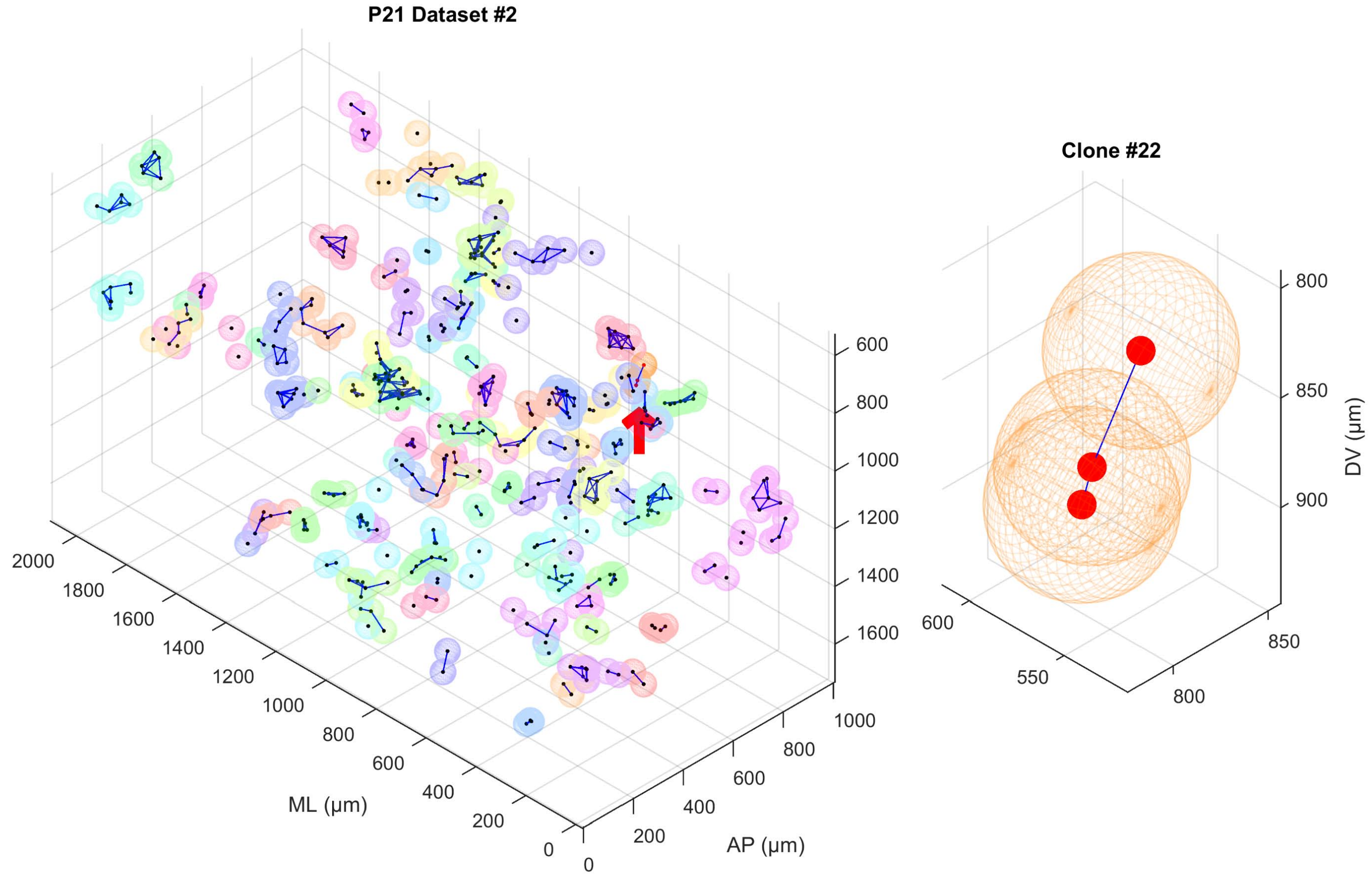

P21 Dataset #2
600
800
1000
1200
1400
1600
2000
1800
1600
1400
1200
1000
800
600
400
200
0
ML (µm)
0
200
400
600
800
1000
AP (µm)
Clone #22
800
850
900
DV (µm)
600
550
800
850

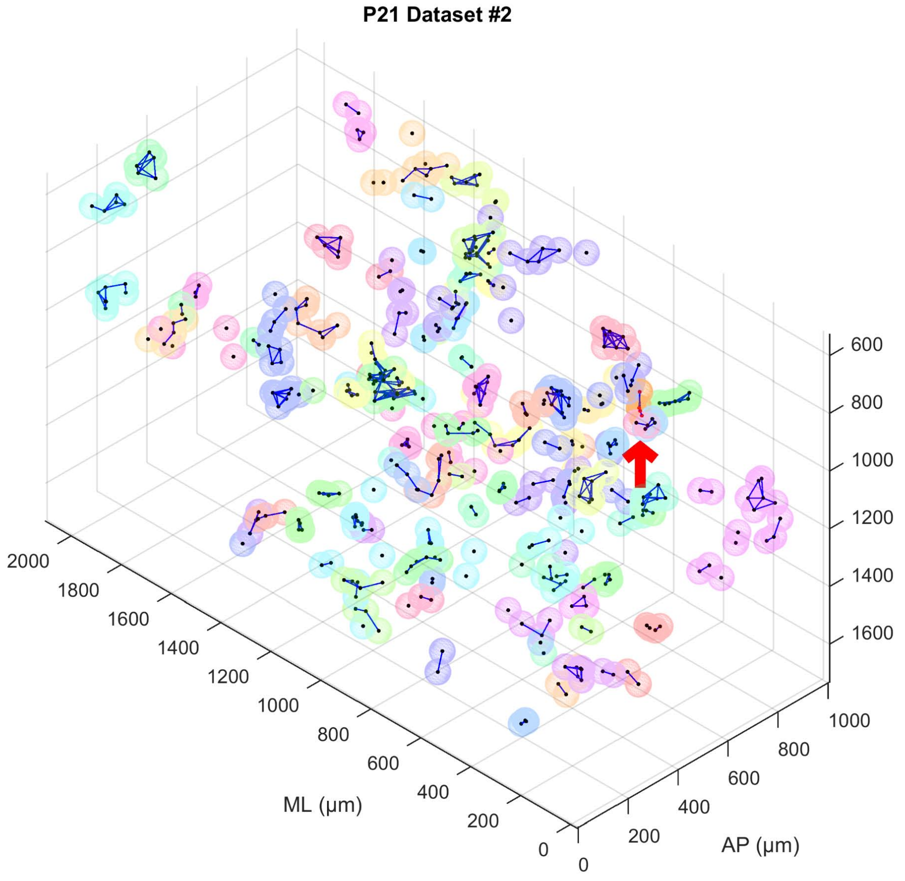

P21 Dataset #2
600
800
1000
1200
1400
1600
2000
1800
1600
1400
1200
1000
800
600
400
200
0
ML (µm)
0
200
400
600
800
1000
AP (µm)

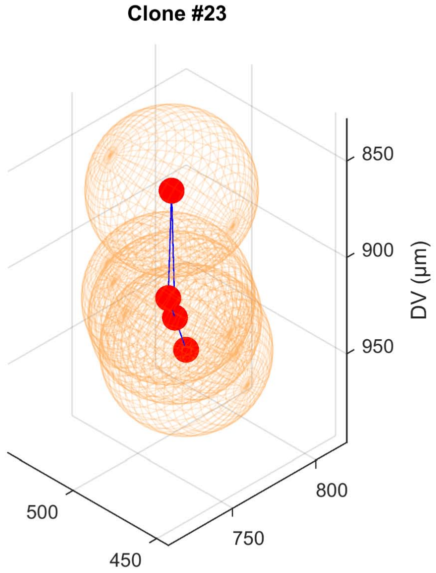

Clone #23
850
900
950
DV (µm)
500
450
750
800

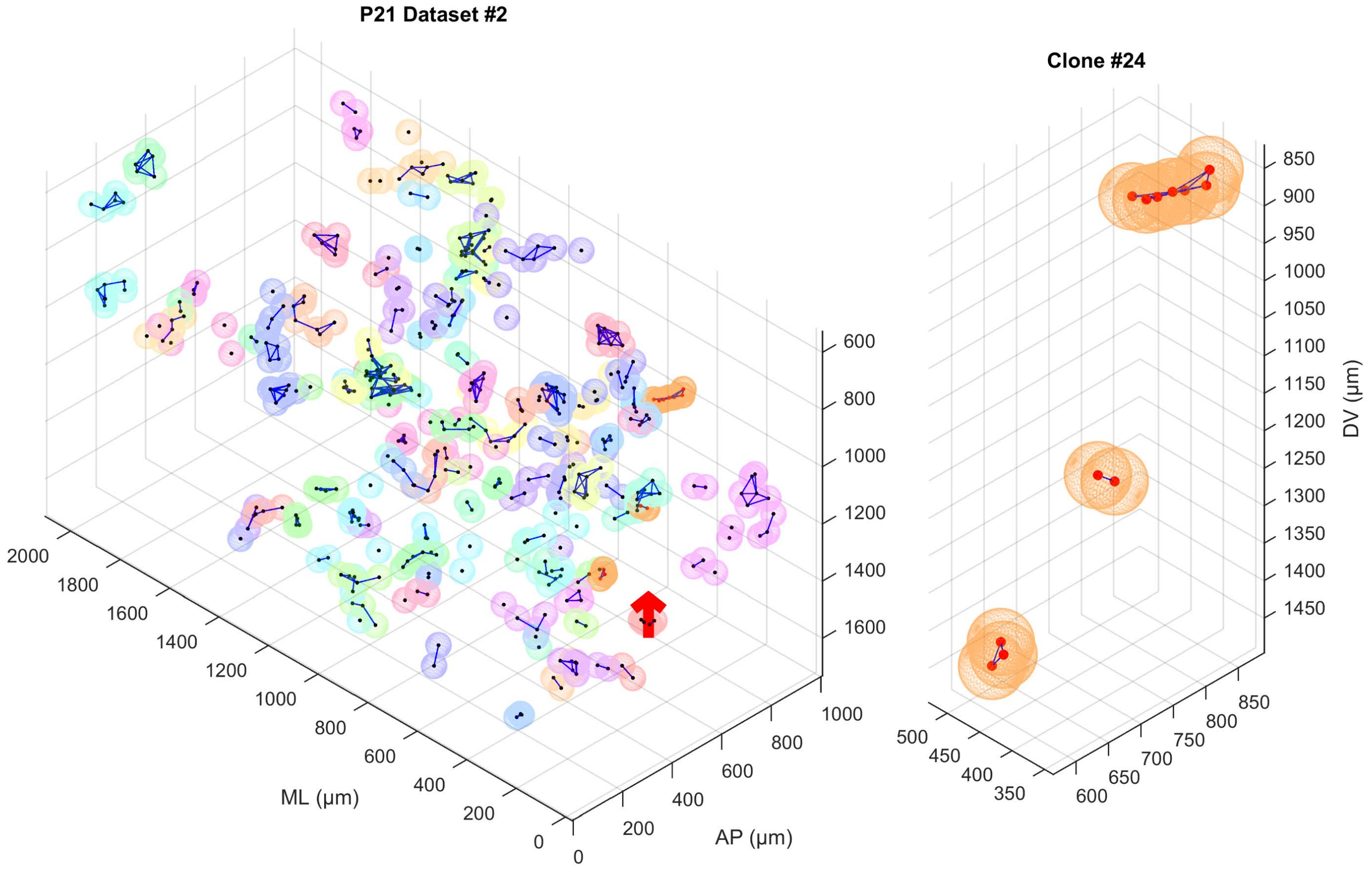

P21 Dataset #2
Clone #24
ML (µm)
AP (µm)
DV (µm)

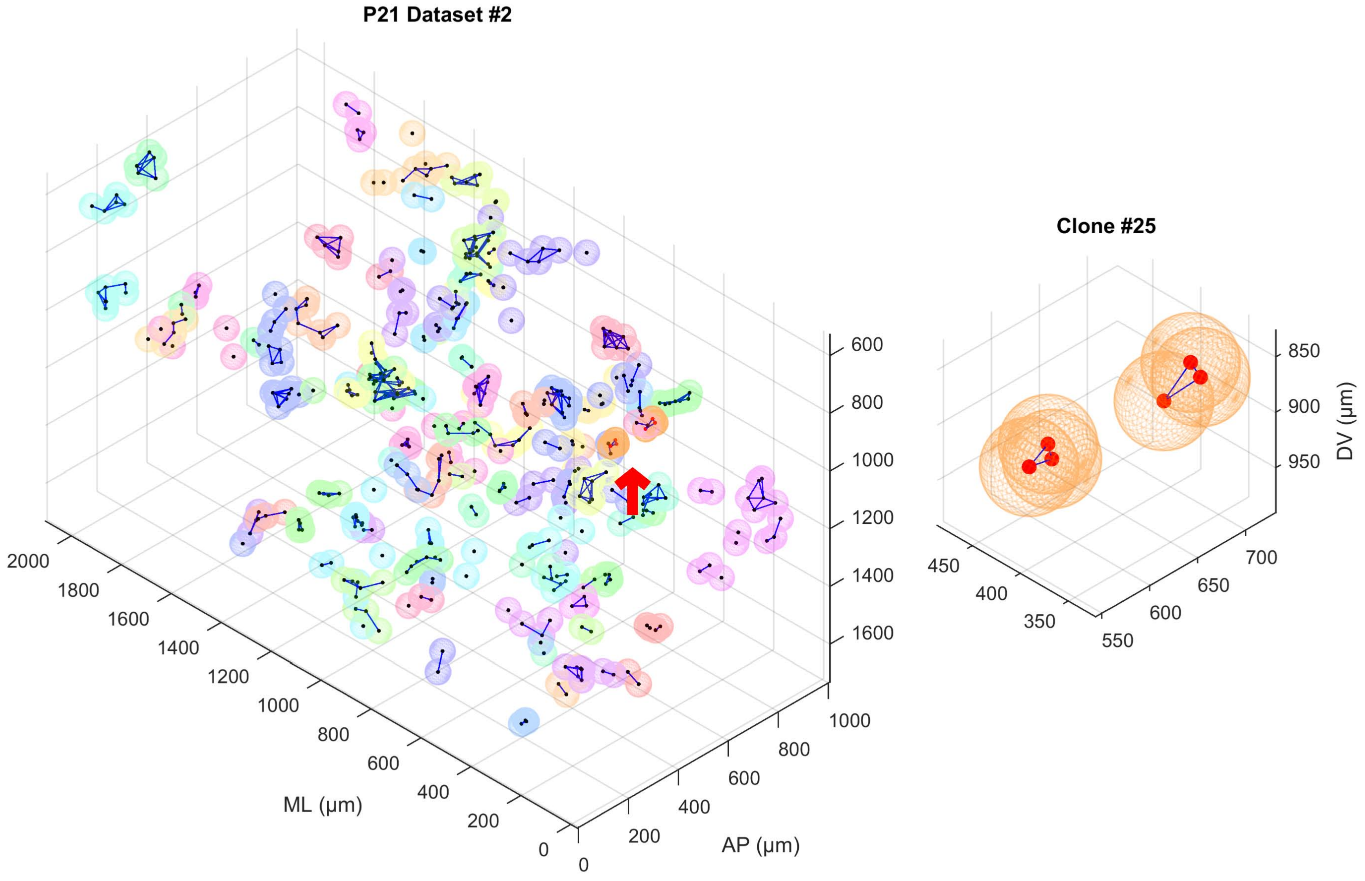
P21 Dataset #2
600
800
1000
1200
1400
1600
2000
1800
1600
1400
1200
1000
800
600
400
200
0
ML (µm)
0
200
400
600
800
1000
AP (µm)
Clone #25
850
900
950
DV (µm)
450
400
350
550
600
650
700

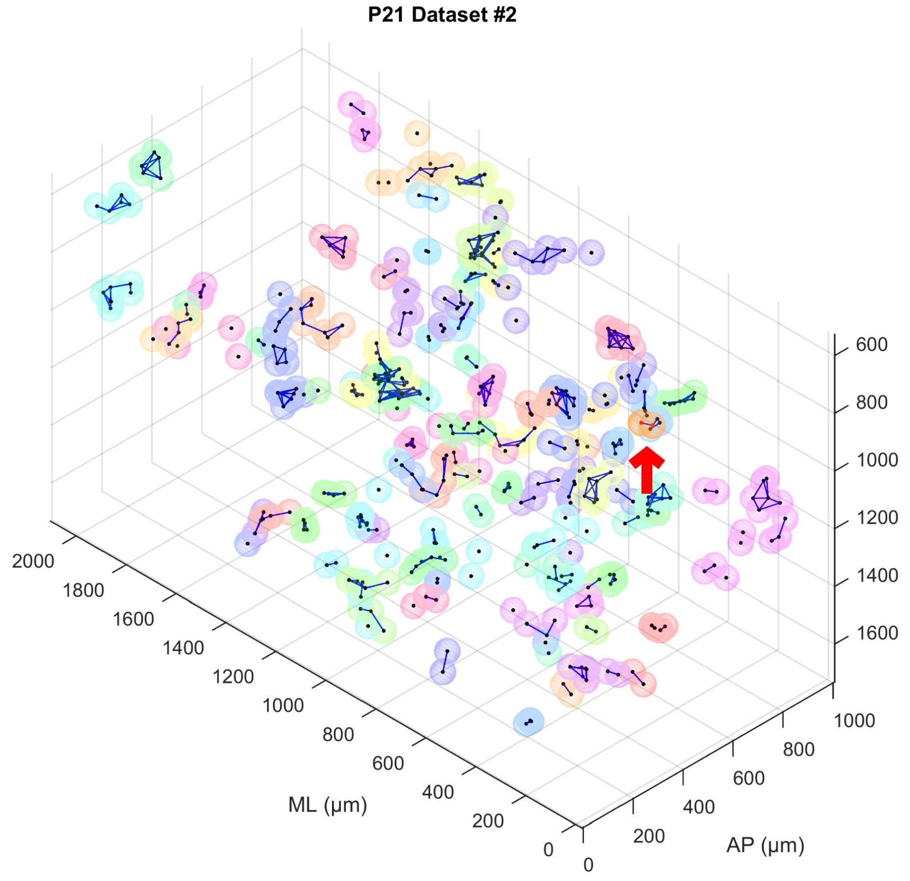

P21 Dataset #2
600
800
1000
1200
1400
1600
2000
1800
1600
1400
1200
1000
800
600
400
200
0
ML (µm)
0
200
400
600
800
1000
AP (µm)

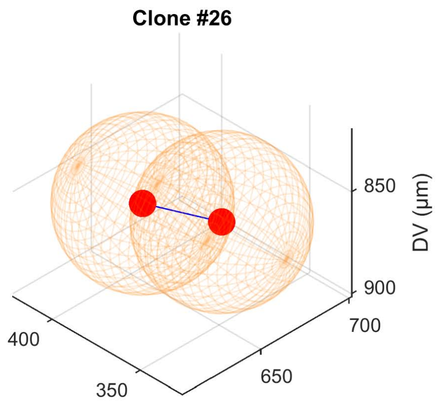

Clone #26
850
900
DV (µm)
400
350
650
700

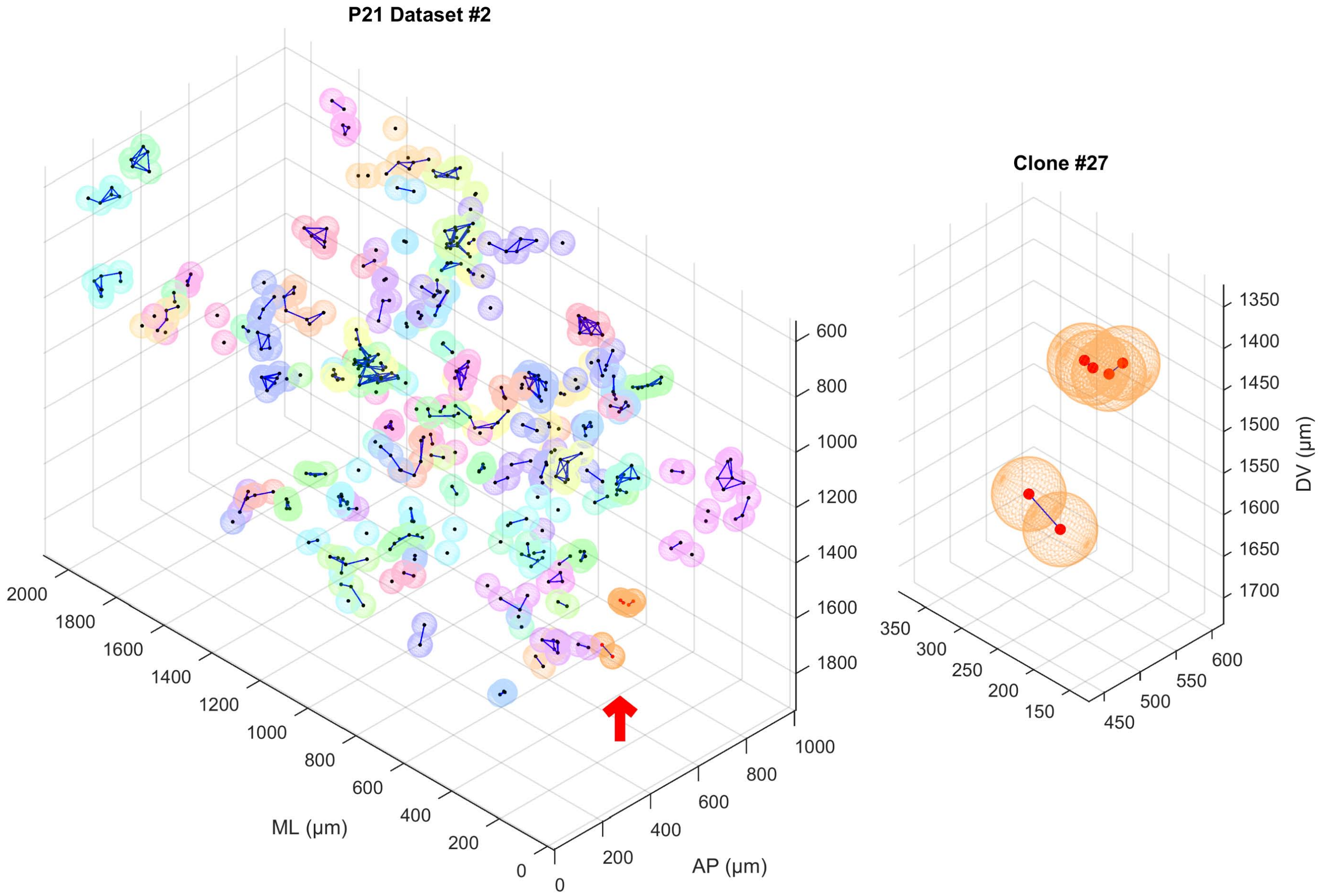

P21 Dataset #2
600
800
1000
1200
1400
1600
1800
2000
1800
1600
1400
1200
1000
800
600
400
200
0
ML (µm)
0
200
400
600
800
1000
AP (µm)
Clone #27
1350
1400
1450
1500
1550
1600
1650
1700
DV (µm)
350
300
250
200
150
450
500
550
600

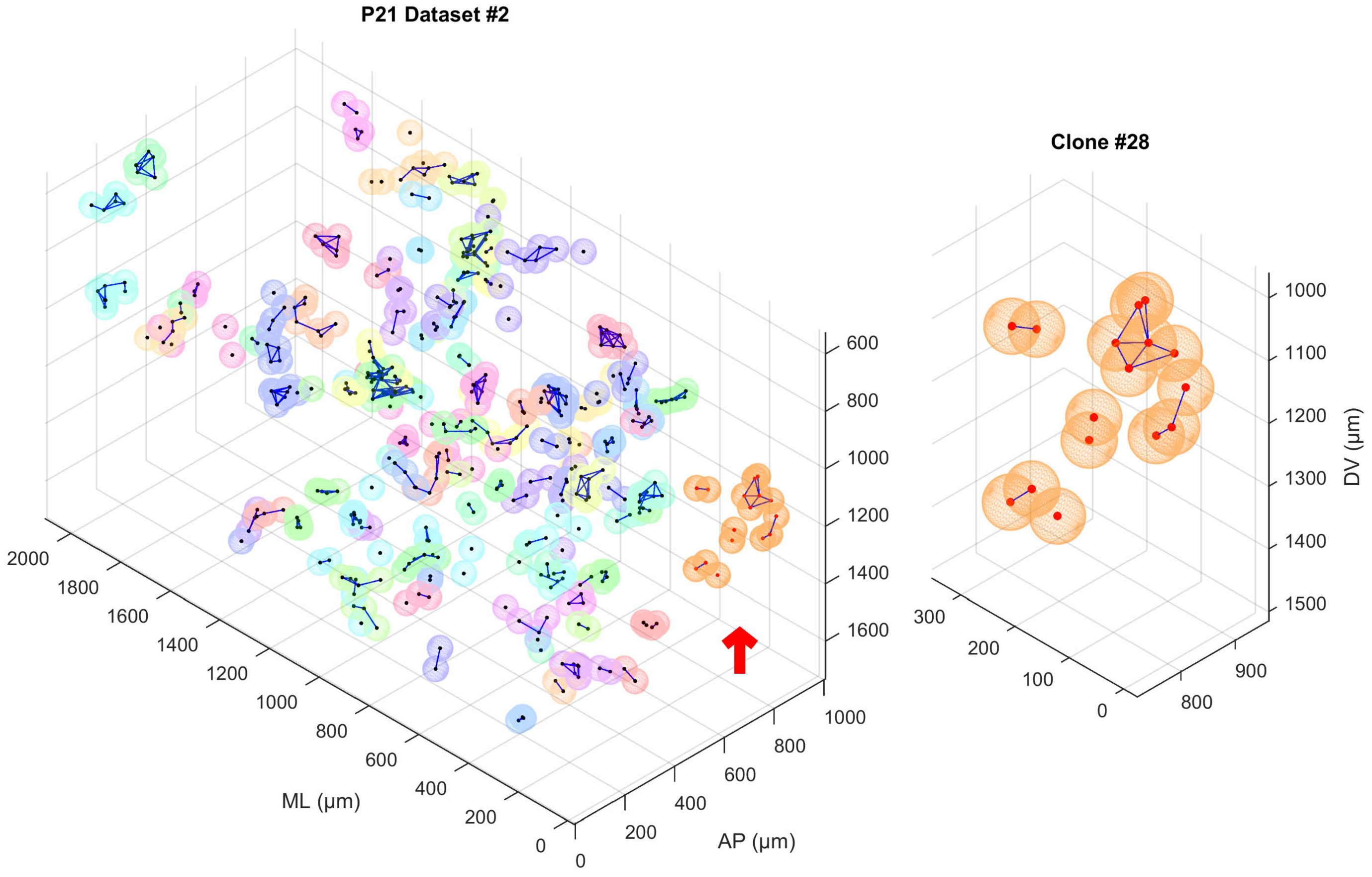

P21 Dataset #2
600
800
1000
1200
1400
1600
2000
1800
1600
1400
1200
1000
800
600
400
200
0
ML (μm)
0
200
400
600
800
1000
AP (μm)
Clone #28
1000
1100
1200
1300
1400
1500
DV (μm)
300
200
100
0
800
900

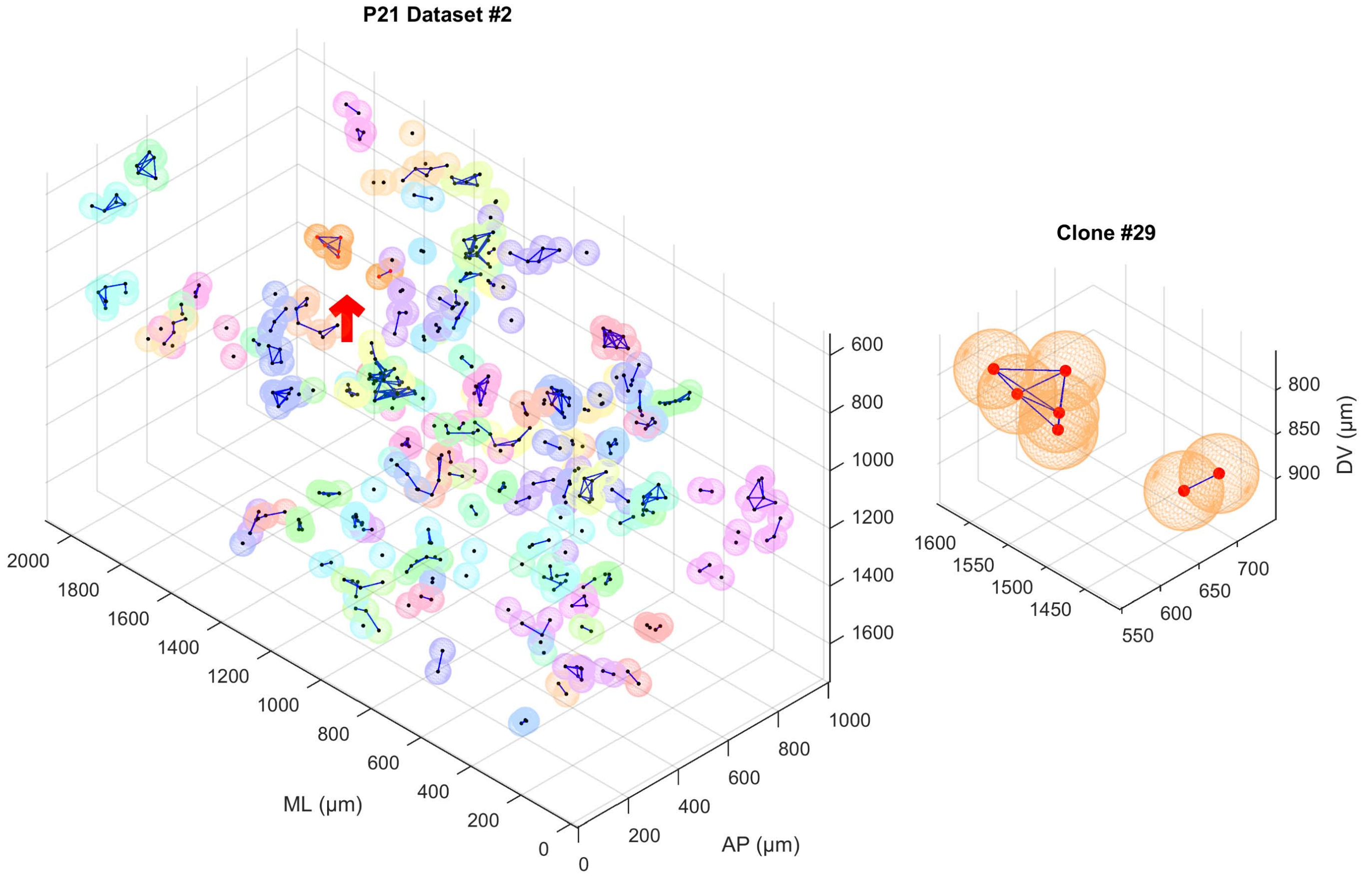

P21 Dataset #2
600
800
1000
1200
1400
1600
2000
1800
1600
1400
1200
1000
800
600
400
200
0
ML (µm)
0
200
400
600
800
1000
AP (µm)
Clone #29
800
850
900
DV (µm)
1600
1550
1500
1450
550
600
650
700

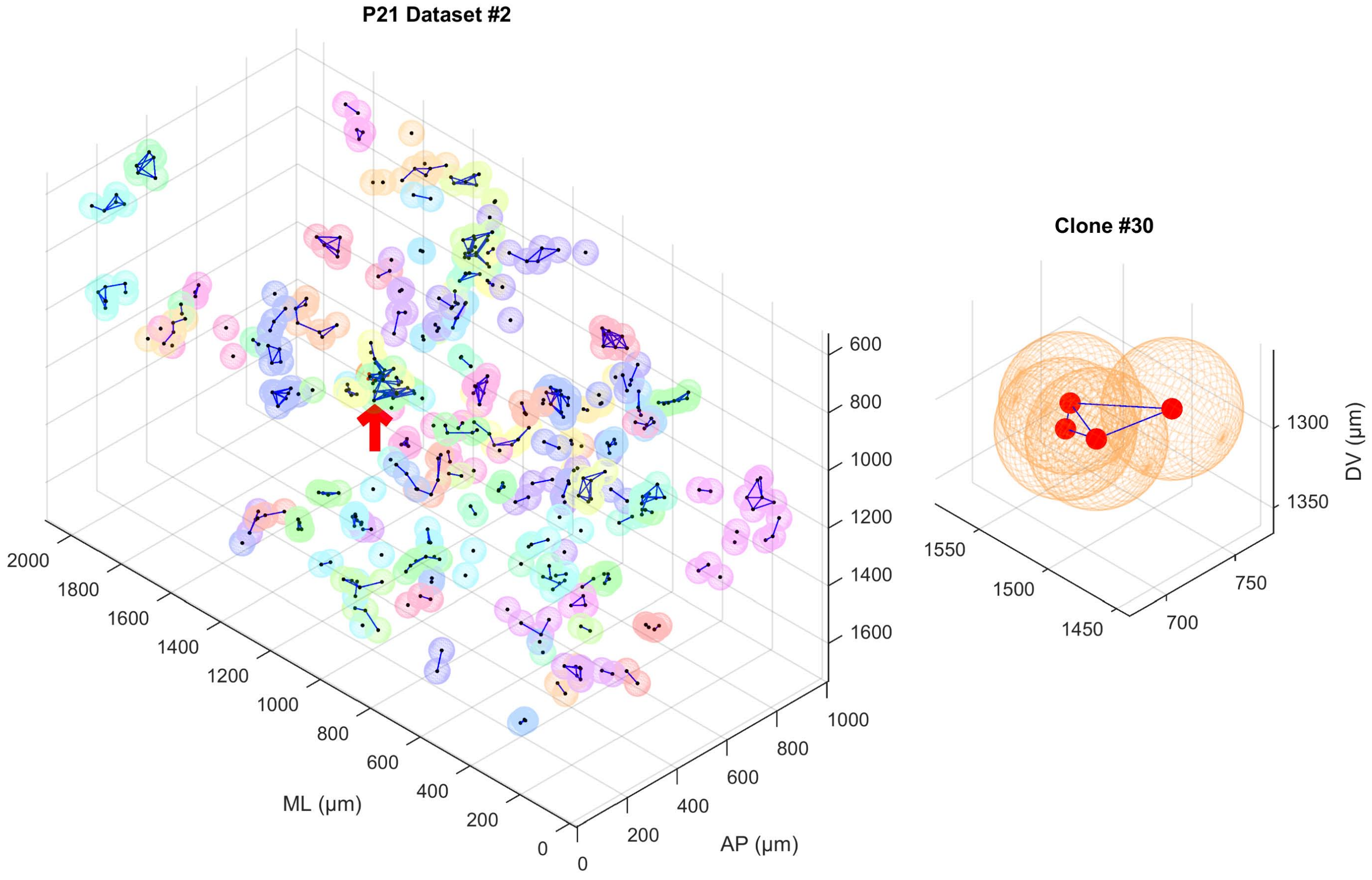

P21 Dataset #2
ML (µm)
AP (µm)
2000
1800
1600
1400
1200
1000
800
600
400
200
0
0
200
400
600
800
1000
600
800
1000
1200
1400
1600
Clone #30
DV (µm)
1300
1350
1550
1500
1450
700
750

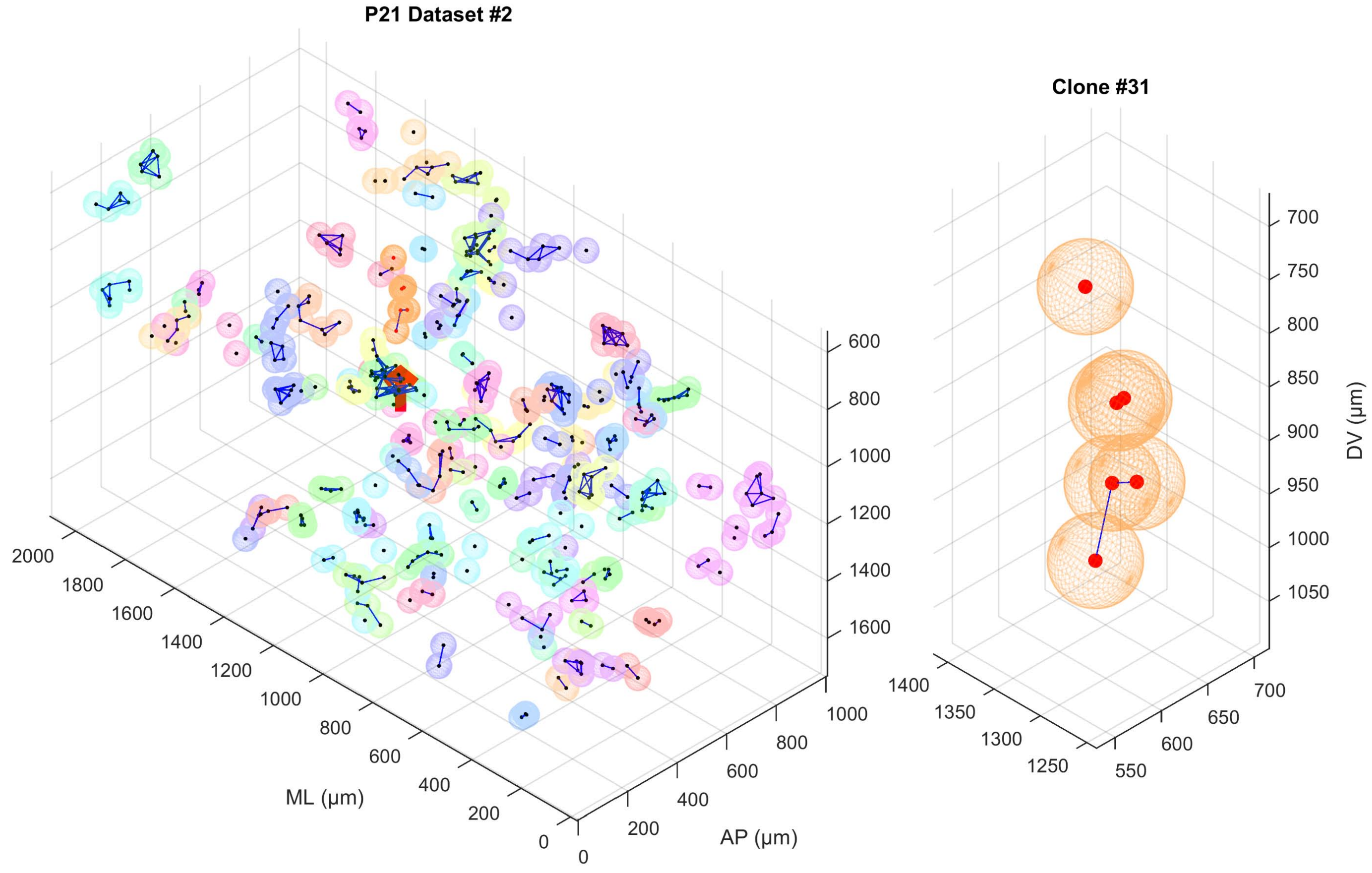

P21 Dataset #2
600
800
1000
1200
1400
1600
2000
1800
1600
1400
1200
1000
800
600
400
200
0
ML (μm)
0
200
400
600
800
1000
AP (μm)
Clone #31
700
750
800
850
900
950
1000
1050
DV (μm)
1400
1350
1300
1250
550
600
650
700

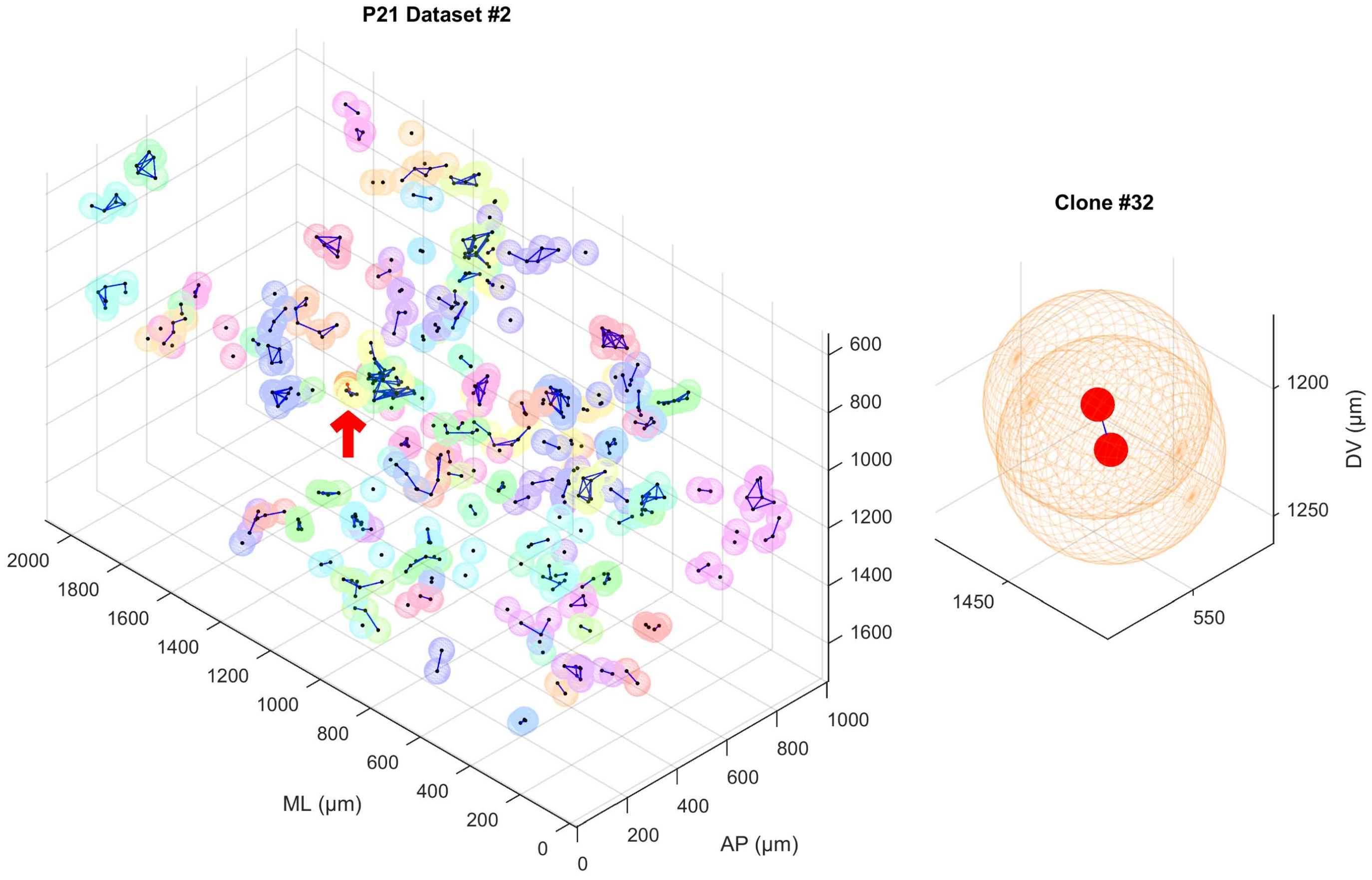

P21 Dataset #2
ML (μm)
AP (μm)
2000
1800
1600
1400
1200
1000
800
600
400
200
0
0
200
400
600
800
1000
600
800
1000
1200
1400
1600
Clone #32
DV (μm)
1200
1250
1450
550

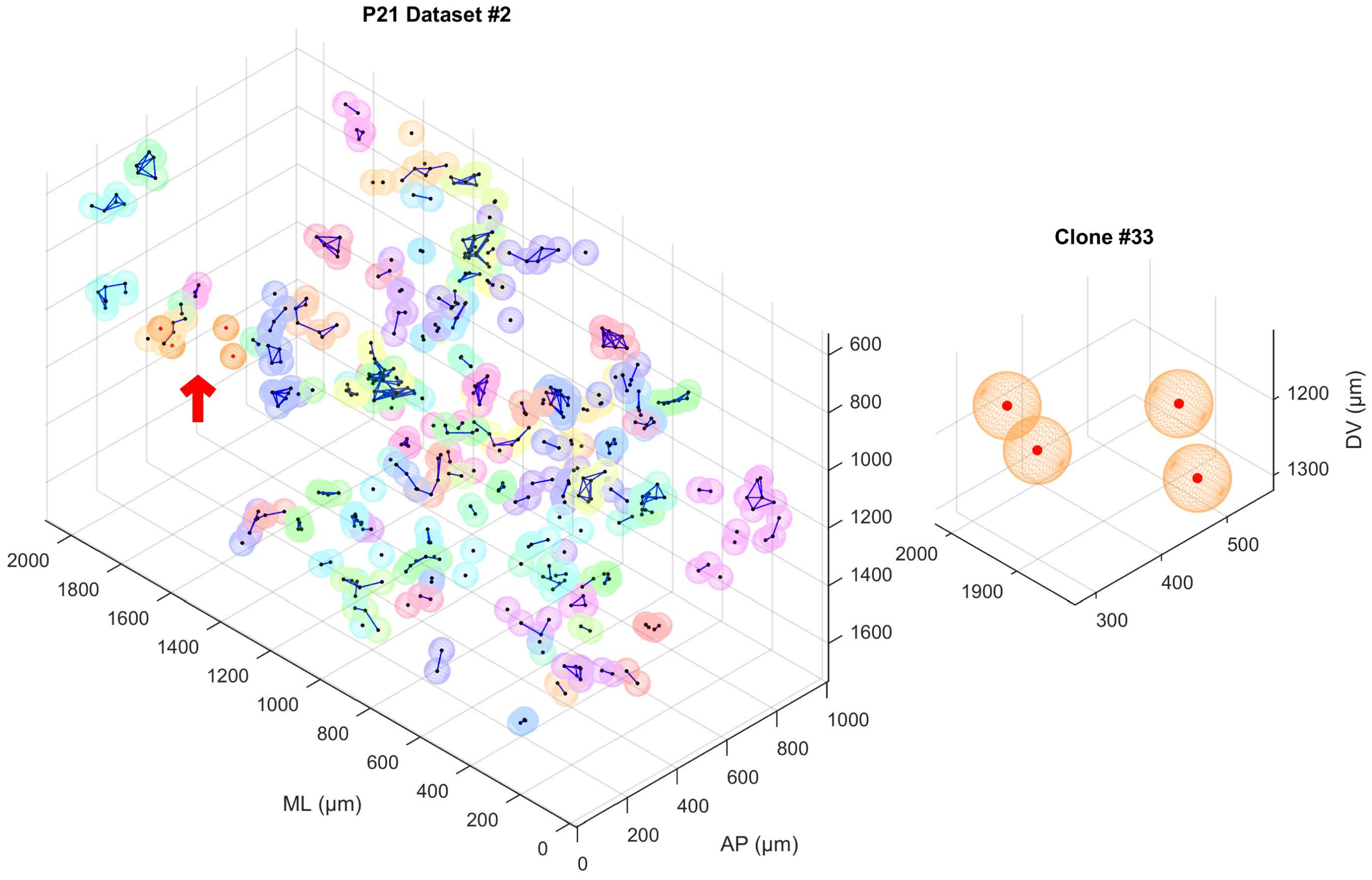
P21 Dataset #2
Clone #33
ML (µm)
AP (µm)
DV (µm)
2000
1800
1600
1400
1200
1000
800
600
400
200
0
200
400
600
800
1000
0
600
800
1000
1200
1400
1600
1900
2000
300
400
500
1200
1300

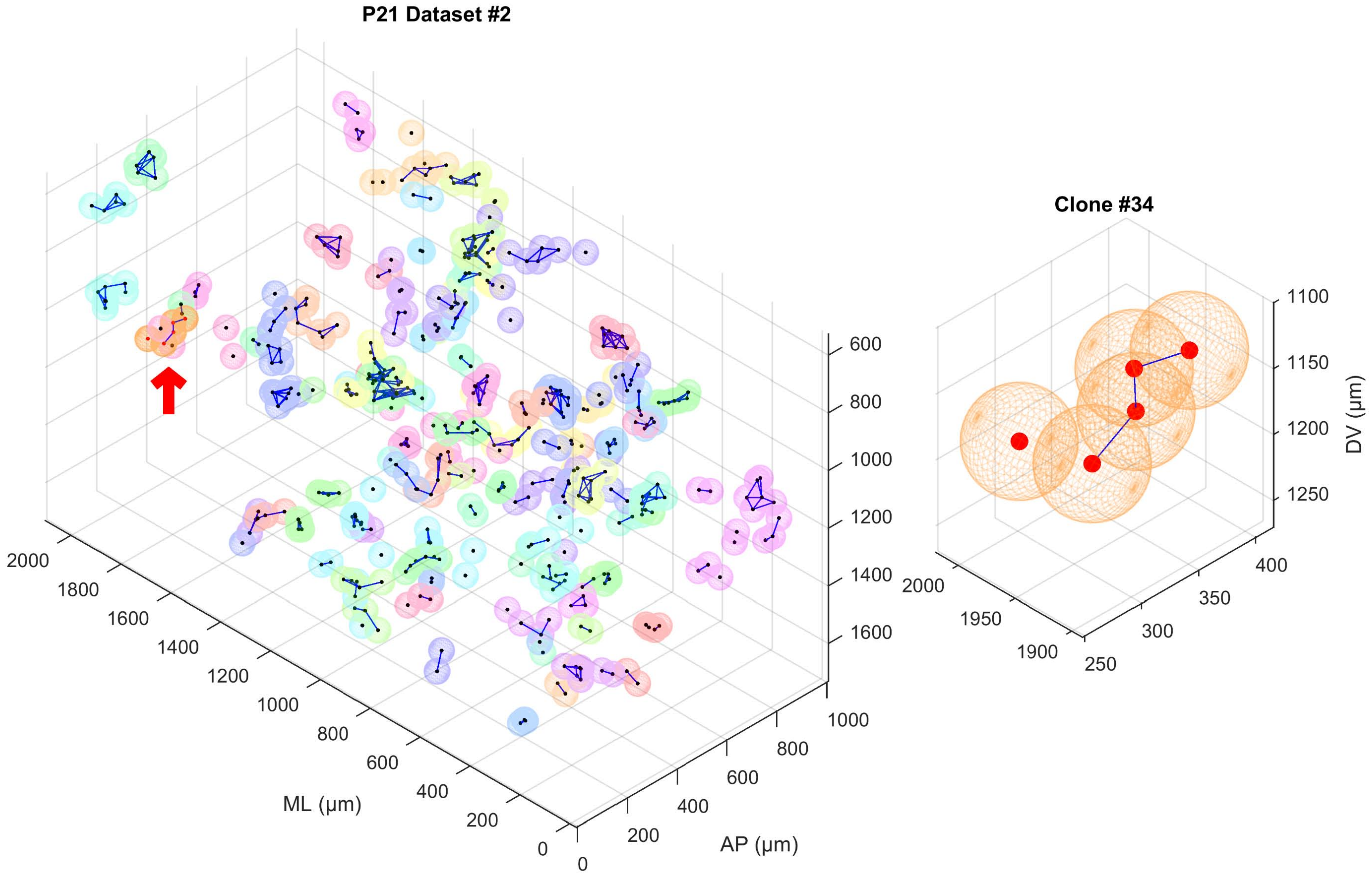

P21 Dataset #2
ML (µm)
2000
1800
1600
1400
1200
1000
800
600
400
200
0
AP (µm)
200
400
600
800
1000
600
800
1000
1200
1400
1600
Clone #34
DV (µm)
1100
1150
1200
1250
2000
1950
1900
250
300
350
400

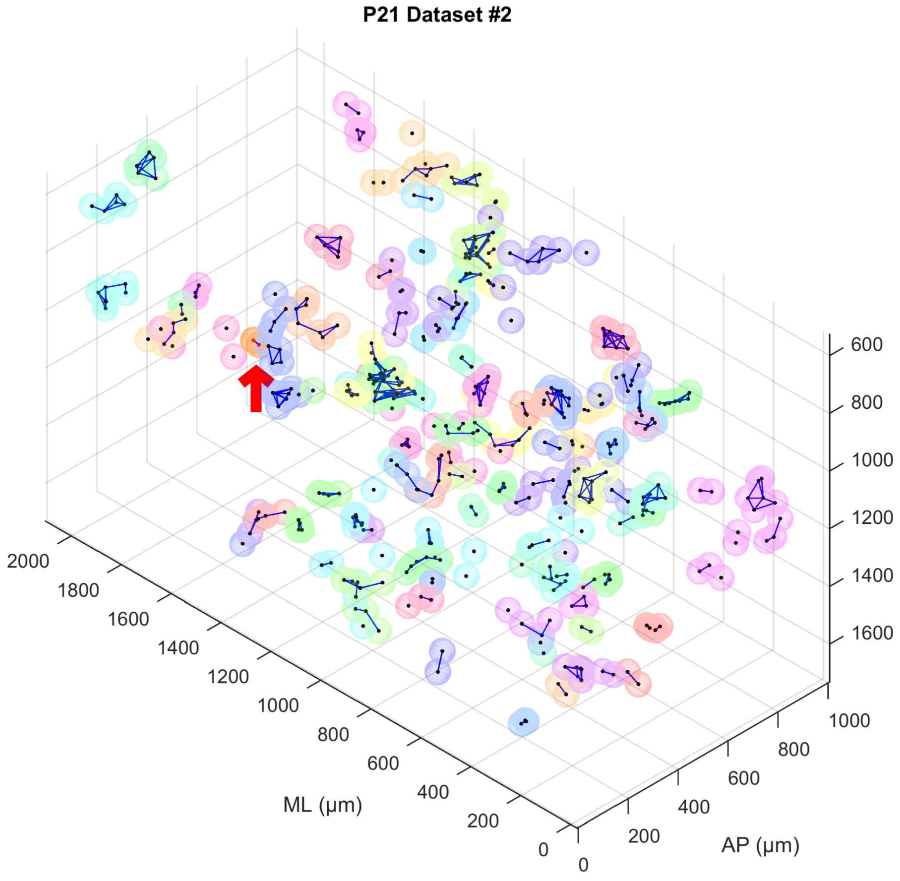

P21 Dataset #2
600
800
1000
1200
1400
1600
2000
1800
1600
1400
1200
1000
800
600
400
200
0
ML (µm)
0
200
400
600
800
1000
AP (µm)

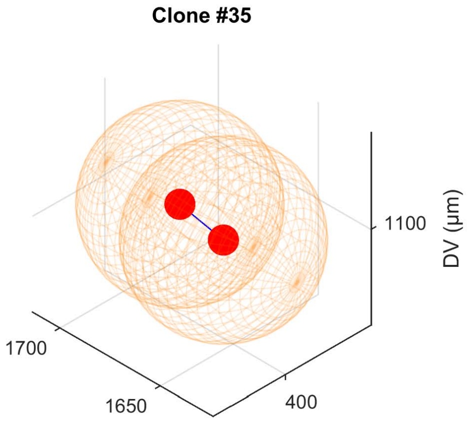

Clone #35
1100
DV (µm)
1700
1650
400

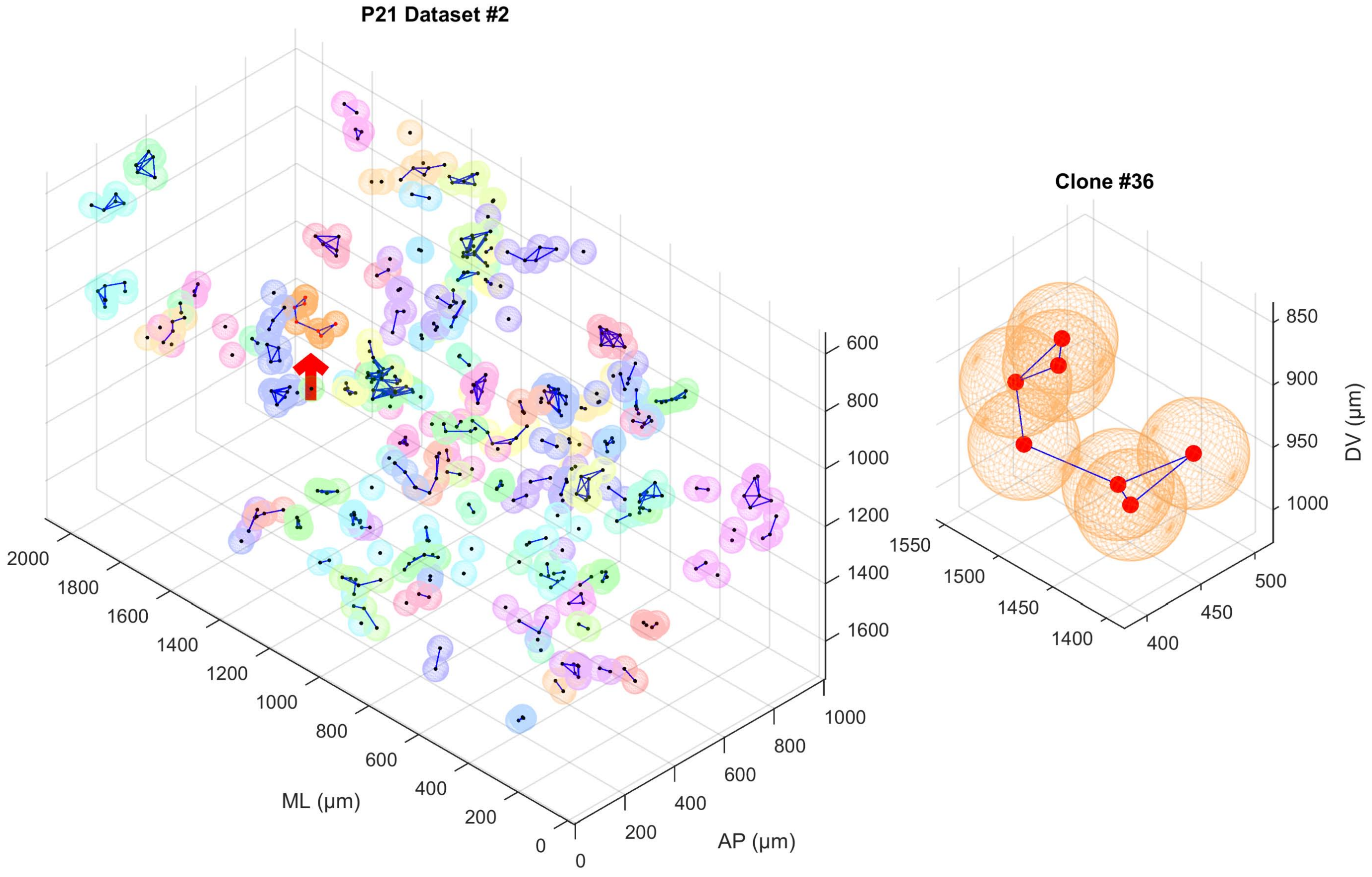
P21 Dataset #2
ML (µm)
AP (µm)
2000
1800
1600
1400
1200
1000
800
600
400
200
0
0
200
400
600
800
1000
600
800
1000
1200
1400
1600
Clone #36
DV (µm)
850
900
950
1000
1550
1500
1450
1400
400
450
500

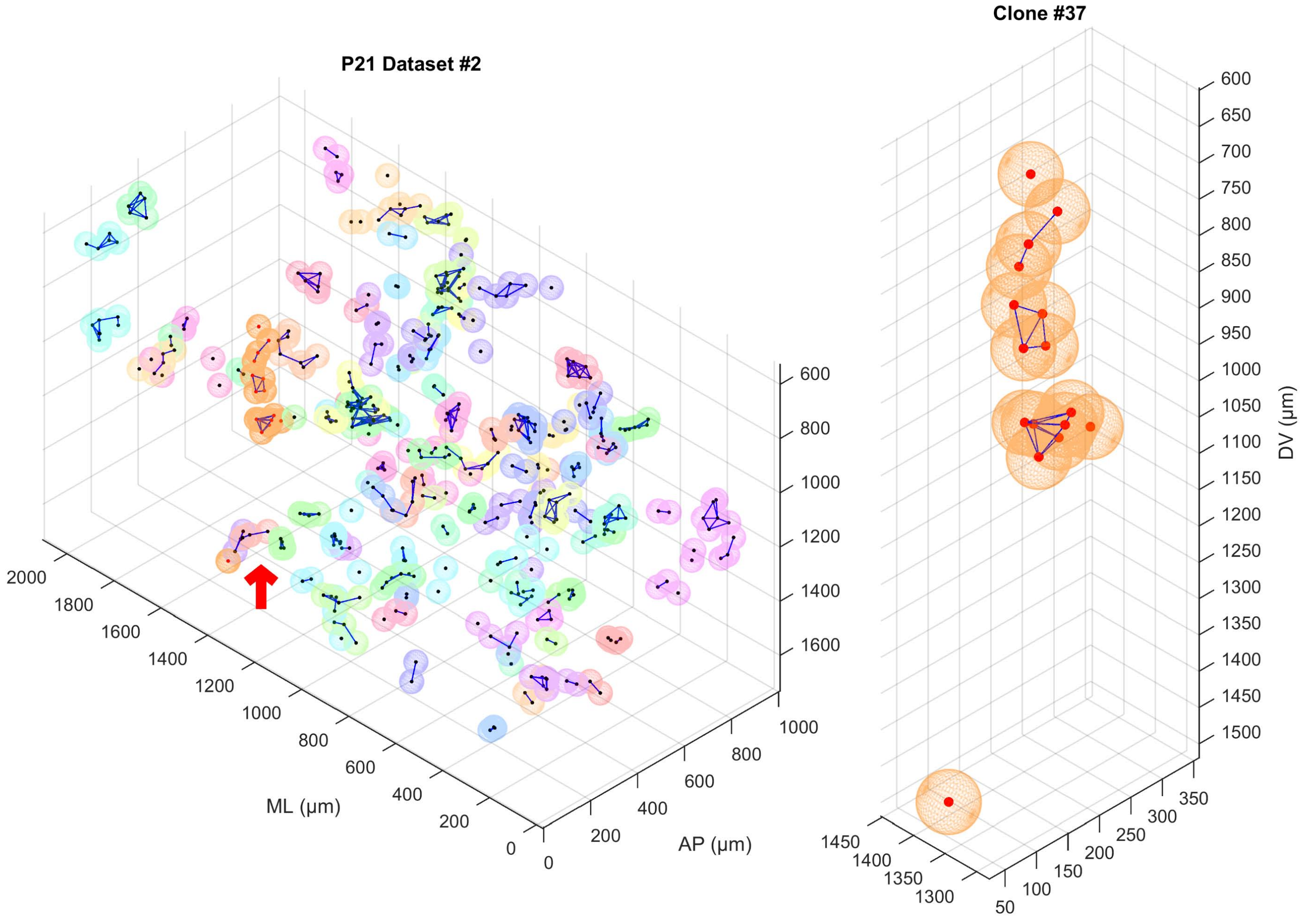
P21 Dataset #2
ML (µm)
AP (µm)
2000
1800
1600
1400
1200
1000
800
600
400
200
0
0
200
400
600
800
1000
600
800
1000
1200
1400
1600
Clone #37
DV (µm)
600
650
700
750
800
850
900
950
1000
1050
1100
1150
1200
1250
1300
1350
1400
1450
1500
1450
1400
1350
1300
50
100
150
200
250
300
350

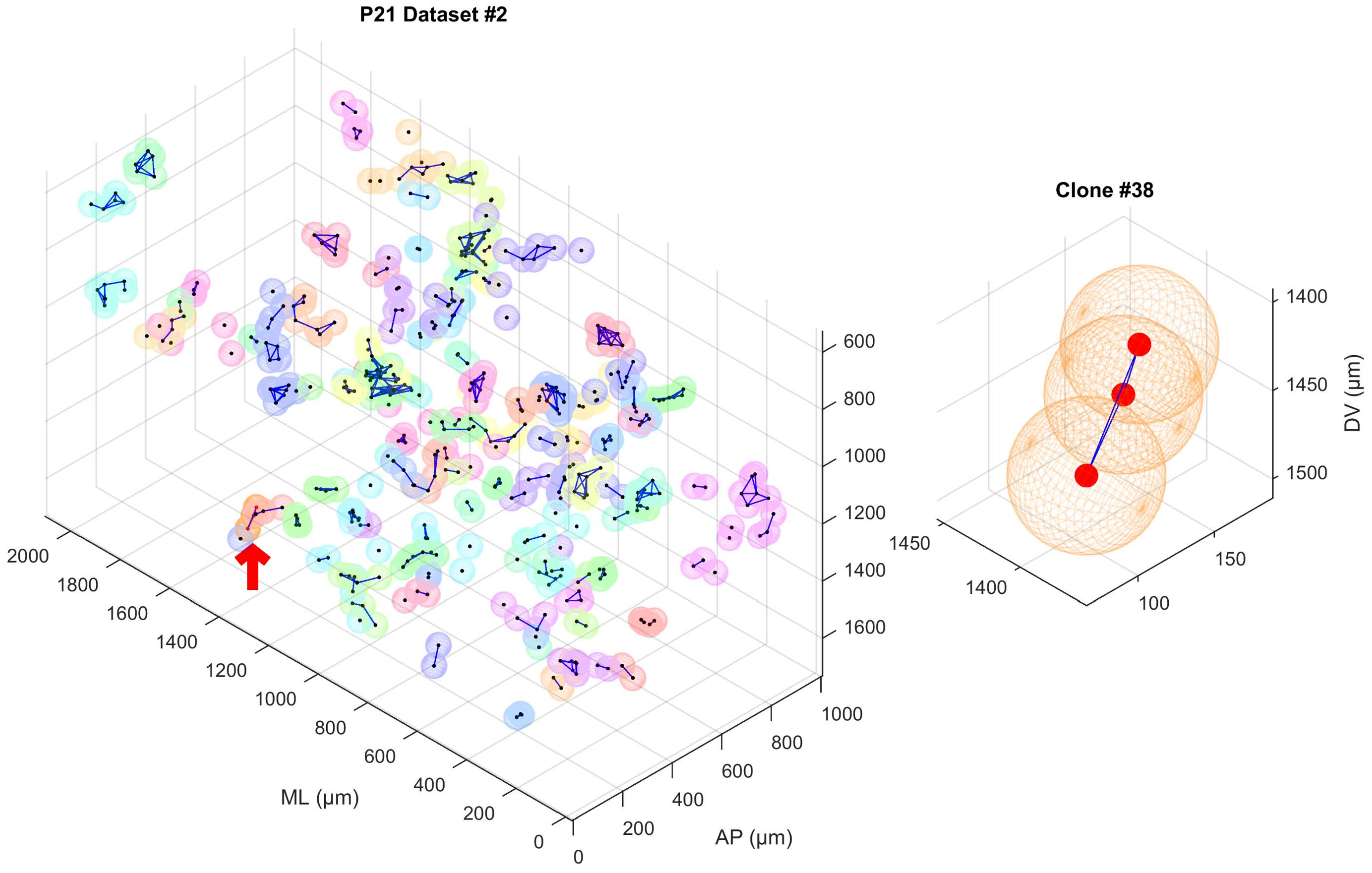

P21 Dataset #2
ML (µm)
AP (µm)
2000
1800
1600
1400
1200
1000
800
600
400
200
0
0
200
400
600
800
1000
600
800
1000
1200
1400
1600
Clone #38
DV (µm)
1400
1450
1500
1450
1400
100
150

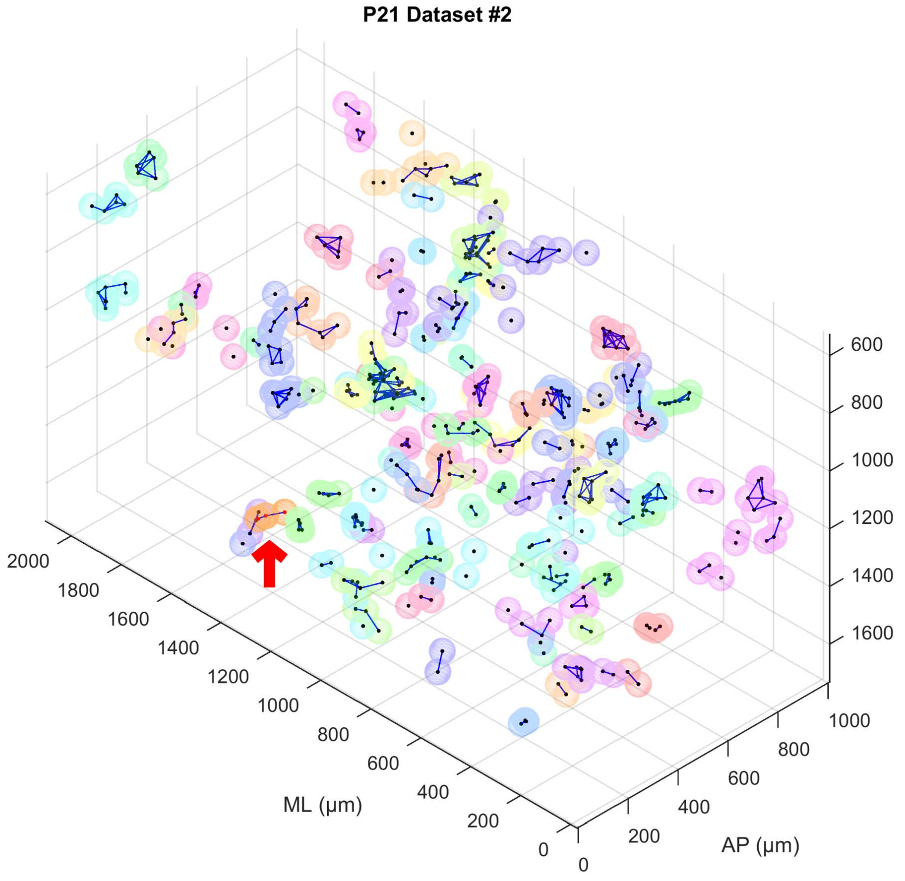

P21 Dataset #2
600
800
1000
1200
1400
1600
2000
1800
1600
1400
1200
1000
800
600
400
200
0
ML (µm)
0
200
400
600
800
1000
AP (µm)

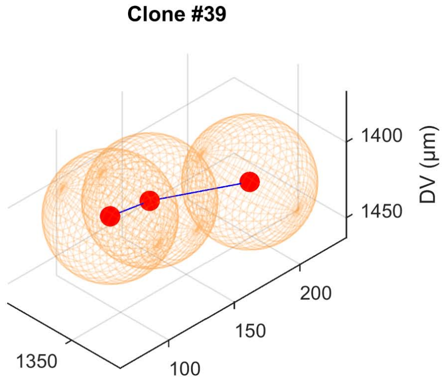

Clone #39
1400
1450
DV (µm)
1350
100
150
200

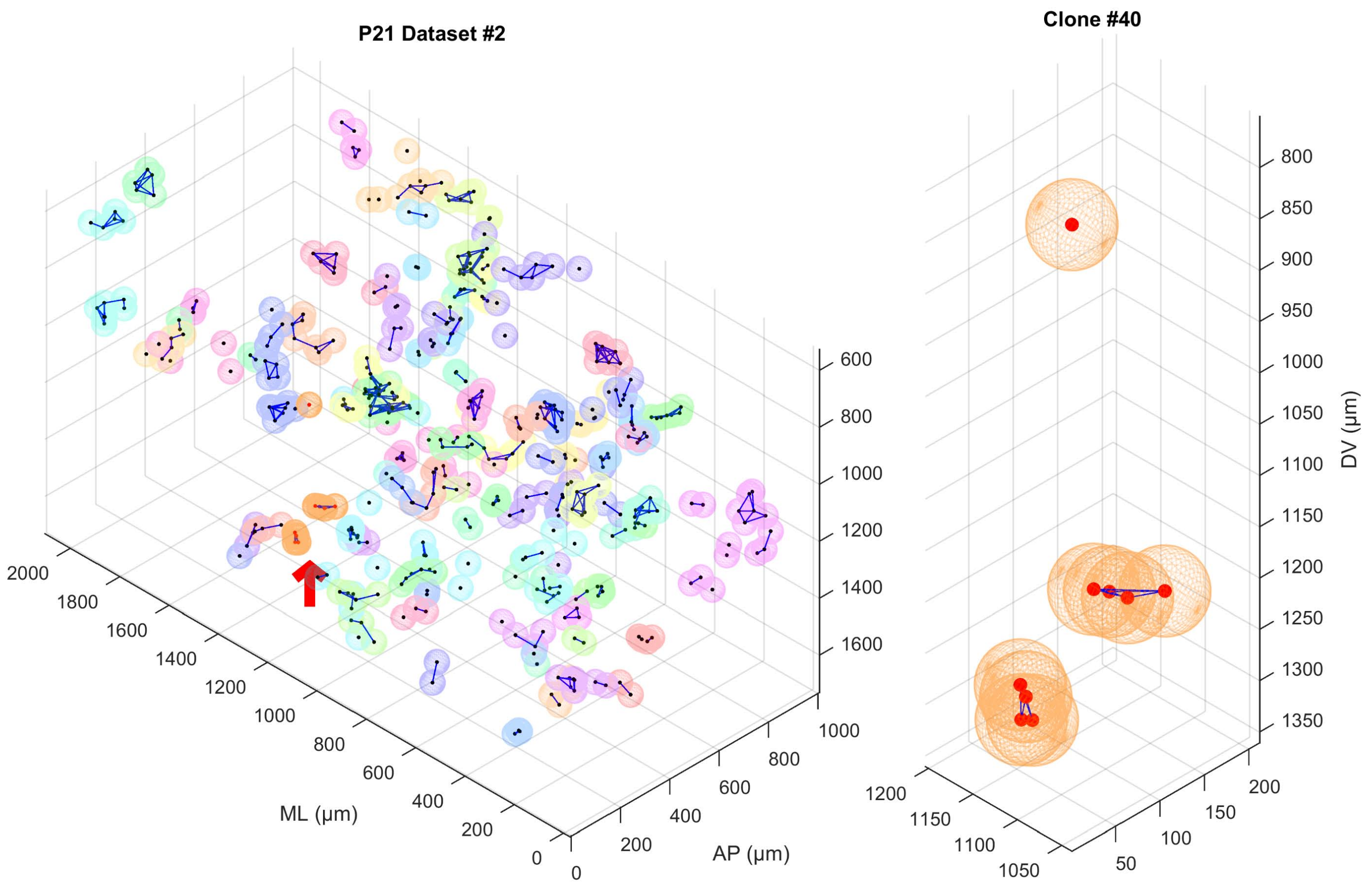

P21 Dataset #2
Clone #40
ML (µm)
AP (µm)
DV (µm)
2000
1800
1600
1400
1200
1000
800
600
400
200
0
0
200
400
600
800
1000
600
800
1000
1200
1400
1600
800
850
900
950
1000
1050
1100
1150
1200
1250
1300
1350
1200
1150
1100
1050
50
100
150
200

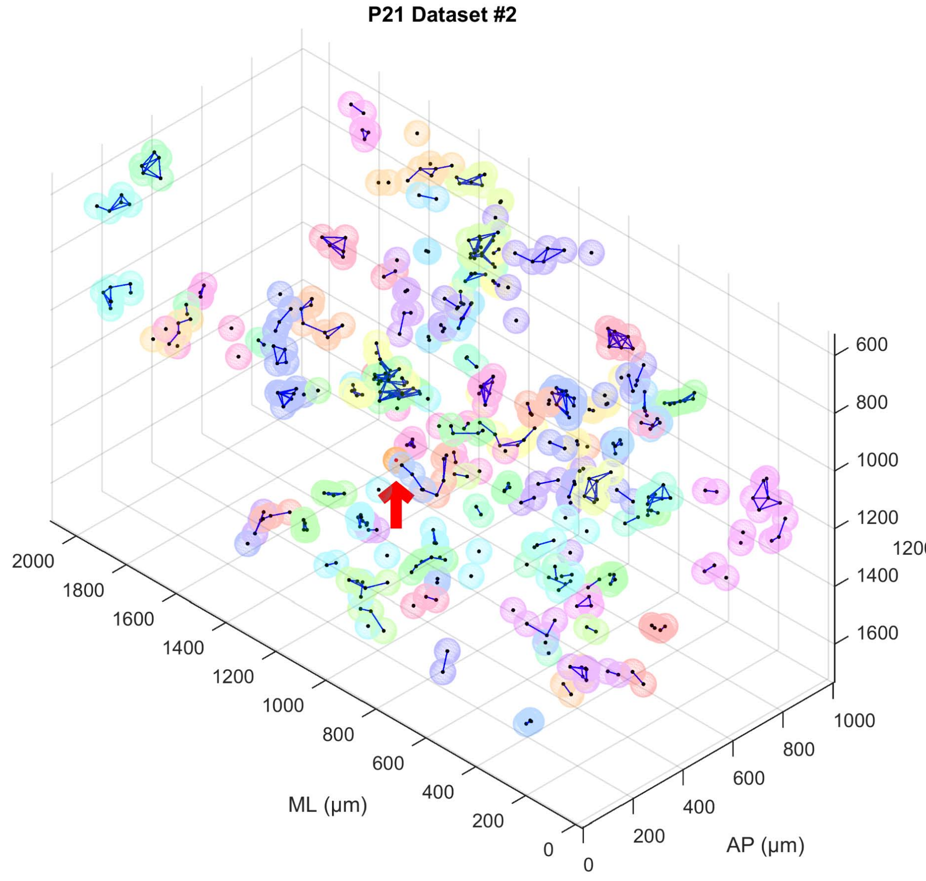

P21 Dataset #2
600
800
1000
1200
1400
1600
2000
1800
1600
1400
1200
1000
800
600
400
200
0
ML (µm)
0
200
400
600
800
1000
AP (µm)

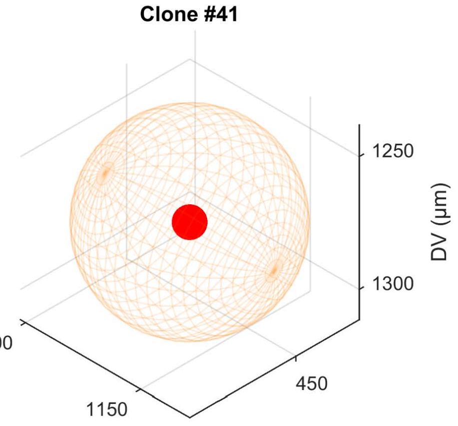

Clone #41
1250
1300
DV (µm)
1200
1150
450

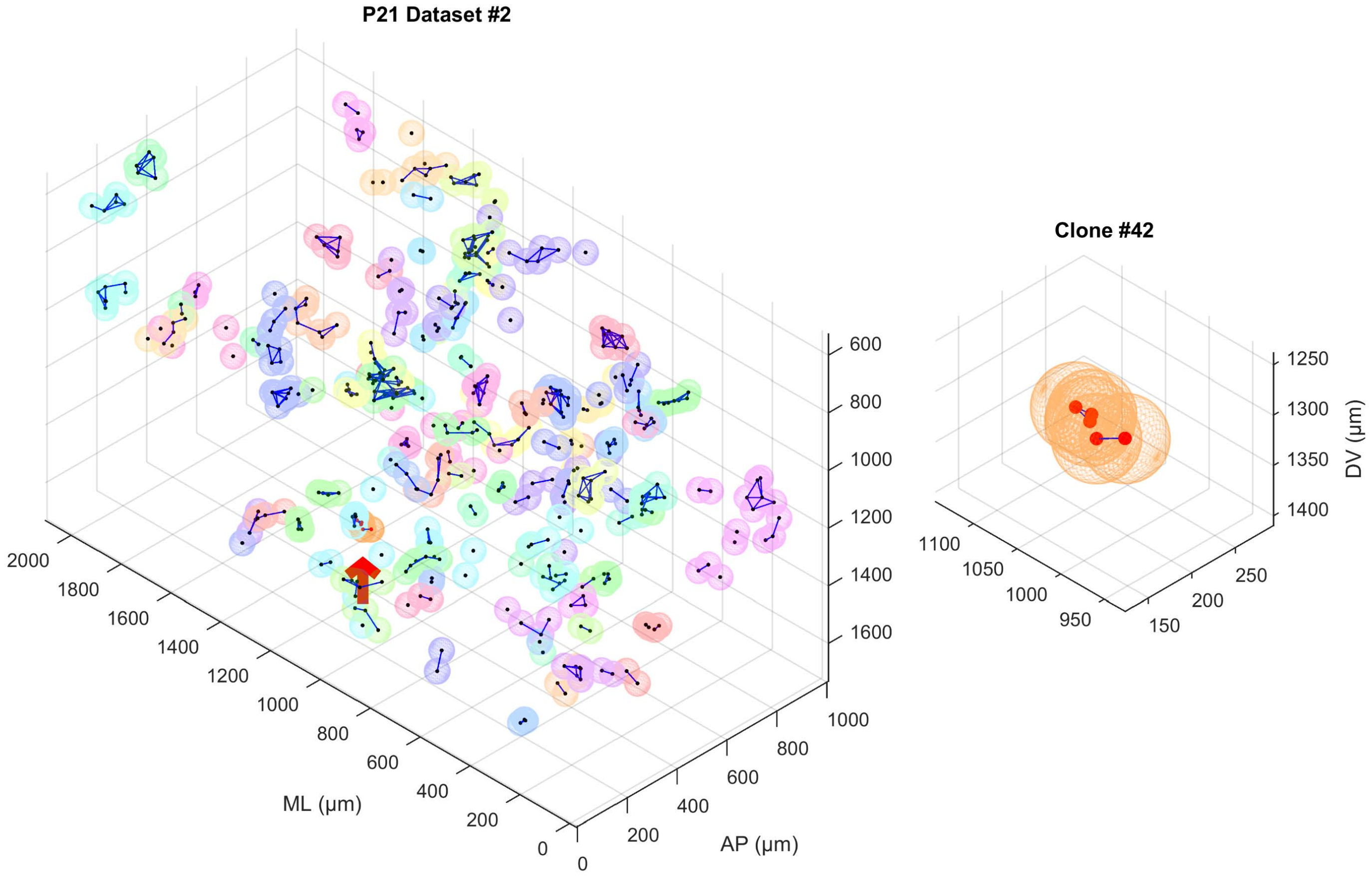

P21 Dataset #2
ML (µm)
AP (µm)
2000
1800
1600
1400
1200
1000
800
600
400
200
0
0
200
400
600
800
1000
600
800
1000
1200
1400
1600
Clone #42
DV (µm)
1250
1300
1350
1400
1100
1050
1000
950
150
200
250

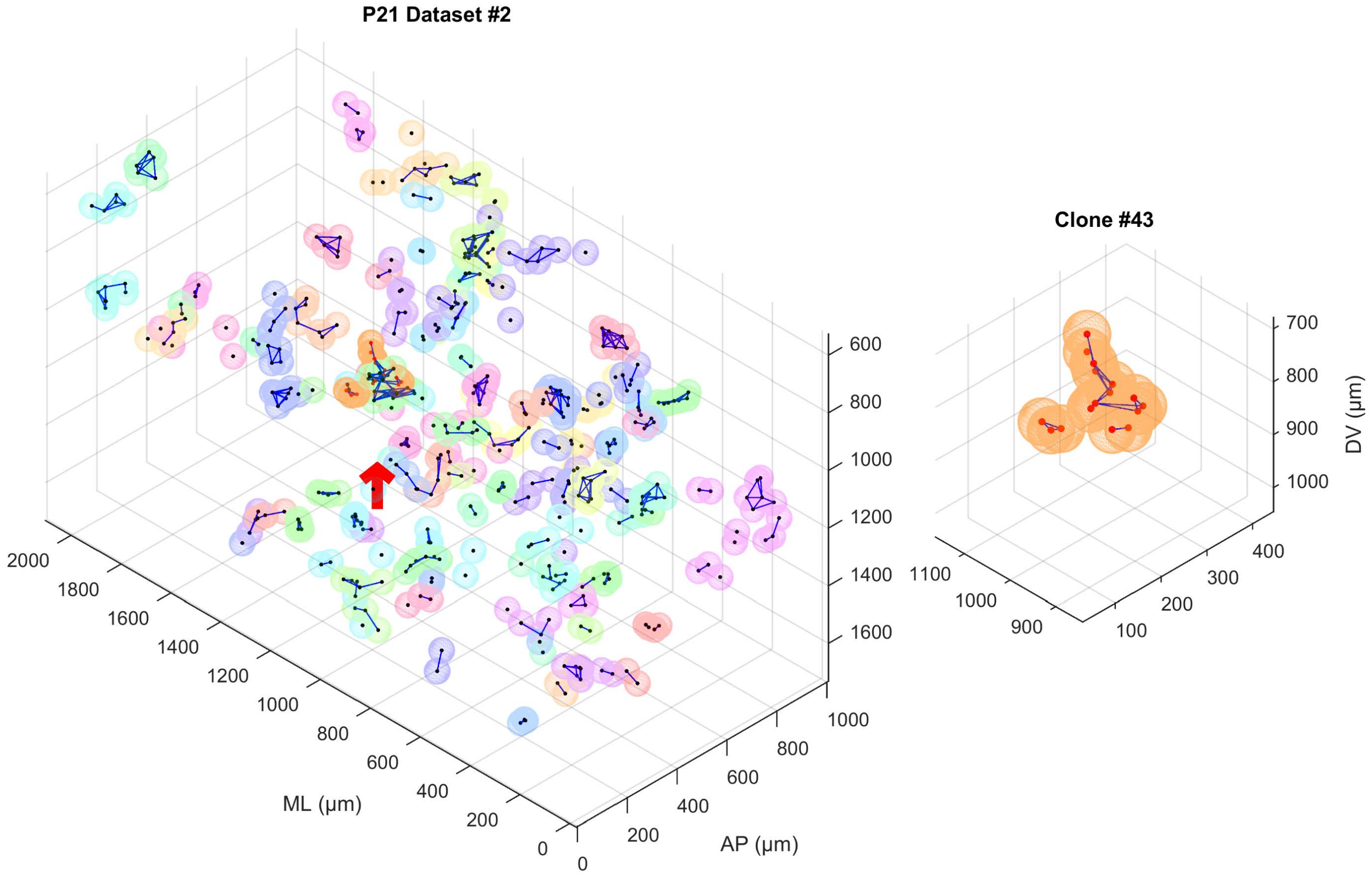

P21 Dataset #2
ML (μm)
2000
1800
1600
1400
1200
1000
800
600
400
200
0
AP (μm)
0
200
400
600
800
1000
600
800
1000
1200
1400
1600
Clone #43
DV (μm)
700
800
900
1000
1100
1000
900
100
200
300
400

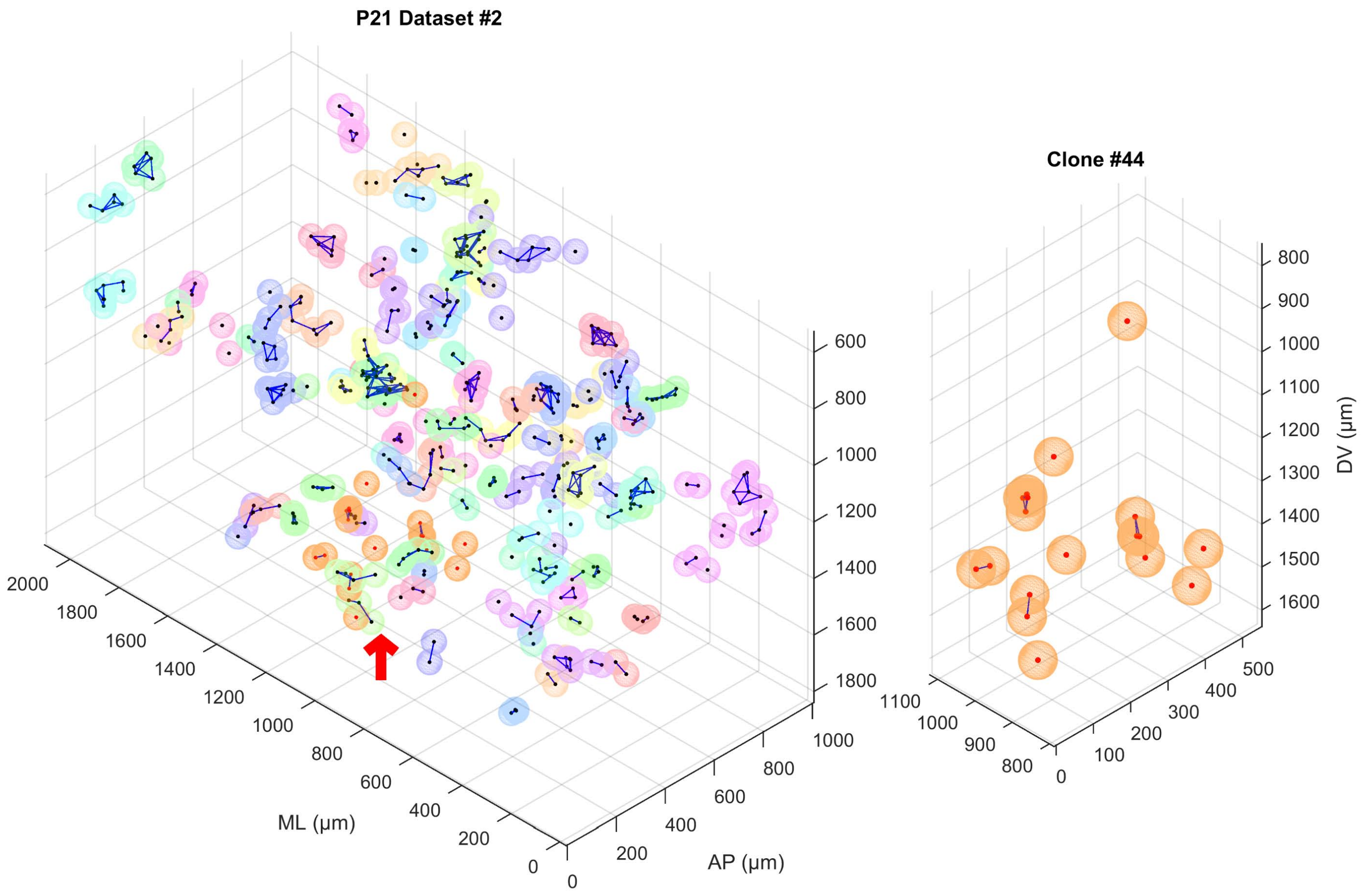
P21 Dataset #2
ML (µm)
AP (µm)
Clone #44
DV (µm)

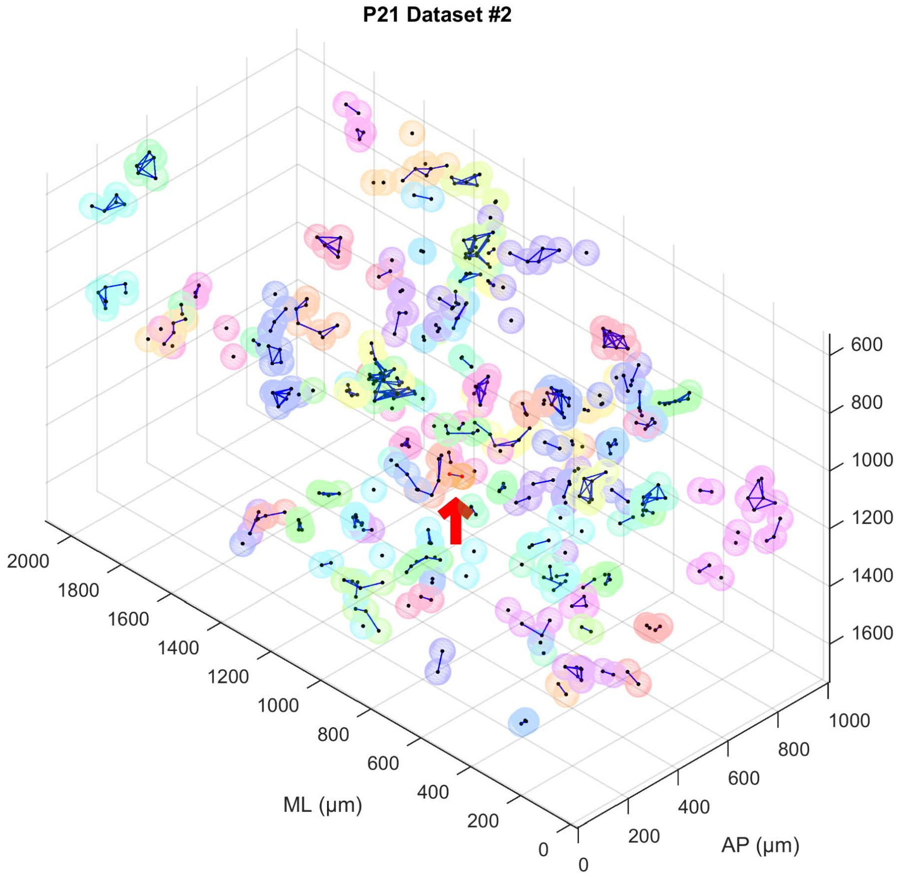

P21 Dataset #2
ML (µm)
2000
1800
1600
1400
1200
1000
800
600
400
200
0
AP (µm)
0
200
400
600
800
1000
600
800
1000
1200
1400
1600

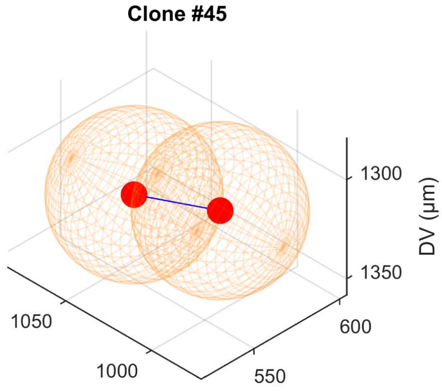

Clone #45
DV (µm)
1300
1350
1050
1000
600
550

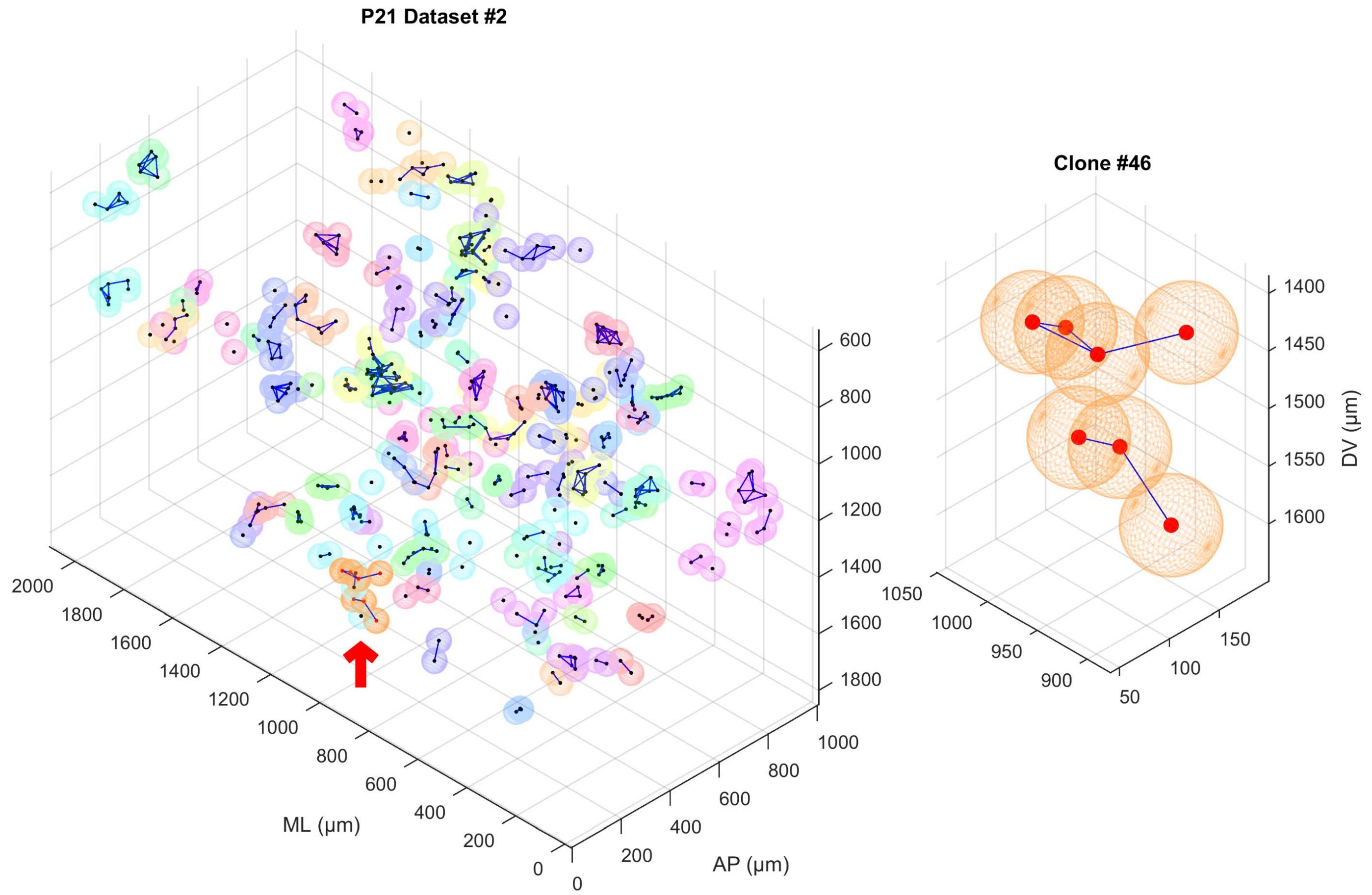

P21 Dataset #2
Clone #46
ML (µm)
AP (µm)
DV (µm)
2000
1800
1600
1400
1200
1000
800
600
400
200
0
0
200
400
600
800
1000
600
800
1000
1200
1400
1600
1800
1050
1000
950
900
50
100
150
1400
1450
1500
1550
1600

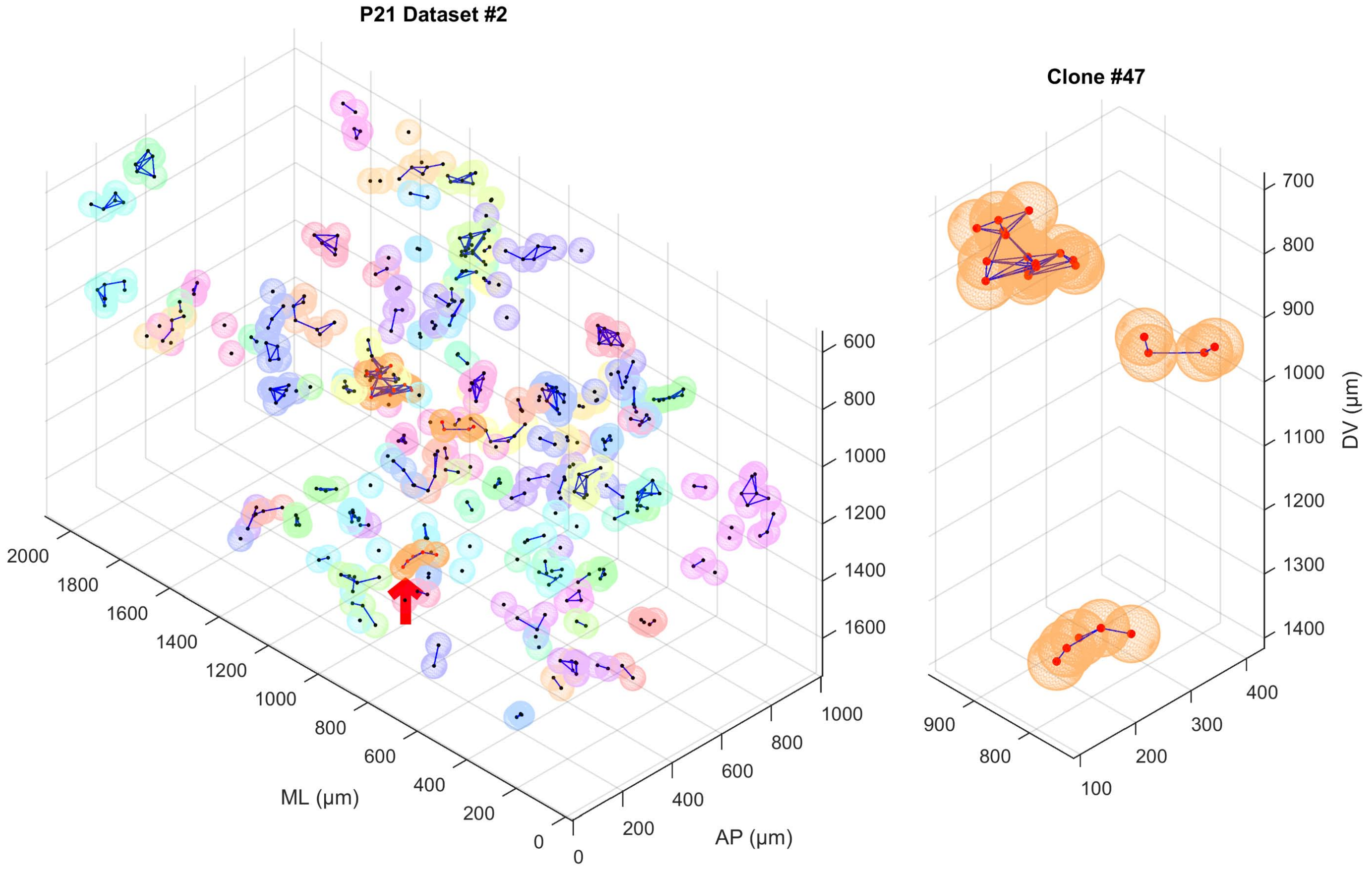
P21 Dataset #2
ML (µm)
2000
1800
1600
1400
1200
1000
800
600
400
200
0
AP (µm)
0
200
400
600
800
1000
600
800
1000
1200
1400
1600
Clone #47
DV (µm)
700
800
900
1000
1100
1200
1300
1400
900
800
100
200
300
400

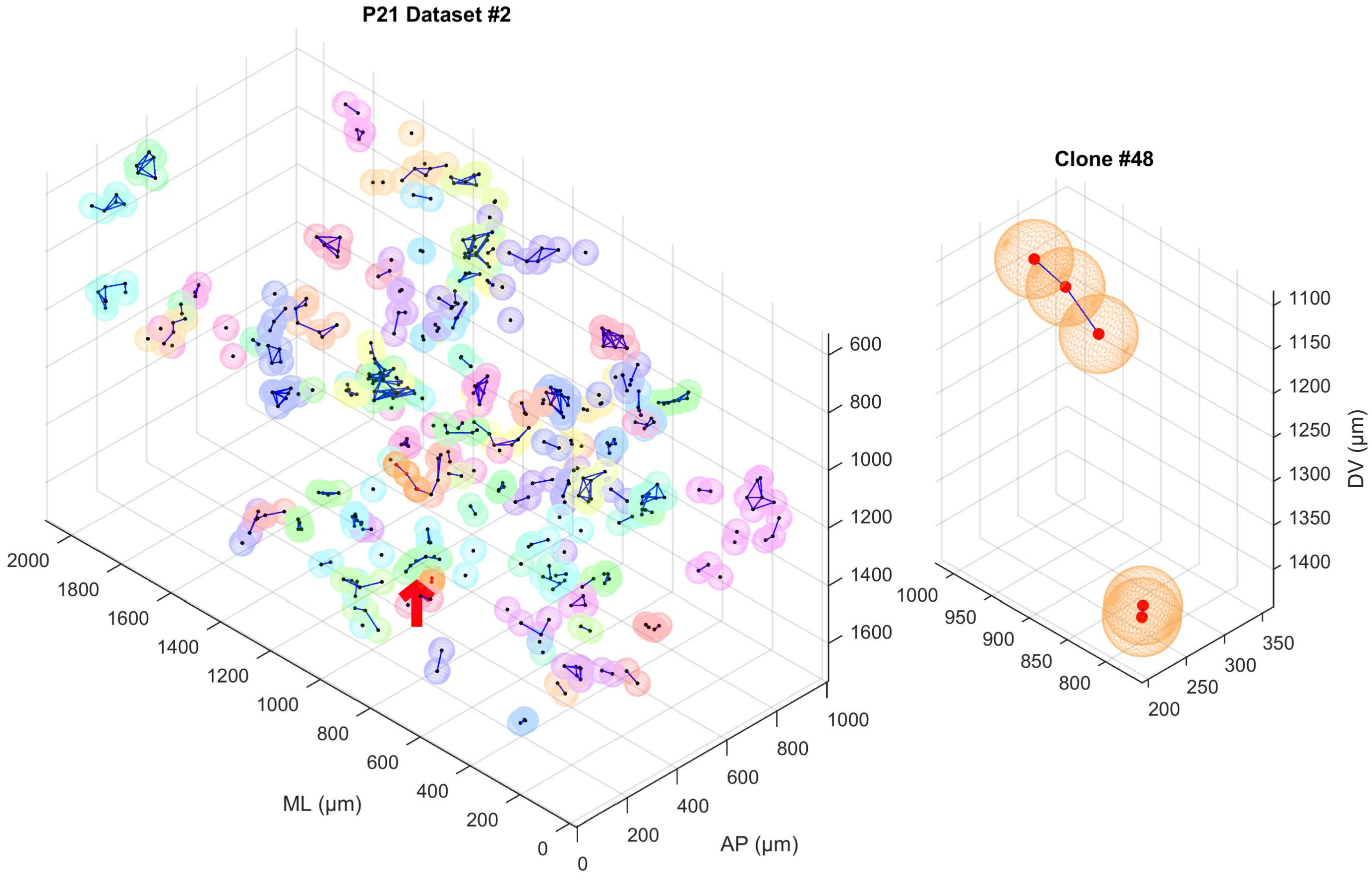
P21 Dataset #2
ML (µm)
2000
1800
1600
1400
1200
1000
800
600
400
200
0
AP (µm)
0
200
400
600
800
1000
600
800
1000
1200
1400
1600
Clone #48
DV (µm)
1100
1150
1200
1250
1300
1350
1400
1000
950
900
850
800
200
250
300
350

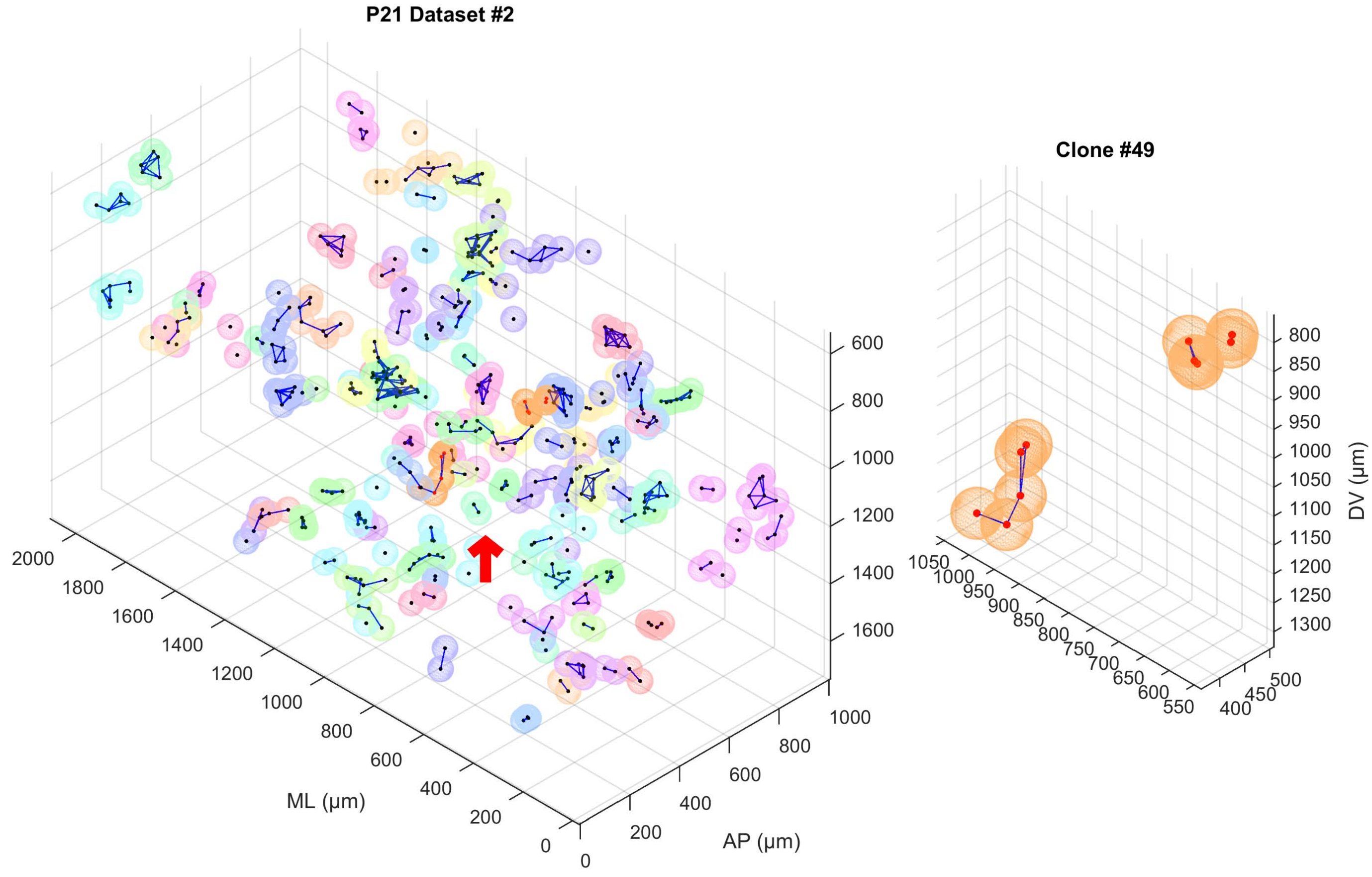
P21 Dataset #2
600
800
1000
1200
1400
1600
2000
1800
1600
1400
1200
1000
800
600
400
200
0
ML (μm)
0
200
400
600
800
1000
AP (μm)
Clone #49
800
850
900
950
1000
1050
1100
1150
1200
1250
1300
DV (μm)
1050
1000
950
900
850
800
750
700
650
600
550
400
450
500

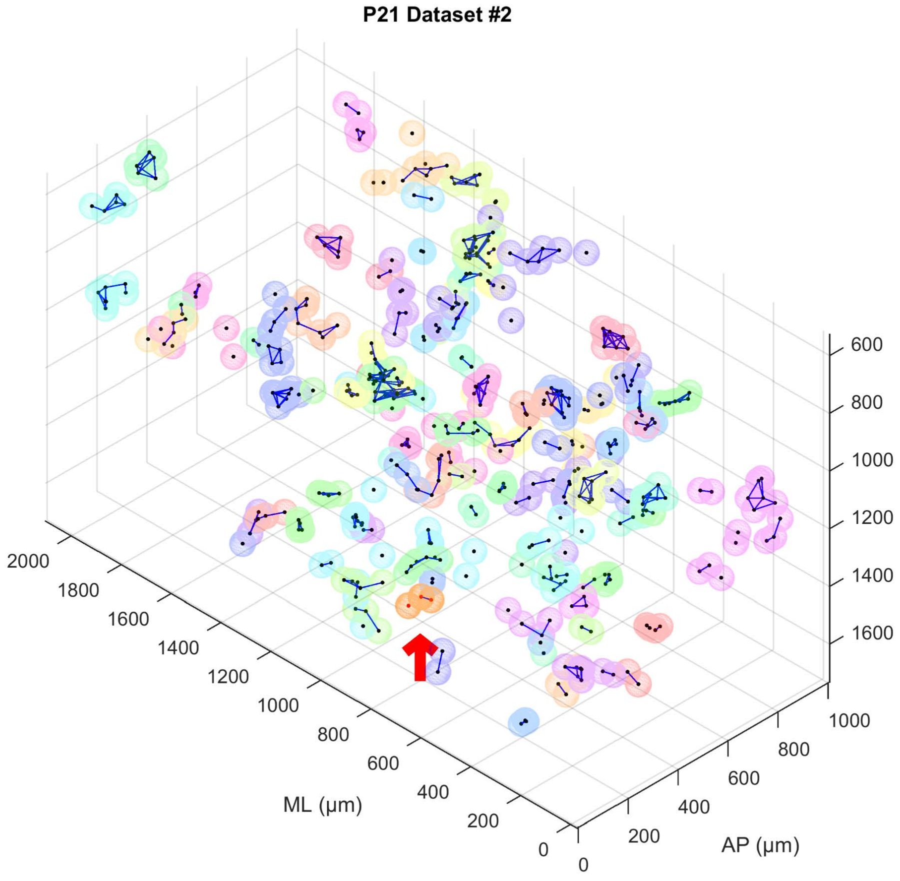
P21 Dataset #2
600
800
1000
1200
1400
1600
2000
1800
1600
1400
1200
1000
800
600
400
200
0
ML (µm)
0
200
400
600
800
1000
AP (µm)

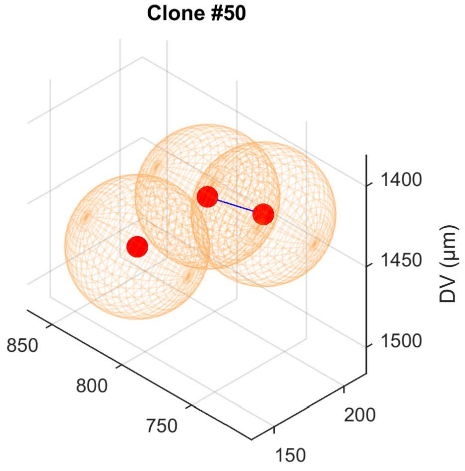
Clone #50
1400
1450
1500
DV (µm)
850
800
750
150
200

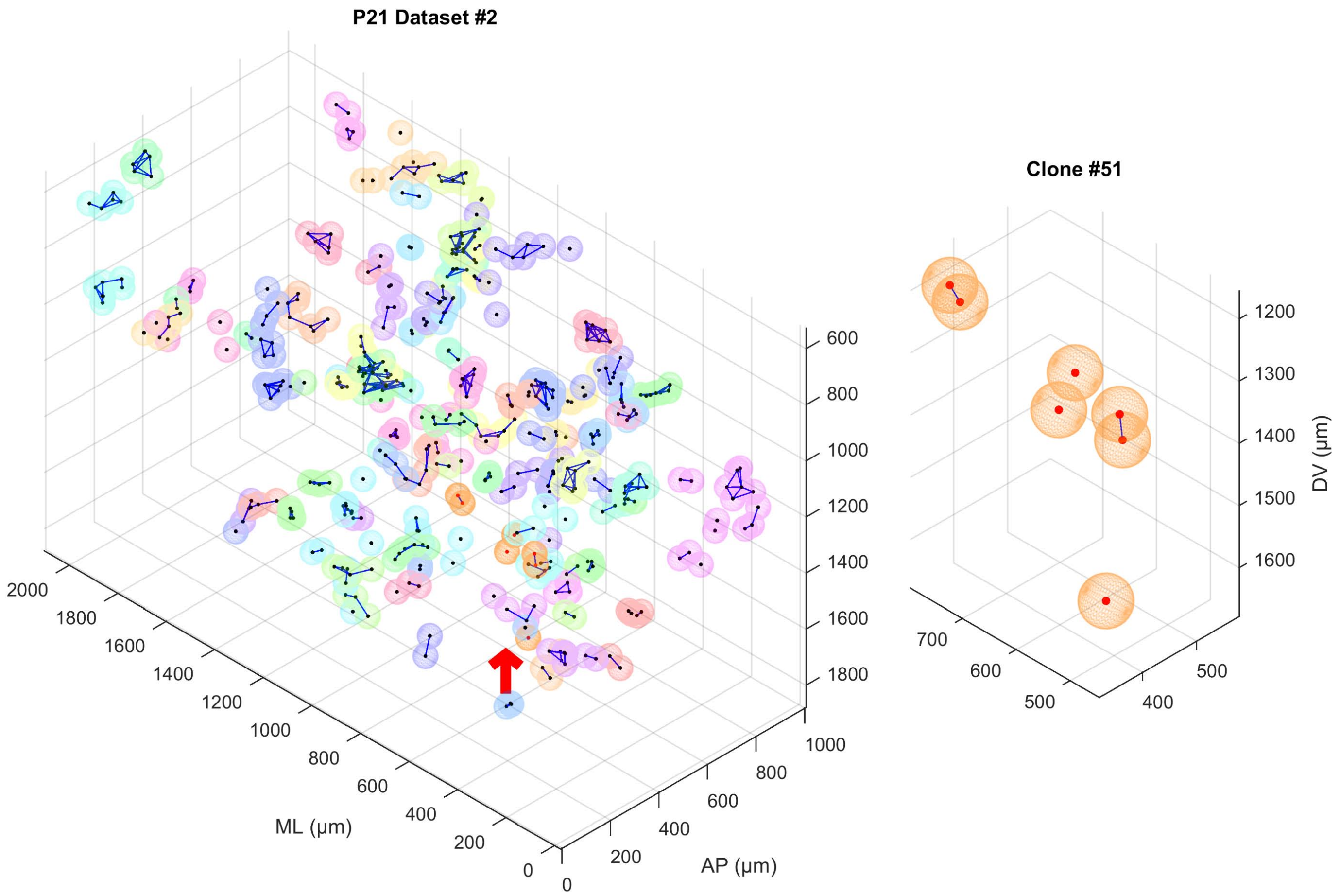

P21 Dataset #2
ML (µm)
AP (µm)
2000
1800
1600
1400
1200
1000
800
600
400
200
0
0
200
400
600
800
1000
600
800
1000
1200
1400
1600
1800
Clone #51
DV (µm)
1200
1300
1400
1500
1600
700
600
500
500
400

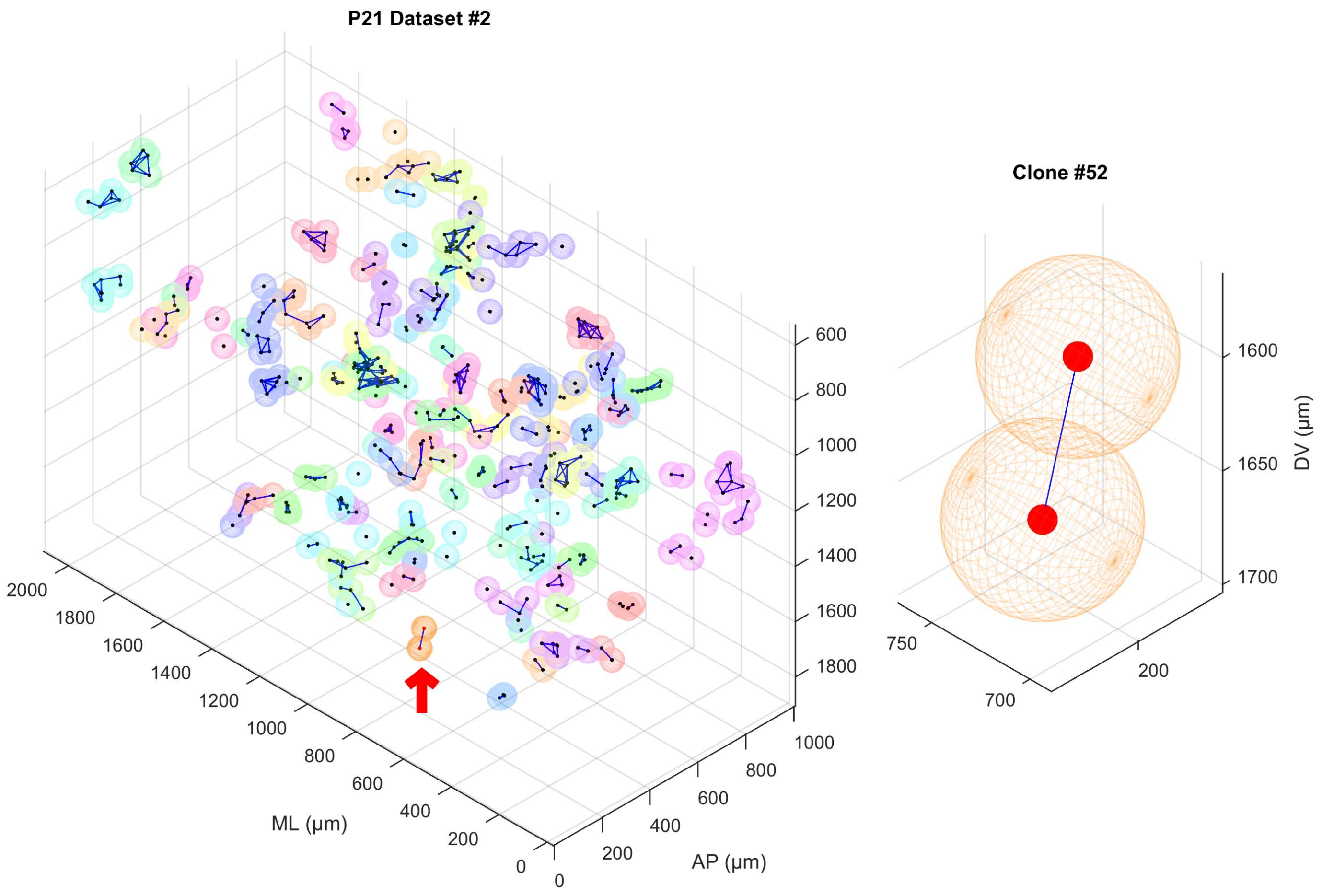

P21 Dataset #2
600
800
1000
1200
1400
1600
1800
2000
1800
1600
1400
1200
1000
800
600
400
200
0
ML (µm)
0
200
400
600
800
1000
AP (µm)
Clone #52
1600
1650
1700
DV (µm)
750
700
200

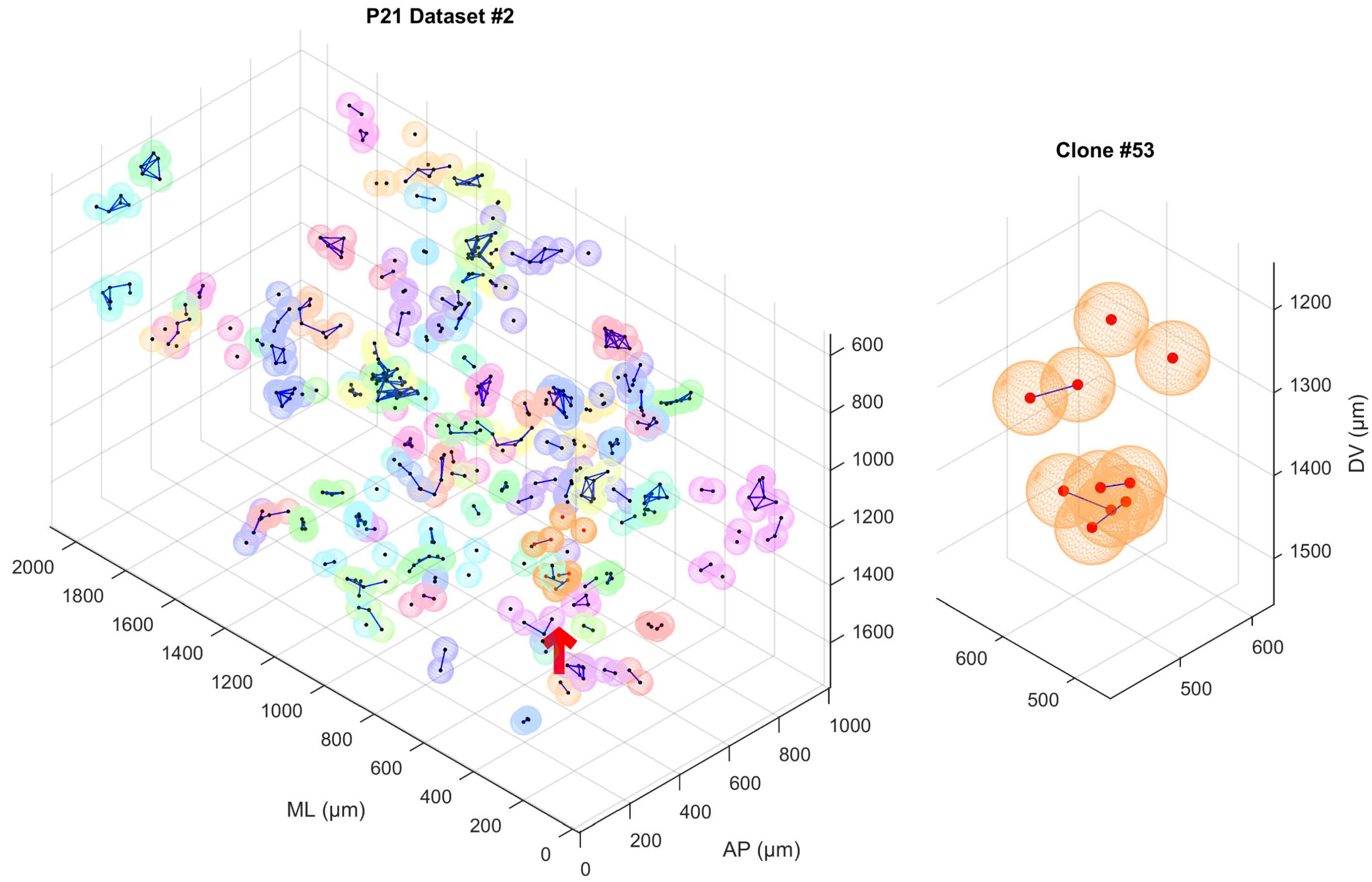

P21 Dataset #2
ML (µm)
2000
1800
1600
1400
1200
1000
800
600
400
200
0
AP (µm)
0
200
400
600
800
1000
600
800
1000
1200
1400
1600
Clone #53
DV (µm)
1200
1300
1400
1500
600
500
500
600

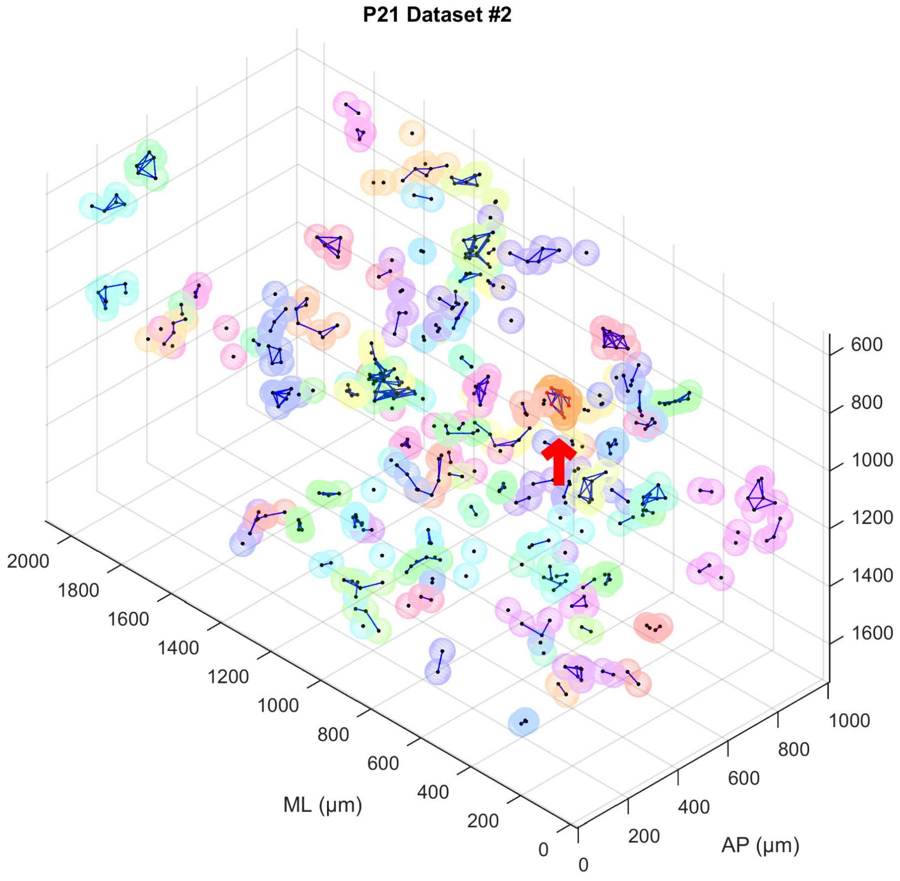

P21 Dataset #2
600
800
1000
1200
1400
1600
2000
1800
1600
1400
1200
1000
800
600
400
200
0
ML (µm)
0
200
400
600
800
1000
AP (µm)

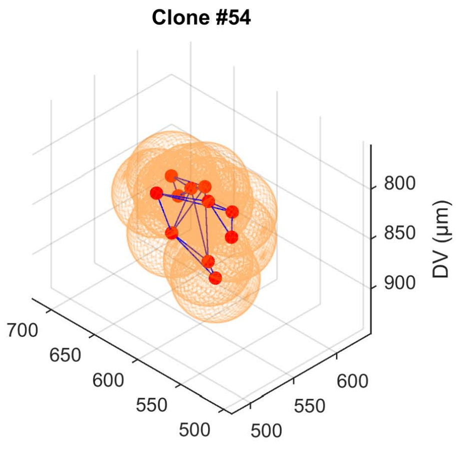

Clone #54
800
850
900
DV (µm)
700
650
600
550
500
500
550
600

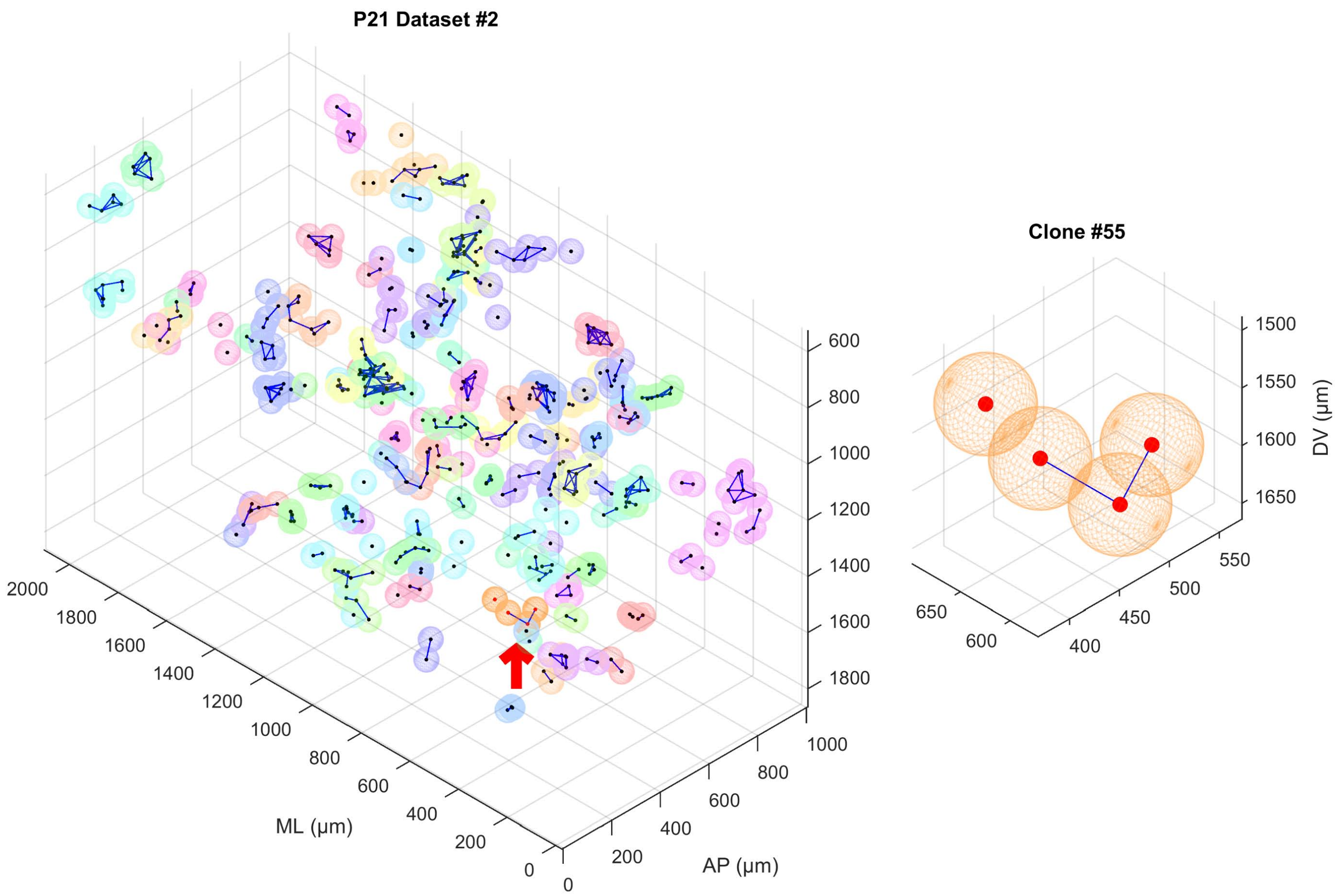

P21 Dataset #2
600
800
1000
1200
1400
1600
1800
2000
1800
1600
1400
1200
1000
800
600
400
200
0
ML (µm)
0
200
400
600
800
1000
AP (µm)
Clone #55
1500
1550
1600
1650
DV (µm)
650
600
400
450
500
550

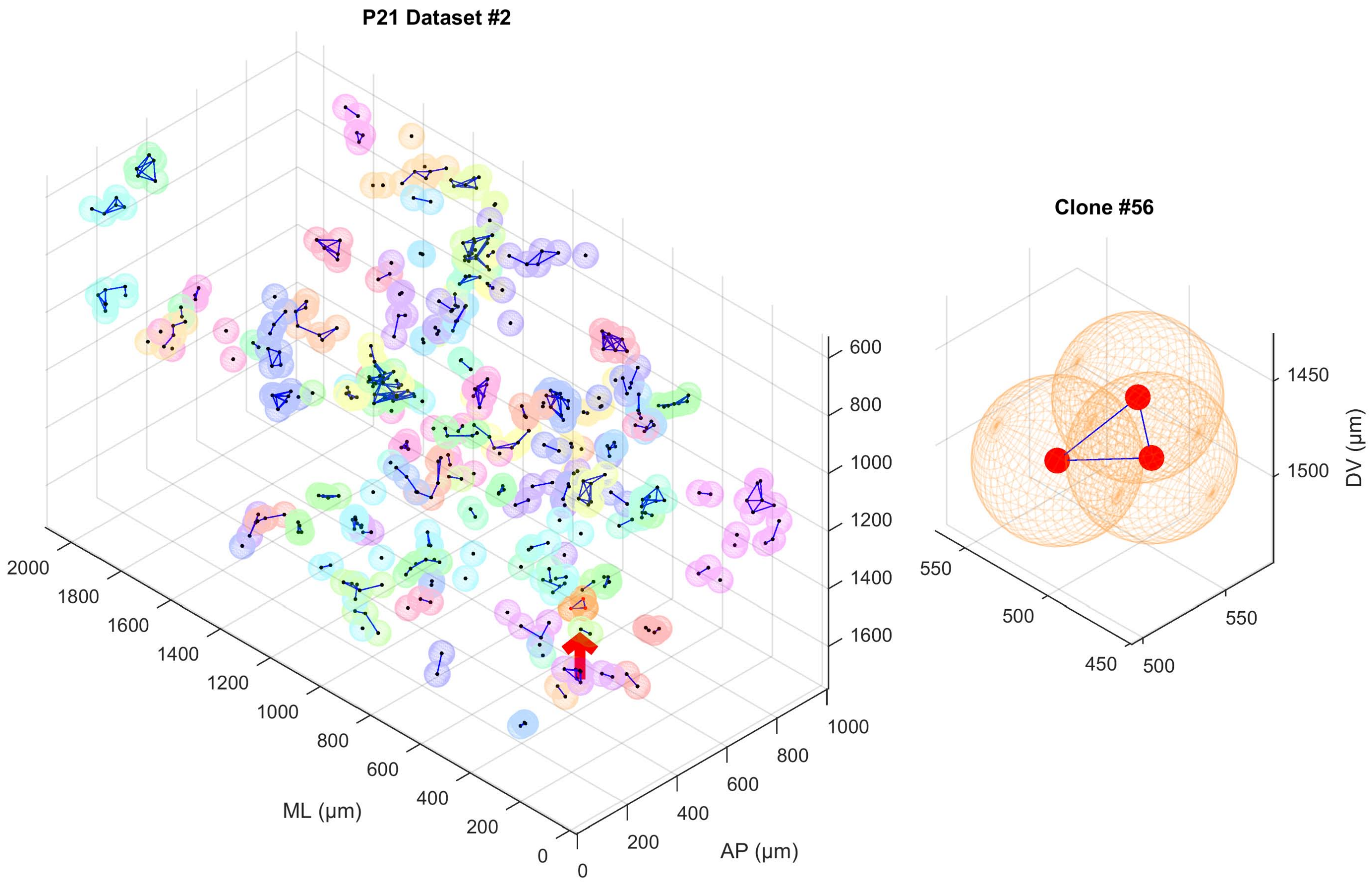

P21 Dataset #2
Clone #56
600
800
1000
1200
1400
1600
2000
1800
1600
1400
1200
1000
800
600
400
200
0
ML (μm)
0
200
400
600
800
1000
AP (μm)
1450
1500
DV (μm)
550
500
450
550
500

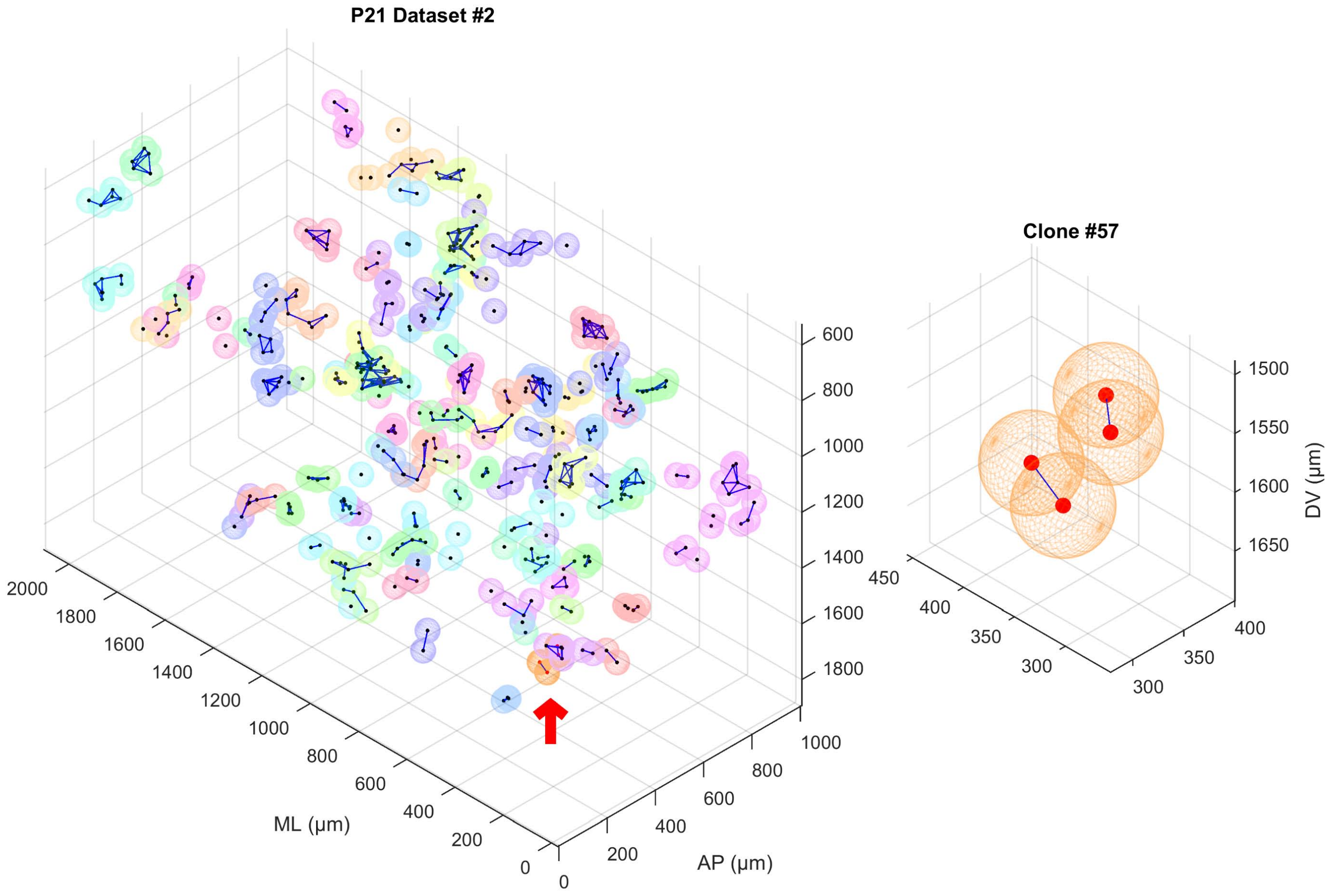

P21 Dataset #2
600
800
1000
1200
1400
1600
1800
2000
1800
1600
1400
1200
1000
800
600
400
200
0
ML (µm)
0
200
400
600
800
1000
AP (µm)
Clone #57
1500
1550
1600
1650
DV (µm)
450
400
350
300
400
350
300

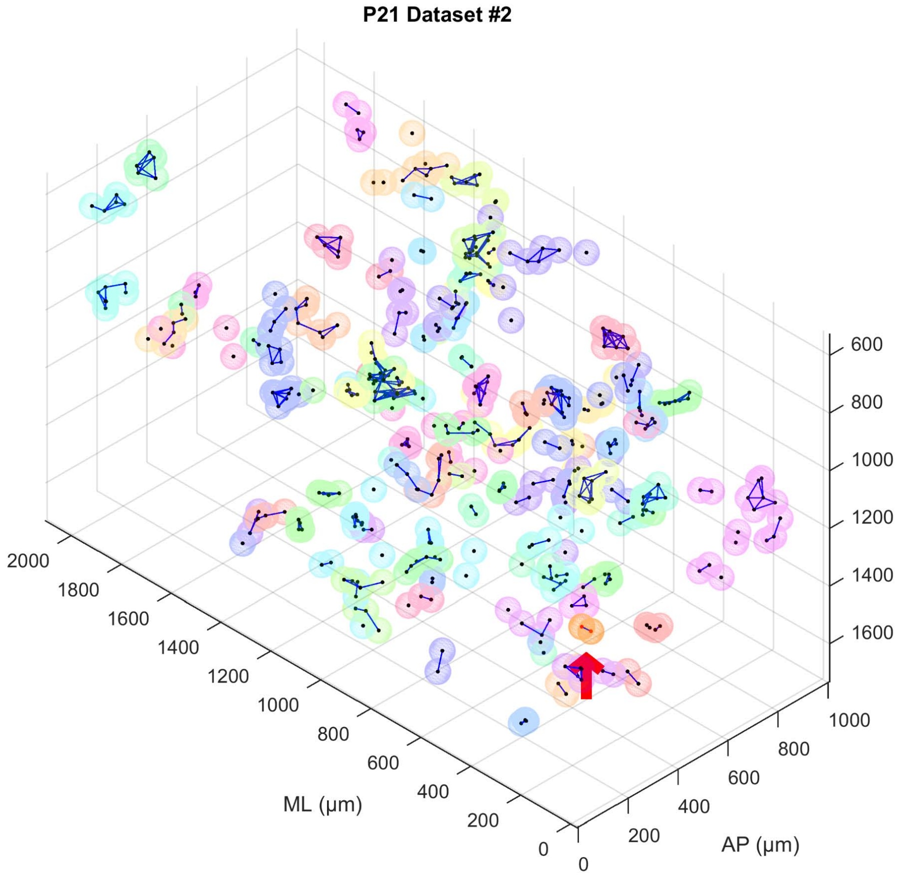
P21 Dataset #2
600
800
1000
1200
1400
1600
2000
1800
1600
1400
1200
1000
800
600
400
200
0
ML (µm)
0
200
400
600
800
1000
AP (µm)

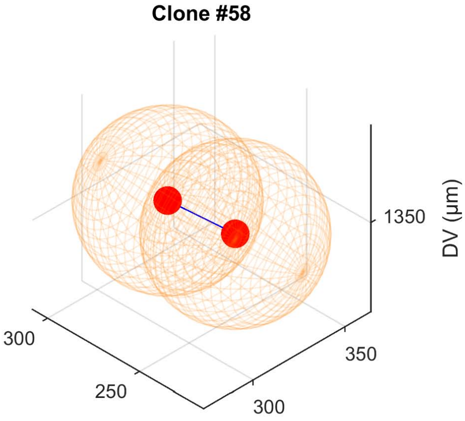
Clone #58
1350
DV (µm)
300
250
300
350

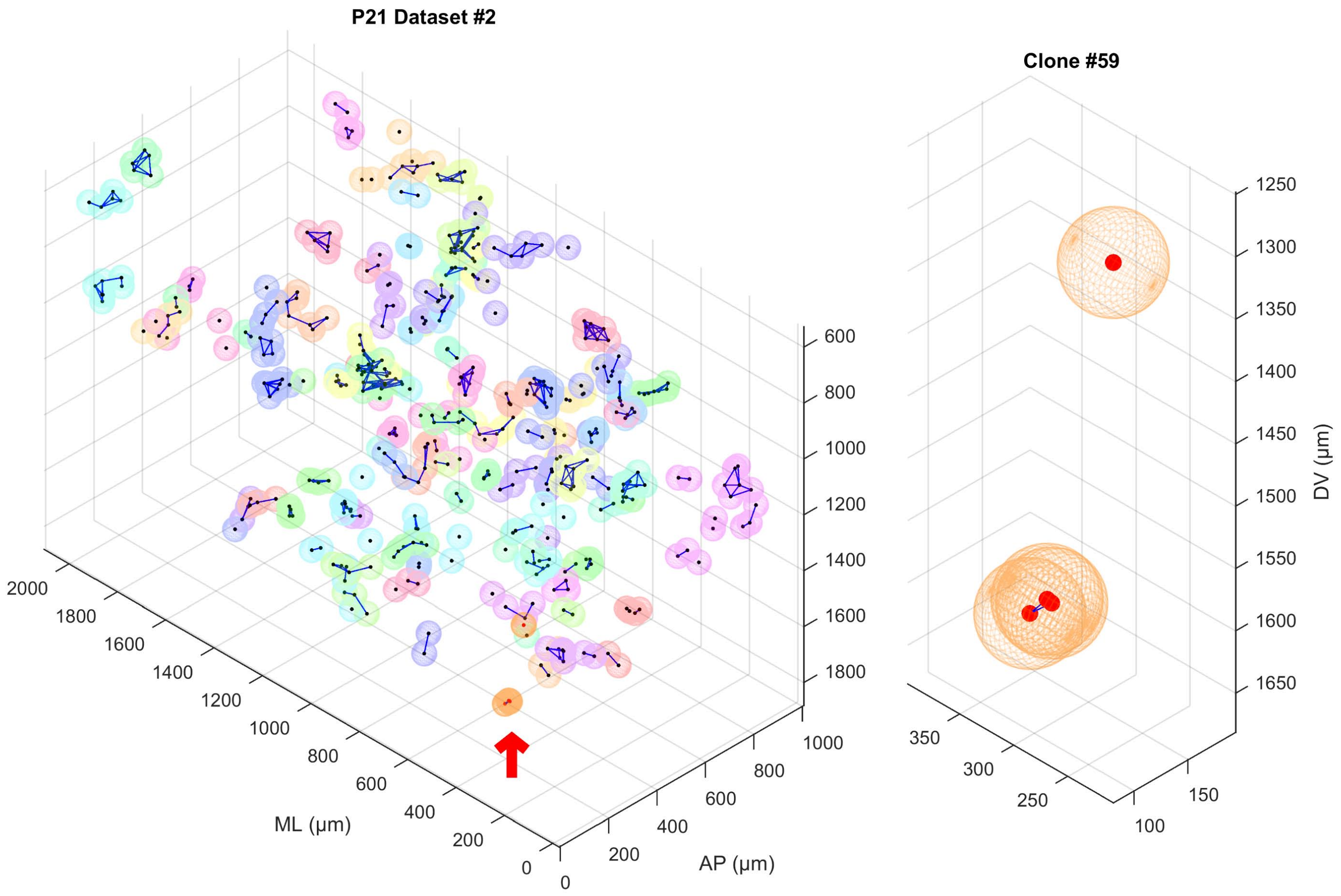

P21 Dataset #2
Clone #59
ML (µm)
AP (µm)
DV (µm)

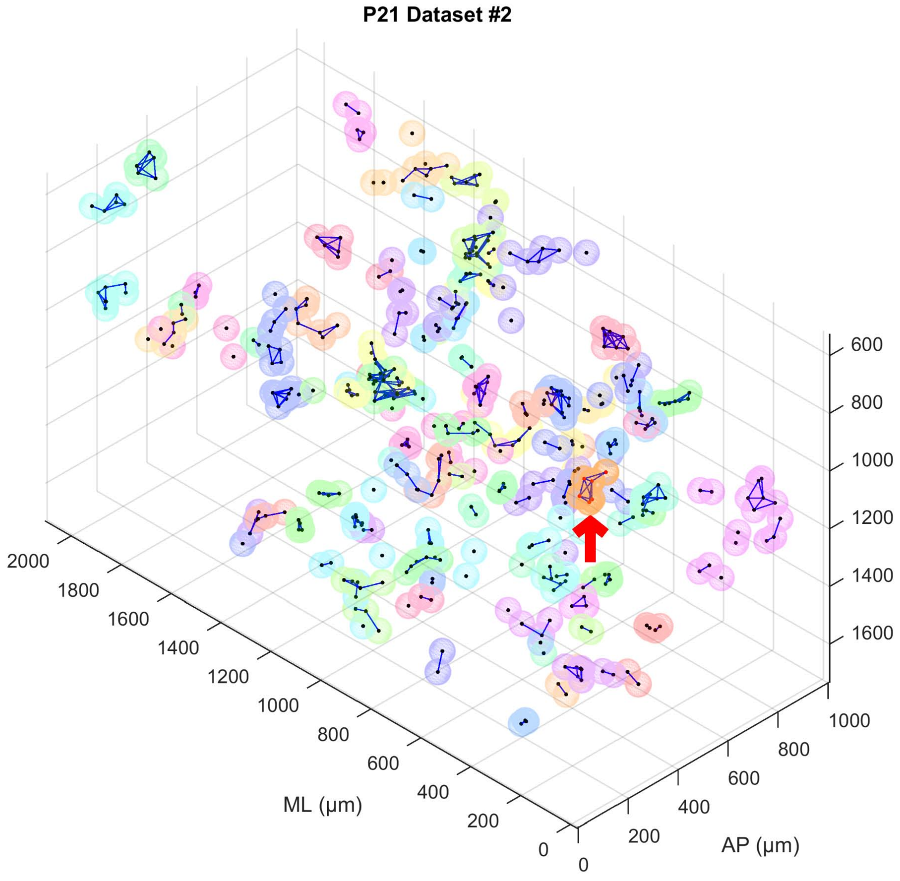

P21 Dataset #2
600
800
1000
1200
1400
1600
2000
1800
1600
1400
1200
1000
800
600
400
200
0
ML (µm)
0
200
400
600
800
1000
AP (µm)

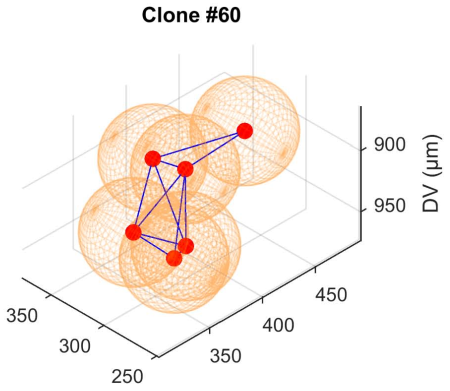

Clone #60
900
950
DV (µm)
350
300
250
350
400
450

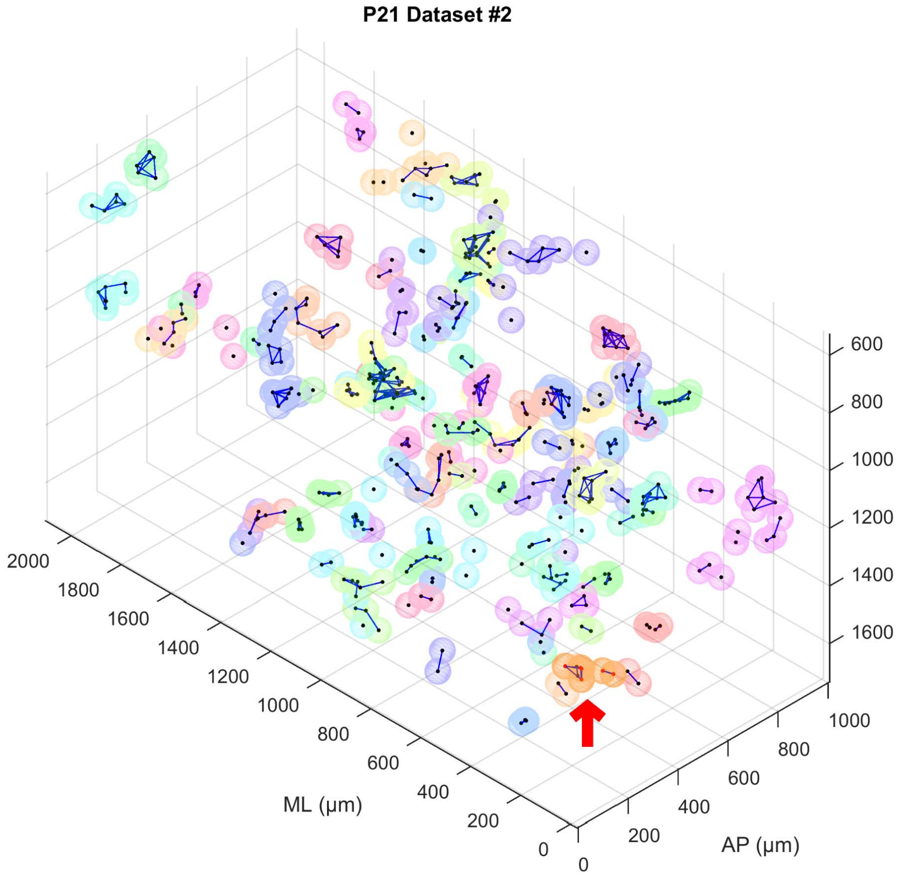
P21 Dataset #2
600
800
1000
1200
1400
1600
2000
1800
1600
1400
1200
1000
800
600
400
200
0
ML (µm)
0
200
400
600
800
1000
AP (µm)

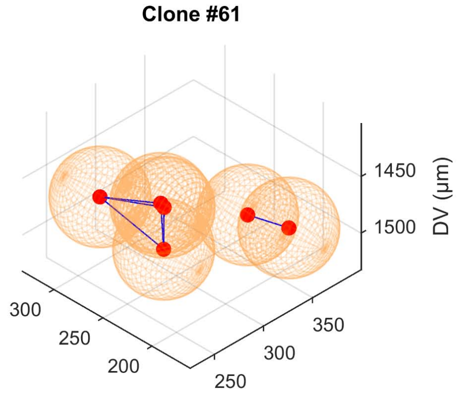
Clone #61
1450
1500
DV (µm)
300
250
200
250
300
350
